# Identification of diagnostic mRNA biomarkers in whole blood for ankylosing spondylitis using WGCNA and machine learning feature selection

**DOI:** 10.3389/fimmu.2022.956027

**Published:** 2022-09-12

**Authors:** Yaguang Han, Yiqin Zhou, Haobo Li, Zhenyu Gong, Ziye Liu, Huan Wang, Bo Wang, Xiaojian Ye, Yi Liu

**Affiliations:** ^1^Department of Orthopaedics, Shanghai Changzheng Hospital, Naval Medical University, Shanghai, China; ^2^Department of Radiology, Longhua Hospital, Shanghai University of Traditional Chinese Medicine, Shanghai, China; ^3^Department of Neurosurgery, Klinikum rechts der Isar, Technische Universität München, Munich, Germany; ^4^Department of Orthopaedics, Tongren Hospital, Shanghai Jiao Tong University School of Medicine, Shanghai, China

**Keywords:** weighted gene co-expression network analysis (WGCBA), recursive feature elimination (RFE), ankylosing spondylitis (AS), mRNAs biomarkers, support vector machine

## Abstract

Ankylosing spondylitis (AS) is a common inflammatory spondyloarthritis affecting the spine and sacroiliac joint that finally results in sclerosis of the axial skeleton. Aside from human leukocyte antigen B27, transcriptomic biomarkers in blood for AS diagnosis still remain unknown. Hence, this study aimed to investigate credible AS-specific mRNA biomarkers from the whole blood of AS patients by analyzing an mRNA expression profile (GSE73754) downloaded Gene Expression Omnibus, which includes AS and healthy control blood samples. Weighted gene co-expression network analysis was performed and revealed three mRNA modules associated with AS. By performing gene set enrichment analysis, the functional annotations of these modules revealed immune biological processes that occur in AS. Several feature mRNAs were identified by analyzing the hubs of the protein-protein interaction network, which was based on the intersection between differentially expressed mRNAs and mRNA modules. A machine learning-based feature selection method, SVM-RFE, was used to further screen out 13 key feature mRNAs. After verifying by qPCR, IL17RA, Sqstm1, Picalm, Eif4e, Srrt, Lrrfip1, Synj1 and Cxcr6 were found to be significant for AS diagnosis. Among them, Cxcr6, IL17RA and Lrrfip1 were correlated with severity of AS symptoms. In conclusion, our findings provide a framework for identifying the key mRNAs in whole blood of AS that is conducive for the development of novel diagnostic markers for AS.

## Introduction

As a kind of chronic axial spondyloarthritis, ankylosing spondylitis (AS) is characterized by aseptic sacroiliitis, spinal stiffness and deformity, ultimately leading to severe disability in patients. Due to the undefined etiology and paucity of early effective detecting methods, the diagnosis of AS is delayed for an average of 8 years (1–3). To date, human leukocyte antigen B27 (HLA-B27), C-reactive protein (CRP) and matrix metalloproteinase 3 (MMP-3), have been found to be associated with AS and positive in 85–95% of patients with AS (4, 5). However, they are also significantly positive in most patients with other immunologic disorders (6–10), indicating their insufficient diagnostic value for assessing AS activity and predicting therapeutic effectiveness. Therefore, to facilitate early diagnosis and assess AS activity, finding novel biomarkers with satisfactory sensitivity and specificity by exploring the molecular mechanisms of AS is crucial.

With the rise of high-throughput transcriptomic techniques such as microarray and sequencing, multiple bioinformatic methods have subsequently been developed and applied in the construction of gene correlation networks on a large scale to shed new light on screening key RNAs in terms of molecular interactions and the exploration of candidate biomarkers for diseases (11). Compared with other developed network analytical methods, weighted gene co-expression network analysis (WGCNA) is a novel systematic biological method that describes the correlation between the expression levels of genes with a weighted value rather than with the all-or-none dichotomy (12). Compared with analyzing single differentially expressed genes, WGCNA can cluster mRNAs into different modules that are more stable and comprehensive in reflecting the underlying pathological mechanism of transcriptomic alterations by calculating the topological parameters of gene correlations. Moreover, WGCNA reveals the correlation of each mRNA module with different clinical traits of interest, which provides more clues for identifying specific biomarkers or therapeutic targets (13).

Generally, the use of traditional experimental methods to validate the function of genes filtered by microarray and sequencing is a long process because of the large amount of data (14). Furthermore, the redundancy and collinearity of high-throughput data severely disrupt the accuracy of bioinformatic analyses. To solve this problem, many gene selection algorithms based on machine learning have been proposed to remove irrelevant or redundant information or features. Among these algorithms, recursive feature elimination based on support vector machine (SVM-RFE) is an effective tool for gene selection (15). As a backward elimination method, SVM-RFE can rank the different genes or features based on the squared sum of the feature coefficients and select the top-ranked genes that significantly influence the classification or identification of different clinical traits (16). Hence, applying SVM-RFE in identifying key mRNAs or biomarkers from transcriptomic data is promising.

To identify novel biomarkers for AS from whole blood, we utilized a microarray dataset to perform WGCNA. After generating the modules of mRNAs specific to AS, we performed gene set enrichment analysis (GSEA) with Gene Ontology (GO) on the mRNAs of these modules and then overlapped them with differentially expressed mRNAs to screen out more specific feature mRNAs to construct a protein-protein interaction (PPI) network. Based on this network, we found hub mRNAs by Cytoscape calculation. Then, we utilized SVM-RFE analysis on these hub mRNAs and screened out 13 feature mRNAs. After verification through qRT-PCR and correlation analysis, 8 key mRNAs were finally identified as the key biomarkers for AS diagnosis.

## Patients and methods

### AS patients and control group

The Ethics Committee of Shanghai Changzheng Hospital has approved this study. All included AS patients and control donors provided the informed consent including details of present study. According to the modified New York criteria (17), 40 AS patients were included in this study. In addition, 40 healthy donors were recruited in control group. The general information (age and gender), symptoms, erythrocyte sedimentation rate (ESR), C-reactive protein (CRP) and Bath Ankylosing Spondylitis Disease Activity Index (BASDAI) of patients were recorded (**Table 1**).

**Table 1:** General information of the AS patients and control donors.

	AS group (n=40)	Control group (n=40)
Age (years)	41.2±11.4	42.9±12.3
Gender(male/female)	15/6	15/5
Positive rate of HLA–B27	85.71%	N/A
Duration of back pain (months)	3.52±2.51	N/A
ESR, mm/hour	49.95±25.63	N/A
CRP, mg/L	33.27±14.86	N/A
BASDAI (10-mm VAS)	5.29±1.44	N/A

ESR, erythrocyte sedimentation rate; CRP, C-reactive protein; BASDAI, Bath Ankylosing Spondylitis Disease Activity Index; VAS, visual analog scale.

### Acquisition of microarray data and processing

The microarray dataset GSE73754 by Eric Gracey et al (18) was downloaded from the Gene Expression Omnibus (GEO) database for analysis. This dataset comprises whole blood mRNA expression data from 72 subjects (52 AS patients and 20 healthy controls). The raw data of GSE73754 were preprocessed using the “affy” and “limma” packages available from Bioconductor in R. The missing values were replenished using the k-nearest neighbor algorithm (19). The normalization of raw data was performed using the robust multiarray average algorithm (20). The batch effect was eliminated using the “sva” package of R based on the COMBAT method. Due to the public availability of relevant data, approval from a local ethics committee was not required.

### WGCNA

The “WGCNA” package of R was used for clustering modules and constructing a co-expression network. To eliminate noise and speed up the computation, the mRNAs whose variance in expression was in the top 25% of all the expression profiles were selected. The power parameter β was determined based on the function of the scale-free topology fit index. Based on the weighted Pearson correlation coefficients, an adjacency matrix was constructed to reveal unsupervised co-expression relationships between each mRNA. To simplify this step, the function “blockwiseConsensusModules” was performed with a minimum module size of 30 to construct a network and detect a consensus module. The conservation of each module was assessed using the “modulePreservation” function, which predicts the Z-score. Module-trait correlations were calculated using “modTraitCor” to detect the modules correlated with AS.

### GSEA

GSEA of GO is an effective computational method that assesses an a priori-defined set of genes enriched in specific biological states (21). GSEA was performed on the modules selected from WGCNA with the GO gene sets database (c5.all.v6.2.symbols.gmt). The cutoff criterion of the P-value was set as < 0.05.

### Identification of differentially expressed mRNAs

The screening of differentially expressed mRNAs was performed using the “limma” package of R software (version 3.6.2), and Benjamini-Hochberg adjusted P-values < 0.01 and |fold change| >1 were set as the cutoff criteria. The heatmap was visualized using the “pheatmap” package of R.

### PPI network construction and hub gene identification

The online analysis tool, Search Tool for the Retrieval of Interacting Genes/Proteins (STRING), was used to evaluate the interactions between each of the selected mRNAs. Afterwards, a PPI network was constructed using Cytoscape. The nodes’ scores of each mRNA in the PPI network were obtained by the cytoHubba plugin of Cytoscape and were defined as the criterion for further mRNA selection.

### Support vector machine based recursive feature elimination

As a powerful machine learning model, SVM has been widely applied in the functional prediction of biological molecules (22). In this study, SVM modeling was performed by using the “e1071” package of R, in which the radial basis function was the selected kernel function.

SVM-RFE is a backward feature deletion method that loops around SVM^22^. First, all of the original features are used to build the SVM learning model to obtain the absolute coefficient |w| of each input feature. Second, the features are ranked based on the square of |w|, and the bottom-ranked features are discarded. Then, the rest of the features are subject to a new loop of SVM model building and ranking with the same procedures as before. These procedures are repeated until all features are removed. The order of removed features represents the level of feature importance (23). The top-ranked features that are discarded later are deemed to be more informative than those that are discarded earlier. In this study, the features correspond to mRNAs. To determine how many top-ranked mRNAs should be selected, 5-fold cross-validation was performed on the dataset. This method randomly divides the dataset into 5 sections, of which 4 sections are selected as the training set, with the last section as the testing set. Depending on these sets, SVM is built with different numbers of top mRNAs for calculating the generalized prediction error. These procedures are repeated 5 times. Finally, the number of top-ranked mRNAs corresponding to the minimum error is the optimal number of selected mRNAs. Using the “pROC” package of R, receiver operating characteristic (ROC) curve analysis was performed to calculate the area under the curve (AUC) value for each selected feature mRNA to evaluate its predictive capability for the diagnosis of AS.

### Validation of mRNA expression

5 ml of whole blood was drawn into an EDTA tube from AS patients before medical interventions. Ficoll was used to separate mononuclear cells from whole blood. The total RNA was isolated from mononuclear cells by using TRIzol LS reagent (Ambion). The extracted RNA was used to synthesize cDNA with a Reverse Transcription kit (Takara). The expression of RNAs was firstly determined by 1.5% agarose gel electrophoresis. Electrophoresis was performed at a constant voltage of 100 V for 30 min in TBE running buffer, and the retardation of RNA mobility was visualized under UV light. Quantitative real-time PCR (qRT-PCR) was performed using SYBR Green qPCR Master Mix (Takara) in qPCR CFX 96 Thermocycler system (Bio-Rad). The primers for each selected mRNAs were listed in **Supplementary Table S1**. The reactions were run according to the following conditions: initial hold at 95°C for 10 min, followed by 40 cycles of amplification at 95°C for 15 s, and annealing for 60s at 60°C and drawing the melting curves by increasing from 60°C to 95°C (0.3°C per second). All expression values were normalized to the expression of GAPDH. Relative expression levels are obtained by calculating 2^-ΔΔCT^.

### Statistical analysis

The statistical analysis was performed with R software (version 3.6.2). The continuous variables were presented with Mean ± SD, while the categorical variables were presented with quartile. The expression values of mRNAs were compared by using one-way analysis of variance (ANOVA) between AS group and control group. Correlation between expression of mRNAs and BASDAI was evaluated by using Pearson’s correlation coefficient test. The P<0.05 was selected as the cut-off for statistical significance.

## Results

### Generation of key modules associated with AS by WGCNA

**Figure 1:**
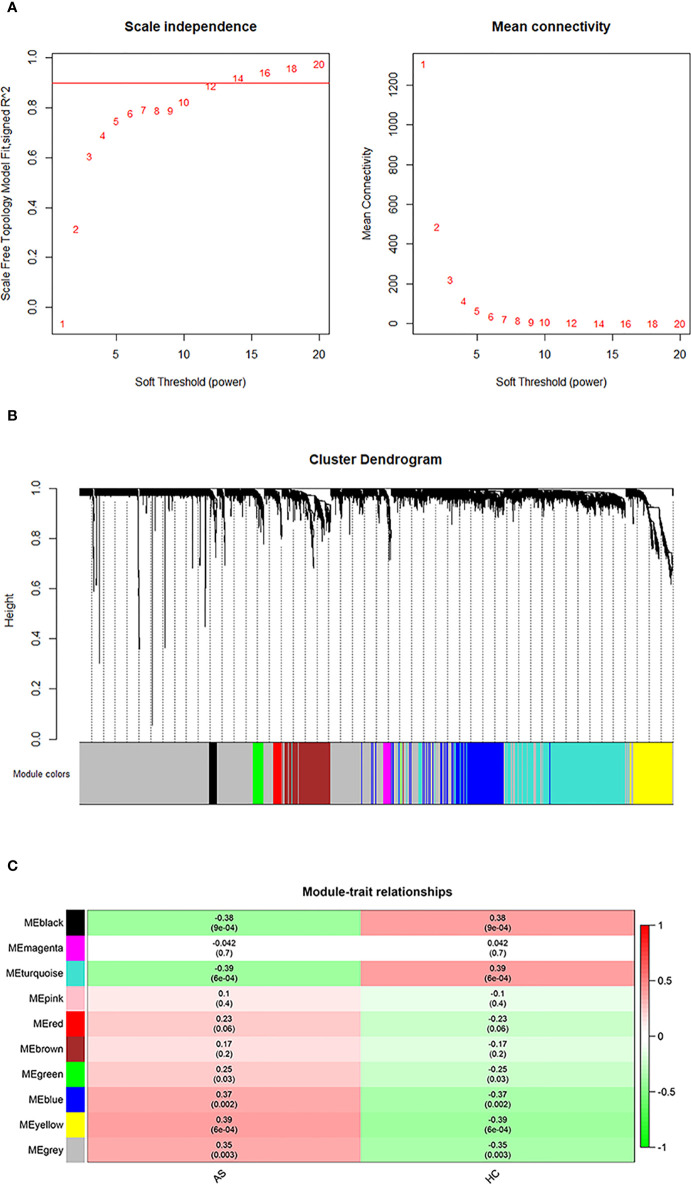
WGCNA analysis. **(A)** Determination of an optimal soft-thresholding power β by calculating the scale-free topology mode fit and mean connectivity. **(B)** The cluster dendrogram of mRNAs in GSE73754, revealing different mRNA co-expression modules marked with colors. **(C)** The heatmap for module-traits relationships, in which the correlation of different modules with AS or HC, P-values are presented in each cell.

The initial step was to generate consensus modules of mRNA expression by constructing a weighted gene co-expression network. We made hclust analysis, with height 45 as cutoff. There was no outlier in included samples (**Supplementary Figure S1**). The determination of the soft thresholding power β is entailed in raising Pearson correlation matrices to obtain the network (24). According to the criterion of approximate scale-free topology, in which the scale-free topology model fit index was more than 0.9 and the mean connectivity degree was close to 0, the optimal power β was chosen to be 14 (**Figure 1A**). Afterwards, the weighted co-expression networks were constructed, and consensus modules with similar expression trends were clustered and labeled with different colors, as shown in a dendrogram (**Figure 1B**). Then, the correlation matrices between consensus modules and clinical traits (AS and HC) were calculated (**Figure 1C**). Based on the cutoff of 0.3 to correlation, the Blue, Yellow and Gray modules with specific relation to AS were selected for further investigation. There were 463 mRNAs in the Blue module, 318 mRNAs in the Yellow module, and 404 mRNAs in the Gray module, of which information about the network is presented in **Supplementary Table S2**. In addition, we performed correlation analysis of Module Membership vs. Gene Significance, and found significant correlation coefficients were 0.28 in Blue module, 0.44 in Grey module, and 0.38 in Yellow module, respectively (**Supplementary Figure S2**).

### GSEA with GO on selected modules

**Figure 2:**
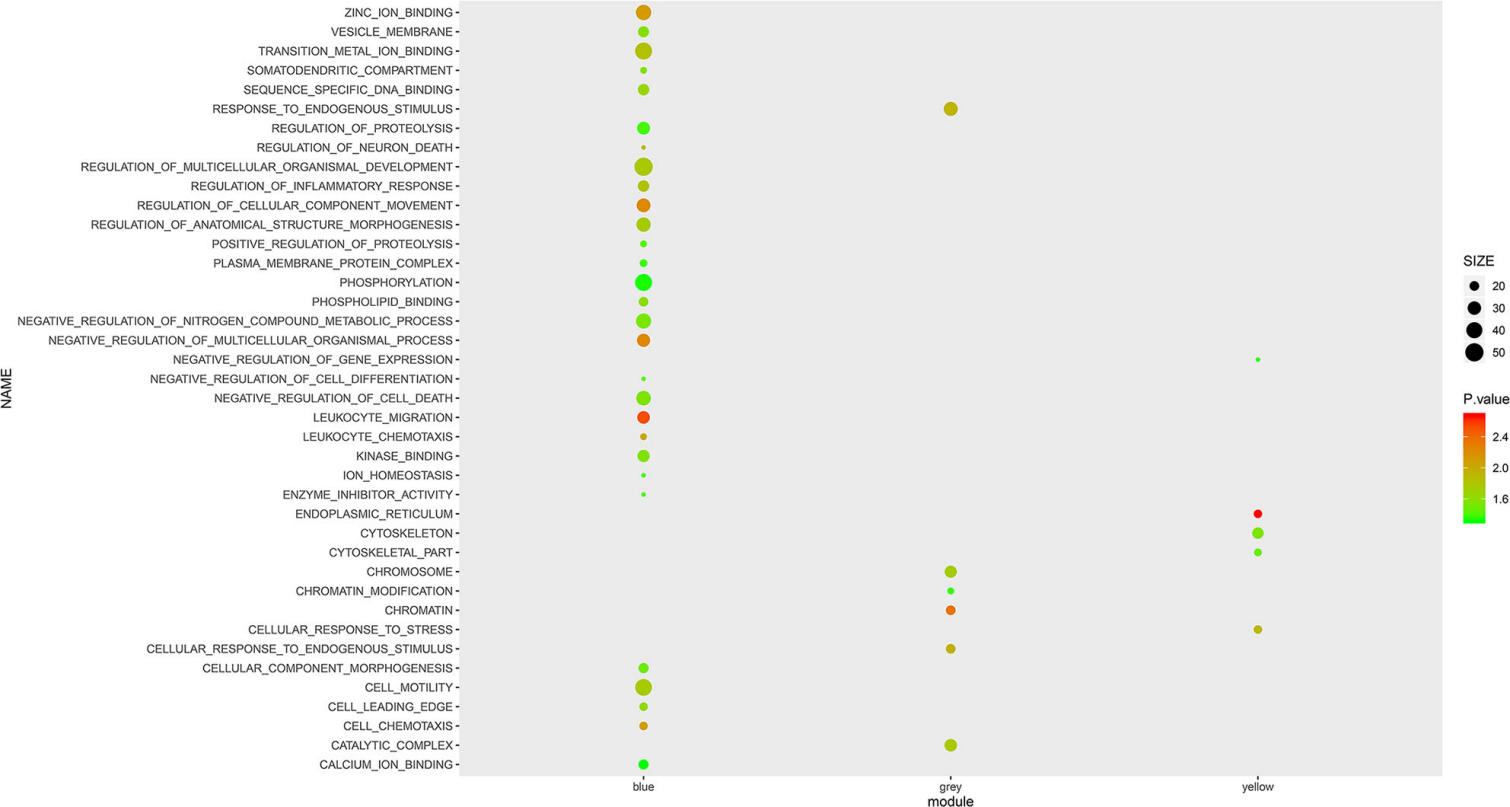
GSEA with GO terms on Blue, Yellow and Grey modules. The size and color intensity of a circle represent the numbers of mRNAs and −log_10_ (P value) of enrichment for each module.

To further investigate the role of the selected mRNA modules and pathological processes in white blood cells, we performed GSEA with GO terms on mRNAs of the Blue, Yellow and Gray modules. As shown in **Figure 2**, mRNAs in the Blue module were enriched in the top 10 GO terms with the lowest normalized P-value, including “leukocyte chemotaxis”, “leukocyte migration”, “cell chemotaxis” and “regulation of inflammatory response”, which implicated active inflammatory and immune responses in AS patients’ blood. However, in contrast to the Blue module, most GO terms enriched by the mRNAs in the Yellow and Gray modules are unspecific to AS activity, except for “leukocyte cell adhesion”, suggesting that these two modules may represent secondary pathological processes of AS. Therefore, it can be inferred that mRNAs in the Blue module exert more imperative effects than those in the Yellow and Gray modules and are immune dysregulated by AS activity.

### Screening of differential expressed mRNAs

To further investigate the discrepancy in whole blood between AS and HC, we filtered differentially expressed mRNAs. A total of 1116 mRNAs were differentially expressed, among which 491 mRNAs were upregulated and 625 mRNAs were downregulated (**Supplementary Table S3**). Next, we constructed a heatmap for the top 100 most differentially expressed mRNAs to show the consistencies and discrepancies in mRNA expression among the samples. As shown in **Supplementary Figure S3**, most AS blood samples are clustered together with similar expression tendencies, which means that their expression patterns differ from the patterns of HC samples.

### Selection of feature mRNAs from modules and differential expressed mRNAs

To obtain comprehensive information from the whole blood mRNA expression of AS, finding a balance between WGCNA modules and differentially expressed mRNAs is critical. Accordingly, we overlapped 1185 mRNAs from the Blue module, Yellow module and Gray module with 1116 differentially expressed mRNAs and screened out 296 feature mRNAs for AS. The intersection of each module with differentially expressed mRNAs is shown in **Supplementary Table S4**.

### Construction of PPI network based on feature mRNAs

Given the interaction between key genes in various pathological processes, performing interaction network analysis on mRNA groups is effective for identifying candidate biomarkers. To this end, we constructed a PPI network on the 296 feature mRNAs by STRING (**Supplementary Figure S4**). A total of 427 protein interactions and 280 gene nodes were identified in this network with an enrichment P-value of 5.26e-07.

In the expression network, hub genes are a series of key genes that have great topological connectiveness with their neighboring genes. To distinguish the hub genes in a network, Closeness Centrality (CC) and Betweenness Centrality (BC), which are based on a concept of moving along the most optimal and shortest paths throughout a network, are widely used in network analysis (25). Because of the vague principles of the usage of these 12 parameters, we simultaneously applied all of them to measure the connectiveness of mRNAs in the PPI network. After inputting the data of the PPI network into Cytoscape and calculating each nodes’ scores through cytoHubba, we sorted feature mRNAs by 12 nodes’ scores in descending order and generated 12 sequences of mRNAs. Then, we selected the top 25% mRNAs from these 12 sequences and converted these selected mRNAs together. Finally, according to the occurrence of mRNAs in each sequence, 63 mRNAs appearing more than 4 times were obtained as the hub genes (**Supplementary Table S5**). The interaction network of these feature mRNAs is shown in **Supplementary Figure S5**.

### Identification of key mRNAs by SVM-RFE

**Figure 3:**
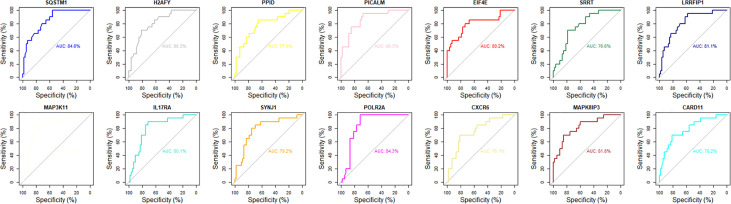
The ROC curve analysis of 14 key mRNAs in diagnostic specificity for AS.

Although the 63 selected feature mRNAs can serve as biomarkers for AS, there is still much redundant information in them, resulting in poor feasibility in practical applications. To solve this problem, we applied SVM-RFE according to the feature ranking of the correlation coefficients to eliminate relatively unspecific feature mRNAs and preserve the key mRNAs. To determine the optimal number of feature mRNAs with the greatest accuracy in the SVM model, 5-fold cross-validation was introduced into the SVM classifier step, and the error rates of different numbers of mRNAs were captured. We plotted the change in the 5-fold cross-validation error rate at each recursive step (**Supplementary Figure S6**). The error rate fluctuated with increasing numbers of mRNAs until it reached a minimum with 14 feature mRNAs, suggesting that discrimination between AS and HC reached almost 90% accuracy. ROC curve analysis was further carried out, and the AUC values of the 14 key mRNAs were calculated to reveal their predictive power (**Figure 3**). Accordingly, MAP3K11 was discarded because of its nonsignificant predictive power in distinguishing between AS and HC. Among the 13 remaining selected feature mRNAs, Sqstm1, Srrt, Cxcr6, Eif4e, Ppid, H2afy, Card11, IL17ra, Picalm, Lrrfip1, Polr2a, Mapk8ip3 and Synj1 were screened out as the key mRNAs of AS for further analysis.

### Validation of key mRNAs expression

**Figure 4:**
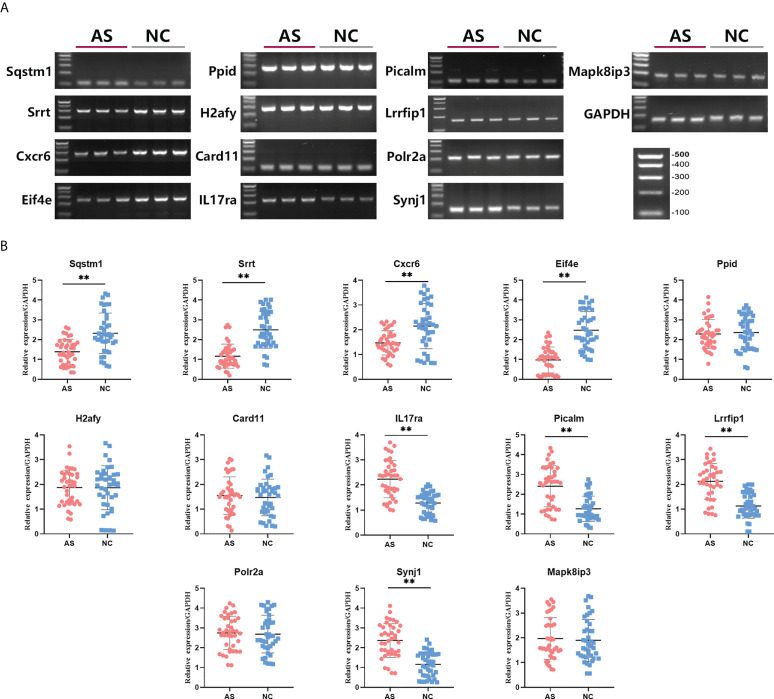
Differences in relative expression level of 13 key mRNAs between AS group and control group. Agarose electrophoresis **(A)** and qRT-PCR quantification **(B)** for Sqstm1, Srrt, Cxcr6, Eif4e, Ppid, H2afy, Card11, IL17ra, Picalm, Lrrfip1, Polr2a, Synj1 and Mapk8ip3. ** means P-value < 0.01.

To verify the prediction of bioinformatic and SVM analysis, we performed qRT-PCR and agarose gel electrophoresis to test the expression levels of these 12 key mRNAs in whole blood of AS group and control group. As shown in **Figure 4**, the expression of Sqstm1, Srrt, Cxcr6, and Eif4e were significantly down-regulated in AS patients, while the expression of IL17ra, Picalm, Lrrfip1 and Synj1 were significantly up-regulated compared with control group. In addition, there were no significant differences on the expression of Ppid, H2afy, Card11, Mapk8ip3 and Polr2a between two groups. These results indicated the expression patterns of 8 significant key mRNAs in included patients were consistent with bioinformatic analysis and SVM prediction.

### Correlating analysis between BASDAI and expression of key mRNAs

**Figure 5:**
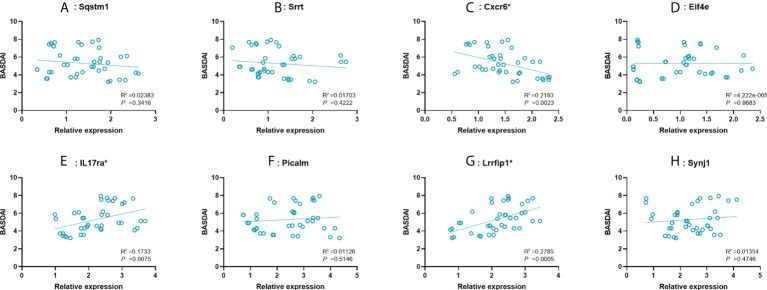
Correlation between expression value of 8 significant mRNAs and BASDAI. **(A–H)**: Sqstm1, Srrt, Cxcr6, Eif4e, IL17ra, Picalm, Lrrfip1, Synj1. R^2^, correlation coefficient. BASDAI, Bath Ankylosing Spondylitis Disease Activity Index; VAS, visual analog scale.

To further examine the predictive strength of 8 significant key mRNAs, we analyzed the correlation between their expression levels and BASDAI of AS patients. In a total of 40 blood samples from AS group, a significant correlation between BASDAI and expression level was revealed in three key mRNAs (Cxcr6, IL17ra, Lrrfip1), while the remaining 5 mRNAs showed no significant correlation with BASDAI (**Figure 5**). There, Cxcr6, IL17ra, Lrrfip1 were proposed to serve as the potential biomarkers for AS.

## Discussion

While HLA-B27 has been demonstrated to mainly account for the genetic effects of AS, the other undefined markers may be associated with this immunologic disease (4, 26, 27). People with positive HLA-B27 have a significantly higher risk of developing AS than those with negative HLA-B27. However, most of the former remain healthy, implying that in addition to HLA-B27, other potential factors may contribute to the onset of AS (28, 29). Hence, elucidating AS pathogenesis from the perspective of immune regulation, especially associated with blood karyocytes, can be regarded as a promising direction for finding diagnostic biomarkers with reliable specificity and sensitivity beyond HLA-B27. In present study, we explored the microarray dataset of GSE73754 by WGCNA and PPI network construction, and then identified 3 modules (Blue, Yellow and Gray) and 63 hub mRNAs.

Several studies have demonstrated the pivotal role of adaptive immune responses in AS pathogenesis (30). The interaction between CD4^+^ T cells and HLA-B27 triggers the cascade reaction of various chemokines and cytokines, contributing to inflammatory damage and bone erosion in AS (31). In addition to the adaptive immune response, innate immune abnormalities also contribute to the initiation of AS (32). In AS, Tumor necrosis factor (TNF) mediates the destabilization of bone morphogenetic signaling proteins in osteoblasts and inhibits the expression of insulin-like growth factor-1, osterix and Runx2, resulting in poor osteoblastogenesis (33–35). Consistent with the preceding findings, the GSEA results of this study regarding GO terms in the Blue module showed the involvement of inflammatory and immune responses in AS, further verifying the imperative role of immune dysregulation in AS progression. However, the results of GO enrichment in Yellow and Gray modules revealed a negative relationship with immune response. Although the mRNAs in the Yellow and Gray modules seem to reflect uncorrelated effects with immune responses, the possibility of their synergism with the immune response cannot be ruled out and needs to be further explored.

In analyzing thousands of gene expression data through bioinformatic method, the “curse of dimensionality” cannot be denied which severely impairs the accuracy of classification and prediction. To reduce the dimensionality, wrapper methods have been developed to be incorporated into a machine learning algorithm, which evaluate the values of different features according to the pre-estimated errors (36). SVM-RFE, as a novel established wrapper method for feature selection, can refine the optimum feature by ranking the coefficients of different features obtained by SVM (23). This is because the rank of each coefficient indirectly reflects the orthogonal degree between the feature and hyperplane generated by SVM. The orthogonality of a feature to the hyperplane signifies that this feature is more informative than others (23). In this study, we used a PPI network to identify 63 hub mRNAs that are already highly correlated with AS. However, to some extent, using these 63 mRNAs as biomarkers for further prediction is also a kind of high-dimensional modeling, which likewise encounters overfitting or other high-dimensional challenges. Therefore, to address these problems, we utilized SVM-RFE and optimally selected 13 out of the 63 feature mRNAs based on a 5-fold cross-validation error rate. Moreover, ROC curves were subsequently plotted and reflected the significant specificities of these 13 key mRNAs for recognizing AS. Then, 8 of 13 key mRNAs (Sqstm1, Srrt, Cxcr6, Eif4e, IL17ra, Picalm, Synj1 and Lrrfip1) in AS blood sample showed significant consistence with microarray data in qRT-PCR validation, and 3 of them (Cxcr6, IL17ra, Lrrfip1) were correlated with symptomatic severity of AS, indicating the efficacy of SVM screening combined with bioinformatics.

IL-17ra is one of five well-known receptor subtypes for IL-17 ligands. When bound by IL-17, this receptor upregulates the expression of various cytokines and chemokines to exert a proinflammatory role in host defense. In whole blood, IL-17 and its receptors are mainly expressed in Th17 cells and neutrophils and were demonstrated to play a pivotal role in AS patients (37–39). Evidence suggests that the binding of IL17 to its receptor triggers several feedback-loop mechanisms in spondyloarthritis, resulting in the proliferation of Th17 cells, thereby causing increased production of IL-17 (40). This was further highlighted by the significant remission of AS symptoms after the application of inhibitory medication targeting IL-17 pathways (41, 42). In addition to Il-17RA, the downregulation of Sqstm1 in whole blood may be related to AS. As a kind of ubiquitin binding protein, Sqstm1 is reduced when autophagy is activated, which subsequently increases the level of IL23 in the intestinal mucosal surfaces of AS patients (43). Intriguingly, thus far, there is no robust proof to verify the direct involvement of the other significant feature mRNAs (Cxcr6, eIF4E, Lrrfip1, Srrt, Synj1 and Picalm) in AS pathogenesis. Cxcr6, eIF4E, and Lrrfip1, were found to be related to innate or adaptive immune processes. C-X-C Motif Chemokine Receptor 6 (CXCR6), a kind of chemokine receptor, is mainly expressed on the CD4+ T cell surface and mediates a series of immune cellular activation and chemotaxis events (44). Eukaryotic translation initiation factor 4E (eIF4E) is mainly expressed in macrophages and activated following the stimulation of LPS, leading to the upregulation of IκBα, which inhibits the expression of inflammatory cytokines and genes (45). LRR Binding FLII Interacting Protein 1 (LRRFIP1) was found to be involved in the innate defense against pathogenic organisms and in the regulation of autoimmune disorders (46). In our study, upregulated IL-17RA and Cxcr6 were found to be positively correlated with BASDAI, while downregulated Lrrfip1 was negatively correlated, implying the potential of IL-17RA, Cxcr6 and Lrrfip1 in predicting AS symptom. In addition, the biological function of Srrt, Synj1 and Picalm has not been shown to be specific to AS, even though they are significant differential expressed in AS patients. But this does not mean that they are unqualified to serve as biomarkers. Their correlations with AS need further investigation to be elucidated in the future.

Undeniably, there was an inevitable limitation in our study. Because of the shortage of a proper microarray dataset for the whole blood of AS patients, there were not sufficient samples for randomly selecting and establishing a training set and testing set for machine learning, so we were incapable of further verifying the efficacy of the SVM classifier made of feature mRNAs. Further studies are expected to include more available datasets and verify the accuracy of prediction.

In summary, this study reveals that IL17RA, Sqstm1, Picalm, Eif4e, Srrt, Lrrfip1, Synj1, Cxcr6 can be seen as potential predictors for AS. These mRNAs may function *via* involvement in various pathways of AS, especially in immune-related pathways. Exploration of their function in AS pathology may be beneficial for the diagnosis of AS.

## Data availability statement

The datasets presented in this study can be found in online repositories. The names of the repository/repositories and accession number(s) can be found in the article/**Supplementary Material**.

## Ethics statement

The studies involving human participants were reviewed and approved by The Ethics Committee of Shanghai Changzheng Hospital. The patients/participants provided their written informed consent to participate in this study. Written informed consent was obtained from the individual(s) for the publication of any potentially identifiable images or data included in this article.

## Author contributions

YL, YH, ZG, and XY were involved in the concept and design of the study. YL, YH, and YZ drafted the manuscript. YL, YH, YZ, HL, ZG, ZL, HW, BW and XY were involved in analysis and interpretation of the data and revision of the manuscript.

## Funding

This study was supported by the National Key R&D Program of China (2020YFC2008404).

## Conflict of interest

The authors declare that the research was conducted in the absence of any commercial or financial relationships that could be construed as a potential conflict of interest.

## Publisher’s note

All claims expressed in this article are solely those of the authors and do not necessarily represent those of their affiliated organizations, or those of the publisher, the editors and the reviewers. Any product that may be evaluated in this article, or claim that may be made by its manufacturer, is not guaranteed or endorsed by the publisher.

## Abbreviations

AS, ankylosing spondylitis; HlA-B27, human leukocyte antigen B27; GEO, Gene Expression Omnibus; GO, Gene Ontology; HC, healthy control; WGCNA, weighted gene co-expression network analysis; GSEA, gene set enrichment analysis; PPI, protein-protein interaction; STRING, Search Tool for the Retrieval of Interacting Genes/Proteins; MMP-3, matrix metalloproteinase 3; SVM; support vector machine; RFE; recursive feature elimination, ROC, receiver operating characteristic; APCs, antigen presenting cells; TNF, tumor necrosis factor; CXCR6, C-X-C Motif Chemokine Receptor 6; eIF4E, eukaryotic translation initiation factor 4E; LRRFIP1, LRR Binding FLII Interacting Protein 1; MAPK8IP3, mitogen-activated protein kinase 8 interacting protein 3; ESR, erythrocyte sedimentation rate; CRP, C-reactive protein; BASDAI, Bath Ankylosing Spondylitis Disease Activity Index; VAS, visual analog scale.
