# Supplementary material for: Identification of diagnostic mRNA biomarkers in whole blood for ankylosing spondylitis using WGCNA and machine learning feature selection
Source: Front Immunol. 2022 Sep 12;13:956027. doi: 10.3389/fimmu.2022.956027 (PMC9510835; doi:10.3389/fimmu.2022.956027)
Supplement: Supplementary file 1 [file DataSheet_1.pdf]

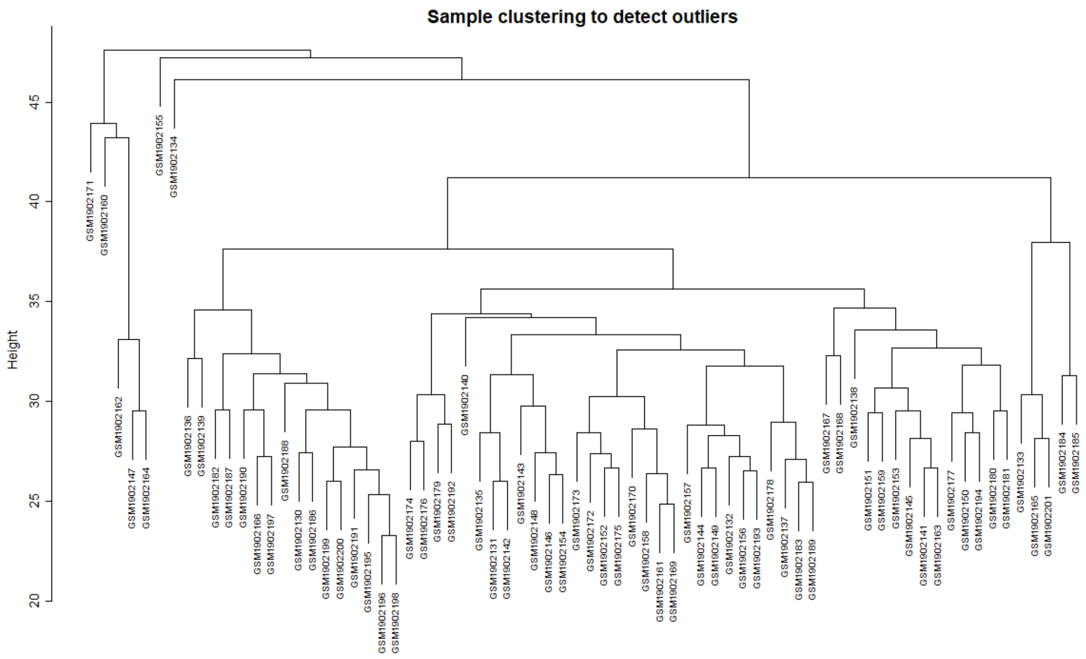


Supplementary Figure 1. Hclust analysis.

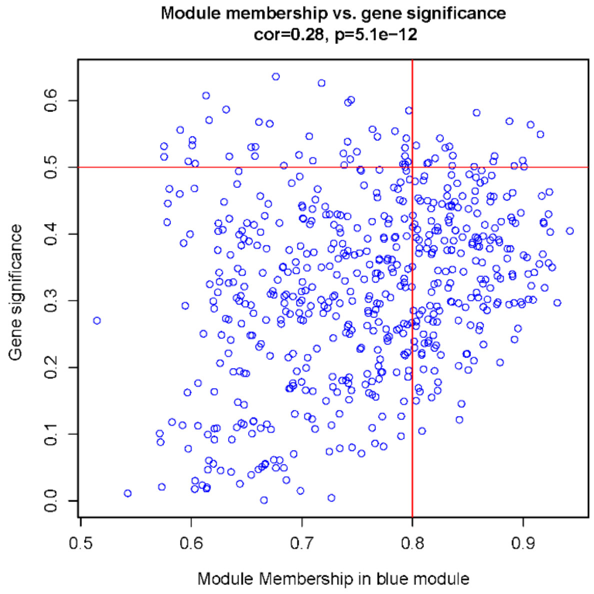
Module membership vs. gene significance
cor=0.28, p=5.1e−12
Gene significance
Module Membership in blue module
0.0
0.1
0.2
0.3
0.4
0.5
0.6
0.5
0.6
0.7
0.8
0.9

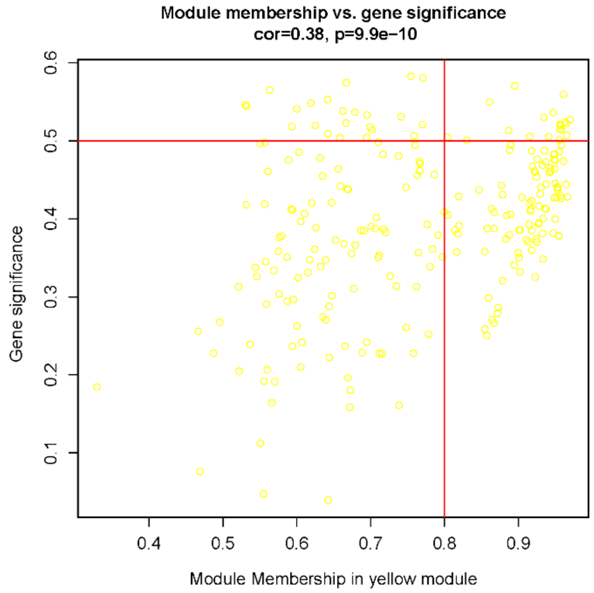
Module membership vs. gene significance
cor=0.38, p=9.9e−10
Gene significance
Module Membership in yellow module
0.1
0.2
0.3
0.4
0.5
0.6
0.4
0.5
0.6
0.7
0.8
0.9

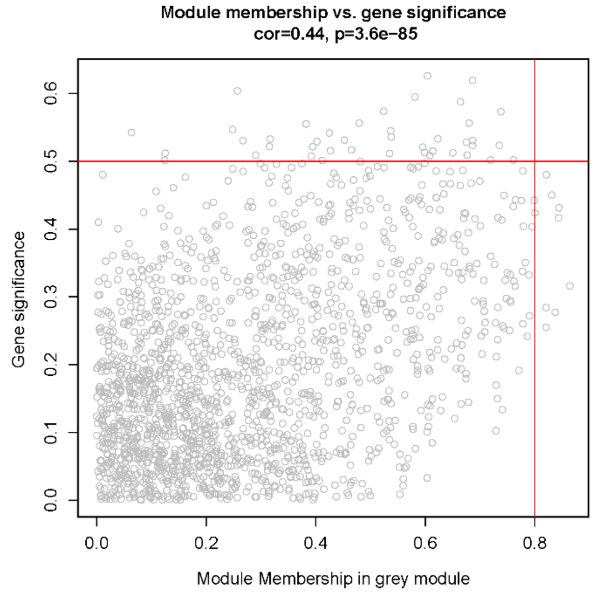
Module membership vs. gene significance
cor=0.44, p=3.6e−85
Gene significance
Module Membership in grey module
0.0
0.1
0.2
0.3
0.4
0.5
0.6
0.0
0.2
0.4
0.6
0.8

Supplementary Figure 2. Correlation analysis of Module Membership vs. Gene Significance.

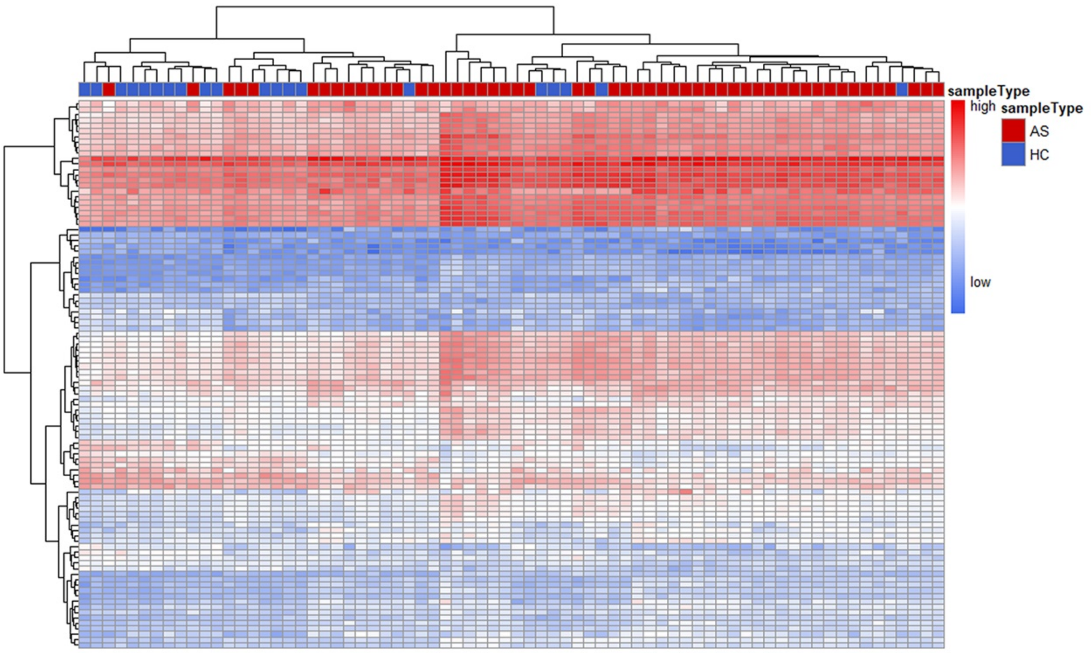


Supplementary Figure 3. Heatmap of the differentially expressed mRNAs with the top 100 greatest fold change.

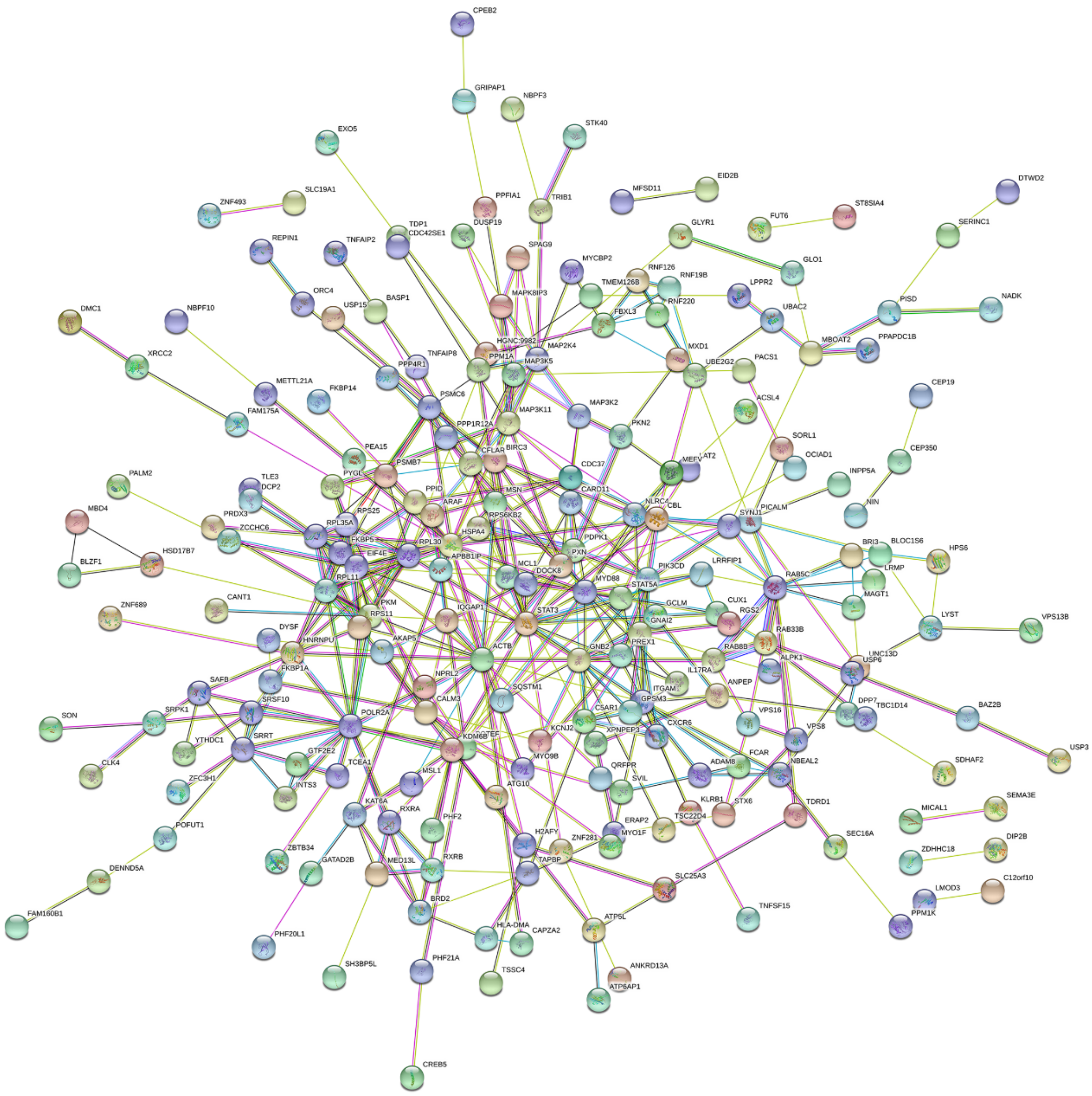


Supplementary Figure 4. PPI network of 296 overlapping feature mRNAs.

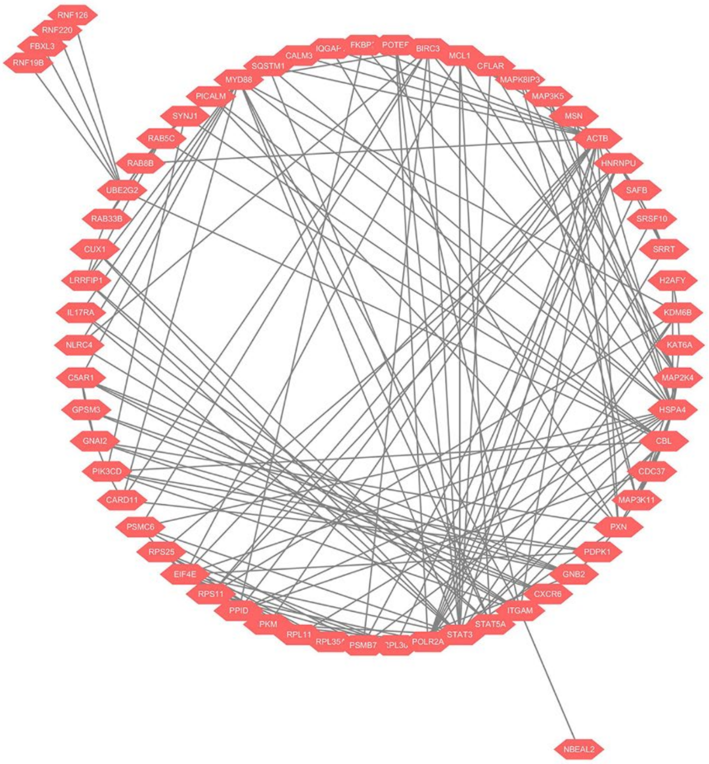


Supplementary Figure 5. PPI network of 63 feature mRNAs with a high probability of serving as hub mRNAs.

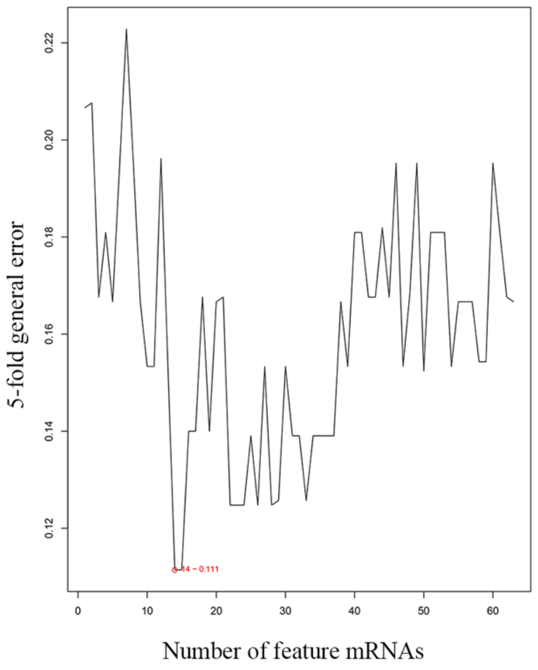


Supplementary Figure 6. The generalization error for different number of feature mRNAs.

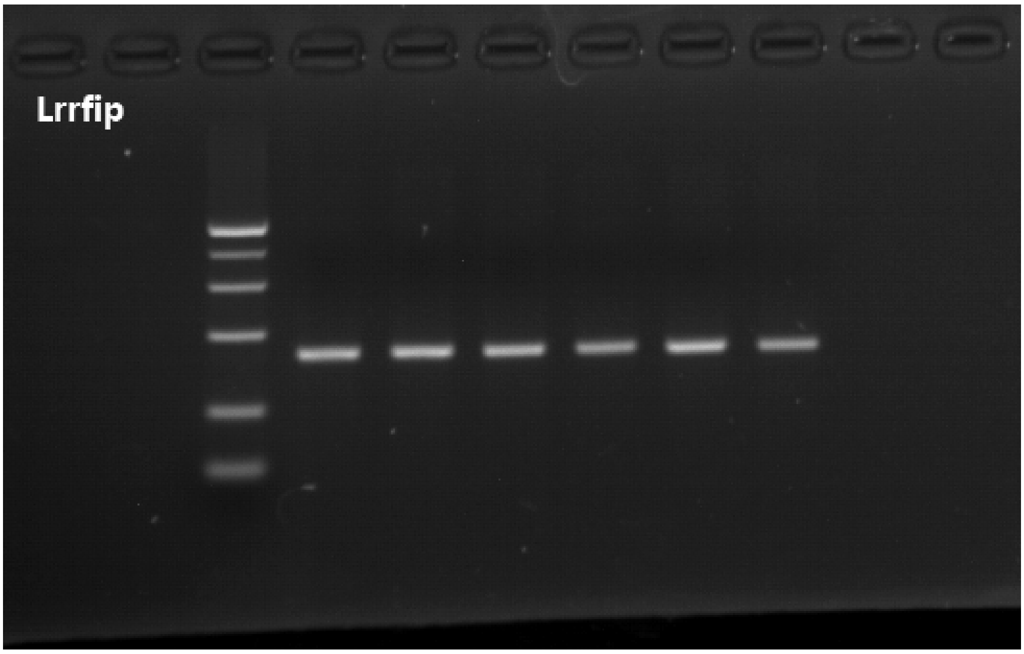
Lrrfip

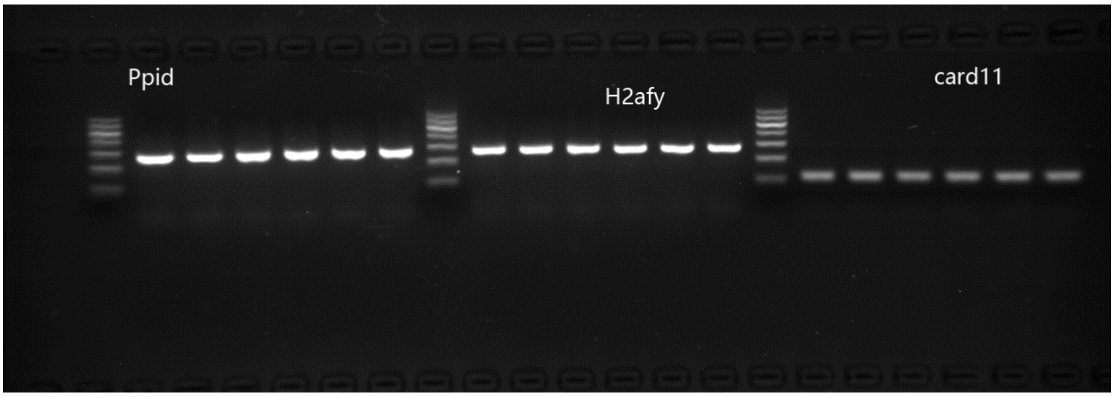
Ppid
H2afy
card11

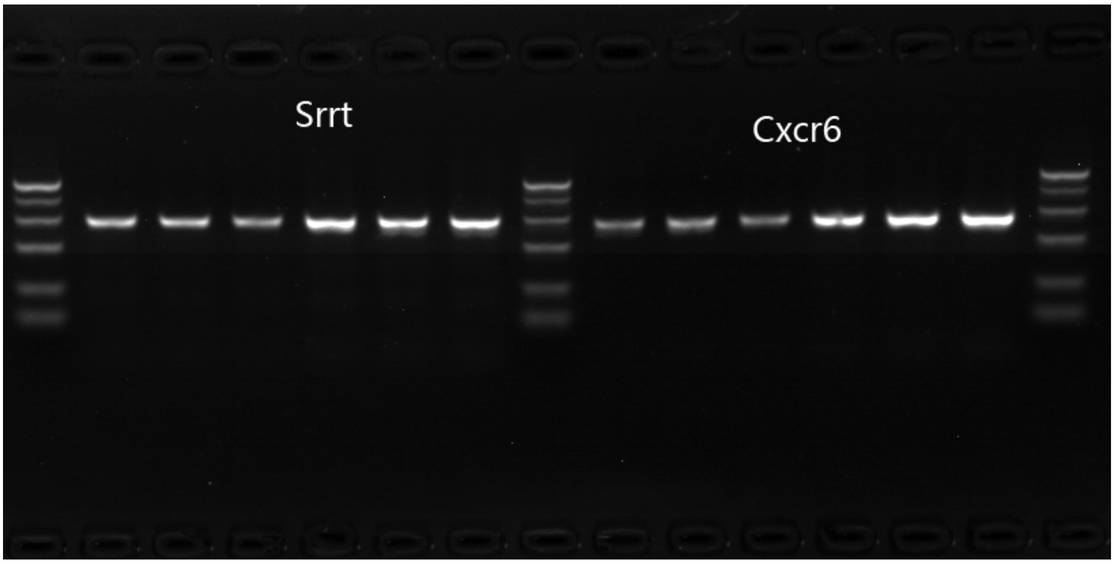
Srrt
Cxcr6

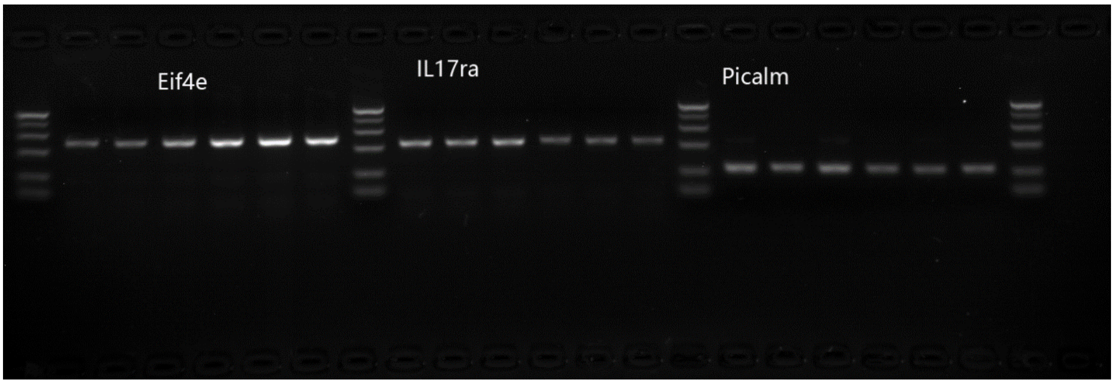
Eif4e
IL17ra
Picalm

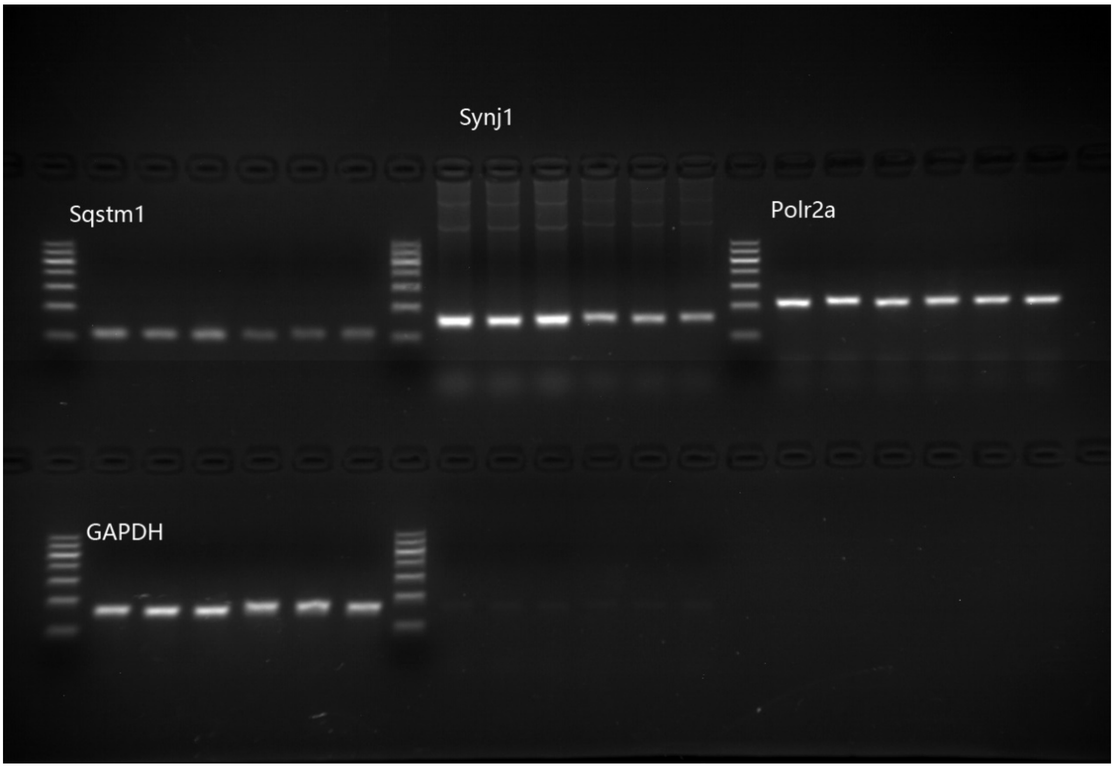
Synj1
Sqstm1
Polr2a
GAPDH

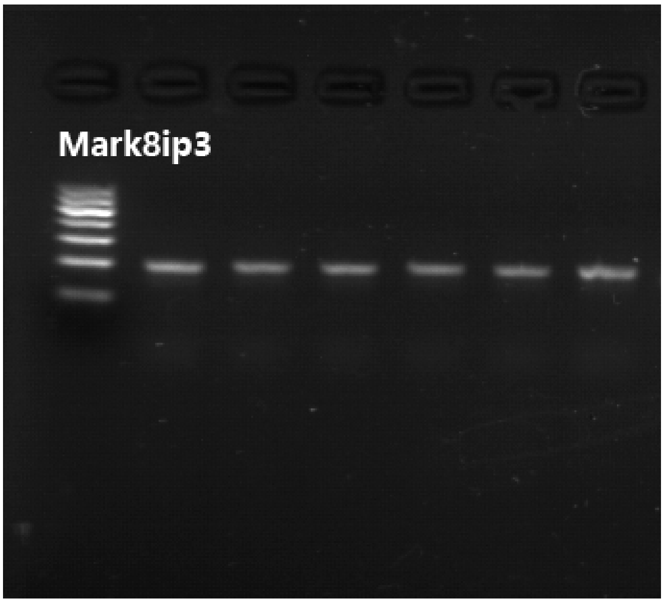
Mark8ip3

Supplementary Figure 7. Agarose gel electrophoresis.
