# Supplementary material for: Identification of diagnostic mRNA biomarkers in whole blood for ankylosing spondylitis using WGCNA and machine learning feature selection
Source: Front Immunol. 2022 Sep 12;13:956027. doi: 10.3389/fimmu.2022.956027 (PMC9510835; doi:10.3389/fimmu.2022.956027)
Supplement: Supplementary file 2 [file Table_1.docx]

Supplementary Table 1. List of Primers

| Gene | Sequences (5’-3’) |
| --- | --- |
| GAPDH | F: GGAAGCTTGTCATCAATGGAAATC |
|  | R: TGATGACCCTTTTGGCTCCC |
|  |  |
| Il17ra | F: CCAACATCACCGTGGAGACC |
|  | R: GTGGCGACAGCACCCTTTAA |
|  |  |
| Srrt | F: TGAGGAGTGGTTTCGGTCTAAGT |
|  | R: CAGCCCGTCCTTCTTCTTTCT |
|  |  |
| Cxcr6 | F: GCCAGGAGGAGCATCAAGAC |
|  | R: GCCCAGTAGGCTCTTGCACAT |
|  |  |
| Ppid | F: CATTACAAGCATGATCGGGAGG |
|  | R: TGACTGTCGCCAGAGCCATC |
|  |  |
| Card11 | F: ACAAACTGGTGACTGGGAAAGAG |
|  | R: TGCAGCTTGATGACCTCGTTC |
|  |  |
| Picalm | F: CTGCAAGCAGGGCAACTACAC |
|  | R: GTGCCTGTTCTTCCTCTAATGCT |
|  |  |
| Lrrfip1 | F: ATGGGGTGACATCGAGCAGT |
|  | R: AACCATTTGTCCAGGCGTAAG |
|  |  |
| Polr2a | F: AGGGTCATACTATTGGCATTGGG |
|  | R: CAGTCTTGTCTCGGGCATCG |
|  |  |
| Synj1 | F: TGGGATCTCATCGTGTCCGTAT |
|  | R: TTTCTGCCTTTCCTCCCTTAAC |
|  |  |
| Eif4e | F: GCAAACCTGCGGCTGATCT |
|  | R: ACAACAGCGCCACATACATCA |
|  |  |
| SQSTM1 | F: CCAGTCCCTACAGATGCCAGAA |
|  | R: GCCGCTCCGATGTCATAGTTC |
|  |  |
| H2afy | F: AGGAAACACGCTGGAGAAGAAA |
|  | R: TGCCGATGGATGGAAATGC |
|  |  |
| Mapk8ip3 | F: TGGAGCACTACGAGTTCCAGA |
|  | R: CTGGTGCAGGGCATTGTACT |
