# Supplementary material for: Identification of diagnostic mRNA biomarkers in whole blood for ankylosing spondylitis using WGCNA and machine learning feature selection
Source: Front Immunol. 2022 Sep 12;13:956027. doi: 10.3389/fimmu.2022.956027 (PMC9510835; doi:10.3389/fimmu.2022.956027)
Supplement: Supplementary file 3 [file Table_2.docx]

Supplementary Table 2. Weighted coexpression network

| Module | FromNode | ToNode | Weight |
| --- | --- | --- | --- |
| blue | ZSWIM6 | RNF24 | 0.020336075 |
| blue | ZSWIM6 | RNF149 | 0.025461893 |
| blue | ZSWIM6 | PTPRE | 0.024175553 |
| blue | ZSWIM6 | MXD1 | 0.029536714 |
| blue | ZSWIM6 | MSRB1 | 0.022315674 |
| blue | ZSWIM6 | MSL1 | 0.021242126 |
| blue | ZSWIM6 | GAB2 | 0.022800384 |
| blue | ZNF746 | UBN1 | 0.029854382 |
| blue | ZNF746 | SLC25A44 | 0.028774751 |
| blue | ZNF746 | REPS2 | 0.027599212 |
| blue | ZNF746 | PFKFB4 | 0.035321552 |
| blue | ZNF746 | PACSIN2 | 0.042568818 |
| blue | ZNF746 | OSBPL2 | 0.027176019 |
| blue | ZNF746 | NOTCH1 | 0.028194095 |
| blue | ZNF746 | NCF4 | 0.028874999 |
| blue | ZNF746 | ITPRIP | 0.029676029 |
| blue | ZNF746 | DHX34 | 0.020526108 |
| blue | ZNF746 | CSF3R | 0.035341628 |
| blue | ZNF746 | CEBPB | 0.021834306 |
| blue | ZNF746 | AQP9 | 0.025467974 |
| blue | ZNF746 | ABTB1 | 0.026975125 |
| blue | ZNF467 | TREM1 | 0.020428604 |
| blue | ZNF467 | SNX27 | 0.020524668 |
| blue | ZNF467 | PFKFB4 | 0.024562935 |
| blue | ZNF467 | PACSIN2 | 0.034377897 |
| blue | ZNF467 | IMPDH1 | 0.047830951 |
| blue | ZNF467 | CEBPB | 0.027151798 |
| blue | ZNF467 | AQP9 | 0.02137117 |
| blue | ZNF438 | BCL6 | 0.025266164 |
| blue | ZNF438 | AQP9 | 0.024131635 |
| blue | ZNF438 | ACSL1 | 0.024790704 |
| blue | ZNF428 | MXD1 | 0.020086584 |
| blue | ZNF281 | DYSF | 0.02006006 |
| blue | ZNF106 | ZDHHC18 | 0.029489092 |
| blue | ZNF106 | TKT | 0.024082578 |
| blue | ZNF106 | TBC1D14 | 0.021343944 |
| blue | ZNF106 | RXRA | 0.026174643 |
| blue | ZNF106 | RTN3 | 0.041757114 |
| blue | ZNF106 | PIK3CD | 0.021339137 |
| blue | ZNF106 | NADK | 0.020867335 |
| blue | ZNF106 | MXD1 | 0.025461837 |
| blue | ZNF106 | MSL1 | 0.021315398 |
| blue | ZNF106 | MME | 0.021959443 |
| blue | ZNF106 | ICAM3 | 0.021638153 |
| blue | ZNF106 | GAB2 | 0.022681065 |
| blue | ZNF106 | ARID3A | 0.030559492 |
| blue | ZFAND3 | ZDHHC18 | 0.026118745 |
| blue | ZFAND3 | TBC1D14 | 0.027934273 |
| blue | ZFAND3 | EXTL3 | 0.020047046 |
| blue | ZFAND3 | ARID3A | 0.021467131 |

| blue | ZDHHC18 | ZBTB34 | 0.022554833 |
| --- | --- | --- | --- |
| blue | ZDHHC18 | XKR8 | 0.048539322 |
| blue | ZDHHC18 | VPS8 | 0.022372433 |
| blue | ZDHHC18 | UNC13D | 0.02089479 |
| blue | ZDHHC18 | TUT7 | 0.023777291 |
| blue | ZDHHC18 | TRIB1 | 0.021108235 |
| blue | ZDHHC18 | TNFRSF1A | 0.038447048 |
| blue | ZDHHC18 | TNFAIP2 | 0.027479726 |
| blue | ZDHHC18 | TMEM126B | 0.034354237 |
| blue | ZDHHC18 | TLE4 | 0.020900638 |
| blue | ZDHHC18 | TLE3 | 0.048096239 |
| blue | ZDHHC18 | TIMP2 | 0.024999883 |
| blue | ZDHHC18 | TBC1D14 | 0.044942901 |
| blue | ZDHHC18 | SVIL | 0.032115026 |
| blue | ZDHHC18 | SUSD6 | 0.027629742 |
| blue | ZDHHC18 | STK40 | 0.032345933 |
| blue | ZDHHC18 | STAT3 | 0.026164726 |
| blue | ZDHHC18 | SPAG9 | 0.023384826 |
| blue | ZDHHC18 | SORL1 | 0.048480541 |
| blue | ZDHHC18 | SLCO3A1 | 0.029121913 |
| blue | ZDHHC18 | SLC19A1 | 0.047042727 |
| blue | ZDHHC18 | SLC16A3 | 0.021642083 |
| blue | ZDHHC18 | SLA | 0.020724554 |
| blue | ZDHHC18 | SKAP2 | 0.021712908 |
| blue | ZDHHC18 | SH3BP5L | 0.045854297 |
| blue | ZDHHC18 | SBNO2 | 0.031851008 |
| blue | ZDHHC18 | RXRA | 0.04462411 |
| blue | ZDHHC18 | RTN3 | 0.029903923 |
| blue | ZDHHC18 | RRAGD | 0.02050226 |
| blue | ZDHHC18 | RNF24 | 0.035402857 |
| blue | ZDHHC18 | RNF216P1 | 0.060934642 |
| blue | ZDHHC18 | RNF19B | 0.034817882 |
| blue | ZDHHC18 | RNF130 | 0.023911182 |
| blue | ZDHHC18 | RGS2 | 0.03311125 |
| blue | ZDHHC18 | RFX1 | 0.025068565 |
| blue | ZDHHC18 | RASSF2 | 0.038075855 |
| blue | ZDHHC18 | RAB5C | 0.03210072 |
| blue | ZDHHC18 | PYGL | 0.025778426 |
| blue | ZDHHC18 | PXN | 0.039824367 |
| blue | ZDHHC18 | PTPRE | 0.022370817 |
| blue | ZDHHC18 | PREX1 | 0.024430392 |
| blue | ZDHHC18 | PPP4R1 | 0.032983411 |
| blue | ZDHHC18 | PPP1R12A | 0.021338502 |
| blue | ZDHHC18 | POLR2H | 0.025242155 |
| blue | ZDHHC18 | PLPPR2 | 0.050513887 |
| blue | ZDHHC18 | PLEKHO2 | 0.048916682 |
| blue | ZDHHC18 | PKN2 | 0.041679526 |
| blue | ZDHHC18 | PISD | 0.026957847 |
| blue | ZDHHC18 | PIK3CD | 0.027667372 |
| blue | ZDHHC18 | PICALM | 0.036159154 |
| blue | ZDHHC18 | PELI2 | 0.033377387 |
| blue | ZDHHC18 | PECAM1 | 0.031732061 |

| blue | ZDHHC18 | PAK1 | 0.023671363 |
| --- | --- | --- | --- |
| blue | ZDHHC18 | NUP214 | 0.024952654 |
| blue | ZDHHC18 | NSMCE4A | 0.036309585 |
| blue | ZDHHC18 | NLRP12 | 0.026983422 |
| blue | ZDHHC18 | NCF1C | 0.02391242 |
| blue | ZDHHC18 | NADK | 0.03868811 |
| blue | ZDHHC18 | MYO9B | 0.023083622 |
| blue | ZDHHC18 | MYO1F | 0.040947551 |
| blue | ZDHHC18 | MXD1 | 0.034225126 |
| blue | ZDHHC18 | MTF1 | 0.022244532 |
| blue | ZDHHC18 | MSL1 | 0.042140296 |
| blue | ZDHHC18 | MME | 0.024570295 |
| blue | ZDHHC18 | MEFV | 0.043431552 |
| blue | ZDHHC18 | MED13L | 0.027027056 |
| blue | ZDHHC18 | MBOAT7 | 0.048060314 |
| blue | ZDHHC18 | MAST3 | 0.03576093 |
| blue | ZDHHC18 | MAP3K5 | 0.027870764 |
| blue | ZDHHC18 | MAP3K2 | 0.020758527 |
| blue | ZDHHC18 | MAP3K11 | 0.039292881 |
| blue | ZDHHC18 | MAP2K4 | 0.03258764 |
| blue | ZDHHC18 | LYST | 0.02063286 |
| blue | ZDHHC18 | LYN | 0.021910091 |
| blue | ZDHHC18 | LITAF | 0.022867066 |
| blue | ZDHHC18 | LAT2 | 0.032527796 |
| blue | ZDHHC18 | KIF1B | 0.024759909 |
| blue | ZDHHC18 | KIAA0513 | 0.033533051 |
| blue | ZDHHC18 | KDM6B | 0.028336976 |
| blue | ZDHHC18 | ITPK1 | 0.034332557 |
| blue | ZDHHC18 | IQSEC1 | 0.036858987 |
| blue | ZDHHC18 | IQGAP1 | 0.043871555 |
| blue | ZDHHC18 | IL17RA | 0.032768343 |
| blue | ZDHHC18 | ICAM3 | 0.037063289 |
| blue | ZDHHC18 | HSDL2 | 0.021360049 |
| blue | ZDHHC18 | HCK | 0.020286574 |
| blue | ZDHHC18 | GNB2 | 0.027929601 |
| blue | ZDHHC18 | GMIP | 0.023432631 |
| blue | ZDHHC18 | GLT1D1 | 0.024697964 |
| blue | ZDHHC18 | GAB2 | 0.027266825 |
| blue | ZDHHC18 | FNDC3B | 0.020788954 |
| blue | ZDHHC18 | FCGRT | 0.025046615 |
| blue | ZDHHC18 | EXTL3 | 0.030920165 |
| blue | ZDHHC18 | DYSF | 0.030543525 |
| blue | ZDHHC18 | DCP2 | 0.02378789 |
| blue | ZDHHC18 | CUX1 | 0.028380198 |
| blue | ZDHHC18 | CPSF4 | 0.020553132 |
| blue | ZDHHC18 | CPPED1 | 0.020256585 |
| blue | ZDHHC18 | CCNJL | 0.026261168 |
| blue | ZDHHC18 | CBL | 0.020447768 |
| blue | ZDHHC18 | CANT1 | 0.054078103 |
| blue | ZDHHC18 | C5AR1 | 0.047686755 |
| blue | ZDHHC18 | C3orf62 | 0.026357939 |
| blue | ZDHHC18 | C16orf58 | 0.035449419 |

| blue | ZDHHC18 | C15orf39 | 0.024594585 |
| --- | --- | --- | --- |
| blue | ZDHHC18 | BRI3 | 0.021612827 |
| blue | ZDHHC18 | BAZ2B | 0.02913402 |
| blue | ZDHHC18 | BASP1 | 0.026722672 |
| blue | ZDHHC18 | ARID3A | 0.033802439 |
| blue | ZDHHC18 | ARHGAP25 | 0.027919503 |
| blue | ZDHHC18 | ARAP1 | 0.03943452 |
| blue | ZDHHC18 | APBB1IP | 0.028671887 |
| blue | ZDHHC18 | ANTXR2 | 0.021304274 |
| blue | ZDHHC18 | ANPEP | 0.031698043 |
| blue | ZDHHC18 | AKIRIN2 | 0.021286572 |
| blue | ZDHHC18 | AGTPBP1 | 0.029253686 |
| blue | ZDHHC18 | ADAM8 | 0.044078116 |
| blue | ZBTB34 | XPO6 | 0.02174662 |
| blue | ZBTB34 | XKR8 | 0.021253015 |
| blue | ZBTB34 | VPS8 | 0.02630738 |
| blue | ZBTB34 | USP15 | 0.026914006 |
| blue | ZBTB34 | UBXN2B | 0.022573313 |
| blue | ZBTB34 | UBR2 | 0.028032808 |
| blue | ZBTB34 | TUT7 | 0.020304643 |
| blue | ZBTB34 | TNFRSF1A | 0.023991767 |
| blue | ZBTB34 | TNFAIP2 | 0.021804825 |
| blue | ZBTB34 | TMEM154 | 0.039162576 |
| blue | ZBTB34 | TMEM126B | 0.026015461 |
| blue | ZBTB34 | TLE3 | 0.020180411 |
| blue | ZBTB34 | TBC1D14 | 0.023680035 |
| blue | ZBTB34 | TALDO1 | 0.025011385 |
| blue | ZBTB34 | SUSD6 | 0.0244436 |
| blue | ZBTB34 | STX3 | 0.022616688 |
| blue | ZBTB34 | SPAG9 | 0.033629252 |
| blue | ZBTB34 | SLCO3A1 | 0.024278254 |
| blue | ZBTB34 | RNF24 | 0.021297085 |
| blue | ZBTB34 | RNF149 | 0.022533375 |
| blue | ZBTB34 | REPS2 | 0.020234147 |
| blue | ZBTB34 | RASSF2 | 0.021680877 |
| blue | ZBTB34 | RAF1 | 0.020054522 |
| blue | ZBTB34 | PTPRE | 0.022930704 |
| blue | ZBTB34 | PTAFR | 0.020782308 |
| blue | ZBTB34 | POLR2H | 0.021665546 |
| blue | ZBTB34 | PHF20L1 | 0.024150569 |
| blue | ZBTB34 | PELI2 | 0.022923326 |
| blue | ZBTB34 | PELI1 | 0.040022443 |
| blue | ZBTB34 | PAK1 | 0.020339082 |
| blue | ZBTB34 | OSBPL2 | 0.021204776 |
| blue | ZBTB34 | NT5C2 | 0.022979154 |
| blue | ZBTB34 | NSMCE4A | 0.020173308 |
| blue | ZBTB34 | NIBAN1 | 0.027120532 |
| blue | ZBTB34 | NDEL1 | 0.022700855 |
| blue | ZBTB34 | NCOA1 | 0.021970762 |
| blue | ZBTB34 | NCF2 | 0.020257314 |
| blue | ZBTB34 | NADK | 0.029118227 |
| blue | ZBTB34 | MXD1 | 0.038551381 |

| blue | ZBTB34 | MSL1 | 0.025229472 |
| --- | --- | --- | --- |
| blue | ZBTB34 | MCL1 | 0.027143707 |
| blue | ZBTB34 | MBOAT7 | 0.02343397 |
| blue | ZBTB34 | MAST3 | 0.022825977 |
| blue | ZBTB34 | MAP4K1 | 0.023156363 |
| blue | ZBTB34 | LRRK2 | 0.034960585 |
| blue | ZBTB34 | LRMP | 0.021080458 |
| blue | ZBTB34 | ITPRIP | 0.022782771 |
| blue | ZBTB34 | HLX | 0.025557813 |
| blue | ZBTB34 | GAB2 | 0.02924016 |
| blue | ZBTB34 | FNIP1 | 0.022821451 |
| blue | ZBTB34 | FNDC3B | 0.021480321 |
| blue | ZBTB34 | FCHO2 | 0.023187239 |
| blue | ZBTB34 | FCGR2A | 0.021591611 |
| blue | ZBTB34 | F11R | 0.023396039 |
| blue | ZBTB34 | DYSF | 0.020877173 |
| blue | ZBTB34 | DENND5A | 0.021267606 |
| blue | ZBTB34 | CXCR2 | 0.022613065 |
| blue | ZBTB34 | CXCR1 | 0.037501418 |
| blue | ZBTB34 | CTBS | 0.020957677 |
| blue | ZBTB34 | CSF3R | 0.028586933 |
| blue | ZBTB34 | CPSF4 | 0.020248896 |
| blue | ZBTB34 | CPD | 0.020795367 |
| blue | ZBTB34 | CFLAR | 0.024232143 |
| blue | ZBTB34 | C3orf62 | 0.021034187 |
| blue | ZBTB34 | BEST1 | 0.022433418 |
| blue | ZBTB34 | ATG16L2 | 0.021708919 |
| blue | ZBTB34 | ARAP1 | 0.024439908 |
| blue | ZBTB34 | ALPK1 | 0.02949398 |
| blue | ZBTB34 | AKR1B1 | 0.027367369 |
| blue | XPO6 | UBXN2B | 0.023558129 |
| blue | XPO6 | TSEN34 | 0.02058385 |
| blue | XPO6 | TLR8 | 0.020966699 |
| blue | XPO6 | THOC5 | 0.024943221 |
| blue | XPO6 | SULT1B1 | 0.020976817 |
| blue | XPO6 | STX3 | 0.040393627 |
| blue | XPO6 | ST6GALNAC2 | 0.028474953 |
| blue | XPO6 | ROPN1L | 0.020871102 |
| blue | XPO6 | RNF24 | 0.021580972 |
| blue | XPO6 | RNF149 | 0.031210478 |
| blue | XPO6 | REPS2 | 0.028023708 |
| blue | XPO6 | RBM47 | 0.020053809 |
| blue | XPO6 | RALB | 0.038045327 |
| blue | XPO6 | RAF1 | 0.022959099 |
| blue | XPO6 | PHF21A | 0.0217034 |
| blue | XPO6 | PFKFB4 | 0.021255959 |
| blue | XPO6 | OSBPL2 | 0.023183334 |
| blue | XPO6 | NUMB | 0.030328242 |
| blue | XPO6 | NIBAN1 | 0.023955322 |
| blue | XPO6 | NFIL3 | 0.03391447 |
| blue | XPO6 | NDEL1 | 0.031774598 |
| blue | XPO6 | MXD1 | 0.03980856 |

| blue | XPO6 | MSRB1 | 0.028429931 |
| --- | --- | --- | --- |
| blue | XPO6 | MSL1 | 0.020448847 |
| blue | XPO6 | MMP25 | 0.026852238 |
| blue | XPO6 | MGAM | 0.023361999 |
| blue | XPO6 | MAK | 0.022341777 |
| blue | XPO6 | KLHL2 | 0.024688543 |
| blue | XPO6 | ITPRIP | 0.025188841 |
| blue | XPO6 | GCA | 0.027321263 |
| blue | XPO6 | GAB2 | 0.044843561 |
| blue | XPO6 | FPR1 | 0.024146621 |
| blue | XPO6 | EXOC6 | 0.023690134 |
| blue | XPO6 | ETS2 | 0.032179149 |
| blue | XPO6 | CXCR1 | 0.029008568 |
| blue | XPO6 | CTBS | 0.021271734 |
| blue | XPO6 | CSF3R | 0.037656813 |
| blue | XPO6 | CHST15 | 0.024176399 |
| blue | XPO6 | CFLAR | 0.024047454 |
| blue | XPO6 | CEBPB | 0.031700189 |
| blue | XPO6 | BCL6 | 0.04520995 |
| blue | XPO6 | AQP9 | 0.049177164 |
| blue | XPO6 | ACSL1 | 0.032558381 |
| blue | XKR8 | TMEM126B | 0.025848011 |
| blue | XKR8 | RNF216P1 | 0.048075988 |
| blue | XKR8 | PKN2 | 0.021424903 |
| blue | XKR8 | NADK | 0.024904488 |
| blue | XKR8 | MXD1 | 0.026053913 |
| blue | XKR8 | MSL1 | 0.020287384 |
| blue | XKR8 | ADAM8 | 0.038983962 |
| blue | WAS | TNFRSF1A | 0.023535937 |
| blue | WAS | RNF24 | 0.026038201 |
| blue | WAS | RNF149 | 0.020557548 |
| blue | WAS | RGS2 | 0.026811161 |
| blue | WAS | MXD1 | 0.022289861 |
| blue | WAS | MSRB1 | 0.021548104 |
| blue | WAS | MSL1 | 0.028154745 |
| blue | WAS | LYN | 0.028682726 |
| blue | WAS | BASP1 | 0.021150021 |
| blue | WAS | ATP6V1B2 | 0.020622762 |
| blue | VSIR | VMP1 | 0.020974847 |
| blue | VSIR | TLR8 | 0.020930514 |
| blue | VSIR | STX3 | 0.029786202 |
| blue | VSIR | SIRPA | 0.032096793 |
| blue | VSIR | REPS2 | 0.042607916 |
| blue | VSIR | RALB | 0.028694056 |
| blue | VSIR | PFKFB4 | 0.034827123 |
| blue | VSIR | PACSIN2 | 0.022532353 |
| blue | VSIR | OSBPL2 | 0.021485536 |
| blue | VSIR | NUMB | 0.022838305 |
| blue | VSIR | NFIL3 | 0.020491255 |
| blue | VSIR | NDEL1 | 0.02050357 |
| blue | VSIR | NCF4 | 0.022747264 |
| blue | VSIR | MSRB1 | 0.02247593 |

| blue | VSIR | LRG1 | 0.021682138 |
| --- | --- | --- | --- |
| blue | VSIR | ITPRIP | 0.028438474 |
| blue | VSIR | IL1R2 | 0.022306003 |
| blue | VSIR | IFNGR1 | 0.020969682 |
| blue | VSIR | GAB2 | 0.024222814 |
| blue | VSIR | CSF3R | 0.03348028 |
| blue | VSIR | CEBPB | 0.041647369 |
| blue | VSIR | BCL6 | 0.021729141 |
| blue | VSIR | AQP9 | 0.035449113 |
| blue | VPS8 | NADK | 0.023467035 |
| blue | VPS8 | MXD1 | 0.037776265 |
| blue | VPS8 | MSL1 | 0.020868981 |
| blue | VPS8 | GAB2 | 0.036161347 |
| blue | VPS8 | DYSF | 0.021994248 |
| blue | VNN2 | TSEN34 | 0.020044026 |
| blue | VNN2 | STX3 | 0.0223608 |
| blue | VNN2 | S100A11 | 0.023486441 |
| blue | VNN2 | RNF149 | 0.032520937 |
| blue | VNN2 | RGS2 | 0.022521524 |
| blue | VNN2 | RALB | 0.044709556 |
| blue | VNN2 | RAF1 | 0.020794831 |
| blue | VNN2 | QPCT | 0.020586102 |
| blue | VNN2 | PYGL | 0.020762429 |
| blue | VNN2 | NUMB | 0.032501769 |
| blue | VNN2 | NFIL3 | 0.040291048 |
| blue | VNN2 | NDEL1 | 0.034493934 |
| blue | VNN2 | NAMPT | 0.026085562 |
| blue | VNN2 | MXD1 | 0.028751229 |
| blue | VNN2 | MSRB1 | 0.044990938 |
| blue | VNN2 | LYN | 0.028315443 |
| blue | VNN2 | LITAF | 0.021097141 |
| blue | VNN2 | ITPRIP | 0.020232634 |
| blue | VNN2 | GCA | 0.041390667 |
| blue | VNN2 | GAB2 | 0.035998595 |
| blue | VNN2 | DYSF | 0.020482816 |
| blue | VNN2 | CSF3R | 0.02468636 |
| blue | VNN2 | CEBPB | 0.029151348 |
| blue | VNN2 | BCL6 | 0.03369616 |
| blue | VNN2 | BASP1 | 0.027621025 |
| blue | VNN2 | ATP6V1B2 | 0.022243757 |
| blue | VNN2 | AQP9 | 0.048930506 |
| blue | VNN2 | ACSL1 | 0.034813466 |
| blue | VMP1 | REPS2 | 0.038301233 |
| blue | VMP1 | RALB | 0.02596636 |
| blue | VMP1 | PACSIN2 | 0.02466551 |
| blue | VMP1 | OSBPL2 | 0.020126227 |
| blue | VMP1 | NUMB | 0.02524203 |
| blue | VMP1 | IGF2R | 0.021395256 |
| blue | VMP1 | CSF3R | 0.029395243 |
| blue | VMP1 | CEBPB | 0.032856544 |
| blue | VMP1 | AQP9 | 0.029806742 |
| blue | VAMP3 | RNF24 | 0.021885413 |

| blue | VAMP3 | RGS2 | 0.027299813 |
| --- | --- | --- | --- |
| blue | VAMP3 | LYN | 0.022320364 |
| blue | USP6 | MXD1 | 0.020720911 |
| blue | USP6 | CUX1 | 0.021712303 |
| blue | USP15 | PHF20L1 | 0.025731079 |
| blue | USP15 | PELI1 | 0.024668895 |
| blue | USP15 | NADK | 0.02211942 |
| blue | USP15 | MXD1 | 0.050577291 |
| blue | USP15 | GAB2 | 0.0298426 |
| blue | USB1 | ROPN1L | 0.026938582 |
| blue | USB1 | RALB | 0.02466214 |
| blue | USB1 | NFIL3 | 0.026907751 |
| blue | USB1 | NCF4 | 0.027186223 |
| blue | USB1 | NAMPT | 0.025506904 |
| blue | USB1 | LRG1 | 0.028271767 |
| blue | USB1 | ITPRIP | 0.021668426 |
| blue | USB1 | FCGR2A | 0.020126625 |
| blue | USB1 | CSF3R | 0.024887036 |
| blue | USB1 | CEBPB | 0.025285454 |
| blue | USB1 | BCL6 | 0.037857739 |
| blue | USB1 | AQP9 | 0.048097996 |
| blue | USB1 | ACSL1 | 0.034558878 |
| blue | UNC13D | CUX1 | 0.025245045 |
| blue | UBXN2B | TMCC3 | 0.02192075 |
| blue | UBXN2B | STX3 | 0.027353573 |
| blue | UBXN2B | SRPK1 | 0.033023792 |
| blue | UBXN2B | SLC2A14 | 0.023294051 |
| blue | UBXN2B | RTN3 | 0.020694828 |
| blue | UBXN2B | RNF24 | 0.02703301 |
| blue | UBXN2B | RNF149 | 0.029753882 |
| blue | UBXN2B | RGS2 | 0.020069019 |
| blue | UBXN2B | RALB | 0.033627026 |
| blue | UBXN2B | RAF1 | 0.023424207 |
| blue | UBXN2B | PYGL | 0.030035641 |
| blue | UBXN2B | PLXNC1 | 0.023051868 |
| blue | UBXN2B | PHF21A | 0.025356303 |
| blue | UBXN2B | PELI2 | 0.020943075 |
| blue | UBXN2B | NUMB | 0.022456496 |
| blue | UBXN2B | NIBAN1 | 0.020828376 |
| blue | UBXN2B | NFIL3 | 0.030210796 |
| blue | UBXN2B | NDEL1 | 0.029661408 |
| blue | UBXN2B | NADK | 0.020625792 |
| blue | UBXN2B | MXD1 | 0.048380323 |
| blue | UBXN2B | MSRB1 | 0.025635569 |
| blue | UBXN2B | MSL1 | 0.026391335 |
| blue | UBXN2B | MME | 0.029403192 |
| blue | UBXN2B | MGAM2 | 0.0296742 |
| blue | UBXN2B | MGAM | 0.04022755 |
| blue | UBXN2B | LRRK2 | 0.02514449 |
| blue | UBXN2B | KLHL2 | 0.021463318 |
| blue | UBXN2B | GCA | 0.029931162 |
| blue | UBXN2B | GAB2 | 0.040263903 |

| blue | UBXN2B | FAM160B1 | 0.022301863 |
| --- | --- | --- | --- |
| blue | UBXN2B | F5 | 0.020065522 |
| blue | UBXN2B | EXOC6 | 0.026474189 |
| blue | UBXN2B | ETS2 | 0.021680718 |
| blue | UBXN2B | DYSF | 0.032071871 |
| blue | UBXN2B | DENND5A | 0.020897908 |
| blue | UBXN2B | CSF3R | 0.024331596 |
| blue | UBXN2B | CHST15 | 0.022674994 |
| blue | UBXN2B | BCL6 | 0.043478399 |
| blue | UBXN2B | BASP1 | 0.025342226 |
| blue | UBXN2B | AQP9 | 0.038596462 |
| blue | UBXN2B | AGO4 | 0.021453812 |
| blue | UBXN2B | ADGRE3 | 0.021759762 |
| blue | UBXN2B | ACSL1 | 0.032997525 |
| blue | UBR2 | UBN1 | 0.022745332 |
| blue | UBR2 | STX3 | 0.021777982 |
| blue | UBR2 | SPAG9 | 0.023277455 |
| blue | UBR2 | RNF149 | 0.021952156 |
| blue | UBR2 | RALB | 0.025894207 |
| blue | UBR2 | RAF1 | 0.022556775 |
| blue | UBR2 | PELI1 | 0.023068455 |
| blue | UBR2 | OSBPL2 | 0.025052624 |
| blue | UBR2 | NUMB | 0.020848043 |
| blue | UBR2 | NDEL1 | 0.033970199 |
| blue | UBR2 | MXD1 | 0.040971926 |
| blue | UBR2 | ITPRIP | 0.037884552 |
| blue | UBR2 | GAB2 | 0.036331902 |
| blue | UBR2 | CXCR1 | 0.026106423 |
| blue | UBR2 | CSF3R | 0.047997656 |
| blue | UBR2 | AQP9 | 0.03299859 |
| blue | UBN1 | STX3 | 0.028564037 |
| blue | UBN1 | SLC44A2 | 0.02096198 |
| blue | UBN1 | SLC25A44 | 0.035498775 |
| blue | UBN1 | RNF149 | 0.020236094 |
| blue | UBN1 | REPS2 | 0.023921886 |
| blue | UBN1 | RALB | 0.028072594 |
| blue | UBN1 | PFKFB4 | 0.028681275 |
| blue | UBN1 | PACSIN2 | 0.030084973 |
| blue | UBN1 | OSBPL2 | 0.02936749 |
| blue | UBN1 | NUMB | 0.025182723 |
| blue | UBN1 | NOTCH1 | 0.026615833 |
| blue | UBN1 | NDEL1 | 0.030112445 |
| blue | UBN1 | NCOA1 | 0.023513113 |
| blue | UBN1 | NCF4 | 0.022848016 |
| blue | UBN1 | MXD1 | 0.027450241 |
| blue | UBN1 | MSRB1 | 0.026436947 |
| blue | UBN1 | ITPRIP | 0.046059337 |
| blue | UBN1 | IGF2R | 0.026042977 |
| blue | UBN1 | GAB2 | 0.030954616 |
| blue | UBN1 | CXCR1 | 0.023717695 |
| blue | UBN1 | CSF3R | 0.04980081 |
| blue | UBN1 | CHST15 | 0.030320153 |

| blue | UBN1 | CEBPB | 0.025860634 |
| --- | --- | --- | --- |
| blue | UBN1 | BCL6 | 0.023566351 |
| blue | UBN1 | AQP9 | 0.041098196 |
| blue | UBN1 | ABTB1 | 0.02234838 |
| blue | TYROBP | TALDO1 | 0.034104155 |
| blue | TYROBP | S100A11 | 0.02337616 |
| blue | TYROBP | RNF149 | 0.023460492 |
| blue | TYROBP | RNF130 | 0.027582286 |
| blue | TYROBP | RHOG | 0.02365292 |
| blue | TYROBP | RGS2 | 0.022507143 |
| blue | TYROBP | RALB | 0.026400367 |
| blue | TYROBP | NUMB | 0.021903332 |
| blue | TYROBP | NDEL1 | 0.020805244 |
| blue | TYROBP | NCF2 | 0.02063556 |
| blue | TYROBP | MSRB1 | 0.058927138 |
| blue | TYROBP | LYN | 0.033365378 |
| blue | TYROBP | IFNGR2 | 0.023470473 |
| blue | TYROBP | HLX | 0.020360472 |
| blue | TYROBP | GCA | 0.03292914 |
| blue | TYROBP | GAB2 | 0.024734281 |
| blue | TYROBP | ATP6V1B2 | 0.046260819 |
| blue | TYROBP | AQP9 | 0.025503579 |
| blue | TUT7 | TNFRSF1A | 0.021403012 |
| blue | TUT7 | STAT3 | 0.030405404 |
| blue | TUT7 | SORL1 | 0.022898185 |
| blue | TUT7 | NADK | 0.020455067 |
| blue | TUT7 | MXD1 | 0.03343269 |
| blue | TUT7 | MSL1 | 0.023737896 |
| blue | TUT7 | GAB2 | 0.025744226 |
| blue | TUT7 | CFLAR | 0.021076556 |
| blue | TSEN34 | TM6SF1 | 0.020224932 |
| blue | TSEN34 | TLR8 | 0.02335373 |
| blue | TSEN34 | THOC5 | 0.024498939 |
| blue | TSEN34 | TALDO1 | 0.021237534 |
| blue | TSEN34 | STX3 | 0.039608379 |
| blue | TSEN34 | ST6GALNAC2 | 0.022157027 |
| blue | TSEN34 | SRGN | 0.037752671 |
| blue | TSEN34 | SLC2A3 | 0.021767648 |
| blue | TSEN34 | S100A11 | 0.021163769 |
| blue | TSEN34 | ROPN1L | 0.030421239 |
| blue | TSEN34 | RNF149 | 0.039067465 |
| blue | TSEN34 | RBM47 | 0.030050286 |
| blue | TSEN34 | RALB | 0.059868293 |
| blue | TSEN34 | QPCT | 0.025946499 |
| blue | TSEN34 | PFKFB4 | 0.026692867 |
| blue | TSEN34 | NUMB | 0.04045617 |
| blue | TSEN34 | NRBF2 | 0.029577626 |
| blue | TSEN34 | NFIL3 | 0.043860034 |
| blue | TSEN34 | NDEL1 | 0.036693602 |
| blue | TSEN34 | NCF4 | 0.028113796 |
| blue | TSEN34 | NCF2 | 0.023135572 |
| blue | TSEN34 | NAMPT | 0.036595002 |

| blue | TSEN34 | MXD1 | 0.029804137 |
| --- | --- | --- | --- |
| blue | TSEN34 | MSRB1 | 0.035575284 |
| blue | TSEN34 | MNDA | 0.024103228 |
| blue | TSEN34 | MMP25 | 0.022585347 |
| blue | TSEN34 | MANSC1 | 0.021955005 |
| blue | TSEN34 | LRG1 | 0.032627059 |
| blue | TSEN34 | LMNB1 | 0.020577827 |
| blue | TSEN34 | KLHL2 | 0.026575627 |
| blue | TSEN34 | ITPRIP | 0.032570248 |
| blue | TSEN34 | IFNGR1 | 0.026967909 |
| blue | TSEN34 | IFITM2 | 0.024138099 |
| blue | TSEN34 | HIST1H2BC | 0.02169189 |
| blue | TSEN34 | GCA | 0.045910862 |
| blue | TSEN34 | GAB2 | 0.036785063 |
| blue | TSEN34 | FPR1 | 0.022979099 |
| blue | TSEN34 | FLOT2 | 0.033807227 |
| blue | TSEN34 | FCGR2A | 0.02053725 |
| blue | TSEN34 | EXOC6 | 0.020824691 |
| blue | TSEN34 | EVI2B | 0.022106088 |
| blue | TSEN34 | CXCR1 | 0.02301144 |
| blue | TSEN34 | CSF3R | 0.04444356 |
| blue | TSEN34 | CEBPB | 0.045925375 |
| blue | TSEN34 | CEACAM3 | 0.021706139 |
| blue | TSEN34 | CA4 | 0.020431843 |
| blue | TSEN34 | BCL6 | 0.045919436 |
| blue | TSEN34 | BCL2A1 | 0.029897382 |
| blue | TSEN34 | AQP9 | 0.076352783 |
| blue | TSEN34 | ALOX5 | 0.020917225 |
| blue | TSEN34 | ACSL1 | 0.043753222 |
| blue | TRIB1 | RNF24 | 0.026468766 |
| blue | TRIB1 | RGS2 | 0.026326522 |
| blue | TRIB1 | PYGL | 0.023275059 |
| blue | TRIB1 | NDEL1 | 0.021759038 |
| blue | TRIB1 | NADK | 0.020440962 |
| blue | TRIB1 | MXD1 | 0.035831421 |
| blue | TRIB1 | MSL1 | 0.021664271 |
| blue | TRIB1 | GCA | 0.020091952 |
| blue | TRIB1 | GAB2 | 0.032156199 |
| blue | TRIB1 | DYSF | 0.025071309 |
| blue | TRIB1 | BASP1 | 0.022055194 |
| blue | TRIB1 | ARID3A | 0.02274331 |
| blue | TREM1 | MAPK1 | 0.024255957 |
| blue | TREM1 | HIST2H2AC | 0.02316242 |
| blue | TREM1 | CEBPB | 0.02184073 |
| blue | TREM1 | AQP9 | 0.022449513 |
| blue | TOM1 | PACSIN2 | 0.023258412 |
| blue | TOM1 | IMPDH1 | 0.029781266 |
| blue | TNFRSF1A | TNFAIP2 | 0.021202203 |
| blue | TNFRSF1A | TLR1 | 0.023901199 |
| blue | TNFRSF1A | TLE3 | 0.033761562 |
| blue | TNFRSF1A | TKT | 0.029693884 |
| blue | TNFRSF1A | TIMP2 | 0.026625616 |

| blue | TNFRSF1A | TBC1D14 | 0.026870477 |
| --- | --- | --- | --- |
| blue | TNFRSF1A | TALDO1 | 0.026338997 |
| blue | TNFRSF1A | STX3 | 0.021590612 |
| blue | TNFRSF1A | STK3 | 0.041817684 |
| blue | TNFRSF1A | SLCO3A1 | 0.022505295 |
| blue | TNFRSF1A | SLC19A1 | 0.028117663 |
| blue | TNFRSF1A | SLC16A3 | 0.022785662 |
| blue | TNFRSF1A | SERPINA1 | 0.023945877 |
| blue | TNFRSF1A | SBNO2 | 0.022327629 |
| blue | TNFRSF1A | RXRA | 0.025240009 |
| blue | TNFRSF1A | RTN3 | 0.023337592 |
| blue | TNFRSF1A | RNF24 | 0.039014524 |
| blue | TNFRSF1A | RNF149 | 0.032328538 |
| blue | TNFRSF1A | RNF130 | 0.023944084 |
| blue | TNFRSF1A | RHOG | 0.028832648 |
| blue | TNFRSF1A | RGS2 | 0.025575279 |
| blue | TNFRSF1A | RASSF2 | 0.022756688 |
| blue | TNFRSF1A | RALB | 0.021098877 |
| blue | TNFRSF1A | PYGL | 0.025303352 |
| blue | TNFRSF1A | PTPRE | 0.021476249 |
| blue | TNFRSF1A | PTEN | 0.020225082 |
| blue | TNFRSF1A | PLPPR2 | 0.030814271 |
| blue | TNFRSF1A | PLEKHO2 | 0.032274082 |
| blue | TNFRSF1A | PILRA | 0.023514109 |
| blue | TNFRSF1A | PELI2 | 0.023436866 |
| blue | TNFRSF1A | PECAM1 | 0.021111667 |
| blue | TNFRSF1A | PAK1 | 0.020165842 |
| blue | TNFRSF1A | NUP214 | 0.030027533 |
| blue | TNFRSF1A | NLRP12 | 0.021097166 |
| blue | TNFRSF1A | NIBAN1 | 0.02770095 |
| blue | TNFRSF1A | NDEL1 | 0.024594922 |
| blue | TNFRSF1A | NCF1C | 0.022680809 |
| blue | TNFRSF1A | NADK | 0.033494623 |
| blue | TNFRSF1A | MYO1F | 0.025556326 |
| blue | TNFRSF1A | MYD88 | 0.020593522 |
| blue | TNFRSF1A | MXD1 | 0.039464658 |
| blue | TNFRSF1A | MSRB1 | 0.034727203 |
| blue | TNFRSF1A | MSL1 | 0.04035999 |
| blue | TNFRSF1A | MBOAT7 | 0.044952434 |
| blue | TNFRSF1A | MAP3K11 | 0.031710958 |
| blue | TNFRSF1A | LYN | 0.03349925 |
| blue | TNFRSF1A | LTBR | 0.035829805 |
| blue | TNFRSF1A | LITAF | 0.025186716 |
| blue | TNFRSF1A | LCP1 | 0.031076684 |
| blue | TNFRSF1A | LAT2 | 0.023241829 |
| blue | TNFRSF1A | KIAA0513 | 0.022115296 |
| blue | TNFRSF1A | IQSEC1 | 0.025253641 |
| blue | TNFRSF1A | IL17RA | 0.028098011 |
| blue | TNFRSF1A | ICAM3 | 0.021047477 |
| blue | TNFRSF1A | HLX | 0.025300963 |
| blue | TNFRSF1A | HCK | 0.035115247 |
| blue | TNFRSF1A | GCA | 0.023490758 |

| blue | TNFRSF1A | GAB2 | 0.039535712 |
| --- | --- | --- | --- |
| blue | TNFRSF1A | FPR1 | 0.0285414 |
| blue | TNFRSF1A | FCGRT | 0.021325 |
| blue | TNFRSF1A | ETS2 | 0.02086131 |
| blue | TNFRSF1A | DYSF | 0.025737652 |
| blue | TNFRSF1A | DIP2B | 0.024718003 |
| blue | TNFRSF1A | CXCR1 | 0.02852033 |
| blue | TNFRSF1A | CSF3R | 0.02213532 |
| blue | TNFRSF1A | CPD | 0.021003695 |
| blue | TNFRSF1A | CANT1 | 0.022500312 |
| blue | TNFRSF1A | C5AR1 | 0.026175812 |
| blue | TNFRSF1A | C15orf39 | 0.029187693 |
| blue | TNFRSF1A | BCL3 | 0.025701162 |
| blue | TNFRSF1A | BASP1 | 0.028492578 |
| blue | TNFRSF1A | ATP6V1B2 | 0.028906395 |
| blue | TNFRSF1A | ARID3A | 0.023481011 |
| blue | TNFRSF1A | ARHGAP25 | 0.022371382 |
| blue | TNFRSF1A | ARAP1 | 0.034598809 |
| blue | TNFRSF1A | AQP9 | 0.021043236 |
| blue | TNFAIP6 | RNF149 | 0.021138353 |
| blue | TNFAIP6 | AQP9 | 0.02157209 |
| blue | TNFAIP2 | NADK | 0.020512944 |
| blue | TNFAIP2 | MXD1 | 0.020444417 |
| blue | TNFAIP2 | MSL1 | 0.023141178 |
| blue | TNFAIP2 | ARAP1 | 0.020385888 |
| blue | TMEM154 | RAB11FIP1 | 0.022281265 |
| blue | TMEM154 | PELI1 | 0.029098902 |
| blue | TMEM154 | NDEL1 | 0.027281098 |
| blue | TMEM154 | NADK | 0.020374141 |
| blue | TMEM154 | MXD1 | 0.03911316 |
| blue | TMEM154 | ITPRIP | 0.020771036 |
| blue | TMEM154 | GAB2 | 0.031091036 |
| blue | TMEM154 | F11R | 0.029442749 |
| blue | TMEM154 | CXCR1 | 0.02681268 |
| blue | TMEM154 | CSF3R | 0.030007201 |
| blue | TMEM126B | SORL1 | 0.022590133 |
| blue | TMEM126B | SLC19A1 | 0.020729396 |
| blue | TMEM126B | RNF216P1 | 0.023295742 |
| blue | TMEM126B | PKN2 | 0.032291381 |
| blue | TMEM126B | PELI1 | 0.021644487 |
| blue | TMEM126B | NSMCE4A | 0.025399046 |
| blue | TMEM126B | NADK | 0.029701039 |
| blue | TMEM126B | MXD1 | 0.024003182 |
| blue | TMEM126B | MSL1 | 0.021833404 |
| blue | TMEM126B | MEFV | 0.027544974 |
| blue | TMEM126B | MAST3 | 0.020029489 |
| blue | TMEM120A | STX3 | 0.022987514 |
| blue | TMEM120A | CSF3R | 0.020729587 |
| blue | TMEM120A | CEBPB | 0.027802632 |
| blue | TMEM120A | AQP9 | 0.023680717 |
| blue | TMCC3 | SLA | 0.024190676 |
| blue | TMCC3 | RGS2 | 0.020143303 |

| blue | TMCC3 | RAF1 | 0.021122978 |
| --- | --- | --- | --- |
| blue | TMCC3 | PHC2 | 0.020239105 |
| blue | TMCC3 | NDEL1 | 0.020993647 |
| blue | TMCC3 | MXD1 | 0.033906514 |
| blue | TMCC3 | MME | 0.027523482 |
| blue | TMCC3 | MGAM | 0.026879737 |
| blue | TMCC3 | MEGF9 | 0.02113296 |
| blue | TMCC3 | GAB2 | 0.026448724 |
| blue | TMCC3 | CHST15 | 0.024448684 |
| blue | TM6SF1 | SRGN | 0.020635268 |
| blue | TM6SF1 | RALB | 0.030924031 |
| blue | TM6SF1 | QPCT | 0.0239271 |
| blue | TM6SF1 | MIR223 | 0.020157797 |
| blue | TM6SF1 | GCA | 0.023773176 |
| blue | TM6SF1 | CEBPB | 0.022754979 |
| blue | TM6SF1 | AQP9 | 0.024479713 |
| blue | TLR8 | TALDO1 | 0.027567889 |
| blue | TLR8 | STX3 | 0.034227648 |
| blue | TLR8 | ST6GALNAC2 | 0.021735804 |
| blue | TLR8 | SIRPA | 0.023090676 |
| blue | TLR8 | RTN3 | 0.021792598 |
| blue | TLR8 | RNF24 | 0.020087647 |
| blue | TLR8 | RNF149 | 0.033962713 |
| blue | TLR8 | RBM47 | 0.030712237 |
| blue | TLR8 | RALB | 0.054444636 |
| blue | TLR8 | PYGL | 0.024363499 |
| blue | TLR8 | PFKFB4 | 0.020747093 |
| blue | TLR8 | NUMB | 0.037564952 |
| blue | TLR8 | NRBF2 | 0.025424407 |
| blue | TLR8 | NFIL3 | 0.03364278 |
| blue | TLR8 | NDEL1 | 0.033606727 |
| blue | TLR8 | NCF2 | 0.021897108 |
| blue | TLR8 | NAMPT | 0.023144288 |
| blue | TLR8 | MXD1 | 0.036183155 |
| blue | TLR8 | MSRB1 | 0.04843293 |
| blue | TLR8 | MME | 0.022481768 |
| blue | TLR8 | MGAM | 0.025128386 |
| blue | TLR8 | LYN | 0.020631976 |
| blue | TLR8 | LY96 | 0.023550764 |
| blue | TLR8 | ITPRIP | 0.02883775 |
| blue | TLR8 | IGF2R | 0.02302478 |
| blue | TLR8 | IFNGR1 | 0.024573554 |
| blue | TLR8 | HSPA1A | 0.024140926 |
| blue | TLR8 | GCA | 0.034798338 |
| blue | TLR8 | GAB2 | 0.047323115 |
| blue | TLR8 | FPR1 | 0.022771706 |
| blue | TLR8 | EXOC6 | 0.02015717 |
| blue | TLR8 | DYSF | 0.020968237 |
| blue | TLR8 | CXCR1 | 0.023800689 |
| blue | TLR8 | CSF3R | 0.036665111 |
| blue | TLR8 | COP1 | 0.024467189 |
| blue | TLR8 | CMTM2 | 0.023763071 |

| blue | TLR8 | CHST15 | 0.023944091 |
| --- | --- | --- | --- |
| blue | TLR8 | CEBPB | 0.037834348 |
| blue | TLR8 | BCL6 | 0.038635043 |
| blue | TLR8 | BASP1 | 0.022586468 |
| blue | TLR8 | ATP6V1B2 | 0.02624718 |
| blue | TLR8 | AQP9 | 0.056663094 |
| blue | TLR8 | ACSL1 | 0.029169911 |
| blue | TLR5 | PHF21A | 0.020881891 |
| blue | TLR5 | PFKFB3 | 0.030009705 |
| blue | TLR5 | NFIL3 | 0.020962508 |
| blue | TLR5 | MCEMP1 | 0.032159897 |
| blue | TLR5 | MARCKS | 0.022258563 |
| blue | TLR5 | LMNB1 | 0.023828821 |
| blue | TLR5 | FLOT2 | 0.028802922 |
| blue | TLR5 | BCL6 | 0.038331135 |
| blue | TLR5 | AQP9 | 0.024373002 |
| blue | TLR5 | ACSL1 | 0.040808297 |
| blue | TLR4 | STX3 | 0.020726913 |
| blue | TLR4 | SOD2 | 0.022083439 |
| blue | TLR4 | RNF149 | 0.028169016 |
| blue | TLR4 | RALB | 0.044242231 |
| blue | TLR4 | NUMB | 0.023136495 |
| blue | TLR4 | NRBF2 | 0.020430091 |
| blue | TLR4 | NFIL3 | 0.033158648 |
| blue | TLR4 | NDEL1 | 0.028275462 |
| blue | TLR4 | NCF2 | 0.020715495 |
| blue | TLR4 | MXD1 | 0.027629632 |
| blue | TLR4 | MSRB1 | 0.02146183 |
| blue | TLR4 | LYN | 0.022972221 |
| blue | TLR4 | KLHL2 | 0.023051231 |
| blue | TLR4 | GCA | 0.039086206 |
| blue | TLR4 | GAB2 | 0.033893346 |
| blue | TLR4 | CSF3R | 0.024730853 |
| blue | TLR4 | CEBPD | 0.020582477 |
| blue | TLR4 | CEBPB | 0.026032808 |
| blue | TLR4 | BCL6 | 0.024397672 |
| blue | TLR4 | AQP9 | 0.037009341 |
| blue | TLR4 | ACSL1 | 0.025936109 |
| blue | TLR1 | STX3 | 0.022564277 |
| blue | TLR1 | SNX10 | 0.021355264 |
| blue | TLR1 | SERPINA1 | 0.020274435 |
| blue | TLR1 | RNF24 | 0.034108536 |
| blue | TLR1 | RNF149 | 0.028079033 |
| blue | TLR1 | RGS2 | 0.022740547 |
| blue | TLR1 | RBM47 | 0.021023307 |
| blue | TLR1 | RALB | 0.032851953 |
| blue | TLR1 | RAF1 | 0.021729163 |
| blue | TLR1 | PYGL | 0.027653279 |
| blue | TLR1 | OSBPL11 | 0.024748176 |
| blue | TLR1 | NUMB | 0.027098596 |
| blue | TLR1 | NLRP12 | 0.024334275 |
| blue | TLR1 | NDEL1 | 0.027458523 |

| blue | TLR1 | NADK | 0.021705967 |
| --- | --- | --- | --- |
| blue | TLR1 | MXD1 | 0.042258509 |
| blue | TLR1 | MSRB1 | 0.030837876 |
| blue | TLR1 | MSL1 | 0.021124722 |
| blue | TLR1 | MBOAT7 | 0.024810376 |
| blue | TLR1 | LYN | 0.028727574 |
| blue | TLR1 | IL1RN | 0.020314315 |
| blue | TLR1 | HCK | 0.029709827 |
| blue | TLR1 | GCA | 0.028497902 |
| blue | TLR1 | GAB2 | 0.034813952 |
| blue | TLR1 | DYSF | 0.024945244 |
| blue | TLR1 | CSF3R | 0.024262565 |
| blue | TLR1 | BCL6 | 0.023140001 |
| blue | TLR1 | BCL3 | 0.021944211 |
| blue | TLR1 | BASP1 | 0.028470674 |
| blue | TLR1 | AQP9 | 0.028298346 |
| blue | TLR1 | ACSL1 | 0.02354787 |
| blue | TLE4 | SYNJ1 | 0.023756385 |
| blue | TLE4 | SVIL | 0.038081486 |
| blue | TLE4 | RNF24 | 0.023990081 |
| blue | TLE4 | RGS2 | 0.026297212 |
| blue | TLE4 | PYGL | 0.029492233 |
| blue | TLE4 | NLRP12 | 0.024836716 |
| blue | TLE4 | MYO1F | 0.021393342 |
| blue | TLE4 | MXD1 | 0.031241832 |
| blue | TLE4 | LYST | 0.024025932 |
| blue | TLE4 | LAT2 | 0.020193558 |
| blue | TLE4 | DYSF | 0.022368962 |
| blue | TLE4 | CUX1 | 0.033349688 |
| blue | TLE4 | BAZ2B | 0.021399405 |
| blue | TLE3 | TBC1D14 | 0.023710377 |
| blue | TLE3 | SORL1 | 0.023870226 |
| blue | TLE3 | RNF24 | 0.033135461 |
| blue | TLE3 | RNF149 | 0.024988904 |
| blue | TLE3 | RGS2 | 0.025577158 |
| blue | TLE3 | PYGL | 0.021585931 |
| blue | TLE3 | PELI2 | 0.034727534 |
| blue | TLE3 | NDEL1 | 0.023853959 |
| blue | TLE3 | NADK | 0.032170175 |
| blue | TLE3 | MYO1F | 0.021271534 |
| blue | TLE3 | MXD1 | 0.041886237 |
| blue | TLE3 | MSL1 | 0.041276717 |
| blue | TLE3 | MBOAT7 | 0.036607578 |
| blue | TLE3 | ICAM3 | 0.020360336 |
| blue | TLE3 | GAB2 | 0.036528978 |
| blue | TLE3 | DYSF | 0.030561283 |
| blue | TLE3 | CXCR1 | 0.021462016 |
| blue | TLE3 | C5AR1 | 0.024704589 |
| blue | TLE3 | BASP1 | 0.025541619 |
| blue | TLE3 | ARAP1 | 0.022114645 |
| blue | TKT | TIMP2 | 0.03282615 |
| blue | TKT | TALDO1 | 0.023239624 |

| blue | TKT | SYK | 0.021956716 |
| --- | --- | --- | --- |
| blue | TKT | RXRA | 0.020771637 |
| blue | TKT | RTN3 | 0.031437662 |
| blue | TKT | PRAM1 | 0.028668189 |
| blue | TKT | PLXDC2 | 0.020315832 |
| blue | TKT | MSRB1 | 0.037318459 |
| blue | TKT | LCP1 | 0.028413939 |
| blue | TKT | GPAT3 | 0.024328909 |
| blue | TKT | GAB2 | 0.025179335 |
| blue | TKT | ATP6V1B2 | 0.036181085 |
| blue | TKT | ARID3A | 0.022523158 |
| blue | TIMP2 | TALDO1 | 0.026920597 |
| blue | TIMP2 | RXRA | 0.020260946 |
| blue | TIMP2 | RTN3 | 0.03179348 |
| blue | TIMP2 | RRAGD | 0.022502457 |
| blue | TIMP2 | RNF24 | 0.023364121 |
| blue | TIMP2 | RGS2 | 0.022881458 |
| blue | TIMP2 | RASSF2 | 0.020191591 |
| blue | TIMP2 | PYGL | 0.021254019 |
| blue | TIMP2 | PELI2 | 0.021182822 |
| blue | TIMP2 | OSBPL11 | 0.020744092 |
| blue | TIMP2 | NCF1C | 0.022842593 |
| blue | TIMP2 | NADK | 0.023779834 |
| blue | TIMP2 | MXD1 | 0.034434815 |
| blue | TIMP2 | MSRB1 | 0.034355966 |
| blue | TIMP2 | MSL1 | 0.02292541 |
| blue | TIMP2 | MME | 0.02116301 |
| blue | TIMP2 | MBOAT7 | 0.022806825 |
| blue | TIMP2 | LAT2 | 0.033794762 |
| blue | TIMP2 | GPAT3 | 0.025273253 |
| blue | TIMP2 | GAB2 | 0.037042363 |
| blue | TIMP2 | ATP6V1B2 | 0.037567133 |
| blue | TIMP2 | ARID3A | 0.034818393 |
| blue | TIMP2 | ARAP1 | 0.021010989 |
| blue | THOC5 | STX3 | 0.038655215 |
| blue | THOC5 | SLC2A3 | 0.021667365 |
| blue | THOC5 | ROPN1L | 0.033044491 |
| blue | THOC5 | RNF149 | 0.031151118 |
| blue | THOC5 | RBM47 | 0.020160047 |
| blue | THOC5 | RALB | 0.044019339 |
| blue | THOC5 | RAF1 | 0.026666566 |
| blue | THOC5 | PFKFB4 | 0.020029986 |
| blue | THOC5 | OSBPL2 | 0.030364083 |
| blue | THOC5 | NUMB | 0.037157345 |
| blue | THOC5 | NFIL3 | 0.039106092 |
| blue | THOC5 | NDEL1 | 0.044519902 |
| blue | THOC5 | NCF4 | 0.020898688 |
| blue | THOC5 | NAMPT | 0.025210842 |
| blue | THOC5 | MXD1 | 0.03077072 |
| blue | THOC5 | MSRB1 | 0.033913623 |
| blue | THOC5 | MME | 0.020964908 |
| blue | THOC5 | MGAM | 0.026700612 |

| blue | THOC5 | LRG1 | 0.020402319 |
| --- | --- | --- | --- |
| blue | THOC5 | KLHL2 | 0.020226894 |
| blue | THOC5 | ITPRIP | 0.033279662 |
| blue | THOC5 | GCA | 0.030117077 |
| blue | THOC5 | GAB2 | 0.036473624 |
| blue | THOC5 | FPR1 | 0.037551167 |
| blue | THOC5 | CSF3R | 0.036833357 |
| blue | THOC5 | CHST15 | 0.020504949 |
| blue | THOC5 | CHMP2A | 0.02460961 |
| blue | THOC5 | CEBPB | 0.040495599 |
| blue | THOC5 | BCL6 | 0.04806339 |
| blue | THOC5 | AQP9 | 0.062483347 |
| blue | THOC5 | ACSL1 | 0.039131178 |
| blue | TGFA | RNF24 | 0.029617582 |
| blue | TGFA | RNF149 | 0.02602253 |
| blue | TGFA | RALB | 0.02466051 |
| blue | TGFA | RAF1 | 0.028594124 |
| blue | TGFA | PYGL | 0.03188535 |
| blue | TGFA | NFIL3 | 0.020055359 |
| blue | TGFA | NDEL1 | 0.037356831 |
| blue | TGFA | MXD1 | 0.04357797 |
| blue | TGFA | MSRB1 | 0.021582658 |
| blue | TGFA | MSL1 | 0.022610745 |
| blue | TGFA | MME | 0.020689405 |
| blue | TGFA | GCA | 0.022452619 |
| blue | TGFA | GAB2 | 0.033659826 |
| blue | TGFA | DYSF | 0.023023617 |
| blue | TGFA | CYB5R4 | 0.020582535 |
| blue | TGFA | BCL6 | 0.028847475 |
| blue | TGFA | BASP1 | 0.021461444 |
| blue | TGFA | AQP9 | 0.028073698 |
| blue | TGFA | ACSL1 | 0.027037579 |
| blue | TBC1D24 | RNF24 | 0.021041128 |
| blue | TBC1D24 | RNF149 | 0.025260899 |
| blue | TBC1D24 | NDEL1 | 0.02021579 |
| blue | TBC1D24 | LITAF | 0.041299101 |
| blue | TBC1D24 | BCL6 | 0.021828538 |
| blue | TBC1D24 | BCL3 | 0.023120773 |
| blue | TBC1D24 | BASP1 | 0.028654102 |
| blue | TBC1D24 | AQP9 | 0.023651856 |
| blue | TBC1D24 | ACSL1 | 0.026111186 |
| blue | TBC1D14 | SORL1 | 0.029844201 |
| blue | TBC1D14 | SH3BP5L | 0.028142959 |
| blue | TBC1D14 | RTN3 | 0.028238412 |
| blue | TBC1D14 | RNF24 | 0.030543825 |
| blue | TBC1D14 | RGS2 | 0.021819765 |
| blue | TBC1D14 | RASSF2 | 0.023422202 |
| blue | TBC1D14 | PYGL | 0.026569996 |
| blue | TBC1D14 | PLPPR2 | 0.028671138 |
| blue | TBC1D14 | PELI2 | 0.021960065 |
| blue | TBC1D14 | NSMCE4A | 0.020092492 |
| blue | TBC1D14 | NADK | 0.027857728 |

| blue | TBC1D14 | MYO1F | 0.0200576 |
| --- | --- | --- | --- |
| blue | TBC1D14 | MXD1 | 0.03718814 |
| blue | TBC1D14 | MSL1 | 0.038750991 |
| blue | TBC1D14 | MME | 0.021853523 |
| blue | TBC1D14 | MGAM | 0.022739065 |
| blue | TBC1D14 | MBOAT7 | 0.026254563 |
| blue | TBC1D14 | KIF1B | 0.022607536 |
| blue | TBC1D14 | IQSEC1 | 0.024622178 |
| blue | TBC1D14 | IQGAP1 | 0.021854517 |
| blue | TBC1D14 | ICAM3 | 0.022845712 |
| blue | TBC1D14 | GAB2 | 0.037720631 |
| blue | TBC1D14 | EXTL3 | 0.023633064 |
| blue | TBC1D14 | ETS2 | 0.02352788 |
| blue | TBC1D14 | DYSF | 0.033510798 |
| blue | TBC1D14 | CXCR1 | 0.020534139 |
| blue | TBC1D14 | CPD | 0.027291712 |
| blue | TBC1D14 | CCNJL | 0.023436407 |
| blue | TBC1D14 | BASP1 | 0.020799097 |
| blue | TBC1D14 | ARID3A | 0.026750687 |
| blue | TBC1D14 | ARAP1 | 0.023940195 |
| blue | TALDO1 | STX3 | 0.026071433 |
| blue | TALDO1 | SLCO3A1 | 0.021799144 |
| blue | TALDO1 | SLC16A3 | 0.026683941 |
| blue | TALDO1 | SIRPA | 0.025449966 |
| blue | TALDO1 | S100A11 | 0.021457428 |
| blue | TALDO1 | RTN3 | 0.0242923 |
| blue | TALDO1 | RRAGD | 0.02401112 |
| blue | TALDO1 | RNF24 | 0.022322263 |
| blue | TALDO1 | RNF149 | 0.024803126 |
| blue | TALDO1 | RNF130 | 0.026971559 |
| blue | TALDO1 | RHOG | 0.038524201 |
| blue | TALDO1 | RBM47 | 0.021034065 |
| blue | TALDO1 | RALB | 0.028709878 |
| blue | TALDO1 | RAB31 | 0.02541431 |
| blue | TALDO1 | PTEN | 0.025778984 |
| blue | TALDO1 | PLXDC2 | 0.022929175 |
| blue | TALDO1 | PLBD1 | 0.024341851 |
| blue | TALDO1 | PILRA | 0.023065871 |
| blue | TALDO1 | PGD | 0.022688311 |
| blue | TALDO1 | PELI2 | 0.021685178 |
| blue | TALDO1 | PELI1 | 0.021151944 |
| blue | TALDO1 | OSBPL2 | 0.020380592 |
| blue | TALDO1 | OSBPL11 | 0.021019826 |
| blue | TALDO1 | NUP214 | 0.024097844 |
| blue | TALDO1 | NUMB | 0.027328901 |
| blue | TALDO1 | NRBF2 | 0.021591575 |
| blue | TALDO1 | NDEL1 | 0.026459664 |
| blue | TALDO1 | NCOA1 | 0.020128641 |
| blue | TALDO1 | NCF2 | 0.030023368 |
| blue | TALDO1 | NCF1C | 0.020232819 |
| blue | TALDO1 | NADK | 0.025619282 |
| blue | TALDO1 | MXD1 | 0.034717832 |

| blue | TALDO1 | MSRB1 | 0.031415401 |
| --- | --- | --- | --- |
| blue | TALDO1 | MME | 0.023119805 |
| blue | TALDO1 | MBOAT7 | 0.023052334 |
| blue | TALDO1 | LYN | 0.026188128 |
| blue | TALDO1 | LRRK2 | 0.020359697 |
| blue | TALDO1 | KIAA0513 | 0.021618102 |
| blue | TALDO1 | ITPRIP | 0.026878888 |
| blue | TALDO1 | IGF2R | 0.020070961 |
| blue | TALDO1 | IFNGR2 | 0.024273757 |
| blue | TALDO1 | HSPA1A | 0.021693208 |
| blue | TALDO1 | HLX | 0.025424117 |
| blue | TALDO1 | HCK | 0.021886702 |
| blue | TALDO1 | GCA | 0.027927203 |
| blue | TALDO1 | GAB2 | 0.038580083 |
| blue | TALDO1 | FPR1 | 0.030921538 |
| blue | TALDO1 | FCGRT | 0.027940765 |
| blue | TALDO1 | FCGR2A | 0.020426289 |
| blue | TALDO1 | EXOC6 | 0.022958808 |
| blue | TALDO1 | EVI2B | 0.026235453 |
| blue | TALDO1 | CYB5R4 | 0.020353787 |
| blue | TALDO1 | CXCR2 | 0.026139096 |
| blue | TALDO1 | CXCR1 | 0.024808974 |
| blue | TALDO1 | CSF3R | 0.030872246 |
| blue | TALDO1 | CPD | 0.02447212 |
| blue | TALDO1 | COP1 | 0.023455721 |
| blue | TALDO1 | CMTM6 | 0.020623752 |
| blue | TALDO1 | CMTM2 | 0.028859217 |
| blue | TALDO1 | CEBPB | 0.023473579 |
| blue | TALDO1 | ATP6V1B2 | 0.044724146 |
| blue | TALDO1 | ATP6V0B | 0.039398321 |
| blue | TALDO1 | ARID3A | 0.022122301 |
| blue | TALDO1 | AQP9 | 0.02643436 |
| blue | SYNJ1 | PPP1R12A | 0.021421468 |
| blue | SYNJ1 | MXD1 | 0.026474691 |
| blue | SYNJ1 | CUX1 | 0.023846249 |
| blue | SYNJ1 | BAZ2B | 0.029665993 |
| blue | SYK | ARID3A | 0.028715847 |
| blue | SYK | ARAP1 | 0.020592855 |
| blue | SVIL | SORL1 | 0.020893507 |
| blue | SVIL | RTN3 | 0.020240792 |
| blue | SVIL | RNF24 | 0.038605281 |
| blue | SVIL | RGS2 | 0.029236918 |
| blue | SVIL | PYGL | 0.042623402 |
| blue | SVIL | PPP4R1 | 0.024089172 |
| blue | SVIL | PICALM | 0.025316371 |
| blue | SVIL | PELI2 | 0.023367062 |
| blue | SVIL | NLRP12 | 0.032095093 |
| blue | SVIL | NDEL1 | 0.020551675 |
| blue | SVIL | NADK | 0.028221392 |
| blue | SVIL | MYO1F | 0.023159877 |
| blue | SVIL | MXD1 | 0.043559614 |
| blue | SVIL | MSL1 | 0.032375293 |

| blue | SVIL | MME | 0.021472075 |
| --- | --- | --- | --- |
| blue | SVIL | MGAM | 0.025251134 |
| blue | SVIL | MBOAT7 | 0.022756072 |
| blue | SVIL | LYST | 0.020426726 |
| blue | SVIL | IQSEC1 | 0.02977226 |
| blue | SVIL | HSDL2 | 0.02257401 |
| blue | SVIL | GAB2 | 0.031644983 |
| blue | SVIL | DYSF | 0.040174389 |
| blue | SVIL | DENND5A | 0.027221335 |
| blue | SVIL | CUX1 | 0.022292742 |
| blue | SVIL | CHST15 | 0.020871053 |
| blue | SVIL | CBL | 0.022109965 |
| blue | SVIL | BCL6 | 0.020447425 |
| blue | SVIL | BASP1 | 0.03245291 |
| blue | SVIL | ARAP1 | 0.022746428 |
| blue | SVIL | AGTPBP1 | 0.028969385 |
| blue | SUSD6 | STK40 | 0.021452441 |
| blue | SUSD6 | RGS2 | 0.020234272 |
| blue | SUSD6 | PELI1 | 0.027454751 |
| blue | SUSD6 | MXD1 | 0.032107869 |
| blue | SUSD6 | MBOAT7 | 0.021713581 |
| blue | SUSD6 | GAB2 | 0.020624456 |
| blue | SUSD6 | ADAM8 | 0.023082482 |
| blue | SULT1B1 | REPS2 | 0.021892239 |
| blue | SULT1B1 | RALB | 0.028659232 |
| blue | SULT1B1 | NFIL3 | 0.038293605 |
| blue | SULT1B1 | KLHL2 | 0.030641583 |
| blue | SULT1B1 | IL1R2 | 0.026630161 |
| blue | SULT1B1 | BCL6 | 0.028792175 |
| blue | SULT1B1 | AQP9 | 0.029563823 |
| blue | SULT1B1 | ACSL1 | 0.02401583 |
| blue | SULF2 | BRI3 | 0.020417612 |
| blue | STX6 | CUX1 | 0.025494688 |
| blue | STX6 | CBL | 0.02098903 |
| blue | STX6 | CAPZA2 | 0.044295791 |
| blue | STX3 | ST6GALNAC2 | 0.037318435 |
| blue | STX3 | SRGN | 0.022952996 |
| blue | STX3 | SLC2A3 | 0.026989603 |
| blue | STX3 | SLC25A44 | 0.026195139 |
| blue | STX3 | SLC16A3 | 0.025344976 |
| blue | STX3 | SIRPA | 0.036354471 |
| blue | STX3 | S100A11 | 0.023522415 |
| blue | STX3 | ROPN1L | 0.034154205 |
| blue | STX3 | RNF149 | 0.033175082 |
| blue | STX3 | RNASEL | 0.020628847 |
| blue | STX3 | REPS2 | 0.025002206 |
| blue | STX3 | RBM47 | 0.030979875 |
| blue | STX3 | RALB | 0.039971124 |
| blue | STX3 | RAF1 | 0.023055213 |
| blue | STX3 | PTEN | 0.028718868 |
| blue | STX3 | PRR13 | 0.031221305 |
| blue | STX3 | PRKCD | 0.026006675 |

| blue | STX3 | PLXNC1 | 0.028767975 |
| --- | --- | --- | --- |
| blue | STX3 | PHF21A | 0.02737494 |
| blue | STX3 | PFKFB4 | 0.042552307 |
| blue | STX3 | PANX2 | 0.021666632 |
| blue | STX3 | PACSIN2 | 0.025854578 |
| blue | STX3 | OSBPL2 | 0.029297381 |
| blue | STX3 | OSBPL11 | 0.021209341 |
| blue | STX3 | NUMB | 0.04401116 |
| blue | STX3 | NT5C2 | 0.02425733 |
| blue | STX3 | NRBF2 | 0.023593271 |
| blue | STX3 | NIBAN1 | 0.02460039 |
| blue | STX3 | NFIL3 | 0.034055464 |
| blue | STX3 | NDEL1 | 0.030514144 |
| blue | STX3 | NCOA1 | 0.023944152 |
| blue | STX3 | NCF4 | 0.031743167 |
| blue | STX3 | NCF2 | 0.024188394 |
| blue | STX3 | NAMPT | 0.028316046 |
| blue | STX3 | MXD1 | 0.04050867 |
| blue | STX3 | MTMR10 | 0.020329151 |
| blue | STX3 | MSRB1 | 0.037752037 |
| blue | STX3 | MMP25 | 0.03567919 |
| blue | STX3 | MME | 0.022807008 |
| blue | STX3 | MIR223 | 0.025121613 |
| blue | STX3 | MGAM2 | 0.02069177 |
| blue | STX3 | MGAM | 0.026004976 |
| blue | STX3 | MEGF9 | 0.022139696 |
| blue | STX3 | MCL1 | 0.021965593 |
| blue | STX3 | 1-Mar | 0.021891754 |
| blue | STX3 | MANSC1 | 0.020180465 |
| blue | STX3 | LYN | 0.020284391 |
| blue | STX3 | LY96 | 0.027254239 |
| blue | STX3 | LRRK2 | 0.021291842 |
| blue | STX3 | LRG1 | 0.034073554 |
| blue | STX3 | LPGAT1 | 0.024981191 |
| blue | STX3 | LILRA2 | 0.020203514 |
| blue | STX3 | KLHL2 | 0.029816163 |
| blue | STX3 | KCNJ15 | 0.023559129 |
| blue | STX3 | ITPRIP | 0.040970737 |
| blue | STX3 | IGF2R | 0.023508505 |
| blue | STX3 | IFNGR2 | 0.026379138 |
| blue | STX3 | IFNGR1 | 0.028844753 |
| blue | STX3 | IFITM2 | 0.033795798 |
| blue | STX3 | HSPA1A | 0.031017046 |
| blue | STX3 | HMGCR | 0.021448606 |
| blue | STX3 | HLX | 0.023351623 |
| blue | STX3 | HIST2H2AC | 0.026005763 |
| blue | STX3 | HIST1H2BC | 0.022684864 |
| blue | STX3 | HCK | 0.0243864 |
| blue | STX3 | HAL | 0.026000681 |
| blue | STX3 | GCA | 0.033566351 |
| blue | STX3 | GAB2 | 0.039706195 |
| blue | STX3 | FRAT2 | 0.021512542 |

| blue | STX3 | FPR1 | 0.041375374 |
| --- | --- | --- | --- |
| blue | STX3 | FLOT2 | 0.02875789 |
| blue | STX3 | FGR | 0.031719835 |
| blue | STX3 | FCGR2A | 0.023778428 |
| blue | STX3 | EXOC6 | 0.045329575 |
| blue | STX3 | EVI2B | 0.021872265 |
| blue | STX3 | ETS2 | 0.024493417 |
| blue | STX3 | DHX34 | 0.020578491 |
| blue | STX3 | CYB5R4 | 0.020006771 |
| blue | STX3 | CXCR1 | 0.027624754 |
| blue | STX3 | CTBS | 0.021439588 |
| blue | STX3 | CSF3R | 0.046111129 |
| blue | STX3 | CPD | 0.028043459 |
| blue | STX3 | COP1 | 0.034491145 |
| blue | STX3 | CMTM2 | 0.027640562 |
| blue | STX3 | CHST15 | 0.0300102 |
| blue | STX3 | CFLAR | 0.022061415 |
| blue | STX3 | CELF2 | 0.025603898 |
| blue | STX3 | CEBPB | 0.042491492 |
| blue | STX3 | CEACAM3 | 0.026564857 |
| blue | STX3 | C1RL | 0.026279866 |
| blue | STX3 | BEST1 | 0.030854728 |
| blue | STX3 | BCL6 | 0.038113934 |
| blue | STX3 | BCL2A1 | 0.0225499 |
| blue | STX3 | B4GALT5 | 0.026135646 |
| blue | STX3 | ATP6V1B2 | 0.026388176 |
| blue | STX3 | ATP6V0B | 0.023357797 |
| blue | STX3 | AQP9 | 0.046473431 |
| blue | STX3 | ALOX5 | 0.026345686 |
| blue | STX3 | AGO4 | 0.021040294 |
| blue | STX3 | ADGRE5 | 0.031908276 |
| blue | STX3 | ACSL1 | 0.030330758 |
| blue | STX3 | ACOX1 | 0.021752265 |
| blue | STX3 | ABTB1 | 0.024532252 |
| blue | STX11 | RNF149 | 0.027019399 |
| blue | STX11 | RGS2 | 0.020854893 |
| blue | STX11 | RALB | 0.039570584 |
| blue | STX11 | NFIL3 | 0.020306552 |
| blue | STX11 | NDEL1 | 0.021839761 |
| blue | STX11 | MXD1 | 0.02430208 |
| blue | STX11 | MSRB1 | 0.023354237 |
| blue | STX11 | LYN | 0.037701853 |
| blue | STX11 | LITAF | 0.022868989 |
| blue | STX11 | ITM2B | 0.02147613 |
| blue | STX11 | GCA | 0.03600685 |
| blue | STX11 | GAB2 | 0.025572397 |
| blue | STX11 | CPSF4 | 0.022792548 |
| blue | STX11 | BASP1 | 0.023848612 |
| blue | STX11 | AQP9 | 0.027988968 |
| blue | STK40 | RNF24 | 0.023429202 |
| blue | STK40 | RNF19B | 0.026339032 |
| blue | STK40 | RGS2 | 0.02702957 |

| blue | STK40 | POLR2H | 0.020065653 |
| --- | --- | --- | --- |
| blue | STK40 | PELI2 | 0.023527762 |
| blue | STK40 | NDEL1 | 0.021493142 |
| blue | STK40 | NADK | 0.023013963 |
| blue | STK40 | MXD1 | 0.033972478 |
| blue | STK40 | MSL1 | 0.02490123 |
| blue | STK40 | MBOAT7 | 0.022616923 |
| blue | STK40 | MAST3 | 0.026998342 |
| blue | STK40 | LYN | 0.020110755 |
| blue | STK40 | GAB2 | 0.023664452 |
| blue | STK40 | C5AR1 | 0.027552372 |
| blue | STK40 | ARHGAP25 | 0.022594695 |
| blue | STK3 | RNF24 | 0.024358483 |
| blue | STK3 | LYN | 0.022331876 |
| blue | STK3 | LTBR | 0.023774333 |
| blue | STEAP4 | RALB | 0.023136839 |
| blue | STEAP4 | QPCT | 0.027504412 |
| blue | STEAP4 | NFIL3 | 0.020437349 |
| blue | STEAP4 | NAMPT | 0.02639861 |
| blue | STEAP4 | GCA | 0.021894465 |
| blue | STEAP4 | AQP9 | 0.024055094 |
| blue | STEAP4 | ACSL1 | 0.021569313 |
| blue | STAT3 | SORL1 | 0.027546822 |
| blue | STAT3 | RTN3 | 0.021597691 |
| blue | STAT3 | MXD1 | 0.024795848 |
| blue | STAT3 | MSL1 | 0.023996839 |
| blue | STAT3 | MEFV | 0.02206283 |
| blue | STAT3 | ICAM3 | 0.02274231 |
| blue | STAT3 | GAB2 | 0.021469946 |
| blue | STAT3 | ARID3A | 0.022846156 |
| blue | ST8SIA4 | SPAG9 | 0.021287425 |
| blue | ST8SIA4 | MXD1 | 0.023849948 |
| blue | ST6GALNAC | RNF149 | 0.033335304 |
| blue | ST6GALNAC | REPS2 | 0.023081864 |
| blue | ST6GALNAC | RALB | 0.042113308 |
| blue | ST6GALNAC | RAF1 | 0.025247919 |
| blue | ST6GALNAC | PFKFB4 | 0.020742289 |
| blue | ST6GALNAC | OSBPL2 | 0.024513884 |
| blue | ST6GALNAC | NUMB | 0.030656403 |
| blue | ST6GALNAC | NFIL3 | 0.033574099 |
| blue | ST6GALNAC | NDEL1 | 0.038938053 |
| blue | ST6GALNAC | NAMPT | 0.02251259 |
| blue | ST6GALNAC | MXD1 | 0.035734112 |
| blue | ST6GALNAC | MSRB1 | 0.031199649 |
| blue | ST6GALNAC | MGAM | 0.020468275 |
| blue | ST6GALNAC | KLHL2 | 0.022278924 |
| blue | ST6GALNAC | ITPRIP | 0.025035807 |
| blue | ST6GALNAC | GCA | 0.036416265 |
| blue | ST6GALNAC | GAB2 | 0.04059746 |
| blue | ST6GALNAC | CSF3R | 0.033671984 |
| blue | ST6GALNAC | CHST15 | 0.026310263 |
| blue | ST6GALNAC | CEBPB | 0.035553383 |

| blue | ST6GALNAC | BCL6 | 0.036515212 |
| --- | --- | --- | --- |
| blue | ST6GALNAC | AQP9 | 0.053861349 |
| blue | ST6GALNAC | ACSL1 | 0.02692461 |
| blue | SRSF2 | RPAIN | 0.046663298 |
| blue | SRPK1 | SLC37A3 | 0.042013126 |
| blue | SRPK1 | RNF24 | 0.022604653 |
| blue | SRPK1 | RNF149 | 0.024763505 |
| blue | SRPK1 | RALB | 0.026438799 |
| blue | SRPK1 | QPCT | 0.020701097 |
| blue | SRPK1 | PYGL | 0.040260254 |
| blue | SRPK1 | PHF21A | 0.022424825 |
| blue | SRPK1 | PFKFB3 | 0.026208847 |
| blue | SRPK1 | NFIL3 | 0.033036015 |
| blue | SRPK1 | NAMPT | 0.031490455 |
| blue | SRPK1 | MXD1 | 0.022953839 |
| blue | SRPK1 | MGAM | 0.028083383 |
| blue | SRPK1 | KCNJ15 | 0.021333337 |
| blue | SRPK1 | GCA | 0.027098426 |
| blue | SRPK1 | DYSF | 0.030453549 |
| blue | SRPK1 | BCL6 | 0.057720336 |
| blue | SRPK1 | BCL3 | 0.0214947 |
| blue | SRPK1 | BASP1 | 0.035588783 |
| blue | SRPK1 | AQP9 | 0.035982972 |
| blue | SRPK1 | ANXA3 | 0.020674257 |
| blue | SRPK1 | ACSL1 | 0.058851428 |
| blue | SRGN | SLC49A4 | 0.02250948 |
| blue | SRGN | SERPINB1 | 0.021792461 |
| blue | SRGN | ROPN1L | 0.020659294 |
| blue | SRGN | RNF149 | 0.031839864 |
| blue | SRGN | RALB | 0.052795933 |
| blue | SRGN | QPCT | 0.02359149 |
| blue | SRGN | NUMB | 0.028698115 |
| blue | SRGN | NRBF2 | 0.034210297 |
| blue | SRGN | NFIL3 | 0.046654244 |
| blue | SRGN | NDEL1 | 0.026977673 |
| blue | SRGN | NCF2 | 0.024951898 |
| blue | SRGN | NAMPT | 0.051902539 |
| blue | SRGN | MSRB1 | 0.028705595 |
| blue | SRGN | MNDA | 0.061548326 |
| blue | SRGN | LRG1 | 0.020237822 |
| blue | SRGN | LMNB1 | 0.022302258 |
| blue | SRGN | IFNGR1 | 0.024604359 |
| blue | SRGN | HSD17B11 | 0.032193124 |
| blue | SRGN | GCA | 0.050185415 |
| blue | SRGN | GAB2 | 0.023374603 |
| blue | SRGN | CYB5R4 | 0.022168224 |
| blue | SRGN | CSF3R | 0.026208894 |
| blue | SRGN | CEBPB | 0.045323824 |
| blue | SRGN | CD58 | 0.022451667 |
| blue | SRGN | BCL6 | 0.035721257 |
| blue | SRGN | AQP9 | 0.064556599 |
| blue | SRGN | ACSL1 | 0.033975676 |

| blue | SPOPL | RALB | 0.022313568 |
| --- | --- | --- | --- |
| blue | SPOPL | MXD1 | 0.026721003 |
| blue | SPOPL | BCL6 | 0.030822832 |
| blue | SPOPL | AQP9 | 0.023137578 |
| blue | SPAG9 | RNF24 | 0.021838102 |
| blue | SPAG9 | RAF1 | 0.022828371 |
| blue | SPAG9 | PELI2 | 0.027901928 |
| blue | SPAG9 | PELI1 | 0.029663554 |
| blue | SPAG9 | NDEL1 | 0.025605921 |
| blue | SPAG9 | NADK | 0.021598844 |
| blue | SPAG9 | MXD1 | 0.06017782 |
| blue | SPAG9 | MSL1 | 0.027571928 |
| blue | SPAG9 | MAST3 | 0.021478813 |
| blue | SPAG9 | LRRK2 | 0.020305562 |
| blue | SPAG9 | GAB2 | 0.036730561 |
| blue | SPAG9 | CSF3R | 0.022387603 |
| blue | SPAG9 | ANKRD13A | 0.023804307 |
| blue | SORL1 | RNF24 | 0.025425629 |
| blue | SORL1 | RNF216P1 | 0.024218831 |
| blue | SORL1 | RGS2 | 0.02406875 |
| blue | SORL1 | RASSF2 | 0.028010754 |
| blue | SORL1 | PREX1 | 0.02047882 |
| blue | SORL1 | PPP4R1 | 0.024349935 |
| blue | SORL1 | PLPPR2 | 0.020183824 |
| blue | SORL1 | PKN2 | 0.026238458 |
| blue | SORL1 | PIK3CD | 0.027536807 |
| blue | SORL1 | PICALM | 0.051054737 |
| blue | SORL1 | PHF2 | 0.039212186 |
| blue | SORL1 | PELI2 | 0.023505592 |
| blue | SORL1 | NSMCE4A | 0.021172268 |
| blue | SORL1 | NIN | 0.021067972 |
| blue | SORL1 | NADK | 0.026088455 |
| blue | SORL1 | MYO1F | 0.023384987 |
| blue | SORL1 | MXD1 | 0.033522566 |
| blue | SORL1 | MSL1 | 0.035374712 |
| blue | SORL1 | MEFV | 0.030902795 |
| blue | SORL1 | MED13L | 0.033226425 |
| blue | SORL1 | MBOAT7 | 0.02152076 |
| blue | SORL1 | MAP2K4 | 0.02565159 |
| blue | SORL1 | IQSEC1 | 0.037652865 |
| blue | SORL1 | IQGAP1 | 0.038839111 |
| blue | SORL1 | ICAM3 | 0.021716424 |
| blue | SORL1 | GAB2 | 0.023376009 |
| blue | SORL1 | EXTL3 | 0.020522 |
| blue | SORL1 | DYSF | 0.024020532 |
| blue | SORL1 | DCP2 | 0.027777779 |
| blue | SORL1 | CUX1 | 0.020337146 |
| blue | SORL1 | CREBRF | 0.020057167 |
| blue | SORL1 | CDC42SE1 | 0.028570336 |
| blue | SORL1 | CANT1 | 0.031383257 |
| blue | SORL1 | ARID3A | 0.024820918 |
| blue | SORL1 | ARAP1 | 0.02062367 |

| blue | SORL1 | ANTXR2 | 0.033502315 |
| --- | --- | --- | --- |
| blue | SORL1 | ANKRD13A | 0.021318842 |
| blue | SORL1 | AGTPBP1 | 0.024527594 |
| blue | SOD2 | RNF149 | 0.02974175 |
| blue | SOD2 | RALB | 0.041911093 |
| blue | SOD2 | NUMB | 0.025138162 |
| blue | SOD2 | NRBF2 | 0.023425498 |
| blue | SOD2 | NIBAN1 | 0.031844342 |
| blue | SOD2 | NFIL3 | 0.027844112 |
| blue | SOD2 | NDEL1 | 0.026762168 |
| blue | SOD2 | NAMPT | 0.022183641 |
| blue | SOD2 | MXD1 | 0.021053205 |
| blue | SOD2 | MSRB1 | 0.022273664 |
| blue | SOD2 | GK | 0.022294826 |
| blue | SOD2 | GCA | 0.031397029 |
| blue | SOD2 | GAB2 | 0.031334155 |
| blue | SOD2 | FCGR2A | 0.039048445 |
| blue | SOD2 | ETS2 | 0.021710662 |
| blue | SOD2 | CXCR2 | 0.024186269 |
| blue | SOD2 | CXCR1 | 0.03220463 |
| blue | SOD2 | CSF3R | 0.031533646 |
| blue | SOD2 | CEBPB | 0.023891788 |
| blue | SOD2 | BCL6 | 0.027607846 |
| blue | SOD2 | AQP9 | 0.049822315 |
| blue | SOD2 | ACSL1 | 0.021096736 |
| blue | SNX27 | PFKFB4 | 0.024217071 |
| blue | SNX27 | LILRA2 | 0.023770949 |
| blue | SNX27 | IMPDH1 | 0.023097432 |
| blue | SNX10 | RNF149 | 0.023633044 |
| blue | SNX10 | RBM47 | 0.02057203 |
| blue | SNX10 | RALB | 0.046022735 |
| blue | SNX10 | NUMB | 0.033005949 |
| blue | SNX10 | NRBF2 | 0.021506269 |
| blue | SNX10 | NFIL3 | 0.020650811 |
| blue | SNX10 | NDEL1 | 0.028613923 |
| blue | SNX10 | NCF2 | 0.024216163 |
| blue | SNX10 | MXD1 | 0.021617876 |
| blue | SNX10 | MSRB1 | 0.03672107 |
| blue | SNX10 | LYN | 0.036872476 |
| blue | SNX10 | LRRK2 | 0.020384493 |
| blue | SNX10 | GCA | 0.036551461 |
| blue | SNX10 | GAB2 | 0.024681259 |
| blue | SNX10 | EVI2B | 0.022460644 |
| blue | SNX10 | CSF3R | 0.020459085 |
| blue | SNX10 | ATP6V1B2 | 0.028258596 |
| blue | SNX10 | AQP9 | 0.029703359 |
| blue | SLCO3A1 | RNF24 | 0.033504159 |
| blue | SLCO3A1 | RGS2 | 0.021771618 |
| blue | SLCO3A1 | PYGL | 0.025020173 |
| blue | SLCO3A1 | PELI2 | 0.023182152 |
| blue | SLCO3A1 | PELI1 | 0.020167512 |
| blue | SLCO3A1 | NDEL1 | 0.020598846 |

| blue | SLCO3A1 | NADK | 0.029466845 |
| --- | --- | --- | --- |
| blue | SLCO3A1 | MXD1 | 0.035176313 |
| blue | SLCO3A1 | MSL1 | 0.027144205 |
| blue | SLCO3A1 | MBOAT7 | 0.021103458 |
| blue | SLCO3A1 | LYN | 0.020213407 |
| blue | SLCO3A1 | GAB2 | 0.0283179 |
| blue | SLCO3A1 | DYSF | 0.027319528 |
| blue | SLCO3A1 | BASP1 | 0.022171976 |
| blue | SLC49A4 | RALB | 0.021932403 |
| blue | SLC49A4 | NFIL3 | 0.020560379 |
| blue | SLC49A4 | GCA | 0.020530489 |
| blue | SLC49A4 | CEBPB | 0.020570157 |
| blue | SLC49A4 | AQP9 | 0.025716103 |
| blue | SLC44A2 | PACSIN2 | 0.02763837 |
| blue | SLC44A2 | ITPRIP | 0.020211336 |
| blue | SLC44A2 | CSF3R | 0.024994406 |
| blue | SLC44A2 | ABTB1 | 0.021642545 |
| blue | SLC37A4 | C5AR1 | 0.02215446 |
| blue | SLC37A4 | C16orf58 | 0.042787312 |
| blue | SLC37A3 | QPCT | 0.022242633 |
| blue | SLC37A3 | PFKFB3 | 0.049671983 |
| blue | SLC37A3 | NFIL3 | 0.020430463 |
| blue | SLC37A3 | NAMPT | 0.030471402 |
| blue | SLC37A3 | FLOT2 | 0.022086617 |
| blue | SLC37A3 | BCL6 | 0.031425102 |
| blue | SLC37A3 | AQP9 | 0.023604031 |
| blue | SLC37A3 | ACSL1 | 0.05172349 |
| blue | SLC2A3 | SLC2A14 | 0.032550703 |
| blue | SLC2A3 | RTN3 | 0.023801097 |
| blue | SLC2A3 | ROPN1L | 0.032659358 |
| blue | SLC2A3 | RNF24 | 0.020354917 |
| blue | SLC2A3 | RNF149 | 0.031030665 |
| blue | SLC2A3 | RALB | 0.032521622 |
| blue | SLC2A3 | QPCT | 0.022899022 |
| blue | SLC2A3 | PYGL | 0.03205234 |
| blue | SLC2A3 | PHF21A | 0.032011616 |
| blue | SLC2A3 | PFKFB3 | 0.024675929 |
| blue | SLC2A3 | NUMB | 0.025828627 |
| blue | SLC2A3 | NFIL3 | 0.03736372 |
| blue | SLC2A3 | NDEL1 | 0.026321764 |
| blue | SLC2A3 | NCF4 | 0.02272341 |
| blue | SLC2A3 | NAMPT | 0.030632235 |
| blue | SLC2A3 | MXD1 | 0.024494901 |
| blue | SLC2A3 | MSRB1 | 0.037990867 |
| blue | SLC2A3 | MME | 0.024360384 |
| blue | SLC2A3 | MGAM | 0.032465609 |
| blue | SLC2A3 | ITPRIP | 0.023060213 |
| blue | SLC2A3 | GCA | 0.03054376 |
| blue | SLC2A3 | GAB2 | 0.026522255 |
| blue | SLC2A3 | FLOT2 | 0.024798233 |
| blue | SLC2A3 | DYSF | 0.03033876 |
| blue | SLC2A3 | CSF3R | 0.02426029 |

| blue | SLC2A3 | CEBPB | 0.026290287 |
| --- | --- | --- | --- |
| blue | SLC2A3 | BCL6 | 0.054840129 |
| blue | SLC2A3 | BASP1 | 0.032249049 |
| blue | SLC2A3 | AQP9 | 0.056828302 |
| blue | SLC2A3 | ACSL1 | 0.069949265 |
| blue | SLC2A14 | RTN3 | 0.026646493 |
| blue | SLC2A14 | ROPN1L | 0.025015404 |
| blue | SLC2A14 | RNF149 | 0.021181857 |
| blue | SLC2A14 | RALB | 0.0269506 |
| blue | SLC2A14 | PYGL | 0.034509462 |
| blue | SLC2A14 | PHF21A | 0.031232956 |
| blue | SLC2A14 | PFKFB3 | 0.020822353 |
| blue | SLC2A14 | NFIL3 | 0.031014025 |
| blue | SLC2A14 | NAMPT | 0.022010585 |
| blue | SLC2A14 | MXD1 | 0.023358731 |
| blue | SLC2A14 | MSRB1 | 0.020278643 |
| blue | SLC2A14 | MME | 0.03408274 |
| blue | SLC2A14 | MGAM2 | 0.034673464 |
| blue | SLC2A14 | MGAM | 0.059017332 |
| blue | SLC2A14 | GCA | 0.025577182 |
| blue | SLC2A14 | GAB2 | 0.025302178 |
| blue | SLC2A14 | DYSF | 0.03364699 |
| blue | SLC2A14 | BCL6 | 0.050174452 |
| blue | SLC2A14 | BASP1 | 0.030553651 |
| blue | SLC2A14 | AQP9 | 0.041124329 |
| blue | SLC2A14 | ACSL1 | 0.04714766 |
| blue | SLC25A44 | SIRPA | 0.02622917 |
| blue | SLC25A44 | RALB | 0.020741365 |
| blue | SLC25A44 | PFKFB4 | 0.035416355 |
| blue | SLC25A44 | PACSIN2 | 0.040652797 |
| blue | SLC25A44 | OSBPL2 | 0.021382077 |
| blue | SLC25A44 | NUMB | 0.023399614 |
| blue | SLC25A44 | NOTCH1 | 0.045620812 |
| blue | SLC25A44 | NCF4 | 0.038485285 |
| blue | SLC25A44 | MTX1 | 0.021196702 |
| blue | SLC25A44 | MMP25 | 0.025427883 |
| blue | SLC25A44 | LRG1 | 0.025441646 |
| blue | SLC25A44 | ITPRIP | 0.047466307 |
| blue | SLC25A44 | IMPDH1 | 0.027026836 |
| blue | SLC25A44 | CSF3R | 0.048284212 |
| blue | SLC25A44 | CEBPB | 0.02751357 |
| blue | SLC25A44 | BCL6 | 0.027328568 |
| blue | SLC25A44 | AQP9 | 0.039681344 |
| blue | SLC25A44 | ABTB1 | 0.020897798 |
| blue | SLC22A4 | ROPN1L | 0.022581521 |
| blue | SLC22A4 | RNF149 | 0.02191394 |
| blue | SLC22A4 | RALB | 0.033268303 |
| blue | SLC22A4 | PHF21A | 0.025750736 |
| blue | SLC22A4 | NFIL3 | 0.042401272 |
| blue | SLC22A4 | NAMPT | 0.023271434 |
| blue | SLC22A4 | MXD1 | 0.02465364 |
| blue | SLC22A4 | MGAM | 0.022068478 |

| blue | SLC22A4 | KCNJ15 | 0.024583708 |
| --- | --- | --- | --- |
| blue | SLC22A4 | GCA | 0.025076622 |
| blue | SLC22A4 | GAB2 | 0.021086963 |
| blue | SLC22A4 | DYSF | 0.022571004 |
| blue | SLC22A4 | CYSTM1 | 0.026238173 |
| blue | SLC22A4 | BCL6 | 0.044556063 |
| blue | SLC22A4 | BASP1 | 0.022572563 |
| blue | SLC22A4 | B4GALT5 | 0.024985191 |
| blue | SLC22A4 | AQP9 | 0.038137008 |
| blue | SLC22A4 | ANXA3 | 0.029559256 |
| blue | SLC22A4 | ACSL1 | 0.051103812 |
| blue | SLC22A15 | PELI1 | 0.020311585 |
| blue | SLC22A15 | MXD1 | 0.02538968 |
| blue | SLC22A15 | GAB2 | 0.021125268 |
| blue | SLC19A1 | RNF24 | 0.028292353 |
| blue | SLC19A1 | RNF216P1 | 0.021130928 |
| blue | SLC19A1 | RGS2 | 0.025460321 |
| blue | SLC19A1 | NADK | 0.028788765 |
| blue | SLC19A1 | MXD1 | 0.028259421 |
| blue | SLC19A1 | MSL1 | 0.027016068 |
| blue | SLC19A1 | MBOAT7 | 0.026838346 |
| blue | SLC19A1 | GAB2 | 0.025620021 |
| blue | SLC19A1 | C5AR1 | 0.024659062 |
| blue | SLC19A1 | ARAP1 | 0.020619679 |
| blue | SLC16A3 | RTN3 | 0.020520039 |
| blue | SLC16A3 | RNF24 | 0.027981319 |
| blue | SLC16A3 | RNF149 | 0.030072811 |
| blue | SLC16A3 | RGS2 | 0.023945653 |
| blue | SLC16A3 | RALB | 0.029243391 |
| blue | SLC16A3 | RAF1 | 0.024522004 |
| blue | SLC16A3 | PYGL | 0.024823118 |
| blue | SLC16A3 | PPP1R3D | 0.022019461 |
| blue | SLC16A3 | PGD | 0.026805486 |
| blue | SLC16A3 | PELI2 | 0.020388537 |
| blue | SLC16A3 | NUMB | 0.0229131 |
| blue | SLC16A3 | NDEL1 | 0.035540535 |
| blue | SLC16A3 | NADK | 0.022892475 |
| blue | SLC16A3 | MXD1 | 0.045372628 |
| blue | SLC16A3 | MSRB1 | 0.042022636 |
| blue | SLC16A3 | MME | 0.020857786 |
| blue | SLC16A3 | MBOAT7 | 0.026554885 |
| blue | SLC16A3 | LYN | 0.026896021 |
| blue | SLC16A3 | GCA | 0.026455306 |
| blue | SLC16A3 | GAB2 | 0.040171104 |
| blue | SLC16A3 | G6PD | 0.023111956 |
| blue | SLC16A3 | DYSF | 0.021063121 |
| blue | SLC16A3 | CSF3R | 0.027399045 |
| blue | SLC16A3 | CHST15 | 0.020867126 |
| blue | SLC16A3 | CEBPB | 0.023944124 |
| blue | SLC16A3 | BCL6 | 0.021871907 |
| blue | SLC16A3 | BASP1 | 0.02215933 |
| blue | SLC16A3 | ATP6V1B2 | 0.027598392 |

| blue | SLC16A3 | AQP9 | 0.032080201 |
| --- | --- | --- | --- |
| blue | SLC12A9 | SLC11A1 | 0.026585491 |
| blue | SLC12A9 | PFKFB4 | 0.039195765 |
| blue | SLC12A9 | NCF4 | 0.057338931 |
| blue | SLC12A9 | MMP25 | 0.021140727 |
| blue | SLC12A9 | CSF3R | 0.023281591 |
| blue | SLC12A9 | ALOX5 | 0.023991261 |
| blue | SLC11A1 | PFKFB4 | 0.028591148 |
| blue | SLC11A1 | NCF4 | 0.037390405 |
| blue | SLC11A1 | CSF3R | 0.028997477 |
| blue | SLC11A1 | BCL6 | 0.025208991 |
| blue | SLC11A1 | AQP9 | 0.028102978 |
| blue | SLC11A1 | ALOX5 | 0.021393139 |
| blue | SLA | RGS2 | 0.024836627 |
| blue | SLA | PHC2 | 0.029538225 |
| blue | SLA | NADK | 0.02017624 |
| blue | SLA | MXD1 | 0.03174295 |
| blue | SLA | MEGF9 | 0.021049655 |
| blue | SLA | KIAA0319L | 0.021979823 |
| blue | SLA | GAB2 | 0.024530124 |
| blue | SLA | CPSF4 | 0.025208354 |
| blue | SLA | CEBPD | 0.031078863 |
| blue | SIRPA | RNF149 | 0.02008118 |
| blue | SIRPA | RALB | 0.027614877 |
| blue | SIRPA | PFKFB4 | 0.040521465 |
| blue | SIRPA | PACSIN2 | 0.021714118 |
| blue | SIRPA | NUMB | 0.035149177 |
| blue | SIRPA | NDEL1 | 0.023073608 |
| blue | SIRPA | NCF4 | 0.032972492 |
| blue | SIRPA | MXD1 | 0.024527845 |
| blue | SIRPA | MSRB1 | 0.038507463 |
| blue | SIRPA | MMP25 | 0.021742158 |
| blue | SIRPA | ITPRIP | 0.032643985 |
| blue | SIRPA | IMPDH1 | 0.027647084 |
| blue | SIRPA | IFNGR2 | 0.02399069 |
| blue | SIRPA | HSPA1A | 0.034180098 |
| blue | SIRPA | GCA | 0.020515058 |
| blue | SIRPA | GAB2 | 0.029488942 |
| blue | SIRPA | CSF3R | 0.036018604 |
| blue | SIRPA | CEBPB | 0.035059227 |
| blue | SIRPA | BCL6 | 0.024569362 |
| blue | SIRPA | ATP6V1B2 | 0.031695479 |
| blue | SIRPA | AQP9 | 0.034713683 |
| blue | SIRPA | AGO4 | 0.029630096 |
| blue | SIPA1L2 | RALB | 0.022659583 |
| blue | SIPA1L2 | NFIL3 | 0.022568098 |
| blue | SIPA1L2 | KLHL2 | 0.04465277 |
| blue | SIPA1L2 | IRAK3 | 0.020825145 |
| blue | SIPA1L2 | IL1R2 | 0.029132767 |
| blue | SIPA1L2 | FRAT1 | 0.022436269 |
| blue | SIPA1L2 | CEBPB | 0.024698508 |
| blue | SIPA1L2 | AQP9 | 0.023479484 |

| blue | SIPA1L2 | ACSL1 | 0.023565011 |
| --- | --- | --- | --- |
| blue | SH3GLB1 | RALB | 0.021574539 |
| blue | SH3GLB1 | NFIL3 | 0.03373347 |
| blue | SH3GLB1 | LMNB1 | 0.021647802 |
| blue | SH3GLB1 | BCL6 | 0.027630482 |
| blue | SH3GLB1 | AQP9 | 0.026564444 |
| blue | SH3GLB1 | ACSL1 | 0.031848017 |
| blue | SH3BP5L | RTN3 | 0.031080963 |
| blue | SH3BP5L | RNF24 | 0.02569486 |
| blue | SH3BP5L | RGS2 | 0.021299174 |
| blue | SH3BP5L | PYGL | 0.024910671 |
| blue | SH3BP5L | PLPPR2 | 0.023618791 |
| blue | SH3BP5L | NADK | 0.027148061 |
| blue | SH3BP5L | MXD1 | 0.031538167 |
| blue | SH3BP5L | MSL1 | 0.035503915 |
| blue | SH3BP5L | MME | 0.020940713 |
| blue | SH3BP5L | GAB2 | 0.021718253 |
| blue | SH3BP5L | DYSF | 0.036933231 |
| blue | SH3BP5L | ARID3A | 0.021537069 |
| blue | SH3BP5L | ARAP1 | 0.025996558 |
| blue | SERPINB1 | RALB | 0.022524898 |
| blue | SERPINB1 | NFIL3 | 0.026296488 |
| blue | SERPINB1 | NAMPT | 0.020763227 |
| blue | SERPINB1 | GCA | 0.021961937 |
| blue | SERPINB1 | AQP9 | 0.024387792 |
| blue | SERPINB1 | ACSL1 | 0.024182235 |
| blue | SERPINA1 | RNF24 | 0.029144188 |
| blue | SERPINA1 | RGS2 | 0.023014925 |
| blue | SERPINA1 | PYGL | 0.023055033 |
| blue | SERPINA1 | NLRP12 | 0.023746277 |
| blue | SERPINA1 | MXD1 | 0.020537144 |
| blue | SERPINA1 | MSRB1 | 0.026139192 |
| blue | SERPINA1 | MBOAT7 | 0.020717669 |
| blue | SERPINA1 | LYN | 0.027386641 |
| blue | SERPINA1 | GCA | 0.02301511 |
| blue | SERPINA1 | GAB2 | 0.021071752 |
| blue | SERPINA1 | DYSF | 0.021296279 |
| blue | SERPINA1 | BASP1 | 0.026416461 |
| blue | SERPINA1 | ATP6V1B2 | 0.020719915 |
| blue | SERINC1 | RGS2 | 0.035898989 |
| blue | SERINC1 | MAP2K4 | 0.030224185 |
| blue | SERINC1 | CUX1 | 0.020907532 |
| blue | SERINC1 | CBL | 0.028412262 |
| blue | SERINC1 | C5AR1 | 0.021470454 |
| blue | SEMA4A | DYSF | 0.021795809 |
| blue | SELL | RNF149 | 0.02089026 |
| blue | SELL | RALB | 0.025468634 |
| blue | SELL | MSRB1 | 0.026597638 |
| blue | SELL | GCA | 0.022683834 |
| blue | SELL | GAB2 | 0.022161366 |
| blue | SELL | DYSF | 0.021107483 |
| blue | SELL | BCL6 | 0.021601765 |

| blue | SELL | BASP1 | 0.021501255 |
| --- | --- | --- | --- |
| blue | SELL | AQP9 | 0.029074472 |
| blue | SELL | ACSL1 | 0.025256735 |
| blue | SDCBP | RNF24 | 0.022394016 |
| blue | SDCBP | RGS2 | 0.031226377 |
| blue | SDCBP | RALB | 0.020617593 |
| blue | SDCBP | PIP4P2 | 0.048430632 |
| blue | SDCBP | NCF2 | 0.021069968 |
| blue | SDCBP | LYN | 0.041581144 |
| blue | SDCBP | LRRK2 | 0.021230398 |
| blue | SDCBP | LITAF | 0.024890571 |
| blue | SDCBP | ITPR3 | 0.021264547 |
| blue | SDCBP | ITM2B | 0.056706327 |
| blue | SDCBP | HSD17B11 | 0.020817391 |
| blue | SDCBP | GCA | 0.030218821 |
| blue | SDCBP | CYB5R4 | 0.022134289 |
| blue | SDCBP | CMTM6 | 0.023252083 |
| blue | SDCBP | CHIC2 | 0.025282033 |
| blue | SDCBP | CD58 | 0.030320963 |
| blue | SDCBP | BRI3 | 0.020073912 |
| blue | SDCBP | BASP1 | 0.025844547 |
| blue | SDCBP | AKIRIN2 | 0.029155668 |
| blue | SBNO2 | RNF24 | 0.02935689 |
| blue | SBNO2 | RNF13 | 0.023714156 |
| blue | SBNO2 | RGS2 | 0.020715544 |
| blue | SBNO2 | PYGL | 0.023807975 |
| blue | SBNO2 | NADK | 0.02679707 |
| blue | SBNO2 | MXD1 | 0.031406978 |
| blue | SBNO2 | MSL1 | 0.025004556 |
| blue | SBNO2 | MBOAT7 | 0.022642095 |
| blue | SBNO2 | LYN | 0.020760855 |
| blue | SBNO2 | GAB2 | 0.022569328 |
| blue | SBNO2 | DYSF | 0.030607238 |
| blue | SBNO2 | BCL3 | 0.020687336 |
| blue | SBNO2 | BASP1 | 0.023454438 |
| blue | SBNO2 | ARAP1 | 0.023243047 |
| blue | SBNO2 | APBB1IP | 0.032678343 |
| blue | S100A9 | S100A8 | 0.081218135 |
| blue | S100A9 | S100A12 | 0.022221114 |
| blue | S100A8 | S100A12 | 0.021615107 |
| blue | S100A8 | RTN3 | 0.023426616 |
| blue | S100A8 | PYGL | 0.02792592 |
| blue | S100A8 | MSRB1 | 0.033923102 |
| blue | S100A8 | BASP1 | 0.024132225 |
| blue | S100A12 | RTN3 | 0.021155045 |
| blue | S100A12 | RGS2 | 0.020395715 |
| blue | S100A12 | QPCT | 0.027363312 |
| blue | S100A12 | PYGL | 0.030705873 |
| blue | S100A12 | NFIL3 | 0.020297311 |
| blue | S100A12 | MMP9 | 0.025699363 |
| blue | S100A12 | MME | 0.02552844 |
| blue | S100A12 | MGAM | 0.020872673 |

| blue | S100A12 | GCA | 0.021634386 |
| --- | --- | --- | --- |
| blue | S100A12 | DYSF | 0.02708004 |
| blue | S100A12 | BCL6 | 0.02053741 |
| blue | S100A12 | BASP1 | 0.024861081 |
| blue | S100A12 | ACSL1 | 0.025826496 |
| blue | S100A11 | RNF24 | 0.021007555 |
| blue | S100A11 | RNF149 | 0.030841506 |
| blue | S100A11 | RGS2 | 0.020815419 |
| blue | S100A11 | RBM47 | 0.025197128 |
| blue | S100A11 | RALB | 0.039150536 |
| blue | S100A11 | QPCT | 0.022527731 |
| blue | S100A11 | PYGL | 0.030261864 |
| blue | S100A11 | PLXDC2 | 0.036954241 |
| blue | S100A11 | NUMB | 0.02950229 |
| blue | S100A11 | NFIL3 | 0.033516879 |
| blue | S100A11 | NDEL1 | 0.029238891 |
| blue | S100A11 | NCF2 | 0.024963111 |
| blue | S100A11 | NAMPT | 0.023597867 |
| blue | S100A11 | MXD1 | 0.026938099 |
| blue | S100A11 | MSRB1 | 0.041617882 |
| blue | S100A11 | LYN | 0.027105398 |
| blue | S100A11 | IFNGR2 | 0.031852193 |
| blue | S100A11 | HSPA1A | 0.020525283 |
| blue | S100A11 | GMFG | 0.020220352 |
| blue | S100A11 | GCA | 0.042483995 |
| blue | S100A11 | GAB2 | 0.030908176 |
| blue | S100A11 | FRAT2 | 0.030901756 |
| blue | S100A11 | CSF3R | 0.020588641 |
| blue | S100A11 | CEBPB | 0.0312037 |
| blue | S100A11 | CD58 | 0.026818629 |
| blue | S100A11 | BCL6 | 0.031966271 |
| blue | S100A11 | BASP1 | 0.027001095 |
| blue | S100A11 | ATP6V1B2 | 0.025339029 |
| blue | S100A11 | AQP9 | 0.042039947 |
| blue | S100A11 | ALOX5AP | 0.023507299 |
| blue | S100A11 | ACSL1 | 0.039966189 |
| blue | RXRA | RTN3 | 0.028587678 |
| blue | RXRA | RNF24 | 0.027179222 |
| blue | RXRA | RGS2 | 0.023930345 |
| blue | RXRA | PLEKHO2 | 0.025681895 |
| blue | RXRA | PECAM1 | 0.021987715 |
| blue | RXRA | NLRP12 | 0.021732118 |
| blue | RXRA | NADK | 0.02838804 |
| blue | RXRA | MXD1 | 0.023399498 |
| blue | RXRA | MSL1 | 0.02860398 |
| blue | RXRA | MEFV | 0.02650683 |
| blue | RXRA | MBOAT7 | 0.024526496 |
| blue | RXRA | MAP3K11 | 0.026993047 |
| blue | RXRA | LAT2 | 0.024637607 |
| blue | RXRA | KIAA0513 | 0.020177899 |
| blue | RXRA | IL17RA | 0.020564914 |
| blue | RXRA | GPAT3 | 0.021077382 |

| blue | RXRA | DYSF | 0.02226148 |
| --- | --- | --- | --- |
| blue | RXRA | C5AR1 | 0.021774513 |
| blue | RXRA | ARID3A | 0.027578507 |
| blue | RXRA | ARAP1 | 0.025046192 |
| blue | RTN3 | RRAGD | 0.028237866 |
| blue | RTN3 | RNF24 | 0.026950727 |
| blue | RTN3 | RNF144B | 0.02023479 |
| blue | RTN3 | RHOG | 0.035541585 |
| blue | RTN3 | RGS2 | 0.02726509 |
| blue | RTN3 | RASSF2 | 0.028035989 |
| blue | RTN3 | PYGL | 0.02780295 |
| blue | RTN3 | PLPPR2 | 0.023952555 |
| blue | RTN3 | PIK3CD | 0.023616794 |
| blue | RTN3 | PGD | 0.032852115 |
| blue | RTN3 | PELI2 | 0.02498612 |
| blue | RTN3 | PDK3 | 0.026947173 |
| blue | RTN3 | PCNX1 | 0.02402475 |
| blue | RTN3 | PAK1 | 0.023094417 |
| blue | RTN3 | PADI4 | 0.024058532 |
| blue | RTN3 | NUP214 | 0.023183633 |
| blue | RTN3 | NLRX1 | 0.021139524 |
| blue | RTN3 | NLRP12 | 0.025695981 |
| blue | RTN3 | NDEL1 | 0.024415612 |
| blue | RTN3 | NCF1C | 0.020649879 |
| blue | RTN3 | NADK | 0.025085228 |
| blue | RTN3 | MXD1 | 0.033610796 |
| blue | RTN3 | MSRB1 | 0.032351766 |
| blue | RTN3 | MSL1 | 0.029433022 |
| blue | RTN3 | MMP9 | 0.023565946 |
| blue | RTN3 | MME | 0.041818145 |
| blue | RTN3 | MGAM2 | 0.022828136 |
| blue | RTN3 | MGAM | 0.031916711 |
| blue | RTN3 | 1-Mar | 0.020016119 |
| blue | RTN3 | MAP3K5 | 0.023991397 |
| blue | RTN3 | LCP1 | 0.033193146 |
| blue | RTN3 | LAT2 | 0.026524198 |
| blue | RTN3 | LAMP2 | 0.024133019 |
| blue | RTN3 | KIAA0513 | 0.030512137 |
| blue | RTN3 | IQSEC1 | 0.020072891 |
| blue | RTN3 | IL6R | 0.023983677 |
| blue | RTN3 | IGF2R | 0.030477988 |
| blue | RTN3 | ICAM3 | 0.034268878 |
| blue | RTN3 | HSPA1A | 0.028682437 |
| blue | RTN3 | HMGCR | 0.025211263 |
| blue | RTN3 | GPAT3 | 0.048169851 |
| blue | RTN3 | GAB2 | 0.036135346 |
| blue | RTN3 | FCGRT | 0.02616504 |
| blue | RTN3 | EXTL3 | 0.030241461 |
| blue | RTN3 | DYSF | 0.03121745 |
| blue | RTN3 | CYP4F3 | 0.022899024 |
| blue | RTN3 | CPD | 0.032238576 |
| blue | RTN3 | COP1 | 0.023503257 |

| blue | RTN3 | CMTM2 | 0.027270116 |
| --- | --- | --- | --- |
| blue | RTN3 | CKAP4 | 0.021447925 |
| blue | RTN3 | CHST15 | 0.02599252 |
| blue | RTN3 | CFP | 0.02381792 |
| blue | RTN3 | CDA | 0.031314829 |
| blue | RTN3 | CD93 | 0.024194748 |
| blue | RTN3 | CCNJL | 0.026685602 |
| blue | RTN3 | BASP1 | 0.021412206 |
| blue | RTN3 | ATP6V1B2 | 0.025421106 |
| blue | RTN3 | ATP6V0B | 0.020303712 |
| blue | RTN3 | ARID3A | 0.047816606 |
| blue | RTN3 | AQP9 | 0.022314268 |
| blue | RTN3 | ANPEP | 0.022122432 |
| blue | RTN3 | AGO4 | 0.025493929 |
| blue | RTN3 | ADGRE3 | 0.024341555 |
| blue | RTF2 | MSRB1 | 0.020554165 |
| blue | RTF2 | DAZAP2 | 0.05138822 |
| blue | RTF2 | CHMP2A | 0.027611534 |
| blue | RTF2 | CEBPB | 0.021704467 |
| blue | RRAGD | RNF24 | 0.021674265 |
| blue | RRAGD | RGS2 | 0.026231109 |
| blue | RRAGD | PYGL | 0.022477866 |
| blue | RRAGD | PELI2 | 0.024693494 |
| blue | RRAGD | NADK | 0.020428741 |
| blue | RRAGD | MXD1 | 0.035346288 |
| blue | RRAGD | MSRB1 | 0.024292421 |
| blue | RRAGD | MME | 0.026104242 |
| blue | RRAGD | GAB2 | 0.041276111 |
| blue | RRAGD | DYSF | 0.020498882 |
| blue | RRAGD | ATP6V1B2 | 0.023646997 |
| blue | RRAGD | ARID3A | 0.023038981 |
| blue | RPAIN | NSMCE4A | 0.022846538 |
| blue | RP2 | RALB | 0.030308378 |
| blue | RP2 | NFIL3 | 0.020429711 |
| blue | RP2 | GCA | 0.021173909 |
| blue | RP2 | CELF2 | 0.021857994 |
| blue | RP2 | CEBPB | 0.021672891 |
| blue | RP2 | AQP9 | 0.021240926 |
| blue | ROPN1L | RNF149 | 0.02503714 |
| blue | ROPN1L | REPS2 | 0.020436646 |
| blue | ROPN1L | RALB | 0.047202693 |
| blue | ROPN1L | QPCT | 0.031055726 |
| blue | ROPN1L | PLXNC1 | 0.022687362 |
| blue | ROPN1L | PHF21A | 0.025022889 |
| blue | ROPN1L | PFKFB4 | 0.02089441 |
| blue | ROPN1L | PFKFB3 | 0.028396142 |
| blue | ROPN1L | OSBPL2 | 0.022355022 |
| blue | ROPN1L | NUMB | 0.03078393 |
| blue | ROPN1L | NLRX1 | 0.026298504 |
| blue | ROPN1L | NFIL3 | 0.061948229 |
| blue | ROPN1L | NDEL1 | 0.027168226 |
| blue | ROPN1L | NCF4 | 0.030832297 |

| blue | ROPN1L | NAMPT | 0.034813304 |
| --- | --- | --- | --- |
| blue | ROPN1L | MXD1 | 0.025017851 |
| blue | ROPN1L | MSRB1 | 0.02621813 |
| blue | ROPN1L | MMP25 | 0.026775772 |
| blue | ROPN1L | MGAM2 | 0.027524004 |
| blue | ROPN1L | MGAM | 0.039774704 |
| blue | ROPN1L | MAPK1 | 0.027147161 |
| blue | ROPN1L | MANSC1 | 0.034853795 |
| blue | ROPN1L | LRG1 | 0.028090691 |
| blue | ROPN1L | LMNB1 | 0.030241905 |
| blue | ROPN1L | KLHL2 | 0.027748941 |
| blue | ROPN1L | ITPRIP | 0.025414135 |
| blue | ROPN1L | HIST2H2AC | 0.024882273 |
| blue | ROPN1L | GCA | 0.030871778 |
| blue | ROPN1L | GAB2 | 0.02875285 |
| blue | ROPN1L | FLOT2 | 0.035031876 |
| blue | ROPN1L | CYSTM1 | 0.032823579 |
| blue | ROPN1L | CSF3R | 0.032324589 |
| blue | ROPN1L | CEBPB | 0.038848882 |
| blue | ROPN1L | CEACAM3 | 0.028143701 |
| blue | ROPN1L | CA4 | 0.029487395 |
| blue | ROPN1L | BCL6 | 0.063742381 |
| blue | ROPN1L | B4GALT5 | 0.021044256 |
| blue | ROPN1L | AQP9 | 0.07397346 |
| blue | ROPN1L | ANXA3 | 0.024104628 |
| blue | ROPN1L | ALOX5 | 0.033804181 |
| blue | ROPN1L | ACSL1 | 0.057046054 |
| blue | RNF24 | RNF19B | 0.021804746 |
| blue | RNF24 | RNF149 | 0.033976683 |
| blue | RNF24 | RNF130 | 0.020855756 |
| blue | RNF24 | RNASEL | 0.020825551 |
| blue | RNF24 | RGS2 | 0.035121735 |
| blue | RNF24 | RBM47 | 0.027911651 |
| blue | RNF24 | RASSF2 | 0.025965159 |
| blue | RNF24 | RALB | 0.022278314 |
| blue | RNF24 | RAF1 | 0.024886881 |
| blue | RNF24 | PYGL | 0.044202481 |
| blue | RNF24 | PTPRE | 0.03435457 |
| blue | RNF24 | PPP4R1 | 0.026310443 |
| blue | RNF24 | POLR2H | 0.024701635 |
| blue | RNF24 | PLXDC2 | 0.02025696 |
| blue | RNF24 | PLPPR2 | 0.031610394 |
| blue | RNF24 | PLEKHO2 | 0.034167404 |
| blue | RNF24 | PLBD1 | 0.020523977 |
| blue | RNF24 | PICALM | 0.023222251 |
| blue | RNF24 | PHF21A | 0.02824562 |
| blue | RNF24 | PGD | 0.02386283 |
| blue | RNF24 | PELI2 | 0.029295926 |
| blue | RNF24 | PECAM1 | 0.020984704 |
| blue | RNF24 | PAK1 | 0.025122526 |
| blue | RNF24 | OSBPL11 | 0.024243905 |
| blue | RNF24 | NUP214 | 0.023465274 |

| blue | RNF24 | NUMB | 0.021030381 |
| --- | --- | --- | --- |
| blue | RNF24 | NT5C2 | 0.024559797 |
| blue | RNF24 | NSMCE4A | 0.026642665 |
| blue | RNF24 | NRBF2 | 0.020465538 |
| blue | RNF24 | NLRP12 | 0.045192148 |
| blue | RNF24 | NIBAN1 | 0.030018297 |
| blue | RNF24 | NDEL1 | 0.024177677 |
| blue | RNF24 | NCF2 | 0.025856389 |
| blue | RNF24 | NCF1C | 0.025564918 |
| blue | RNF24 | NADK | 0.032373599 |
| blue | RNF24 | MYO1F | 0.028248017 |
| blue | RNF24 | MYD88 | 0.020284179 |
| blue | RNF24 | MXD1 | 0.042685069 |
| blue | RNF24 | MSRB1 | 0.027189811 |
| blue | RNF24 | MSL1 | 0.037324071 |
| blue | RNF24 | MME | 0.026980837 |
| blue | RNF24 | MGAM | 0.025094407 |
| blue | RNF24 | MEGF9 | 0.025109274 |
| blue | RNF24 | MEFV | 0.021245781 |
| blue | RNF24 | MBOAT7 | 0.038878289 |
| blue | RNF24 | MAST3 | 0.022727956 |
| blue | RNF24 | MAP4K1 | 0.02027571 |
| blue | RNF24 | MAP3K5 | 0.02356312 |
| blue | RNF24 | MAP3K11 | 0.020288586 |
| blue | RNF24 | MAP2K4 | 0.020263484 |
| blue | RNF24 | LYST | 0.021081724 |
| blue | RNF24 | LYN | 0.038646841 |
| blue | RNF24 | LRRK2 | 0.027175821 |
| blue | RNF24 | LRMP | 0.023451867 |
| blue | RNF24 | LITAF | 0.034609954 |
| blue | RNF24 | LCP1 | 0.023086849 |
| blue | RNF24 | LAT2 | 0.03386055 |
| blue | RNF24 | KIF1B | 0.027051163 |
| blue | RNF24 | KDM6B | 0.020054829 |
| blue | RNF24 | KCNJ15 | 0.02520547 |
| blue | RNF24 | JUNB | 0.030180411 |
| blue | RNF24 | ITM2B | 0.022014933 |
| blue | RNF24 | ITGAM | 0.025784557 |
| blue | RNF24 | IQSEC1 | 0.028064129 |
| blue | RNF24 | IL1RN | 0.023711115 |
| blue | RNF24 | IL17RA | 0.030869485 |
| blue | RNF24 | IFNGR2 | 0.020138887 |
| blue | RNF24 | ICAM3 | 0.021669302 |
| blue | RNF24 | HLX | 0.024156538 |
| blue | RNF24 | HCK | 0.038121984 |
| blue | RNF24 | GCA | 0.0275087 |
| blue | RNF24 | GAB2 | 0.0346522 |
| blue | RNF24 | FPR1 | 0.025521304 |
| blue | RNF24 | FNDC3B | 0.032976792 |
| blue | RNF24 | FCGR2A | 0.021491874 |
| blue | RNF24 | FBXL5 | 0.023288635 |
| blue | RNF24 | FAM8A1 | 0.021542208 |

| blue | RNF24 | EGLN1 | 0.023042107 |
| --- | --- | --- | --- |
| blue | RNF24 | DYSF | 0.038979181 |
| blue | RNF24 | DENND5A | 0.029510545 |
| blue | RNF24 | CYB5R4 | 0.022740408 |
| blue | RNF24 | CUX1 | 0.028192111 |
| blue | RNF24 | CTBS | 0.021329428 |
| blue | RNF24 | CPSF4 | 0.020559186 |
| blue | RNF24 | CMTM6 | 0.020001278 |
| blue | RNF24 | CHST15 | 0.020510334 |
| blue | RNF24 | CCNJL | 0.022500167 |
| blue | RNF24 | CANT1 | 0.022159263 |
| blue | RNF24 | C5AR1 | 0.031141863 |
| blue | RNF24 | C15orf39 | 0.032220991 |
| blue | RNF24 | BRI3 | 0.023848391 |
| blue | RNF24 | BCL6 | 0.026646908 |
| blue | RNF24 | BCL3 | 0.05328267 |
| blue | RNF24 | BAZ2B | 0.023335879 |
| blue | RNF24 | BASP1 | 0.052780158 |
| blue | RNF24 | ATP6V1B2 | 0.022361756 |
| blue | RNF24 | ARID3A | 0.030407001 |
| blue | RNF24 | ARAP1 | 0.031264347 |
| blue | RNF24 | AQP9 | 0.021459049 |
| blue | RNF24 | APMAP | 0.020964889 |
| blue | RNF24 | APBB1IP | 0.021891967 |
| blue | RNF24 | ANPEP | 0.02561138 |
| blue | RNF24 | ALPK1 | 0.021097781 |
| blue | RNF24 | AKIRIN2 | 0.036054442 |
| blue | RNF24 | AGTPBP1 | 0.023562428 |
| blue | RNF24 | ADGRE3 | 0.021738037 |
| blue | RNF24 | ADAM8 | 0.026357931 |
| blue | RNF24 | ACSL4 | 0.025082158 |
| blue | RNF24 | ACSL1 | 0.02628875 |
| blue | RNF24 | ACOX1 | 0.020612621 |
| blue | RNF216P1 | RGS2 | 0.021974949 |
| blue | RNF216P1 | PKN2 | 0.021800572 |
| blue | RNF216P1 | NADK | 0.023411347 |
| blue | RNF216P1 | MXD1 | 0.022388183 |
| blue | RNF216P1 | MSL1 | 0.022617101 |
| blue | RNF216P1 | MBOAT7 | 0.022655806 |
| blue | RNF216P1 | ICAM3 | 0.021136043 |
| blue | RNF216P1 | CANT1 | 0.024774024 |
| blue | RNF216P1 | C5AR1 | 0.021895443 |
| blue | RNF216P1 | ADAM8 | 0.029974347 |
| blue | RNF19B | RGS2 | 0.022671613 |
| blue | RNF19B | PKN2 | 0.024279021 |
| blue | RNF19B | NADK | 0.022074643 |
| blue | RNF19B | MXD1 | 0.022228136 |
| blue | RNF19B | MSL1 | 0.025980938 |
| blue | RNF19B | LAT2 | 0.020977943 |
| blue | RNF19B | CUX1 | 0.020890246 |
| blue | RNF19B | C5AR1 | 0.025220566 |
| blue | RNF19B | BAZ2B | 0.02808567 |

| blue | RNF19B | ADAM8 | 0.023837833 |
| --- | --- | --- | --- |
| blue | RNF149 | RNASEL | 0.021921206 |
| blue | RNF149 | RHOG | 0.021761369 |
| blue | RNF149 | RGS2 | 0.021736245 |
| blue | RNF149 | RBM47 | 0.036970571 |
| blue | RNF149 | RALB | 0.046605588 |
| blue | RNF149 | RAF1 | 0.024775651 |
| blue | RNF149 | QPCT | 0.025264248 |
| blue | RNF149 | PYGL | 0.025340587 |
| blue | RNF149 | PTPRE | 0.021426427 |
| blue | RNF149 | PTEN | 0.023317177 |
| blue | RNF149 | PRR13 | 0.029078659 |
| blue | RNF149 | PLXNC1 | 0.026338324 |
| blue | RNF149 | PLXDC2 | 0.022300477 |
| blue | RNF149 | PILRA | 0.021578455 |
| blue | RNF149 | PHF21A | 0.031055052 |
| blue | RNF149 | PFKFB3 | 0.02190665 |
| blue | RNF149 | PELI2 | 0.023518188 |
| blue | RNF149 | PANX2 | 0.020774727 |
| blue | RNF149 | OSBPL2 | 0.021115936 |
| blue | RNF149 | OSBPL11 | 0.023785432 |
| blue | RNF149 | NUMB | 0.041743458 |
| blue | RNF149 | NT5C2 | 0.020314356 |
| blue | RNF149 | NRBF2 | 0.035368004 |
| blue | RNF149 | NLRP12 | 0.021027199 |
| blue | RNF149 | NIBAN1 | 0.036746849 |
| blue | RNF149 | NFIL3 | 0.034201185 |
| blue | RNF149 | NDEL1 | 0.046116768 |
| blue | RNF149 | NCF2 | 0.033202438 |
| blue | RNF149 | NAMPT | 0.041930729 |
| blue | RNF149 | NADK | 0.021807384 |
| blue | RNF149 | MXD1 | 0.046666732 |
| blue | RNF149 | MSRB1 | 0.044171885 |
| blue | RNF149 | MSL1 | 0.025162145 |
| blue | RNF149 | MNDA | 0.022170048 |
| blue | RNF149 | MMP25 | 0.02091496 |
| blue | RNF149 | MME | 0.022427589 |
| blue | RNF149 | MGAM2 | 0.020338885 |
| blue | RNF149 | MGAM | 0.024282969 |
| blue | RNF149 | MEGF9 | 0.022128566 |
| blue | RNF149 | MCL1 | 0.021563287 |
| blue | RNF149 | MBOAT7 | 0.024680143 |
| blue | RNF149 | LYN | 0.043219935 |
| blue | RNF149 | LY96 | 0.025819293 |
| blue | RNF149 | LRRK2 | 0.025479456 |
| blue | RNF149 | LRP10 | 0.022691976 |
| blue | RNF149 | LRMP | 0.021278974 |
| blue | RNF149 | LRG1 | 0.025190102 |
| blue | RNF149 | LMNB1 | 0.023456678 |
| blue | RNF149 | LITAF | 0.041829904 |
| blue | RNF149 | LCP1 | 0.026264378 |
| blue | RNF149 | KPNB1 | 0.021750631 |

| blue | RNF149 | KLHL2 | 0.022184958 |
| --- | --- | --- | --- |
| blue | RNF149 | KCNJ15 | 0.031220344 |
| blue | RNF149 | ITPRIP | 0.032463611 |
| blue | RNF149 | ITM2B | 0.020631307 |
| blue | RNF149 | IL1RN | 0.023769799 |
| blue | RNF149 | IGF2R | 0.023198995 |
| blue | RNF149 | IFNGR2 | 0.033494215 |
| blue | RNF149 | IFNGR1 | 0.020759239 |
| blue | RNF149 | IFITM2 | 0.03364267 |
| blue | RNF149 | HSPA1A | 0.024435606 |
| blue | RNF149 | HLX | 0.023621914 |
| blue | RNF149 | HCK | 0.033709722 |
| blue | RNF149 | GK | 0.020809844 |
| blue | RNF149 | GCA | 0.046570607 |
| blue | RNF149 | GAB2 | 0.045582174 |
| blue | RNF149 | FRAT2 | 0.024688246 |
| blue | RNF149 | FPR1 | 0.043761743 |
| blue | RNF149 | FNDC3B | 0.022999768 |
| blue | RNF149 | FLOT2 | 0.035093094 |
| blue | RNF149 | FCGR2A | 0.033861186 |
| blue | RNF149 | FBXL5 | 0.024316902 |
| blue | RNF149 | FAM8A1 | 0.035619477 |
| blue | RNF149 | FAM160B1 | 0.020452284 |
| blue | RNF149 | EXOC6 | 0.029395036 |
| blue | RNF149 | EVI2B | 0.021408297 |
| blue | RNF149 | ETS2 | 0.029798151 |
| blue | RNF149 | EGLN1 | 0.024783542 |
| blue | RNF149 | DYSF | 0.027087877 |
| blue | RNF149 | DENND5A | 0.024102226 |
| blue | RNF149 | CYB5R4 | 0.0284642 |
| blue | RNF149 | CXCR2 | 0.0222789 |
| blue | RNF149 | CXCR1 | 0.029201838 |
| blue | RNF149 | CTBS | 0.020625954 |
| blue | RNF149 | CSF3R | 0.04266589 |
| blue | RNF149 | CPD | 0.023052948 |
| blue | RNF149 | COP1 | 0.026018505 |
| blue | RNF149 | CMTM6 | 0.024785321 |
| blue | RNF149 | CMTM2 | 0.026918273 |
| blue | RNF149 | CHST15 | 0.024438521 |
| blue | RNF149 | CEBPB | 0.034139403 |
| blue | RNF149 | C15orf39 | 0.021035948 |
| blue | RNF149 | BEST1 | 0.025995363 |
| blue | RNF149 | BCL6 | 0.040580705 |
| blue | RNF149 | BCL3 | 0.045720339 |
| blue | RNF149 | BCL2A1 | 0.020308655 |
| blue | RNF149 | BASP1 | 0.040159063 |
| blue | RNF149 | B4GALT5 | 0.026090576 |
| blue | RNF149 | ATP6V1B2 | 0.03135753 |
| blue | RNF149 | ATP6V0B | 0.023078524 |
| blue | RNF149 | AQP9 | 0.056290435 |
| blue | RNF149 | ADGRE5 | 0.027673617 |
| blue | RNF149 | ACSL1 | 0.037005347 |

| blue | RNF149 | ACOX1 | 0.032019956 |
| --- | --- | --- | --- |
| blue | RNF130 | RHOG | 0.023011619 |
| blue | RNF130 | RGS2 | 0.026997485 |
| blue | RNF130 | RBP7 | 0.021760559 |
| blue | RNF130 | NADK | 0.020084569 |
| blue | RNF130 | MSRB1 | 0.026198249 |
| blue | RNF130 | MBOAT7 | 0.021747418 |
| blue | RNF130 | LYN | 0.02429499 |
| blue | RNF130 | ITPR3 | 0.020512741 |
| blue | RNF130 | HLX | 0.020501975 |
| blue | RNF130 | GCA | 0.024367769 |
| blue | RNF130 | GAB2 | 0.024326799 |
| blue | RNF130 | C5AR1 | 0.023747534 |
| blue | RNF130 | ATP6V1B2 | 0.020238564 |
| blue | RNF13 | LYN | 0.024294206 |
| blue | RNF13 | CYB5R4 | 0.026111778 |
| blue | RNASEL | RALB | 0.020581895 |
| blue | RNASEL | RAF1 | 0.020087777 |
| blue | RNASEL | NDEL1 | 0.022193942 |
| blue | RNASEL | MXD1 | 0.034419708 |
| blue | RNASEL | LYN | 0.021282025 |
| blue | RNASEL | LRRK2 | 0.02399122 |
| blue | RNASEL | GCA | 0.021521872 |
| blue | RNASEL | GAB2 | 0.023887966 |
| blue | RIPOR2 | IL1R2 | 0.034916597 |
| blue | RIPOR2 | IFNGR1 | 0.021521273 |
| blue | RHOG | MSRB1 | 0.055191447 |
| blue | RHOG | LYN | 0.024207984 |
| blue | RHOG | KIAA0513 | 0.020720076 |
| blue | RHOG | GCA | 0.022061568 |
| blue | RHOG | GAB2 | 0.029182314 |
| blue | RHOG | DYSF | 0.020255463 |
| blue | RHOG | CFP | 0.03273877 |
| blue | RHOG | BASP1 | 0.022312529 |
| blue | RHOG | ATP6V1B2 | 0.037257008 |
| blue | RHOG | AQP9 | 0.022403575 |
| blue | RGS2 | RBP7 | 0.028178692 |
| blue | RGS2 | RBM47 | 0.021088264 |
| blue | RGS2 | RASSF2 | 0.023026709 |
| blue | RGS2 | RAF1 | 0.022760793 |
| blue | RGS2 | RAB5C | 0.020614688 |
| blue | RGS2 | PYGL | 0.027540341 |
| blue | RGS2 | PTPRE | 0.023644306 |
| blue | RGS2 | PPP4R1 | 0.029832252 |
| blue | RGS2 | PPP1R12A | 0.021434771 |
| blue | RGS2 | POLR2H | 0.028977425 |
| blue | RGS2 | PLXDC2 | 0.024539048 |
| blue | RGS2 | PLPPR2 | 0.030789079 |
| blue | RGS2 | PLEKHO2 | 0.028759315 |
| blue | RGS2 | PKN2 | 0.020818815 |
| blue | RGS2 | PICALM | 0.021705593 |
| blue | RGS2 | PELI2 | 0.027392913 |

| blue | RGS2 | PECAM1 | 0.027351096 |
| --- | --- | --- | --- |
| blue | RGS2 | PAK1 | 0.023480257 |
| blue | RGS2 | OSBPL11 | 0.022647712 |
| blue | RGS2 | NUP214 | 0.024092484 |
| blue | RGS2 | NSMCE4A | 0.02447054 |
| blue | RGS2 | NOP2 | 0.031652812 |
| blue | RGS2 | NLRP12 | 0.036857563 |
| blue | RGS2 | NDEL1 | 0.024978653 |
| blue | RGS2 | NCF1C | 0.029134765 |
| blue | RGS2 | NADK | 0.025064487 |
| blue | RGS2 | MYO1F | 0.027003687 |
| blue | RGS2 | MXD1 | 0.035111505 |
| blue | RGS2 | MSRB1 | 0.02266398 |
| blue | RGS2 | MSL1 | 0.026064658 |
| blue | RGS2 | MME | 0.032719783 |
| blue | RGS2 | MEGF9 | 0.024949064 |
| blue | RGS2 | MEFV | 0.022644107 |
| blue | RGS2 | MBOAT7 | 0.032114225 |
| blue | RGS2 | MAST3 | 0.022811661 |
| blue | RGS2 | MAP3K5 | 0.032208709 |
| blue | RGS2 | MAP2K4 | 0.034364713 |
| blue | RGS2 | LYN | 0.035081325 |
| blue | RGS2 | LITAF | 0.047889637 |
| blue | RGS2 | LCP1 | 0.037007824 |
| blue | RGS2 | LAT2 | 0.03064537 |
| blue | RGS2 | KIF1B | 0.027754842 |
| blue | RGS2 | ITPK1 | 0.021008877 |
| blue | RGS2 | ITM2B | 0.040732896 |
| blue | RGS2 | ITGAM | 0.026687764 |
| blue | RGS2 | IQSEC1 | 0.021878068 |
| blue | RGS2 | IQGAP1 | 0.029802195 |
| blue | RGS2 | IFRD1 | 0.030782619 |
| blue | RGS2 | ICAM3 | 0.039342759 |
| blue | RGS2 | HSDL2 | 0.044382792 |
| blue | RGS2 | HCK | 0.021251946 |
| blue | RGS2 | HBP1 | 0.021048683 |
| blue | RGS2 | GNB2 | 0.024826071 |
| blue | RGS2 | GCA | 0.024433748 |
| blue | RGS2 | GAB2 | 0.029247974 |
| blue | RGS2 | FOS | 0.020050991 |
| blue | RGS2 | FNDC3B | 0.023417146 |
| blue | RGS2 | FAM49B | 0.022599044 |
| blue | RGS2 | DYSF | 0.027421878 |
| blue | RGS2 | CYB5R4 | 0.028017395 |
| blue | RGS2 | CUX1 | 0.025206684 |
| blue | RGS2 | CPSF4 | 0.020871836 |
| blue | RGS2 | CPPED1 | 0.024044899 |
| blue | RGS2 | CMTM6 | 0.020435511 |
| blue | RGS2 | CMTM2 | 0.021807857 |
| blue | RGS2 | CD58 | 0.02052171 |
| blue | RGS2 | CBL | 0.0246783 |
| blue | RGS2 | CAPZA2 | 0.024296464 |

| blue | RGS2 | CAP1 | 0.026604898 |
| --- | --- | --- | --- |
| blue | RGS2 | CANT1 | 0.027062724 |
| blue | RGS2 | C5AR1 | 0.0506754 |
| blue | RGS2 | C16orf58 | 0.025188458 |
| blue | RGS2 | BRI3 | 0.028685108 |
| blue | RGS2 | BCL3 | 0.03077273 |
| blue | RGS2 | BAZ2B | 0.02007451 |
| blue | RGS2 | BASP1 | 0.043463144 |
| blue | RGS2 | ATP6V1B2 | 0.021179166 |
| blue | RGS2 | ARID3A | 0.02937251 |
| blue | RGS2 | APMAP | 0.025388707 |
| blue | RGS2 | APBB1IP | 0.029282376 |
| blue | RGS2 | ANPEP | 0.024241787 |
| blue | RGS2 | AKIRIN2 | 0.027835401 |
| blue | RGS2 | ADGRE3 | 0.027055524 |
| blue | RGS2 | ACSL4 | 0.021297988 |
| blue | RGL4 | CEBPB | 0.020006305 |
| blue | RGL4 | BCL6 | 0.024005743 |
| blue | RGL4 | AQP9 | 0.028623292 |
| blue | RGL4 | ACSL1 | 0.021448777 |
| blue | RFX1 | MSL1 | 0.024867561 |
| blue | RESF1 | PDPK1 | 0.021213335 |
| blue | RESF1 | MXD1 | 0.028046471 |
| blue | REPS2 | RALB | 0.032497919 |
| blue | REPS2 | PFKFB4 | 0.022703439 |
| blue | REPS2 | PACSIN2 | 0.030929379 |
| blue | REPS2 | OSBPL2 | 0.030928348 |
| blue | REPS2 | NUMB | 0.020621908 |
| blue | REPS2 | NFIL3 | 0.02068208 |
| blue | REPS2 | NDEL1 | 0.026605595 |
| blue | REPS2 | NCOA1 | 0.024459847 |
| blue | REPS2 | MXD1 | 0.023261746 |
| blue | REPS2 | MMP25 | 0.023008994 |
| blue | REPS2 | MANSC1 | 0.030009541 |
| blue | REPS2 | LRG1 | 0.026378794 |
| blue | REPS2 | KLHL2 | 0.023307487 |
| blue | REPS2 | ITPRIP | 0.026261815 |
| blue | REPS2 | IL1R2 | 0.043125065 |
| blue | REPS2 | IGF2R | 0.021619655 |
| blue | REPS2 | IFNGR1 | 0.028306099 |
| blue | REPS2 | HAL | 0.028004832 |
| blue | REPS2 | GAB2 | 0.025875371 |
| blue | REPS2 | FAM49A | 0.03138447 |
| blue | REPS2 | EVI2B | 0.024622721 |
| blue | REPS2 | ETS2 | 0.020097407 |
| blue | REPS2 | CXCR1 | 0.025491637 |
| blue | REPS2 | CSF3R | 0.036842528 |
| blue | REPS2 | CELF2 | 0.027046946 |
| blue | REPS2 | CEBPB | 0.026533965 |
| blue | REPS2 | AQP9 | 0.03082679 |
| blue | REPS2 | ANKS1A | 0.029863226 |
| blue | REPS2 | ABTB1 | 0.029388864 |

| blue | RBPJ | RALB | 0.028689272 |
| --- | --- | --- | --- |
| blue | RBPJ | MSRB1 | 0.020482732 |
| blue | RBPJ | LYN | 0.021846965 |
| blue | RBPJ | GCA | 0.023111103 |
| blue | RBPJ | AQP9 | 0.021623976 |
| blue | RBP7 | LAT2 | 0.023472929 |
| blue | RBP7 | C5AR1 | 0.020079232 |
| blue | RBP7 | BRI3 | 0.032604332 |
| blue | RBM47 | RALB | 0.060446274 |
| blue | RBM47 | RAF1 | 0.020572196 |
| blue | RBM47 | QPCT | 0.023436225 |
| blue | RBM47 | PYGL | 0.035664118 |
| blue | RBM47 | PROK2 | 0.02145267 |
| blue | RBM47 | PLXDC2 | 0.021599571 |
| blue | RBM47 | PHF21A | 0.020961707 |
| blue | RBM47 | NUMB | 0.049227638 |
| blue | RBM47 | NRBF2 | 0.037849809 |
| blue | RBM47 | NFIL3 | 0.044306136 |
| blue | RBM47 | NDEL1 | 0.037749614 |
| blue | RBM47 | NCF2 | 0.026552967 |
| blue | RBM47 | NAMPT | 0.033637854 |
| blue | RBM47 | MXD1 | 0.033836614 |
| blue | RBM47 | MSRB1 | 0.04790582 |
| blue | RBM47 | MME | 0.021601329 |
| blue | RBM47 | MGAM | 0.02451414 |
| blue | RBM47 | MEGF9 | 0.02173802 |
| blue | RBM47 | LYN | 0.027881025 |
| blue | RBM47 | KLHL2 | 0.02188243 |
| blue | RBM47 | ITPRIP | 0.021663956 |
| blue | RBM47 | IFNGR2 | 0.026612304 |
| blue | RBM47 | GCA | 0.044135367 |
| blue | RBM47 | GAB2 | 0.039822042 |
| blue | RBM47 | FRAT2 | 0.034210506 |
| blue | RBM47 | FPR1 | 0.022270347 |
| blue | RBM47 | FBXL5 | 0.023580256 |
| blue | RBM47 | EGLN1 | 0.024982867 |
| blue | RBM47 | DYSF | 0.022035886 |
| blue | RBM47 | CYB5R4 | 0.022581169 |
| blue | RBM47 | CSF3R | 0.029016286 |
| blue | RBM47 | CMTM2 | 0.024304735 |
| blue | RBM47 | CEBPB | 0.032336555 |
| blue | RBM47 | BCL6 | 0.05024693 |
| blue | RBM47 | BASP1 | 0.036437575 |
| blue | RBM47 | ATP6V1B2 | 0.022548946 |
| blue | RBM47 | AQP9 | 0.060772685 |
| blue | RBM47 | ACSL1 | 0.045189833 |
| blue | RASSF2 | PILRA | 0.020313569 |
| blue | RASSF2 | PICALM | 0.021505952 |
| blue | RASSF2 | PELI2 | 0.029628723 |
| blue | RASSF2 | PAK1 | 0.020327936 |
| blue | RASSF2 | NADK | 0.028475635 |
| blue | RASSF2 | MXD1 | 0.034778888 |

| blue | RASSF2 | MSL1 | 0.031131579 |
| --- | --- | --- | --- |
| blue | RASSF2 | MBOAT7 | 0.02285459 |
| blue | RASSF2 | MAP3K5 | 0.024690473 |
| blue | RASSF2 | LAT2 | 0.024674103 |
| blue | RASSF2 | IQGAP1 | 0.020015019 |
| blue | RASSF2 | GAB2 | 0.034317675 |
| blue | RASSF2 | ERGIC1 | 0.025956588 |
| blue | RASSF2 | DYSF | 0.021380774 |
| blue | RASSF2 | DCP2 | 0.029830325 |
| blue | RASSF2 | CHST15 | 0.022319197 |
| blue | RASSF2 | ARID3A | 0.035582085 |
| blue | RASSF2 | ARAP1 | 0.026845124 |
| blue | RALB | RAF1 | 0.031233048 |
| blue | RALB | RAB31 | 0.025862583 |
| blue | RALB | QPCT | 0.045154616 |
| blue | RALB | PYGL | 0.02741959 |
| blue | RALB | PTEN | 0.024584116 |
| blue | RALB | PRR13 | 0.024566588 |
| blue | RALB | PRKCD | 0.022241314 |
| blue | RALB | POLR2H | 0.022106447 |
| blue | RALB | PLXNC1 | 0.045553076 |
| blue | RALB | PLXDC2 | 0.025207927 |
| blue | RALB | PLBD1 | 0.024991067 |
| blue | RALB | PILRA | 0.026150128 |
| blue | RALB | PHF21A | 0.036992602 |
| blue | RALB | PHC2 | 0.021554915 |
| blue | RALB | PFKFB4 | 0.027927313 |
| blue | RALB | PFKFB3 | 0.029754708 |
| blue | RALB | PELI2 | 0.02356206 |
| blue | RALB | PANX2 | 0.036123794 |
| blue | RALB | PACSIN2 | 0.022675425 |
| blue | RALB | OSGIN2 | 0.023014018 |
| blue | RALB | OSBPL2 | 0.030034662 |
| blue | RALB | OSBPL11 | 0.029682835 |
| blue | RALB | NUMB | 0.061061428 |
| blue | RALB | NT5C2 | 0.024211846 |
| blue | RALB | NRBF2 | 0.066544416 |
| blue | RALB | NIBAN1 | 0.037857527 |
| blue | RALB | NFIL3 | 0.065022647 |
| blue | RALB | NDEL1 | 0.042992828 |
| blue | RALB | NCOA1 | 0.029274568 |
| blue | RALB | NCF4 | 0.030011477 |
| blue | RALB | NCF2 | 0.042629622 |
| blue | RALB | NAMPT | 0.060708011 |
| blue | RALB | MXD1 | 0.03756086 |
| blue | RALB | MTMR10 | 0.020581079 |
| blue | RALB | MSRB1 | 0.04261767 |
| blue | RALB | MNDA | 0.031151451 |
| blue | RALB | MMP25 | 0.031576755 |
| blue | RALB | MME | 0.029744046 |
| blue | RALB | MIR223 | 0.025411976 |
| blue | RALB | MGAM2 | 0.030017373 |

| blue | RALB | MGAM | 0.033937944 |
| --- | --- | --- | --- |
| blue | RALB | MEGF9 | 0.042272857 |
| blue | RALB | MCL1 | 0.020585436 |
| blue | RALB | MAT2A | 0.022008061 |
| blue | RALB | 1-Mar | 0.023576375 |
| blue | RALB | MAPK1 | 0.021004353 |
| blue | RALB | MAP4K1 | 0.024963275 |
| blue | RALB | MANSC1 | 0.040957467 |
| blue | RALB | MAEA | 0.021337111 |
| blue | RALB | LYN | 0.034909756 |
| blue | RALB | LY96 | 0.030985023 |
| blue | RALB | LRRK2 | 0.0318163 |
| blue | RALB | LRP10 | 0.0260265 |
| blue | RALB | LRMP | 0.022148015 |
| blue | RALB | LRG1 | 0.040362603 |
| blue | RALB | LMNB1 | 0.038189247 |
| blue | RALB | LITAF | 0.031245705 |
| blue | RALB | KLHL2 | 0.043393771 |
| blue | RALB | KCNJ2 | 0.020153699 |
| blue | RALB | KCNJ15 | 0.034214573 |
| blue | RALB | KBTBD7 | 0.021604289 |
| blue | RALB | ITPRIP | 0.041877854 |
| blue | RALB | ITM2B | 0.021330361 |
| blue | RALB | IRS2 | 0.020624361 |
| blue | RALB | IL6R | 0.023709274 |
| blue | RALB | IL1R2 | 0.021225367 |
| blue | RALB | IGF2R | 0.033441948 |
| blue | RALB | IFRD1 | 0.025868932 |
| blue | RALB | IFNGR2 | 0.033051336 |
| blue | RALB | IFNGR1 | 0.048126249 |
| blue | RALB | IFITM2 | 0.030607309 |
| blue | RALB | HSPA1A | 0.030262083 |
| blue | RALB | HSD17B11 | 0.026710273 |
| blue | RALB | HLX | 0.025348701 |
| blue | RALB | HIST2H2AC | 0.027531931 |
| blue | RALB | HIST1H2BE | 0.026089979 |
| blue | RALB | HIST1H2BC | 0.033658353 |
| blue | RALB | HCK | 0.029873303 |
| blue | RALB | HAL | 0.030649314 |
| blue | RALB | GK | 0.031203033 |
| blue | RALB | GCA | 0.053406558 |
| blue | RALB | GAB2 | 0.040874798 |
| blue | RALB | FRAT2 | 0.036305641 |
| blue | RALB | FRAT1 | 0.024127018 |
| blue | RALB | FPR2 | 0.027064158 |
| blue | RALB | FPR1 | 0.0423515 |
| blue | RALB | FOS | 0.032973619 |
| blue | RALB | FNIP1 | 0.021524888 |
| blue | RALB | FNDC3B | 0.024351097 |
| blue | RALB | FLOT2 | 0.046636669 |
| blue | RALB | FGR | 0.020484066 |
| blue | RALB | FCGR2A | 0.04444369 |

| blue | RALB | FBXL5 | 0.037606402 |
| --- | --- | --- | --- |
| blue | RALB | FAM8A1 | 0.02309153 |
| blue | RALB | FAM49B | 0.026008543 |
| blue | RALB | FAM49A | 0.024098686 |
| blue | RALB | F5 | 0.020376574 |
| blue | RALB | EXOC6 | 0.036937774 |
| blue | RALB | EVI2B | 0.038023851 |
| blue | RALB | ETS2 | 0.036981509 |
| blue | RALB | EGLN1 | 0.04480245 |
| blue | RALB | DYSF | 0.021021095 |
| blue | RALB | DNTTIP1 | 0.023905683 |
| blue | RALB | CYSTM1 | 0.027130357 |
| blue | RALB | CYB5R4 | 0.048476813 |
| blue | RALB | CXCR2 | 0.030056069 |
| blue | RALB | CXCR1 | 0.023715091 |
| blue | RALB | CTBS | 0.025047361 |
| blue | RALB | CSF3R | 0.038640316 |
| blue | RALB | CREBBP | 0.025113837 |
| blue | RALB | CPSF4 | 0.021050256 |
| blue | RALB | CPD | 0.028031375 |
| blue | RALB | COP1 | 0.035044286 |
| blue | RALB | CNEP1R1 | 0.024947908 |
| blue | RALB | CMTM6 | 0.035259223 |
| blue | RALB | CMTM2 | 0.038030216 |
| blue | RALB | CHST15 | 0.029194294 |
| blue | RALB | CHMP2A | 0.021924025 |
| blue | RALB | CELF2 | 0.033494812 |
| blue | RALB | CEBPD | 0.022435988 |
| blue | RALB | CEBPB | 0.055083109 |
| blue | RALB | CEACAM3 | 0.026757671 |
| blue | RALB | CD58 | 0.021073743 |
| blue | RALB | CD46 | 0.02813738 |
| blue | RALB | CA4 | 0.023848774 |
| blue | RALB | C1RL | 0.026254799 |
| blue | RALB | BTBD10 | 0.021429635 |
| blue | RALB | BEST1 | 0.032586674 |
| blue | RALB | BCL6 | 0.053942019 |
| blue | RALB | BCL3 | 0.036759356 |
| blue | RALB | BCL2A1 | 0.040337115 |
| blue | RALB | BASP1 | 0.03494598 |
| blue | RALB | B4GALT5 | 0.045391708 |
| blue | RALB | ATP6V1B2 | 0.031136842 |
| blue | RALB | ATP6V0B | 0.030161625 |
| blue | RALB | ASPRV1 | 0.027323253 |
| blue | RALB | AQP9 | 0.063828201 |
| blue | RALB | ANXA3 | 0.026603811 |
| blue | RALB | ANKS1A | 0.020907557 |
| blue | RALB | ALOX5AP | 0.027291415 |
| blue | RALB | ALOX5 | 0.026197303 |
| blue | RALB | AGO4 | 0.020108785 |
| blue | RALB | ADGRE5 | 0.03249215 |
| blue | RALB | ADGRE3 | 0.022089717 |

| blue | RALB | ACSL4 | 0.027773076 |
| --- | --- | --- | --- |
| blue | RALB | ACSL1 | 0.049654437 |
| blue | RALB | ACOX1 | 0.051981353 |
| blue | RALB | ABTB1 | 0.020888168 |
| blue | RALB | ABHD5 | 0.028150056 |
| blue | RAF1 | PYGL | 0.021024512 |
| blue | RAF1 | PPP4R1 | 0.024255241 |
| blue | RAF1 | PLXNC1 | 0.020042949 |
| blue | RAF1 | PHF20L1 | 0.022044579 |
| blue | RAF1 | PELI2 | 0.025178797 |
| blue | RAF1 | PANX2 | 0.043513108 |
| blue | RAF1 | PAK1 | 0.020504354 |
| blue | RAF1 | PADI4 | 0.020231697 |
| blue | RAF1 | OSBPL2 | 0.038005286 |
| blue | RAF1 | OSBPL11 | 0.023416073 |
| blue | RAF1 | NUMB | 0.025697394 |
| blue | RAF1 | NT5C2 | 0.029979602 |
| blue | RAF1 | NRBF2 | 0.020634275 |
| blue | RAF1 | NLRP12 | 0.021657724 |
| blue | RAF1 | NFIL3 | 0.022676564 |
| blue | RAF1 | NEDD9 | 0.022461932 |
| blue | RAF1 | NDEL1 | 0.050013263 |
| blue | RAF1 | NCF2 | 0.020301982 |
| blue | RAF1 | NAMPT | 0.020184541 |
| blue | RAF1 | NADK | 0.020877776 |
| blue | RAF1 | MXD1 | 0.053309672 |
| blue | RAF1 | MTMR10 | 0.028560908 |
| blue | RAF1 | MSRB1 | 0.022323755 |
| blue | RAF1 | MSL1 | 0.020514555 |
| blue | RAF1 | MME | 0.029565847 |
| blue | RAF1 | MGAM2 | 0.021188173 |
| blue | RAF1 | MGAM | 0.025099715 |
| blue | RAF1 | MEGF9 | 0.024314274 |
| blue | RAF1 | MAST3 | 0.020613495 |
| blue | RAF1 | LRRK2 | 0.020336657 |
| blue | RAF1 | LRP10 | 0.040068558 |
| blue | RAF1 | LRMP | 0.022671807 |
| blue | RAF1 | LITAF | 0.022062267 |
| blue | RAF1 | KLHL2 | 0.02232972 |
| blue | RAF1 | KBTBD7 | 0.020874478 |
| blue | RAF1 | ITPRIP | 0.021056933 |
| blue | RAF1 | IGF2R | 0.02960722 |
| blue | RAF1 | IFRD1 | 0.03502057 |
| blue | RAF1 | ICAM3 | 0.020814829 |
| blue | RAF1 | HSDL2 | 0.024420965 |
| blue | RAF1 | HAL | 0.031406067 |
| blue | RAF1 | GCA | 0.025810247 |
| blue | RAF1 | GAB2 | 0.037339702 |
| blue | RAF1 | FPR1 | 0.021680076 |
| blue | RAF1 | FNIP1 | 0.021620518 |
| blue | RAF1 | FAM8A1 | 0.023389136 |
| blue | RAF1 | FAM49B | 0.022181562 |

| blue | RAF1 | FAM160B1 | 0.022792216 |
| --- | --- | --- | --- |
| blue | RAF1 | DYSF | 0.02451133 |
| blue | RAF1 | CYB5R4 | 0.026105684 |
| blue | RAF1 | CTBS | 0.021100381 |
| blue | RAF1 | CSF3R | 0.029248098 |
| blue | RAF1 | CPD | 0.020717069 |
| blue | RAF1 | COP1 | 0.021391814 |
| blue | RAF1 | CMTM2 | 0.028416958 |
| blue | RAF1 | CHST15 | 0.031063198 |
| blue | RAF1 | BEST1 | 0.026702534 |
| blue | RAF1 | BCL6 | 0.025124671 |
| blue | RAF1 | BASP1 | 0.021098577 |
| blue | RAF1 | ATP6V0B | 0.024102292 |
| blue | RAF1 | ARHGAP9 | 0.039919955 |
| blue | RAF1 | AQP9 | 0.029525356 |
| blue | RAF1 | ADGRE3 | 0.023423488 |
| blue | RAF1 | ACSL1 | 0.021011148 |
| blue | RAF1 | ACOX1 | 0.027980674 |
| blue | RAF1 | ABHD5 | 0.025984099 |
| blue | RAB5C | MSL1 | 0.02041181 |
| blue | RAB5C | CUX1 | 0.025062423 |
| blue | RAB5C | C5AR1 | 0.025093622 |
| blue | RAB5C | C16orf58 | 0.021666059 |
| blue | RAB33B | CUX1 | 0.022138068 |
| blue | RAB31 | NFIL3 | 0.024646888 |
| blue | RAB31 | MSRB1 | 0.031317048 |
| blue | RAB31 | GCA | 0.021355814 |
| blue | RAB31 | GAB2 | 0.026079178 |
| blue | RAB31 | ATP6V1B2 | 0.020163251 |
| blue | RAB31 | AQP9 | 0.033241204 |
| blue | RAB11FIP1 | PELI2 | 0.020012315 |
| blue | RAB11FIP1 | OSBPL2 | 0.023189857 |
| blue | RAB11FIP1 | NDEL1 | 0.028195312 |
| blue | RAB11FIP1 | MXD1 | 0.0262997 |
| blue | RAB11FIP1 | IGF2R | 0.025111426 |
| blue | RAB11FIP1 | GAB2 | 0.026307788 |
| blue | RAB11FIP1 | CSF3R | 0.026190249 |
| blue | RAB11FIP1 | CHST15 | 0.020728351 |
| blue | QPCT | PYGL | 0.030164274 |
| blue | QPCT | PROK2 | 0.024812455 |
| blue | QPCT | PFKFB3 | 0.021031738 |
| blue | QPCT | NUMB | 0.026974454 |
| blue | QPCT | NRBF2 | 0.032217221 |
| blue | QPCT | NFIL3 | 0.049900424 |
| blue | QPCT | NDEL1 | 0.023319016 |
| blue | QPCT | NCF2 | 0.025444284 |
| blue | QPCT | NAMPT | 0.046714063 |
| blue | QPCT | MSRB1 | 0.030248428 |
| blue | QPCT | MNDA | 0.025660528 |
| blue | QPCT | MMP9 | 0.022578929 |
| blue | QPCT | MGAM | 0.022653967 |
| blue | QPCT | MANSC1 | 0.020339663 |

| blue | QPCT | LMNB1 | 0.027038592 |
| --- | --- | --- | --- |
| blue | QPCT | KLHL2 | 0.025528637 |
| blue | QPCT | IFNGR2 | 0.021219389 |
| blue | QPCT | GCA | 0.041596673 |
| blue | QPCT | GAB2 | 0.022484731 |
| blue | QPCT | FRAT2 | 0.045736769 |
| blue | QPCT | FLOT2 | 0.02356951 |
| blue | QPCT | FBXL5 | 0.042303614 |
| blue | QPCT | EGLN1 | 0.02778686 |
| blue | QPCT | CYB5R4 | 0.021399347 |
| blue | QPCT | CHIC2 | 0.022338701 |
| blue | QPCT | CEBPB | 0.026722145 |
| blue | QPCT | CD58 | 0.022200843 |
| blue | QPCT | BCL6 | 0.043499332 |
| blue | QPCT | BASP1 | 0.028284007 |
| blue | QPCT | AQP9 | 0.049988964 |
| blue | QPCT | ACSL1 | 0.05464287 |
| blue | QPCT | ACOX1 | 0.020423146 |
| blue | PYGL | PTPRE | 0.031103911 |
| blue | PYGL | PRKCD | 0.020391409 |
| blue | PYGL | PLPPR2 | 0.021677489 |
| blue | PYGL | PLEKHO2 | 0.020019134 |
| blue | PYGL | PLBD1 | 0.02199944 |
| blue | PYGL | PICALM | 0.020230499 |
| blue | PYGL | PHF21A | 0.037499481 |
| blue | PYGL | PGD | 0.041002141 |
| blue | PYGL | PFKFB3 | 0.025458032 |
| blue | PYGL | PELI2 | 0.023317347 |
| blue | PYGL | OSBPL11 | 0.023064848 |
| blue | PYGL | NUP214 | 0.021248825 |
| blue | PYGL | NUMB | 0.020710813 |
| blue | PYGL | NT5C2 | 0.021202614 |
| blue | PYGL | NRBF2 | 0.022727274 |
| blue | PYGL | NLRP12 | 0.04391723 |
| blue | PYGL | NLRC4 | 0.024175275 |
| blue | PYGL | NIBAN1 | 0.027003245 |
| blue | PYGL | NFIL3 | 0.026135857 |
| blue | PYGL | NDEL1 | 0.023824242 |
| blue | PYGL | NCF2 | 0.028890822 |
| blue | PYGL | NCF1C | 0.022978923 |
| blue | PYGL | NAMPT | 0.02517299 |
| blue | PYGL | NADK | 0.028087666 |
| blue | PYGL | MYO1F | 0.023443102 |
| blue | PYGL | MYD88 | 0.021811322 |
| blue | PYGL | MXD1 | 0.043901423 |
| blue | PYGL | MSRB1 | 0.026485705 |
| blue | PYGL | MSL1 | 0.029054901 |
| blue | PYGL | MOSPD2 | 0.023680899 |
| blue | PYGL | MME | 0.033287996 |
| blue | PYGL | MGAM2 | 0.031080318 |
| blue | PYGL | MGAM | 0.037881012 |
| blue | PYGL | MEGF9 | 0.02228809 |

| blue | PYGL | MBOAT7 | 0.026151496 |
| --- | --- | --- | --- |
| blue | PYGL | LYST | 0.020057138 |
| blue | PYGL | LYN | 0.026523982 |
| blue | PYGL | LRRK2 | 0.025711344 |
| blue | PYGL | LRMP | 0.026161139 |
| blue | PYGL | LITAF | 0.024753198 |
| blue | PYGL | LAT2 | 0.026292685 |
| blue | PYGL | KIF1B | 0.023944729 |
| blue | PYGL | KCNJ15 | 0.027716225 |
| blue | PYGL | JUNB | 0.020448159 |
| blue | PYGL | ITGAM | 0.0293475 |
| blue | PYGL | IQSEC1 | 0.034937872 |
| blue | PYGL | IL17RA | 0.023463392 |
| blue | PYGL | IFNGR2 | 0.025826769 |
| blue | PYGL | HCK | 0.042489065 |
| blue | PYGL | GCA | 0.028906641 |
| blue | PYGL | GAB2 | 0.035168626 |
| blue | PYGL | FRAT2 | 0.032985363 |
| blue | PYGL | FRAT1 | 0.022171791 |
| blue | PYGL | FPR1 | 0.022311979 |
| blue | PYGL | FNDC3B | 0.021772086 |
| blue | PYGL | FLOT2 | 0.02198655 |
| blue | PYGL | FBXL5 | 0.036787442 |
| blue | PYGL | F5 | 0.027412041 |
| blue | PYGL | EXOC6 | 0.021646625 |
| blue | PYGL | ETS2 | 0.020218312 |
| blue | PYGL | EGLN1 | 0.022160411 |
| blue | PYGL | DYSF | 0.048473434 |
| blue | PYGL | DENND5A | 0.033616785 |
| blue | PYGL | CYB5R4 | 0.024808582 |
| blue | PYGL | CUX1 | 0.020193953 |
| blue | PYGL | CREB5 | 0.041156413 |
| blue | PYGL | CDA | 0.021448148 |
| blue | PYGL | BRI3 | 0.02556828 |
| blue | PYGL | BCL6 | 0.036019515 |
| blue | PYGL | BCL3 | 0.040022017 |
| blue | PYGL | BASP1 | 0.047869861 |
| blue | PYGL | ARID3A | 0.027849914 |
| blue | PYGL | ARAP1 | 0.024937249 |
| blue | PYGL | AQP9 | 0.028383232 |
| blue | PYGL | APMAP | 0.025119546 |
| blue | PYGL | APBB1IP | 0.022354911 |
| blue | PYGL | ANPEP | 0.026189392 |
| blue | PYGL | AKIRIN2 | 0.025245832 |
| blue | PYGL | AGTPBP1 | 0.025759623 |
| blue | PYGL | ADGRG3 | 0.021676069 |
| blue | PYGL | ADAM8 | 0.020121616 |
| blue | PYGL | ACSL4 | 0.025746599 |
| blue | PYGL | ACSL1 | 0.038669523 |
| blue | PXN | NADK | 0.029067936 |
| blue | PXN | MXD1 | 0.022276453 |
| blue | PXN | MSL1 | 0.020296089 |

| blue | PXN | MBOAT7 | 0.021394831 |
| --- | --- | --- | --- |
| blue | PXN | DYSF | 0.020366244 |
| blue | PXN | ARAP1 | 0.020835744 |
| blue | PTPRE | PELI2 | 0.023089396 |
| blue | PTPRE | PELI1 | 0.020020672 |
| blue | PTPRE | PAK1 | 0.020510933 |
| blue | PTPRE | NLRP12 | 0.025974605 |
| blue | PTPRE | NDEL1 | 0.02151776 |
| blue | PTPRE | NADK | 0.02504103 |
| blue | PTPRE | MXD1 | 0.041826547 |
| blue | PTPRE | MSRB1 | 0.020365511 |
| blue | PTPRE | MSL1 | 0.025370744 |
| blue | PTPRE | LYN | 0.021042599 |
| blue | PTPRE | LRRK2 | 0.027337486 |
| blue | PTPRE | GAB2 | 0.031655455 |
| blue | PTPRE | DYSF | 0.029714603 |
| blue | PTPRE | BASP1 | 0.022205094 |
| blue | PTEN | OSBPL2 | 0.020089073 |
| blue | PTEN | NUMB | 0.025099622 |
| blue | PTEN | NDEL1 | 0.030105904 |
| blue | PTEN | MXD1 | 0.040879858 |
| blue | PTEN | MSRB1 | 0.03217144 |
| blue | PTEN | MBOAT7 | 0.020300971 |
| blue | PTEN | LYN | 0.024020263 |
| blue | PTEN | GCA | 0.027055031 |
| blue | PTEN | GAB2 | 0.047914596 |
| blue | PTEN | EXOC6 | 0.023802569 |
| blue | PTEN | CSF3R | 0.025222378 |
| blue | PTEN | CHST15 | 0.020616939 |
| blue | PTEN | CEBPB | 0.031747799 |
| blue | PTEN | ATP6V1B2 | 0.025817737 |
| blue | PTEN | AQP9 | 0.027802265 |
| blue | PTAFR | PELI1 | 0.022108233 |
| blue | PTAFR | MXD1 | 0.022557886 |
| blue | PTAFR | MBOAT7 | 0.022758707 |
| blue | PTAFR | CXCR1 | 0.023353288 |
| blue | PRR13 | NUMB | 0.026652622 |
| blue | PRR13 | NDEL1 | 0.021929812 |
| blue | PRR13 | MSRB1 | 0.065306662 |
| blue | PRR13 | GCA | 0.028551747 |
| blue | PRR13 | GAB2 | 0.02402412 |
| blue | PRR13 | CSF3R | 0.023585176 |
| blue | PRR13 | CEBPB | 0.027621743 |
| blue | PRR13 | ATP6V1B2 | 0.031835771 |
| blue | PRR13 | ARPC5 | 0.021155083 |
| blue | PRR13 | AQP9 | 0.033641051 |
| blue | PRR13 | ADGRE5 | 0.021483858 |
| blue | PROK2 | PHF21A | 0.021507451 |
| blue | PROK2 | FRAT2 | 0.020100759 |
| blue | PROK2 | FLOT2 | 0.024028041 |
| blue | PROK2 | FBXL5 | 0.023766776 |
| blue | PROK2 | BCL6 | 0.026772352 |

| blue | PROK2 | ACSL1 | 0.041299679 |
| --- | --- | --- | --- |
| blue | PRKCD | NCF4 | 0.027072517 |
| blue | PRKCD | MXD1 | 0.023281003 |
| blue | PRKCD | MSRB1 | 0.026762294 |
| blue | PRKCD | KIFAP3 | 0.022922937 |
| blue | PRKCD | GAB2 | 0.022672363 |
| blue | PRKCD | CSF3R | 0.020198959 |
| blue | PRKCD | CEBPB | 0.021583097 |
| blue | PRKCD | BCL6 | 0.020575143 |
| blue | PRKCD | AQP9 | 0.023546149 |
| blue | PRKCD | ALOX5 | 0.025194196 |
| blue | PRKCD | ACSL1 | 0.020823947 |
| blue | PREX1 | MXD1 | 0.027119767 |
| blue | PREX1 | MSL1 | 0.025039725 |
| blue | PREX1 | EXTL3 | 0.02175636 |
| blue | PRAM1 | LAT2 | 0.020109644 |
| blue | PPP4R1 | PKN2 | 0.020676437 |
| blue | PPP4R1 | PELI2 | 0.029558955 |
| blue | PPP4R1 | NDEL1 | 0.025772062 |
| blue | PPP4R1 | NADK | 0.025514602 |
| blue | PPP4R1 | MXD1 | 0.043939338 |
| blue | PPP4R1 | MSL1 | 0.030886801 |
| blue | PPP4R1 | MME | 0.020188748 |
| blue | PPP4R1 | MBOAT7 | 0.022757587 |
| blue | PPP4R1 | IQGAP1 | 0.020152778 |
| blue | PPP4R1 | GAB2 | 0.033708405 |
| blue | PPP4R1 | FAM160B1 | 0.025666274 |
| blue | PPP4R1 | DYSF | 0.025826031 |
| blue | PPP4R1 | CHST15 | 0.027013953 |
| blue | PPP4R1 | BASP1 | 0.02152121 |
| blue | PPP1R12A | MXD1 | 0.022935047 |
| blue | PPP1R12A | CUX1 | 0.026900605 |
| blue | PPP1R12A | CBL | 0.021162067 |
| blue | PPP1R12A | BAZ2B | 0.020006079 |
| blue | PPFIA1 | MXD1 | 0.02838513 |
| blue | PPFIA1 | MSL1 | 0.021671794 |
| blue | POLR2H | PELI2 | 0.024330882 |
| blue | POLR2H | PELI1 | 0.021975795 |
| blue | POLR2H | NDEL1 | 0.025135885 |
| blue | POLR2H | NADK | 0.022737447 |
| blue | POLR2H | MXD1 | 0.031872339 |
| blue | POLR2H | MBOAT7 | 0.021280486 |
| blue | POLR2H | LYN | 0.02550687 |
| blue | POLR2H | ICAM3 | 0.021280784 |
| blue | POLR2H | GAB2 | 0.029085172 |
| blue | POLR2H | CPSF4 | 0.020675315 |
| blue | POLR2H | C5AR1 | 0.020259002 |
| blue | PLXNC1 | NUMB | 0.025323318 |
| blue | PLXNC1 | NFIL3 | 0.040333455 |
| blue | PLXNC1 | NDEL1 | 0.028537884 |
| blue | PLXNC1 | NCF4 | 0.026844281 |
| blue | PLXNC1 | NAMPT | 0.02899375 |

| blue | PLXNC1 | MXD1 | 0.031521815 |
| --- | --- | --- | --- |
| blue | PLXNC1 | MSRB1 | 0.022522158 |
| blue | PLXNC1 | MGAM | 0.030263758 |
| blue | PLXNC1 | KLHL2 | 0.026744958 |
| blue | PLXNC1 | GCA | 0.028908671 |
| blue | PLXNC1 | GAB2 | 0.029686284 |
| blue | PLXNC1 | CSF3R | 0.028417888 |
| blue | PLXNC1 | CEBPB | 0.030508327 |
| blue | PLXNC1 | BCL6 | 0.05157458 |
| blue | PLXNC1 | AQP9 | 0.050931618 |
| blue | PLXNC1 | ACSL1 | 0.038406981 |
| blue | PLXDC2 | NUMB | 0.024745551 |
| blue | PLXDC2 | NDEL1 | 0.023343457 |
| blue | PLXDC2 | MXD1 | 0.021767286 |
| blue | PLXDC2 | MSRB1 | 0.037675114 |
| blue | PLXDC2 | LYN | 0.033724294 |
| blue | PLXDC2 | IFNGR2 | 0.026114645 |
| blue | PLXDC2 | GCA | 0.027590889 |
| blue | PLXDC2 | GAB2 | 0.025923576 |
| blue | PLXDC2 | BASP1 | 0.020289153 |
| blue | PLXDC2 | ATP6V1B2 | 0.040683531 |
| blue | PLXDC2 | AQP9 | 0.020224867 |
| blue | PLPPR2 | PELI2 | 0.02020694 |
| blue | PLPPR2 | NADK | 0.026977448 |
| blue | PLPPR2 | MYO1F | 0.022925046 |
| blue | PLPPR2 | MXD1 | 0.032797239 |
| blue | PLPPR2 | MSL1 | 0.035766406 |
| blue | PLPPR2 | MBOAT7 | 0.032788525 |
| blue | PLPPR2 | LYN | 0.02028712 |
| blue | PLPPR2 | LAT2 | 0.020662118 |
| blue | PLPPR2 | GAB2 | 0.028918529 |
| blue | PLPPR2 | DYSF | 0.023888416 |
| blue | PLPPR2 | CUX1 | 0.021821451 |
| blue | PLPPR2 | C5AR1 | 0.0261437 |
| blue | PLPPR2 | BASP1 | 0.020082736 |
| blue | PLPPR2 | ARAP1 | 0.028152495 |
| blue | PLEKHO2 | NADK | 0.032700356 |
| blue | PLEKHO2 | MXD1 | 0.025927684 |
| blue | PLEKHO2 | MSL1 | 0.029674407 |
| blue | PLEKHO2 | MBOAT7 | 0.040313312 |
| blue | PLEKHO2 | MAP3K11 | 0.025456542 |
| blue | PLEKHO2 | GAB2 | 0.020506742 |
| blue | PLEKHO2 | DYSF | 0.024631432 |
| blue | PLEKHO2 | C5AR1 | 0.028374718 |
| blue | PLEKHO2 | BASP1 | 0.021153419 |
| blue | PLEKHO2 | ARAP1 | 0.026857736 |
| blue | PLEKHO2 | ADAM8 | 0.022924018 |
| blue | PLBD1 | MSRB1 | 0.022419043 |
| blue | PLBD1 | GCA | 0.026172298 |
| blue | PLBD1 | GAB2 | 0.021370859 |
| blue | PLBD1 | BASP1 | 0.02131433 |
| blue | PLAUR | AQP9 | 0.021236412 |

| blue | PKN2 | PHF2 | 0.025194011 |
| --- | --- | --- | --- |
| blue | PKN2 | PDPK1 | 0.02733599 |
| blue | PKN2 | NADK | 0.023336072 |
| blue | PKN2 | MXD1 | 0.026897171 |
| blue | PKN2 | MSL1 | 0.030290397 |
| blue | PKN2 | MEFV | 0.033075344 |
| blue | PKN2 | MAST3 | 0.020826649 |
| blue | PKN2 | IQGAP1 | 0.021109077 |
| blue | PKN2 | BAZ2B | 0.021913118 |
| blue | PISD | NADK | 0.028565456 |
| blue | PISD | MXD1 | 0.022136995 |
| blue | PIP4P2 | ITM2B | 0.026949844 |
| blue | PILRA | PELI2 | 0.021666773 |
| blue | PILRA | NUMB | 0.023827821 |
| blue | PILRA | NDEL1 | 0.031474827 |
| blue | PILRA | MXD1 | 0.030002256 |
| blue | PILRA | MSRB1 | 0.037482292 |
| blue | PILRA | LYN | 0.021838549 |
| blue | PILRA | GAB2 | 0.035445683 |
| blue | PILRA | CXCR1 | 0.024221088 |
| blue | PILRA | CSF3R | 0.025019059 |
| blue | PILRA | CMTM2 | 0.02059321 |
| blue | PILRA | ATP6V1B2 | 0.042634521 |
| blue | PILRA | AQP9 | 0.023411527 |
| blue | PIK3CD | PHF2 | 0.021810718 |
| blue | PIK3CD | MYO1F | 0.024159285 |
| blue | PIK3CD | MSL1 | 0.022955993 |
| blue | PIK3CD | ARID3A | 0.021737492 |
| blue | PICALM | PELI2 | 0.023124006 |
| blue | PICALM | NADK | 0.026200214 |
| blue | PICALM | MYO1F | 0.022394831 |
| blue | PICALM | MXD1 | 0.038855824 |
| blue | PICALM | MSL1 | 0.031417683 |
| blue | PICALM | MEFV | 0.026344221 |
| blue | PICALM | IQGAP1 | 0.020323488 |
| blue | PICALM | GAB2 | 0.021313818 |
| blue | PICALM | DYSF | 0.024436397 |
| blue | PICALM | ARID3A | 0.021955711 |
| blue | PICALM | ARAP1 | 0.023390008 |
| blue | PHF21A | PFKFB3 | 0.030212287 |
| blue | PHF21A | NUMB | 0.024509014 |
| blue | PHF21A | NIBAN1 | 0.02724829 |
| blue | PHF21A | NFIL3 | 0.041556635 |
| blue | PHF21A | NDEL1 | 0.023119557 |
| blue | PHF21A | NCF4 | 0.021101286 |
| blue | PHF21A | NAMPT | 0.025953241 |
| blue | PHF21A | MXD1 | 0.038560165 |
| blue | PHF21A | MSRB1 | 0.025280362 |
| blue | PHF21A | MSL1 | 0.024051867 |
| blue | PHF21A | MME | 0.021755025 |
| blue | PHF21A | MGAM2 | 0.030482138 |
| blue | PHF21A | MGAM | 0.047420229 |

| blue | PHF21A | LMNB1 | 0.025967063 |
| --- | --- | --- | --- |
| blue | PHF21A | KCNJ15 | 0.035817221 |
| blue | PHF21A | GCA | 0.029705736 |
| blue | PHF21A | GAB2 | 0.036651674 |
| blue | PHF21A | FLOT2 | 0.034045105 |
| blue | PHF21A | FCGR2A | 0.020389078 |
| blue | PHF21A | DYSF | 0.043771219 |
| blue | PHF21A | DENND5A | 0.026563486 |
| blue | PHF21A | CYSTM1 | 0.031616158 |
| blue | PHF21A | CSF3R | 0.024805049 |
| blue | PHF21A | CEBPB | 0.021298771 |
| blue | PHF21A | BCL6 | 0.074404609 |
| blue | PHF21A | BASP1 | 0.036628021 |
| blue | PHF21A | B4GALT5 | 0.022560045 |
| blue | PHF21A | AQP9 | 0.053854069 |
| blue | PHF21A | ANXA3 | 0.03665628 |
| blue | PHF21A | ACSL1 | 0.068822541 |
| blue | PHF20L1 | PELI1 | 0.020136291 |
| blue | PHF20L1 | NDEL1 | 0.023119329 |
| blue | PHF20L1 | MXD1 | 0.05209431 |
| blue | PHF20L1 | MCL1 | 0.027702223 |
| blue | PHF20L1 | GAB2 | 0.026792127 |
| blue | PHF20L1 | C1RL | 0.0200287 |
| blue | PHF2 | IQGAP1 | 0.023927017 |
| blue | PHC2 | PDZD8 | 0.020078196 |
| blue | PHC2 | NADK | 0.021593437 |
| blue | PHC2 | MXD1 | 0.028071649 |
| blue | PHC2 | MEGF9 | 0.021651933 |
| blue | PHC2 | IRS2 | 0.020708194 |
| blue | PHC2 | GAB2 | 0.025843472 |
| blue | PHC2 | EXTL3 | 0.022709334 |
| blue | PHC2 | C3orf62 | 0.020017391 |
| blue | PGS1 | PFKFB3 | 0.029644401 |
| blue | PGS1 | NAMPT | 0.022732988 |
| blue | PGS1 | BCL6 | 0.027089846 |
| blue | PGS1 | ANXA3 | 0.034649243 |
| blue | PGS1 | ACSL1 | 0.056578064 |
| blue | PGLYRP1 | MMP9 | 0.03029609 |
| blue | PGD | MXD1 | 0.027384209 |
| blue | PGD | MSRB1 | 0.022290373 |
| blue | PGD | MME | 0.022631331 |
| blue | PGD | MGAM | 0.023409813 |
| blue | PGD | GAB2 | 0.026934144 |
| blue | PGD | DYSF | 0.026829777 |
| blue | PGD | ARID3A | 0.024578538 |
| blue | PFKFB4 | PACSIN2 | 0.032472856 |
| blue | PFKFB4 | OSBPL2 | 0.020979677 |
| blue | PFKFB4 | NUMB | 0.025031662 |
| blue | PFKFB4 | NCF4 | 0.042287257 |
| blue | PFKFB4 | NARF | 0.02191354 |
| blue | PFKFB4 | MXD1 | 0.022755595 |
| blue | PFKFB4 | MTX1 | 0.022856632 |

| blue | PFKFB4 | MMP25 | 0.039727168 |
| --- | --- | --- | --- |
| blue | PFKFB4 | LRG1 | 0.032920623 |
| blue | PFKFB4 | LILRA2 | 0.038562409 |
| blue | PFKFB4 | ITPRIP | 0.034175256 |
| blue | PFKFB4 | IMPDH1 | 0.038284342 |
| blue | PFKFB4 | IFNGR1 | 0.034667193 |
| blue | PFKFB4 | HSPA1A | 0.020273118 |
| blue | PFKFB4 | HAL | 0.022344419 |
| blue | PFKFB4 | GAB2 | 0.023009618 |
| blue | PFKFB4 | FPR1 | 0.020288181 |
| blue | PFKFB4 | FGR | 0.051900384 |
| blue | PFKFB4 | FAM49A | 0.022163884 |
| blue | PFKFB4 | EXOC6 | 0.026541705 |
| blue | PFKFB4 | DHX34 | 0.027795676 |
| blue | PFKFB4 | CSF3R | 0.043058665 |
| blue | PFKFB4 | CELF2 | 0.029441451 |
| blue | PFKFB4 | CEBPB | 0.039632704 |
| blue | PFKFB4 | CEACAM3 | 0.025592475 |
| blue | PFKFB4 | C1RL | 0.020260014 |
| blue | PFKFB4 | BCL6 | 0.024496122 |
| blue | PFKFB4 | AQP9 | 0.034236109 |
| blue | PFKFB4 | ALOX5 | 0.039702053 |
| blue | PFKFB4 | ADGRE5 | 0.026279488 |
| blue | PFKFB4 | ABTB1 | 0.040802728 |
| blue | PFKFB3 | NFIL3 | 0.039398177 |
| blue | PFKFB3 | NAMPT | 0.042206035 |
| blue | PFKFB3 | MGAM2 | 0.022662279 |
| blue | PFKFB3 | MGAM | 0.02746036 |
| blue | PFKFB3 | MCEMP1 | 0.023404415 |
| blue | PFKFB3 | LMNB1 | 0.036024813 |
| blue | PFKFB3 | KLHL2 | 0.021113741 |
| blue | PFKFB3 | KCNJ15 | 0.023604693 |
| blue | PFKFB3 | GCA | 0.025625519 |
| blue | PFKFB3 | FLOT2 | 0.042587824 |
| blue | PFKFB3 | DYSF | 0.02551957 |
| blue | PFKFB3 | CYSTM1 | 0.02532618 |
| blue | PFKFB3 | BCL6 | 0.056505482 |
| blue | PFKFB3 | BASP1 | 0.026361591 |
| blue | PFKFB3 | AQP9 | 0.04652875 |
| blue | PFKFB3 | ANXA3 | 0.045788202 |
| blue | PFKFB3 | ACSL1 | 0.087725366 |
| blue | PELI2 | PELI1 | 0.023602656 |
| blue | PELI2 | PECAM1 | 0.026978718 |
| blue | PELI2 | PANX2 | 0.027020269 |
| blue | PELI2 | PAK1 | 0.023465813 |
| blue | PELI2 | OSBPL11 | 0.02221481 |
| blue | PELI2 | NDEL1 | 0.032164115 |
| blue | PELI2 | NADK | 0.030471455 |
| blue | PELI2 | MYO1F | 0.020387054 |
| blue | PELI2 | MXD1 | 0.0467281 |
| blue | PELI2 | MSRB1 | 0.025410244 |
| blue | PELI2 | MSL1 | 0.030800538 |

| blue | PELI2 | MME | 0.026562024 |
| --- | --- | --- | --- |
| blue | PELI2 | MGAM | 0.021638961 |
| blue | PELI2 | MBOAT7 | 0.023701592 |
| blue | PELI2 | MAST3 | 0.026022564 |
| blue | PELI2 | MAP2K4 | 0.020125681 |
| blue | PELI2 | LYN | 0.02264759 |
| blue | PELI2 | LITAF | 0.024487053 |
| blue | PELI2 | KIAA0513 | 0.022640038 |
| blue | PELI2 | IGF2R | 0.02895491 |
| blue | PELI2 | ICAM3 | 0.031727035 |
| blue | PELI2 | GAB2 | 0.046976928 |
| blue | PELI2 | DYSF | 0.031047525 |
| blue | PELI2 | CSF3R | 0.023685795 |
| blue | PELI2 | CPD | 0.025485854 |
| blue | PELI2 | CHST15 | 0.02671671 |
| blue | PELI2 | C5AR1 | 0.022606291 |
| blue | PELI2 | C15orf39 | 0.028031813 |
| blue | PELI2 | BASP1 | 0.022504407 |
| blue | PELI2 | ATP6V1B2 | 0.024597952 |
| blue | PELI2 | ARID3A | 0.025863038 |
| blue | PELI2 | AQP9 | 0.023098342 |
| blue | PELI2 | AGTPBP1 | 0.021061471 |
| blue | PELI2 | ADGRE3 | 0.02412987 |
| blue | PELI1 | NEDD9 | 0.022667876 |
| blue | PELI1 | NADK | 0.023536272 |
| blue | PELI1 | MXD1 | 0.037672313 |
| blue | PELI1 | MAST3 | 0.022888539 |
| blue | PELI1 | LRRK2 | 0.029030856 |
| blue | PELI1 | IL1RAP | 0.024874206 |
| blue | PELI1 | HLX | 0.022604483 |
| blue | PELI1 | GAB2 | 0.029532173 |
| blue | PELI1 | CXCR2 | 0.023304181 |
| blue | PELI1 | CXCR1 | 0.028973767 |
| blue | PELI1 | CSF3R | 0.026764707 |
| blue | PELI1 | ALPK1 | 0.025204436 |
| blue | PELI1 | AKR1B1 | 0.024050538 |
| blue | PECAM1 | PAK1 | 0.023230487 |
| blue | PECAM1 | NADK | 0.022639719 |
| blue | PECAM1 | MXD1 | 0.028011801 |
| blue | PECAM1 | MSL1 | 0.024692073 |
| blue | PECAM1 | MEFV | 0.022333748 |
| blue | PECAM1 | MBOAT7 | 0.020571059 |
| blue | PECAM1 | ICAM3 | 0.022099186 |
| blue | PECAM1 | GAB2 | 0.023764596 |
| blue | PECAM1 | ADGRE3 | 0.027347922 |
| blue | PDZD8 | KBTBD7 | 0.021250806 |
| blue | PDPK1 | NIN | 0.027848409 |
| blue | PDPK1 | MXD1 | 0.0201818 |
| blue | PDPK1 | CUX1 | 0.025983116 |
| blue | PDPK1 | CBL | 0.020694756 |
| blue | PDPK1 | ANKRD13A | 0.024260854 |
| blue | PCNX1 | MME | 0.027701563 |

| blue | PCNX1 | MGAM | 0.030848449 |
| --- | --- | --- | --- |
| blue | PCNX1 | IGF2R | 0.023734547 |
| blue | PCNX1 | CHST15 | 0.021339039 |
| blue | PCNX1 | BCL6 | 0.021563726 |
| blue | PCNX1 | AQP9 | 0.023804907 |
| blue | PANX2 | OSBPL2 | 0.03104209 |
| blue | PANX2 | NUMB | 0.03043091 |
| blue | PANX2 | NFIL3 | 0.024076477 |
| blue | PANX2 | NDEL1 | 0.039087935 |
| blue | PANX2 | MXD1 | 0.038516409 |
| blue | PANX2 | MSRB1 | 0.025387729 |
| blue | PANX2 | MME | 0.029028369 |
| blue | PANX2 | MGAM | 0.025717696 |
| blue | PANX2 | KLHL2 | 0.020229534 |
| blue | PANX2 | IGF2R | 0.030818877 |
| blue | PANX2 | GCA | 0.024927201 |
| blue | PANX2 | GAB2 | 0.036189052 |
| blue | PANX2 | CSF3R | 0.028625058 |
| blue | PANX2 | CMTM2 | 0.023189658 |
| blue | PANX2 | CHST15 | 0.030487294 |
| blue | PANX2 | CEBPB | 0.021111979 |
| blue | PANX2 | BCL6 | 0.02531376 |
| blue | PANX2 | AQP9 | 0.03387379 |
| blue | PANX2 | ACOX1 | 0.022190886 |
| blue | PAK1 | OSBPL11 | 0.02128323 |
| blue | PAK1 | NLRP12 | 0.02207176 |
| blue | PAK1 | NDEL1 | 0.022126228 |
| blue | PAK1 | NADK | 0.02664052 |
| blue | PAK1 | MXD1 | 0.043001824 |
| blue | PAK1 | MTMR10 | 0.020998067 |
| blue | PAK1 | MSRB1 | 0.022779282 |
| blue | PAK1 | MSL1 | 0.027305478 |
| blue | PAK1 | MME | 0.024442937 |
| blue | PAK1 | MBOAT7 | 0.020745519 |
| blue | PAK1 | LRRK2 | 0.020527892 |
| blue | PAK1 | LAT2 | 0.023197518 |
| blue | PAK1 | LAMP2 | 0.023802029 |
| blue | PAK1 | JAML | 0.025256594 |
| blue | PAK1 | GAB2 | 0.032673666 |
| blue | PAK1 | DYSF | 0.020758423 |
| blue | PAK1 | CHST15 | 0.022852404 |
| blue | PAK1 | CD93 | 0.023190544 |
| blue | PAK1 | ATP6V1B2 | 0.021240148 |
| blue | PAK1 | ARID3A | 0.021279785 |
| blue | PAK1 | APAF1 | 0.034385258 |
| blue | PADI4 | NLRP12 | 0.021209058 |
| blue | PADI4 | MXD1 | 0.026586073 |
| blue | PADI4 | MMP9 | 0.040533711 |
| blue | PADI4 | MME | 0.021506481 |
| blue | PACSIN2 | OSBPL2 | 0.024186941 |
| blue | PACSIN2 | NUMB | 0.025262966 |
| blue | PACSIN2 | NOTCH1 | 0.046175115 |

| blue | PACSIN2 | NDEL1 | 0.022236199 |
| --- | --- | --- | --- |
| blue | PACSIN2 | NCOA1 | 0.033326509 |
| blue | PACSIN2 | NCF4 | 0.021896663 |
| blue | PACSIN2 | MMP25 | 0.029129533 |
| blue | PACSIN2 | LRG1 | 0.022429405 |
| blue | PACSIN2 | ITPRIP | 0.041067429 |
| blue | PACSIN2 | IMPDH1 | 0.046696644 |
| blue | PACSIN2 | GAB2 | 0.021091049 |
| blue | PACSIN2 | FAM49A | 0.026519841 |
| blue | PACSIN2 | DHX34 | 0.029948807 |
| blue | PACSIN2 | CXCR1 | 0.022092443 |
| blue | PACSIN2 | CSF3R | 0.047211867 |
| blue | PACSIN2 | CEBPB | 0.026096383 |
| blue | PACSIN2 | AQP9 | 0.036305427 |
| blue | PACSIN2 | ABTB1 | 0.0380398 |
| blue | OSGIN2 | NFIL3 | 0.026085547 |
| blue | OSGIN2 | AQP9 | 0.02237185 |
| blue | OSER1 | NDEL1 | 0.021183721 |
| blue | OSCAR | BRI3 | 0.037761057 |
| blue | OSBPL2 | NUMB | 0.023258482 |
| blue | OSBPL2 | NT5C2 | 0.02311119 |
| blue | OSBPL2 | NLRX1 | 0.020503337 |
| blue | OSBPL2 | NDEL1 | 0.043874753 |
| blue | OSBPL2 | NCOA1 | 0.022072839 |
| blue | OSBPL2 | MXD1 | 0.044276881 |
| blue | OSBPL2 | MTMR10 | 0.020092702 |
| blue | OSBPL2 | MSRB1 | 0.021430018 |
| blue | OSBPL2 | MME | 0.0269855 |
| blue | OSBPL2 | MGAM2 | 0.022498263 |
| blue | OSBPL2 | MGAM | 0.026941953 |
| blue | OSBPL2 | LRP10 | 0.02987118 |
| blue | OSBPL2 | LRG1 | 0.02024559 |
| blue | OSBPL2 | ITPRIP | 0.028544491 |
| blue | OSBPL2 | IGF2R | 0.034573827 |
| blue | OSBPL2 | IFRD1 | 0.023125606 |
| blue | OSBPL2 | HAL | 0.037279099 |
| blue | OSBPL2 | GAB2 | 0.039157803 |
| blue | OSBPL2 | FPR1 | 0.020321635 |
| blue | OSBPL2 | FNIP1 | 0.028284474 |
| blue | OSBPL2 | CXCR1 | 0.023940581 |
| blue | OSBPL2 | CTBS | 0.020534648 |
| blue | OSBPL2 | CSF3R | 0.041937306 |
| blue | OSBPL2 | CPD | 0.022199555 |
| blue | OSBPL2 | COP1 | 0.029450875 |
| blue | OSBPL2 | CMTM2 | 0.024944166 |
| blue | OSBPL2 | CHST15 | 0.034869933 |
| blue | OSBPL2 | CEBPB | 0.024119367 |
| blue | OSBPL2 | CCNJL | 0.020225649 |
| blue | OSBPL2 | BEST1 | 0.022744947 |
| blue | OSBPL2 | BCL6 | 0.02441851 |
| blue | OSBPL2 | ATP6V0B | 0.025655625 |
| blue | OSBPL2 | AQP9 | 0.033194898 |

| blue | OSBPL2 | AGO4 | 0.020133049 |
| --- | --- | --- | --- |
| blue | OSBPL2 | ABTB1 | 0.021867898 |
| blue | OSBPL11 | NUMB | 0.024153121 |
| blue | OSBPL11 | NDEL1 | 0.030237834 |
| blue | OSBPL11 | NCF2 | 0.02055853 |
| blue | OSBPL11 | MXD1 | 0.036702991 |
| blue | OSBPL11 | MSRB1 | 0.037340939 |
| blue | OSBPL11 | MSL1 | 0.021008817 |
| blue | OSBPL11 | LYN | 0.023872501 |
| blue | OSBPL11 | IGF2R | 0.020092745 |
| blue | OSBPL11 | HSPA1A | 0.022275012 |
| blue | OSBPL11 | GCA | 0.025854904 |
| blue | OSBPL11 | GAB2 | 0.036828976 |
| blue | OSBPL11 | CHST15 | 0.021627173 |
| blue | OSBPL11 | BCL6 | 0.020145442 |
| blue | OSBPL11 | BASP1 | 0.022700815 |
| blue | OSBPL11 | ATP6V1B2 | 0.030376826 |
| blue | OSBPL11 | AQP9 | 0.02659275 |
| blue | OSBPL11 | ADGRE3 | 0.021859023 |
| blue | NUP214 | NDEL1 | 0.02042226 |
| blue | NUP214 | NADK | 0.023261365 |
| blue | NUP214 | MXD1 | 0.026143568 |
| blue | NUP214 | MSRB1 | 0.03301973 |
| blue | NUP214 | MBOAT7 | 0.033750104 |
| blue | NUP214 | GAB2 | 0.026305908 |
| blue | NUP214 | CMTM2 | 0.025725861 |
| blue | NUP214 | BASP1 | 0.021328831 |
| blue | NUP214 | ATP6V1B2 | 0.022595739 |
| blue | NUMB | NRBF2 | 0.035662885 |
| blue | NUMB | NIBAN1 | 0.028432664 |
| blue | NUMB | NFIL3 | 0.038882281 |
| blue | NUMB | NDEL1 | 0.045832066 |
| blue | NUMB | NCOA1 | 0.022283115 |
| blue | NUMB | NCF4 | 0.022904709 |
| blue | NUMB | NCF2 | 0.026795737 |
| blue | NUMB | NAMPT | 0.035168811 |
| blue | NUMB | MXD1 | 0.041041183 |
| blue | NUMB | MTMR10 | 0.024059564 |
| blue | NUMB | MSRB1 | 0.049904708 |
| blue | NUMB | MNDA | 0.021436764 |
| blue | NUMB | MMP25 | 0.028573315 |
| blue | NUMB | MGAM | 0.021501611 |
| blue | NUMB | MEGF9 | 0.020198698 |
| blue | NUMB | MANSC1 | 0.023071217 |
| blue | NUMB | LYN | 0.027878937 |
| blue | NUMB | LY96 | 0.025375256 |
| blue | NUMB | LRP10 | 0.020160465 |
| blue | NUMB | LRG1 | 0.029250435 |
| blue | NUMB | LMNB1 | 0.021037897 |
| blue | NUMB | LITAF | 0.020274927 |
| blue | NUMB | KLHL2 | 0.026159871 |
| blue | NUMB | ITPRIP | 0.034329968 |

| blue | NUMB | IGF2R | 0.028875894 |
| --- | --- | --- | --- |
| blue | NUMB | IFNGR2 | 0.039751675 |
| blue | NUMB | IFNGR1 | 0.035406824 |
| blue | NUMB | IFITM2 | 0.028713416 |
| blue | NUMB | HSPA1A | 0.035668166 |
| blue | NUMB | HMGCR | 0.020450301 |
| blue | NUMB | HIST2H2AC | 0.0206656 |
| blue | NUMB | HIST1H2BC | 0.021471331 |
| blue | NUMB | HCK | 0.022710342 |
| blue | NUMB | GCA | 0.048793253 |
| blue | NUMB | GAB2 | 0.046391383 |
| blue | NUMB | FRAT2 | 0.035930461 |
| blue | NUMB | FPR1 | 0.038305878 |
| blue | NUMB | FLOT2 | 0.032156302 |
| blue | NUMB | FCGR2A | 0.028088286 |
| blue | NUMB | FBXL5 | 0.022606967 |
| blue | NUMB | EXOC6 | 0.034025879 |
| blue | NUMB | EVI2B | 0.027386682 |
| blue | NUMB | ETS2 | 0.031819688 |
| blue | NUMB | EGLN1 | 0.02170321 |
| blue | NUMB | DNTTIP1 | 0.024756335 |
| blue | NUMB | CYB5R4 | 0.025740152 |
| blue | NUMB | CXCR1 | 0.025783535 |
| blue | NUMB | CSF3R | 0.05283324 |
| blue | NUMB | CPD | 0.026437672 |
| blue | NUMB | COP1 | 0.030727156 |
| blue | NUMB | CMTM6 | 0.020505366 |
| blue | NUMB | CMTM2 | 0.031344386 |
| blue | NUMB | CHST15 | 0.026320357 |
| blue | NUMB | CHMP2A | 0.020160667 |
| blue | NUMB | CELF2 | 0.022721809 |
| blue | NUMB | CEBPB | 0.045807775 |
| blue | NUMB | CEACAM3 | 0.023121213 |
| blue | NUMB | BEST1 | 0.025652397 |
| blue | NUMB | BCL6 | 0.042418941 |
| blue | NUMB | BCL3 | 0.021809336 |
| blue | NUMB | BASP1 | 0.02350065 |
| blue | NUMB | B4GALT5 | 0.023598599 |
| blue | NUMB | ATP6V1B2 | 0.031322358 |
| blue | NUMB | ATP6V0B | 0.024471596 |
| blue | NUMB | AQP9 | 0.065397725 |
| blue | NUMB | AGO4 | 0.023017751 |
| blue | NUMB | ADGRE5 | 0.027705681 |
| blue | NUMB | ACSL1 | 0.035154297 |
| blue | NUMB | ACOX1 | 0.035341551 |
| blue | NT5C2 | NDEL1 | 0.030135845 |
| blue | NT5C2 | NADK | 0.024115012 |
| blue | NT5C2 | MXD1 | 0.059091829 |
| blue | NT5C2 | MSL1 | 0.02029002 |
| blue | NT5C2 | MME | 0.0201679 |
| blue | NT5C2 | MGAM | 0.022164355 |
| blue | NT5C2 | GAB2 | 0.037666016 |

| blue | NT5C2 | DYSF | 0.021805859 |
| --- | --- | --- | --- |
| blue | NT5C2 | CSF3R | 0.025987873 |
| blue | NT5C2 | CHST15 | 0.02370883 |
| blue | NT5C2 | BCL6 | 0.026145219 |
| blue | NT5C2 | AQP9 | 0.024020443 |
| blue | NT5C2 | AKR1B1 | 0.021328538 |
| blue | NT5C2 | ACSL1 | 0.020518072 |
| blue | NT5C | MAP2K4 | 0.043139622 |
| blue | NT5C | CUX1 | 0.039849428 |
| blue | NT5C | CBL | 0.029989901 |
| blue | NSUN7 | KLHL2 | 0.024032973 |
| blue | NSUN7 | IL1R2 | 0.024265885 |
| blue | NSMCE4A | NADK | 0.029420172 |
| blue | NSMCE4A | MXD1 | 0.024343614 |
| blue | NSMCE4A | MSL1 | 0.021867434 |
| blue | NSMCE4A | GAB2 | 0.021679526 |
| blue | NSMCE4A | EXTL3 | 0.025761315 |
| blue | NRBF2 | NFIL3 | 0.049103979 |
| blue | NRBF2 | NDEL1 | 0.040478607 |
| blue | NRBF2 | NCF2 | 0.037207736 |
| blue | NRBF2 | NAMPT | 0.047172877 |
| blue | NRBF2 | MXD1 | 0.027270895 |
| blue | NRBF2 | MSRB1 | 0.033065654 |
| blue | NRBF2 | MNDA | 0.028159643 |
| blue | NRBF2 | LYN | 0.026528935 |
| blue | NRBF2 | LMNB1 | 0.021926684 |
| blue | NRBF2 | KLHL2 | 0.02282196 |
| blue | NRBF2 | IFNGR1 | 0.023364597 |
| blue | NRBF2 | HSD17B11 | 0.040260202 |
| blue | NRBF2 | GCA | 0.046428863 |
| blue | NRBF2 | GAB2 | 0.034779933 |
| blue | NRBF2 | FRAT2 | 0.023158331 |
| blue | NRBF2 | FPR2 | 0.020954373 |
| blue | NRBF2 | FPR1 | 0.020884934 |
| blue | NRBF2 | FOS | 0.021937127 |
| blue | NRBF2 | FCGR2A | 0.025942122 |
| blue | NRBF2 | FBXL5 | 0.028384662 |
| blue | NRBF2 | EVI2B | 0.02391579 |
| blue | NRBF2 | EGLN1 | 0.036898006 |
| blue | NRBF2 | CYB5R4 | 0.032384573 |
| blue | NRBF2 | CXCR2 | 0.021244629 |
| blue | NRBF2 | CSF3R | 0.02869965 |
| blue | NRBF2 | CMTM6 | 0.027319102 |
| blue | NRBF2 | CMTM2 | 0.024578651 |
| blue | NRBF2 | CEBPB | 0.034688581 |
| blue | NRBF2 | CD46 | 0.020744222 |
| blue | NRBF2 | BCL6 | 0.041652486 |
| blue | NRBF2 | BASP1 | 0.026832491 |
| blue | NRBF2 | AQP9 | 0.060929491 |
| blue | NRBF2 | ACSL1 | 0.036323256 |
| blue | NRBF2 | ACOX1 | 0.025757979 |
| blue | NPRL2 | C16orf58 | 0.023935024 |

| blue | NOTCH1 | IMPDH1 | 0.026091502 |
| --- | --- | --- | --- |
| blue | NLRX1 | NFIL3 | 0.022833955 |
| blue | NLRX1 | MME | 0.02447865 |
| blue | NLRX1 | MGAM2 | 0.024917007 |
| blue | NLRX1 | MGAM | 0.051022298 |
| blue | NLRX1 | IGF2R | 0.020413204 |
| blue | NLRX1 | CHST15 | 0.028156786 |
| blue | NLRX1 | BCL6 | 0.026895864 |
| blue | NLRX1 | AQP9 | 0.027034426 |
| blue | NLRP12 | NDEL1 | 0.022191095 |
| blue | NLRP12 | NCF1C | 0.021035125 |
| blue | NLRP12 | NADK | 0.028295955 |
| blue | NLRP12 | MXD1 | 0.03951345 |
| blue | NLRP12 | MSRB1 | 0.024473579 |
| blue | NLRP12 | MSL1 | 0.026419681 |
| blue | NLRP12 | MME | 0.02543151 |
| blue | NLRP12 | MBOAT7 | 0.022766128 |
| blue | NLRP12 | LYN | 0.023341549 |
| blue | NLRP12 | LAT2 | 0.027531251 |
| blue | NLRP12 | HCK | 0.0201522 |
| blue | NLRP12 | GCA | 0.022729306 |
| blue | NLRP12 | GAB2 | 0.031363833 |
| blue | NLRP12 | FBXL5 | 0.022361739 |
| blue | NLRP12 | DYSF | 0.036951768 |
| blue | NLRP12 | BRI3 | 0.020627132 |
| blue | NLRP12 | BCL3 | 0.022245574 |
| blue | NLRP12 | BASP1 | 0.032279164 |
| blue | NLRP12 | ARID3A | 0.020695829 |
| blue | NLRP12 | ARAP1 | 0.021483186 |
| blue | NLRP12 | ANPEP | 0.021089449 |
| blue | NLRP12 | ACSL1 | 0.021058982 |
| blue | NLRC4 | MXD1 | 0.020978661 |
| blue | NLRC4 | MGAM | 0.028454873 |
| blue | NLRC4 | GAB2 | 0.021934758 |
| blue | NLRC4 | DYSF | 0.021664065 |
| blue | NLRC4 | BCL6 | 0.033830634 |
| blue | NLRC4 | AQP9 | 0.024252034 |
| blue | NLRC4 | ACSL1 | 0.025610087 |
| blue | NIN | MXD1 | 0.021453523 |
| blue | NIN | CDC42SE1 | 0.033128074 |
| blue | NIBAN1 | NFIL3 | 0.024257252 |
| blue | NIBAN1 | NDEL1 | 0.032566376 |
| blue | NIBAN1 | NAMPT | 0.020511062 |
| blue | NIBAN1 | MXD1 | 0.039570079 |
| blue | NIBAN1 | MSRB1 | 0.026737781 |
| blue | NIBAN1 | MSL1 | 0.026488699 |
| blue | NIBAN1 | MGAM | 0.022928273 |
| blue | NIBAN1 | LYN | 0.023105886 |
| blue | NIBAN1 | HCK | 0.02326653 |
| blue | NIBAN1 | GCA | 0.028820539 |
| blue | NIBAN1 | GAB2 | 0.039833838 |
| blue | NIBAN1 | FPR1 | 0.023998891 |

| blue | NIBAN1 | FCGR2A | 0.036740765 |
| --- | --- | --- | --- |
| blue | NIBAN1 | ETS2 | 0.042062405 |
| blue | NIBAN1 | DYSF | 0.0269508 |
| blue | NIBAN1 | CXCR1 | 0.043174774 |
| blue | NIBAN1 | CSF3R | 0.036371092 |
| blue | NIBAN1 | CEBPB | 0.021942698 |
| blue | NIBAN1 | BCL6 | 0.039376492 |
| blue | NIBAN1 | BCL3 | 0.020745705 |
| blue | NIBAN1 | BASP1 | 0.028822648 |
| blue | NIBAN1 | AQP9 | 0.040796631 |
| blue | NIBAN1 | ALPK1 | 0.023927084 |
| blue | NIBAN1 | ACSL1 | 0.030662017 |
| blue | NFIL3 | NDEL1 | 0.033757195 |
| blue | NFIL3 | NCF4 | 0.026855369 |
| blue | NFIL3 | NCF2 | 0.028032156 |
| blue | NFIL3 | NAMPT | 0.056151412 |
| blue | NFIL3 | MXD1 | 0.032356193 |
| blue | NFIL3 | MSRB1 | 0.034024629 |
| blue | NFIL3 | MNDA | 0.030324028 |
| blue | NFIL3 | MMP25 | 0.031616078 |
| blue | NFIL3 | MME | 0.024039224 |
| blue | NFIL3 | MGAM2 | 0.033459832 |
| blue | NFIL3 | MGAM | 0.037763711 |
| blue | NFIL3 | MEGF9 | 0.029267671 |
| blue | NFIL3 | MARCKS | 0.021058119 |
| blue | NFIL3 | MAPK1 | 0.025745782 |
| blue | NFIL3 | MANSC1 | 0.043874979 |
| blue | NFIL3 | MAEA | 0.02080403 |
| blue | NFIL3 | LY96 | 0.027079766 |
| blue | NFIL3 | LRG1 | 0.03065754 |
| blue | NFIL3 | LMNB1 | 0.063320614 |
| blue | NFIL3 | KLHL2 | 0.035655206 |
| blue | NFIL3 | KCNJ15 | 0.030198405 |
| blue | NFIL3 | ITPRIP | 0.027010885 |
| blue | NFIL3 | IFNGR2 | 0.021661146 |
| blue | NFIL3 | IFNGR1 | 0.039564839 |
| blue | NFIL3 | IFITM2 | 0.021301476 |
| blue | NFIL3 | HSPA1A | 0.023233621 |
| blue | NFIL3 | HIST2H2AC | 0.034383986 |
| blue | NFIL3 | HIST1H2BE | 0.020696283 |
| blue | NFIL3 | HIST1H2BC | 0.023775558 |
| blue | NFIL3 | GK | 0.023818227 |
| blue | NFIL3 | GCA | 0.04480326 |
| blue | NFIL3 | GAB2 | 0.037395697 |
| blue | NFIL3 | FRAT2 | 0.035464598 |
| blue | NFIL3 | FRAT1 | 0.021501 |
| blue | NFIL3 | FPR1 | 0.027019267 |
| blue | NFIL3 | FOS | 0.026036688 |
| blue | NFIL3 | FLOT2 | 0.046667071 |
| blue | NFIL3 | FCGR2A | 0.031371283 |
| blue | NFIL3 | FBXL5 | 0.037957849 |
| blue | NFIL3 | F5 | 0.032409198 |

| blue | NFIL3 | EXOC6 | 0.030547184 |
| --- | --- | --- | --- |
| blue | NFIL3 | EVI2B | 0.025093655 |
| blue | NFIL3 | ETS2 | 0.029441722 |
| blue | NFIL3 | EGLN1 | 0.044784164 |
| blue | NFIL3 | DYSF | 0.021406895 |
| blue | NFIL3 | CYSTM1 | 0.034638554 |
| blue | NFIL3 | CYB5R4 | 0.031093653 |
| blue | NFIL3 | CSF3R | 0.030586144 |
| blue | NFIL3 | COP1 | 0.021858239 |
| blue | NFIL3 | CMTM6 | 0.023781946 |
| blue | NFIL3 | CMTM2 | 0.024820963 |
| blue | NFIL3 | CELF2 | 0.029226136 |
| blue | NFIL3 | CEBPB | 0.045046023 |
| blue | NFIL3 | CEACAM3 | 0.027140044 |
| blue | NFIL3 | CA4 | 0.023506387 |
| blue | NFIL3 | C1RL | 0.020794518 |
| blue | NFIL3 | BCL6 | 0.07277229 |
| blue | NFIL3 | BCL3 | 0.022832949 |
| blue | NFIL3 | BCL2A1 | 0.035040101 |
| blue | NFIL3 | BASP1 | 0.02587696 |
| blue | NFIL3 | B4GALT5 | 0.036895748 |
| blue | NFIL3 | AQP9 | 0.08098058 |
| blue | NFIL3 | ANXA3 | 0.037515664 |
| blue | NFIL3 | ALOX5 | 0.025289144 |
| blue | NFIL3 | ACSL1 | 0.066078246 |
| blue | NFIL3 | ACOX1 | 0.029059891 |
| blue | NEDD9 | NDEL1 | 0.026134735 |
| blue | NEDD9 | MXD1 | 0.030027875 |
| blue | NEDD9 | GAB2 | 0.024564625 |
| blue | NDEL1 | NCOA1 | 0.027393847 |
| blue | NDEL1 | NCF4 | 0.021608931 |
| blue | NDEL1 | NCF2 | 0.033613111 |
| blue | NDEL1 | NAMPT | 0.038499038 |
| blue | NDEL1 | MXD1 | 0.050544007 |
| blue | NDEL1 | MTMR10 | 0.032081103 |
| blue | NDEL1 | MSRB1 | 0.039334918 |
| blue | NDEL1 | MSL1 | 0.021622948 |
| blue | NDEL1 | MMP25 | 0.024933147 |
| blue | NDEL1 | MME | 0.033064993 |
| blue | NDEL1 | MIR223 | 0.02012995 |
| blue | NDEL1 | MGAM2 | 0.025000495 |
| blue | NDEL1 | MGAM | 0.029150247 |
| blue | NDEL1 | MEGF9 | 0.026907894 |
| blue | NDEL1 | MCL1 | 0.023597267 |
| blue | NDEL1 | MBOAT7 | 0.025209637 |
| blue | NDEL1 | MAST3 | 0.020056956 |
| blue | NDEL1 | MANSC1 | 0.025458116 |
| blue | NDEL1 | LYN | 0.034865951 |
| blue | NDEL1 | LY96 | 0.02314431 |
| blue | NDEL1 | LRRK2 | 0.027425058 |
| blue | NDEL1 | LRP10 | 0.045397569 |
| blue | NDEL1 | LRMP | 0.025678577 |

| blue | NDEL1 | LRG1 | 0.028200193 |
| --- | --- | --- | --- |
| blue | NDEL1 | LITAF | 0.040618731 |
| blue | NDEL1 | LCP1 | 0.020862347 |
| blue | NDEL1 | LAMP2 | 0.022501177 |
| blue | NDEL1 | KLHL2 | 0.025116023 |
| blue | NDEL1 | KIAA0232 | 0.021118007 |
| blue | NDEL1 | KCNJ15 | 0.020601148 |
| blue | NDEL1 | KBTBD7 | 0.024543354 |
| blue | NDEL1 | ITPRIP | 0.037846209 |
| blue | NDEL1 | ITM2B | 0.023446109 |
| blue | NDEL1 | IL6R | 0.030088553 |
| blue | NDEL1 | IL1RAP | 0.021041474 |
| blue | NDEL1 | IGF2R | 0.043898166 |
| blue | NDEL1 | IFRD1 | 0.038524255 |
| blue | NDEL1 | IFNGR2 | 0.025922913 |
| blue | NDEL1 | IFNGR1 | 0.023344965 |
| blue | NDEL1 | IFITM2 | 0.026815033 |
| blue | NDEL1 | ICAM3 | 0.029518448 |
| blue | NDEL1 | HSPA1A | 0.02618891 |
| blue | NDEL1 | HSDL2 | 0.026183636 |
| blue | NDEL1 | HMGCR | 0.020993413 |
| blue | NDEL1 | HLX | 0.023176888 |
| blue | NDEL1 | HIST1H2BC | 0.029636881 |
| blue | NDEL1 | HCK | 0.02298997 |
| blue | NDEL1 | HAL | 0.033753269 |
| blue | NDEL1 | GCA | 0.038296869 |
| blue | NDEL1 | GAB2 | 0.044740505 |
| blue | NDEL1 | FRAT2 | 0.022768911 |
| blue | NDEL1 | FRAT1 | 0.021660108 |
| blue | NDEL1 | FPR1 | 0.044170969 |
| blue | NDEL1 | FOS | 0.025974312 |
| blue | NDEL1 | FNIP1 | 0.029522948 |
| blue | NDEL1 | FNDC3B | 0.022934765 |
| blue | NDEL1 | FLOT2 | 0.022233643 |
| blue | NDEL1 | FCHO2 | 0.023246999 |
| blue | NDEL1 | FCGR2A | 0.03083902 |
| blue | NDEL1 | FAM8A1 | 0.028609658 |
| blue | NDEL1 | FAM49B | 0.025849522 |
| blue | NDEL1 | FAM49A | 0.021224258 |
| blue | NDEL1 | FAM160B1 | 0.024880451 |
| blue | NDEL1 | F11R | 0.020398798 |
| blue | NDEL1 | EXOC6 | 0.027314184 |
| blue | NDEL1 | EVI2B | 0.034185621 |
| blue | NDEL1 | ETS2 | 0.027826223 |
| blue | NDEL1 | EGLN1 | 0.021581196 |
| blue | NDEL1 | DYSF | 0.020951515 |
| blue | NDEL1 | DNTTIP1 | 0.020339785 |
| blue | NDEL1 | CYB5R4 | 0.040544086 |
| blue | NDEL1 | CXCR2 | 0.031568917 |
| blue | NDEL1 | CXCR1 | 0.027434071 |
| blue | NDEL1 | CTBS | 0.029204528 |
| blue | NDEL1 | CSF3R | 0.043319127 |

| blue | NDEL1 | CREBBP | 0.02610599 |
| --- | --- | --- | --- |
| blue | NDEL1 | CPD | 0.035933973 |
| blue | NDEL1 | COP1 | 0.038142947 |
| blue | NDEL1 | CMTM6 | 0.025986903 |
| blue | NDEL1 | CMTM2 | 0.046793418 |
| blue | NDEL1 | CHST15 | 0.038831995 |
| blue | NDEL1 | CHMP2A | 0.02141109 |
| blue | NDEL1 | CEBPB | 0.034880972 |
| blue | NDEL1 | CD46 | 0.028914504 |
| blue | NDEL1 | CCNJL | 0.020704359 |
| blue | NDEL1 | CA4 | 0.023102382 |
| blue | NDEL1 | C15orf39 | 0.020325576 |
| blue | NDEL1 | BTBD10 | 0.023509129 |
| blue | NDEL1 | BEST1 | 0.033746004 |
| blue | NDEL1 | BCL6 | 0.034187532 |
| blue | NDEL1 | BCL3 | 0.028574967 |
| blue | NDEL1 | BCL2A1 | 0.020145398 |
| blue | NDEL1 | BASP1 | 0.029714678 |
| blue | NDEL1 | B4GALT5 | 0.024813154 |
| blue | NDEL1 | ATP6V1B2 | 0.036473611 |
| blue | NDEL1 | ATP6V0B | 0.040554728 |
| blue | NDEL1 | ASPRV1 | 0.021864422 |
| blue | NDEL1 | ARHGAP9 | 0.026626052 |
| blue | NDEL1 | ARHGAP25 | 0.02257014 |
| blue | NDEL1 | AQP9 | 0.045189746 |
| blue | NDEL1 | ALOX5AP | 0.024606448 |
| blue | NDEL1 | AGO4 | 0.023804371 |
| blue | NDEL1 | ADGRE5 | 0.028040156 |
| blue | NDEL1 | ADGRE3 | 0.03111807 |
| blue | NDEL1 | ACSL1 | 0.028911653 |
| blue | NDEL1 | ACOX1 | 0.038050951 |
| blue | NDEL1 | ABHD5 | 0.031100831 |
| blue | NCOA1 | MXD1 | 0.022466197 |
| blue | NCOA1 | ITPRIP | 0.0344438 |
| blue | NCOA1 | GAB2 | 0.02646408 |
| blue | NCOA1 | CXCR1 | 0.031906305 |
| blue | NCOA1 | CSF3R | 0.044474098 |
| blue | NCOA1 | AQP9 | 0.03195711 |
| blue | NCF4 | NARF | 0.023295826 |
| blue | NCF4 | NAMPT | 0.031479995 |
| blue | NCF4 | MXD1 | 0.027630242 |
| blue | NCF4 | MTX1 | 0.021274199 |
| blue | NCF4 | MMP25 | 0.058840993 |
| blue | NCF4 | MAEA | 0.025116725 |
| blue | NCF4 | LRG1 | 0.031441607 |
| blue | NCF4 | LILRA2 | 0.025524329 |
| blue | NCF4 | KLHL2 | 0.020838357 |
| blue | NCF4 | KIFAP3 | 0.021846246 |
| blue | NCF4 | ITPRIP | 0.028332873 |
| blue | NCF4 | IMPDH1 | 0.030977174 |
| blue | NCF4 | IFNGR1 | 0.02088896 |
| blue | NCF4 | HAL | 0.021534983 |

| blue | NCF4 | GCA | 0.020228487 |
| --- | --- | --- | --- |
| blue | NCF4 | GAB2 | 0.023766674 |
| blue | NCF4 | FGR | 0.02207846 |
| blue | NCF4 | EXOC6 | 0.023824785 |
| blue | NCF4 | CSF3R | 0.037767435 |
| blue | NCF4 | CELF2 | 0.023446972 |
| blue | NCF4 | CEBPB | 0.034668159 |
| blue | NCF4 | CEACAM3 | 0.031427017 |
| blue | NCF4 | C1RL | 0.033822243 |
| blue | NCF4 | BCL6 | 0.042151859 |
| blue | NCF4 | AQP9 | 0.042729088 |
| blue | NCF4 | ALOX5 | 0.06095604 |
| blue | NCF4 | ACSL1 | 0.034160882 |
| blue | NCF4 | ABTB1 | 0.031732842 |
| blue | NCF2 | NAMPT | 0.031460408 |
| blue | NCF2 | NADK | 0.020157032 |
| blue | NCF2 | MXD1 | 0.035388999 |
| blue | NCF2 | MSRB1 | 0.032793122 |
| blue | NCF2 | MNDA | 0.020790371 |
| blue | NCF2 | LYN | 0.029283289 |
| blue | NCF2 | LRRK2 | 0.023643772 |
| blue | NCF2 | LRMP | 0.022547248 |
| blue | NCF2 | LITAF | 0.02198475 |
| blue | NCF2 | IFNGR2 | 0.024472357 |
| blue | NCF2 | HLX | 0.020629798 |
| blue | NCF2 | HCK | 0.024758824 |
| blue | NCF2 | GCA | 0.043619867 |
| blue | NCF2 | GAB2 | 0.035278343 |
| blue | NCF2 | FRAT2 | 0.024314341 |
| blue | NCF2 | FPR1 | 0.024049089 |
| blue | NCF2 | FOS | 0.021004117 |
| blue | NCF2 | FCGR2A | 0.023839556 |
| blue | NCF2 | FBXL5 | 0.023679435 |
| blue | NCF2 | EVI2B | 0.022304646 |
| blue | NCF2 | EGLN1 | 0.021352693 |
| blue | NCF2 | CYB5R4 | 0.026319016 |
| blue | NCF2 | CXCR2 | 0.02013784 |
| blue | NCF2 | CXCR1 | 0.020671606 |
| blue | NCF2 | CSF3R | 0.02814247 |
| blue | NCF2 | CMTM6 | 0.028985505 |
| blue | NCF2 | CMTM2 | 0.02043026 |
| blue | NCF2 | CEBPB | 0.025281346 |
| blue | NCF2 | BCL6 | 0.031342382 |
| blue | NCF2 | BCL3 | 0.020683463 |
| blue | NCF2 | BASP1 | 0.024583915 |
| blue | NCF2 | ATP6V1B2 | 0.023626002 |
| blue | NCF2 | AQP9 | 0.039824893 |
| blue | NCF2 | ACSL1 | 0.027954353 |
| blue | NCF2 | ACOX1 | 0.024958726 |
| blue | NCF1C | NCF1 | 0.032060076 |
| blue | NCF1C | NADK | 0.028009648 |
| blue | NCF1C | MXD1 | 0.041952342 |

| blue | NCF1C | MSRB1 | 0.024104805 |
| --- | --- | --- | --- |
| blue | NCF1C | MSL1 | 0.021433136 |
| blue | NCF1C | MME | 0.021386023 |
| blue | NCF1C | MBOAT7 | 0.025648078 |
| blue | NCF1C | LAT2 | 0.034969154 |
| blue | NCF1C | GAB2 | 0.028960699 |
| blue | NCF1C | DYSF | 0.021475911 |
| blue | NCF1C | ARID3A | 0.021008843 |
| blue | NARF | BCL6 | 0.027776271 |
| blue | NARF | AQP9 | 0.02283954 |
| blue | NARF | ACSL1 | 0.022160237 |
| blue | NAMPT | MXD1 | 0.028796623 |
| blue | NAMPT | MSRB1 | 0.031903594 |
| blue | NAMPT | MNDA | 0.055461913 |
| blue | NAMPT | MMP25 | 0.023776345 |
| blue | NAMPT | MGAM | 0.022449796 |
| blue | NAMPT | MANSC1 | 0.020991566 |
| blue | NAMPT | MAEA | 0.025812435 |
| blue | NAMPT | LYN | 0.020273295 |
| blue | NAMPT | LY96 | 0.020551497 |
| blue | NAMPT | LRG1 | 0.025401149 |
| blue | NAMPT | LMNB1 | 0.049094391 |
| blue | NAMPT | KLHL2 | 0.026061497 |
| blue | NAMPT | KCNJ15 | 0.025690286 |
| blue | NAMPT | ITPRIP | 0.023717487 |
| blue | NAMPT | IFNGR2 | 0.021086404 |
| blue | NAMPT | IFNGR1 | 0.023475003 |
| blue | NAMPT | HSD17B11 | 0.022161961 |
| blue | NAMPT | GK | 0.044082667 |
| blue | NAMPT | GCA | 0.048585048 |
| blue | NAMPT | GAB2 | 0.031312126 |
| blue | NAMPT | FRAT2 | 0.032459171 |
| blue | NAMPT | FPR2 | 0.021192246 |
| blue | NAMPT | FPR1 | 0.024239941 |
| blue | NAMPT | FOS | 0.021160855 |
| blue | NAMPT | FLOT2 | 0.037793374 |
| blue | NAMPT | FCGR2A | 0.034344277 |
| blue | NAMPT | FBXL5 | 0.050400328 |
| blue | NAMPT | EVI2B | 0.021947128 |
| blue | NAMPT | EGLN1 | 0.027785152 |
| blue | NAMPT | CYSTM1 | 0.022294505 |
| blue | NAMPT | CYB5R4 | 0.026809232 |
| blue | NAMPT | CSF3R | 0.032722567 |
| blue | NAMPT | CELF2 | 0.024304383 |
| blue | NAMPT | CEBPB | 0.040631334 |
| blue | NAMPT | CEACAM3 | 0.027548114 |
| blue | NAMPT | CD46 | 0.021078717 |
| blue | NAMPT | CA4 | 0.020112702 |
| blue | NAMPT | BCL6 | 0.068109899 |
| blue | NAMPT | BCL3 | 0.023880266 |
| blue | NAMPT | BASP1 | 0.026313229 |
| blue | NAMPT | B4GALT5 | 0.020326795 |

| blue | NAMPT | AQP9 | 0.079660024 |
| --- | --- | --- | --- |
| blue | NAMPT | ANXA3 | 0.036797765 |
| blue | NAMPT | ALOX5 | 0.028769607 |
| blue | NAMPT | ACSL1 | 0.0671973 |
| blue | NAMPT | ACOX1 | 0.023826599 |
| blue | NADK | MYO1F | 0.025018068 |
| blue | NADK | MXD1 | 0.039676022 |
| blue | NADK | MSL1 | 0.032562467 |
| blue | NADK | MOSPD2 | 0.022308436 |
| blue | NADK | MME | 0.028075213 |
| blue | NADK | MGAM | 0.022305947 |
| blue | NADK | MEFV | 0.02697514 |
| blue | NADK | MCL1 | 0.022912525 |
| blue | NADK | MBOAT7 | 0.037169976 |
| blue | NADK | MAST3 | 0.023338508 |
| blue | NADK | MAP4K1 | 0.024114061 |
| blue | NADK | MAP3K5 | 0.027843613 |
| blue | NADK | MAP3K11 | 0.023127949 |
| blue | NADK | LYN | 0.023042103 |
| blue | NADK | LRRK2 | 0.024145522 |
| blue | NADK | LRMP | 0.021577188 |
| blue | NADK | LITAF | 0.022203883 |
| blue | NADK | LAT2 | 0.03016294 |
| blue | NADK | KIAA0513 | 0.028004315 |
| blue | NADK | KCNJ15 | 0.020413581 |
| blue | NADK | ITPK1 | 0.026180128 |
| blue | NADK | IQSEC1 | 0.023791666 |
| blue | NADK | IQGAP1 | 0.020087621 |
| blue | NADK | IL17RA | 0.025013698 |
| blue | NADK | ICAM3 | 0.021153572 |
| blue | NADK | HLX | 0.02228531 |
| blue | NADK | HCK | 0.024827339 |
| blue | NADK | GAB2 | 0.030777184 |
| blue | NADK | FNDC3B | 0.022532075 |
| blue | NADK | FAM8A1 | 0.02072493 |
| blue | NADK | DYSF | 0.034774407 |
| blue | NADK | DENND5A | 0.026133314 |
| blue | NADK | CXCR1 | 0.023105776 |
| blue | NADK | CPSF4 | 0.022188357 |
| blue | NADK | CHST15 | 0.02690089 |
| blue | NADK | CFLAR | 0.020142806 |
| blue | NADK | CCNJL | 0.021420673 |
| blue | NADK | CANT1 | 0.026505368 |
| blue | NADK | C5AR1 | 0.026172279 |
| blue | NADK | C3orf62 | 0.025743842 |
| blue | NADK | C16orf58 | 0.02541971 |
| blue | NADK | C15orf39 | 0.027755245 |
| blue | NADK | BCL3 | 0.022353995 |
| blue | NADK | BAZ2B | 0.0242731 |
| blue | NADK | BASP1 | 0.026199604 |
| blue | NADK | ATG16L2 | 0.020303368 |
| blue | NADK | ARID3A | 0.029938376 |

| blue | NADK | ARAP1 | 0.036180757 |
| --- | --- | --- | --- |
| blue | NADK | ANPEP | 0.022579451 |
| blue | NADK | ALPK1 | 0.021300808 |
| blue | NADK | AKR1B1 | 0.022599279 |
| blue | NADK | AGTPBP1 | 0.021540492 |
| blue | NADK | ADAM8 | 0.028684693 |
| blue | MYO9B | MYO1F | 0.030135241 |
| blue | MYO9B | MXD1 | 0.024904499 |
| blue | MYO9B | ARAP1 | 0.023088898 |
| blue | MYO1F | MXD1 | 0.048868923 |
| blue | MYO1F | MSL1 | 0.039128506 |
| blue | MYO1F | MME | 0.020619965 |
| blue | MYO1F | MBOAT7 | 0.032365769 |
| blue | MYO1F | IQSEC1 | 0.02519601 |
| blue | MYO1F | IQGAP1 | 0.021833549 |
| blue | MYO1F | GAB2 | 0.027326368 |
| blue | MYO1F | DYSF | 0.025313075 |
| blue | MYO1F | CANT1 | 0.022167458 |
| blue | MYO1F | BAZ2B | 0.021906758 |
| blue | MYO1F | ARAP1 | 0.027986505 |
| blue | MYO1F | AGTPBP1 | 0.02011244 |
| blue | MYO1F | ADAM8 | 0.022941223 |
| blue | MYD88 | MTF1 | 0.024521099 |
| blue | MYD88 | BRI3 | 0.021142589 |
| blue | MXD1 | MTMR10 | 0.031483596 |
| blue | MXD1 | MSRB1 | 0.038084502 |
| blue | MXD1 | MSL1 | 0.045475699 |
| blue | MXD1 | MOSPD2 | 0.031752072 |
| blue | MXD1 | MMP25 | 0.025886176 |
| blue | MXD1 | MME | 0.052886871 |
| blue | MXD1 | MIR223 | 0.022975356 |
| blue | MXD1 | MGAM2 | 0.033338963 |
| blue | MXD1 | MGAM | 0.04536088 |
| blue | MXD1 | MEGF9 | 0.036581684 |
| blue | MXD1 | MEFV | 0.024195458 |
| blue | MXD1 | MED13L | 0.023931017 |
| blue | MXD1 | MCL1 | 0.06307457 |
| blue | MXD1 | MBOAT7 | 0.047284078 |
| blue | MXD1 | MAST3 | 0.036359279 |
| blue | MXD1 | 1-Mar | 0.022308851 |
| blue | MXD1 | MAP4K1 | 0.029530246 |
| blue | MXD1 | MAP3K5 | 0.026522231 |
| blue | MXD1 | MAP3K2 | 0.025534933 |
| blue | MXD1 | LYST | 0.02935944 |
| blue | MXD1 | LYN | 0.038402003 |
| blue | MXD1 | LY96 | 0.021199269 |
| blue | MXD1 | LRRK2 | 0.048499555 |
| blue | MXD1 | LRP10 | 0.034566096 |
| blue | MXD1 | LRMP | 0.051780653 |
| blue | MXD1 | LPGAT1 | 0.023198705 |
| blue | MXD1 | LITAF | 0.0364369 |
| blue | MXD1 | LINC02035 | 0.023345619 |

| blue | MXD1 | LCP1 | 0.023775519 |
| --- | --- | --- | --- |
| blue | MXD1 | LAT2 | 0.030862732 |
| blue | MXD1 | LAMP2 | 0.023172942 |
| blue | MXD1 | KLHL2 | 0.030197495 |
| blue | MXD1 | KIF1B | 0.029767776 |
| blue | MXD1 | KIAA0513 | 0.020984205 |
| blue | MXD1 | KIAA0319L | 0.020513447 |
| blue | MXD1 | KIAA0232 | 0.020178736 |
| blue | MXD1 | KDM6B | 0.023205453 |
| blue | MXD1 | KCNJ15 | 0.031853768 |
| blue | MXD1 | KBTBD7 | 0.035246911 |
| blue | MXD1 | JAML | 0.029660632 |
| blue | MXD1 | ITPRIP | 0.034929462 |
| blue | MXD1 | ITPK1 | 0.022447778 |
| blue | MXD1 | ITM2B | 0.021302611 |
| blue | MXD1 | ITGAM | 0.024502489 |
| blue | MXD1 | IRS2 | 0.022342865 |
| blue | MXD1 | IQSEC1 | 0.033734088 |
| blue | MXD1 | IQGAP1 | 0.024830106 |
| blue | MXD1 | IL6R | 0.031915028 |
| blue | MXD1 | IL1RAP | 0.023632393 |
| blue | MXD1 | IL17RA | 0.023597616 |
| blue | MXD1 | IGF2R | 0.035812875 |
| blue | MXD1 | IFRD1 | 0.042497899 |
| blue | MXD1 | IFNGR2 | 0.02609928 |
| blue | MXD1 | IFITM2 | 0.024409725 |
| blue | MXD1 | ICAM3 | 0.031181833 |
| blue | MXD1 | HSPA1A | 0.024835121 |
| blue | MXD1 | HSDL2 | 0.022557592 |
| blue | MXD1 | HLX | 0.034539934 |
| blue | MXD1 | HCK | 0.034848319 |
| blue | MXD1 | HAL | 0.035116346 |
| blue | MXD1 | GCA | 0.036394391 |
| blue | MXD1 | GAB2 | 0.053616487 |
| blue | MXD1 | G6PD | 0.020916832 |
| blue | MXD1 | FRAT2 | 0.022246882 |
| blue | MXD1 | FRAT1 | 0.03324455 |
| blue | MXD1 | FPR1 | 0.037895204 |
| blue | MXD1 | FOS | 0.02386818 |
| blue | MXD1 | FNIP1 | 0.034099897 |
| blue | MXD1 | FNDC3B | 0.040226718 |
| blue | MXD1 | FCHO2 | 0.033578261 |
| blue | MXD1 | FCGR2A | 0.029303548 |
| blue | MXD1 | FBXL5 | 0.021188705 |
| blue | MXD1 | FAM8A1 | 0.050900567 |
| blue | MXD1 | FAM49B | 0.027392682 |
| blue | MXD1 | FAM160B1 | 0.042763583 |
| blue | MXD1 | F5 | 0.020472066 |
| blue | MXD1 | F11R | 0.022725122 |
| blue | MXD1 | EXOC6 | 0.038399148 |
| blue | MXD1 | ETS2 | 0.026832692 |
| blue | MXD1 | DYSF | 0.043758475 |

| blue | MXD1 | DIP2B | 0.02558102 |
| --- | --- | --- | --- |
| blue | MXD1 | DENND5A | 0.040309807 |
| blue | MXD1 | DCP2 | 0.021261718 |
| blue | MXD1 | CYB5R4 | 0.036906455 |
| blue | MXD1 | CXCR2 | 0.021144863 |
| blue | MXD1 | CXCR1 | 0.033338556 |
| blue | MXD1 | CUX1 | 0.021390214 |
| blue | MXD1 | CTBS | 0.042368915 |
| blue | MXD1 | CSF3R | 0.037913739 |
| blue | MXD1 | CPSF4 | 0.033038315 |
| blue | MXD1 | CPPED1 | 0.023320664 |
| blue | MXD1 | CPEB2 | 0.025125564 |
| blue | MXD1 | CPD | 0.04226986 |
| blue | MXD1 | COP1 | 0.037528631 |
| blue | MXD1 | CMTM6 | 0.027339526 |
| blue | MXD1 | CMTM2 | 0.033440606 |
| blue | MXD1 | CHST15 | 0.04807537 |
| blue | MXD1 | CFLAR | 0.039207628 |
| blue | MXD1 | CEBPD | 0.028743045 |
| blue | MXD1 | CEBPB | 0.029319941 |
| blue | MXD1 | CDA | 0.020387178 |
| blue | MXD1 | CD46 | 0.024993959 |
| blue | MXD1 | CCNJL | 0.027550092 |
| blue | MXD1 | CANT1 | 0.02648695 |
| blue | MXD1 | C5AR1 | 0.024934594 |
| blue | MXD1 | C3orf62 | 0.038633511 |
| blue | MXD1 | C1RL | 0.030943531 |
| blue | MXD1 | C15orf39 | 0.027527015 |
| blue | MXD1 | BMS1 | 0.023213902 |
| blue | MXD1 | BEST1 | 0.047830108 |
| blue | MXD1 | BCL6 | 0.041347663 |
| blue | MXD1 | BCL3 | 0.031446418 |
| blue | MXD1 | BCL2A1 | 0.025704911 |
| blue | MXD1 | BAZ2B | 0.038346543 |
| blue | MXD1 | BASP1 | 0.037431873 |
| blue | MXD1 | B4GALT5 | 0.031201616 |
| blue | MXD1 | ATP6V1B2 | 0.033976397 |
| blue | MXD1 | ATP6V0B | 0.02704637 |
| blue | MXD1 | ATG16L2 | 0.036479054 |
| blue | MXD1 | ASPRV1 | 0.020954863 |
| blue | MXD1 | ARID3A | 0.032817587 |
| blue | MXD1 | ARHGAP9 | 0.032415561 |
| blue | MXD1 | ARHGAP25 | 0.023957851 |
| blue | MXD1 | ARAP1 | 0.042957607 |
| blue | MXD1 | AQP9 | 0.036790125 |
| blue | MXD1 | ANPEP | 0.031736734 |
| blue | MXD1 | ANKRD13A | 0.046122089 |
| blue | MXD1 | ALPK1 | 0.034552053 |
| blue | MXD1 | ALOX5AP | 0.022044293 |
| blue | MXD1 | AKR1B1 | 0.038649534 |
| blue | MXD1 | AKIRIN2 | 0.021722006 |
| blue | MXD1 | AGTPBP1 | 0.04259347 |

| blue | MXD1 | AGO4 | 0.027790673 |
| --- | --- | --- | --- |
| blue | MXD1 | ADGRE5 | 0.024680648 |
| blue | MXD1 | ADGRE3 | 0.036280646 |
| blue | MXD1 | ADAM8 | 0.031768397 |
| blue | MXD1 | ACSL4 | 0.033441944 |
| blue | MXD1 | ACSL1 | 0.035059282 |
| blue | MXD1 | ACOX1 | 0.033836859 |
| blue | MXD1 | ABHD5 | 0.028687825 |
| blue | MX2 | IL1RN | 0.025256682 |
| blue | MTX1 | IMPDH1 | 0.020713117 |
| blue | MTX1 | CSF3R | 0.022598269 |
| blue | MTX1 | CEBPB | 0.027684823 |
| blue | MTX1 | AQP9 | 0.02576025 |
| blue | MTMR10 | MSRB1 | 0.029617441 |
| blue | MTMR10 | MME | 0.025143859 |
| blue | MTMR10 | MGAM | 0.023026553 |
| blue | MTMR10 | IGF2R | 0.02297636 |
| blue | MTMR10 | GAB2 | 0.027698185 |
| blue | MTMR10 | CSF3R | 0.021942464 |
| blue | MTMR10 | CPD | 0.021640523 |
| blue | MTMR10 | COP1 | 0.030030262 |
| blue | MTMR10 | CMTM2 | 0.031223176 |
| blue | MTMR10 | CHST15 | 0.030020781 |
| blue | MTMR10 | BCL6 | 0.021036647 |
| blue | MTMR10 | AQP9 | 0.026333721 |
| blue | MTF1 | CUX1 | 0.027471336 |
| blue | MTF1 | C16orf58 | 0.028766198 |
| blue | MSRB1 | MNDA | 0.023768802 |
| blue | MSRB1 | MMP25 | 0.021907288 |
| blue | MSRB1 | MME | 0.027328338 |
| blue | MSRB1 | MIDN | 0.020873173 |
| blue | MSRB1 | MGAM | 0.025400645 |
| blue | MSRB1 | MEGF9 | 0.020822407 |
| blue | MSRB1 | MBOAT7 | 0.027330539 |
| blue | MSRB1 | LYN | 0.042055521 |
| blue | MSRB1 | LY96 | 0.036068434 |
| blue | MSRB1 | LRRK2 | 0.024213946 |
| blue | MSRB1 | LRG1 | 0.024854608 |
| blue | MSRB1 | LITAF | 0.032750285 |
| blue | MSRB1 | LCP1 | 0.051527036 |
| blue | MSRB1 | LAT2 | 0.022011105 |
| blue | MSRB1 | LAMP2 | 0.021553282 |
| blue | MSRB1 | KCNJ15 | 0.022556988 |
| blue | MSRB1 | ITPRIP | 0.038085094 |
| blue | MSRB1 | IL6R | 0.0228558 |
| blue | MSRB1 | IL1RN | 0.032072514 |
| blue | MSRB1 | IGF2R | 0.034177665 |
| blue | MSRB1 | IFNGR2 | 0.061407076 |
| blue | MSRB1 | IFNGR1 | 0.025838057 |
| blue | MSRB1 | IFNAR1 | 0.021064346 |
| blue | MSRB1 | IFITM2 | 0.042721181 |
| blue | MSRB1 | ICAM3 | 0.021633196 |

| blue | MSRB1 | HSPA1A | 0.047763484 |
| --- | --- | --- | --- |
| blue | MSRB1 | HMGCR | 0.036899614 |
| blue | MSRB1 | HLX | 0.032045842 |
| blue | MSRB1 | HIST2H2AC | 0.020082823 |
| blue | MSRB1 | HCK | 0.039336212 |
| blue | MSRB1 | GMFG | 0.023898683 |
| blue | MSRB1 | GCA | 0.040831942 |
| blue | MSRB1 | GAB2 | 0.045953767 |
| blue | MSRB1 | FRAT2 | 0.036300301 |
| blue | MSRB1 | FPR1 | 0.044698173 |
| blue | MSRB1 | FNDC3B | 0.020575868 |
| blue | MSRB1 | FLOT2 | 0.026599984 |
| blue | MSRB1 | FGR | 0.020935436 |
| blue | MSRB1 | FGL2 | 0.025848209 |
| blue | MSRB1 | FCGRT | 0.023087368 |
| blue | MSRB1 | FCGR2A | 0.029347953 |
| blue | MSRB1 | FBXL5 | 0.029620415 |
| blue | MSRB1 | FAM8A1 | 0.020425771 |
| blue | MSRB1 | EXOC6 | 0.03212721 |
| blue | MSRB1 | EVI2B | 0.028440541 |
| blue | MSRB1 | ETS2 | 0.025474513 |
| blue | MSRB1 | EGLN1 | 0.022090312 |
| blue | MSRB1 | DYSF | 0.022328634 |
| blue | MSRB1 | DNTTIP1 | 0.024329569 |
| blue | MSRB1 | DENND5A | 0.021195385 |
| blue | MSRB1 | DAZAP2 | 0.025140399 |
| blue | MSRB1 | CYB5R4 | 0.03042967 |
| blue | MSRB1 | CXCR1 | 0.022479702 |
| blue | MSRB1 | CSF3R | 0.037009829 |
| blue | MSRB1 | CPD | 0.033281859 |
| blue | MSRB1 | COP1 | 0.040493575 |
| blue | MSRB1 | CMTM6 | 0.027999036 |
| blue | MSRB1 | CMTM2 | 0.044295841 |
| blue | MSRB1 | CHST15 | 0.028351861 |
| blue | MSRB1 | CHMP2A | 0.025525554 |
| blue | MSRB1 | CEBPB | 0.03690337 |
| blue | MSRB1 | CAP1 | 0.023672 |
| blue | MSRB1 | C15orf39 | 0.027433261 |
| blue | MSRB1 | BST1 | 0.021959385 |
| blue | MSRB1 | BEST1 | 0.021197339 |
| blue | MSRB1 | BCL6 | 0.03407232 |
| blue | MSRB1 | BCL3 | 0.030499643 |
| blue | MSRB1 | BASP1 | 0.035378356 |
| blue | MSRB1 | ATP6V1B2 | 0.058936275 |
| blue | MSRB1 | ATP6V1A | 0.021301706 |
| blue | MSRB1 | ATP6V0B | 0.039284389 |
| blue | MSRB1 | ARPC5 | 0.031813233 |
| blue | MSRB1 | ARID3A | 0.022991323 |
| blue | MSRB1 | AQP9 | 0.052732849 |
| blue | MSRB1 | APOBR | 0.022616208 |
| blue | MSRB1 | ALOX5AP | 0.032242659 |
| blue | MSRB1 | AGO4 | 0.026786611 |

| blue | MSRB1 | ADGRE5 | 0.040519439 |
| --- | --- | --- | --- |
| blue | MSRB1 | ADGRE3 | 0.022482697 |
| blue | MSRB1 | ACSL1 | 0.030322137 |
| blue | MSRB1 | ACOX1 | 0.030329257 |
| blue | MSL1 | MME | 0.027095204 |
| blue | MSL1 | MGAM2 | 0.020418264 |
| blue | MSL1 | MGAM | 0.024793168 |
| blue | MSL1 | MEFV | 0.027942528 |
| blue | MSL1 | MED13L | 0.031019093 |
| blue | MSL1 | MCL1 | 0.021057394 |
| blue | MSL1 | MBOAT7 | 0.034806532 |
| blue | MSL1 | MAST3 | 0.03090696 |
| blue | MSL1 | MAP3K5 | 0.022321815 |
| blue | MSL1 | MAP3K2 | 0.03419498 |
| blue | MSL1 | MAP3K11 | 0.021153066 |
| blue | MSL1 | MAP2K4 | 0.020025466 |
| blue | MSL1 | LYN | 0.022476402 |
| blue | MSL1 | LRRK2 | 0.021413077 |
| blue | MSL1 | LITAF | 0.022048185 |
| blue | MSL1 | LCP1 | 0.021209007 |
| blue | MSL1 | LAT2 | 0.026020486 |
| blue | MSL1 | KIF1B | 0.023255607 |
| blue | MSL1 | KDM6B | 0.027636118 |
| blue | MSL1 | IQSEC1 | 0.044456953 |
| blue | MSL1 | IQGAP1 | 0.028835661 |
| blue | MSL1 | IL17RA | 0.023192856 |
| blue | MSL1 | ICAM3 | 0.025627742 |
| blue | MSL1 | HCK | 0.026872731 |
| blue | MSL1 | GLT1D1 | 0.021650014 |
| blue | MSL1 | GAB2 | 0.034964861 |
| blue | MSL1 | FAM160B1 | 0.02510334 |
| blue | MSL1 | DYSF | 0.033784114 |
| blue | MSL1 | DIP2B | 0.025177734 |
| blue | MSL1 | DENND5A | 0.026526605 |
| blue | MSL1 | DCP2 | 0.023355662 |
| blue | MSL1 | CXCR1 | 0.020528984 |
| blue | MSL1 | CUX1 | 0.022962094 |
| blue | MSL1 | CTBS | 0.020619072 |
| blue | MSL1 | CPD | 0.022294695 |
| blue | MSL1 | CHST15 | 0.023071583 |
| blue | MSL1 | CCNJL | 0.022880019 |
| blue | MSL1 | CANT1 | 0.028460625 |
| blue | MSL1 | C5AR1 | 0.029564502 |
| blue | MSL1 | C15orf39 | 0.02353656 |
| blue | MSL1 | BCL3 | 0.02501241 |
| blue | MSL1 | BAZ2B | 0.028934904 |
| blue | MSL1 | BASP1 | 0.030606211 |
| blue | MSL1 | ARID3A | 0.029588504 |
| blue | MSL1 | ARAP1 | 0.039516763 |
| blue | MSL1 | ANPEP | 0.035076036 |
| blue | MSL1 | ANKRD13A | 0.02422782 |
| blue | MSL1 | AGTPBP1 | 0.024282328 |

| blue | MSL1 | ADAM8 | 0.026837767 |
| --- | --- | --- | --- |
| blue | MOSPD2 | GAB2 | 0.031468928 |
| blue | MOSPD2 | CHST15 | 0.02084079 |
| blue | MNDA | GCA | 0.03000326 |
| blue | MNDA | FBXL5 | 0.033489259 |
| blue | MNDA | CEBPB | 0.02654986 |
| blue | MNDA | BCL6 | 0.025417513 |
| blue | MNDA | AQP9 | 0.041612837 |
| blue | MNDA | ACSL1 | 0.026374495 |
| blue | MMP9 | MME | 0.02644197 |
| blue | MMP25 | LRG1 | 0.027265644 |
| blue | MMP25 | ITPRIP | 0.037266976 |
| blue | MMP25 | IMPDH1 | 0.023300922 |
| blue | MMP25 | GCA | 0.020101018 |
| blue | MMP25 | GAB2 | 0.024449894 |
| blue | MMP25 | CSF3R | 0.042452216 |
| blue | MMP25 | CEBPB | 0.03443172 |
| blue | MMP25 | CEACAM3 | 0.030927631 |
| blue | MMP25 | BCL6 | 0.051653077 |
| blue | MMP25 | AQP9 | 0.053675656 |
| blue | MMP25 | ALOX5 | 0.021564401 |
| blue | MMP25 | ACSL1 | 0.031410489 |
| blue | MMP25 | ABTB1 | 0.029662639 |
| blue | MME | MGAM2 | 0.033998809 |
| blue | MME | MGAM | 0.04841525 |
| blue | MME | MEGF9 | 0.022449112 |
| blue | MME | MBOAT7 | 0.020126035 |
| blue | MME | LITAF | 0.021212925 |
| blue | MME | LCP1 | 0.023367235 |
| blue | MME | LAMP2 | 0.020223468 |
| blue | MME | KIF1B | 0.021503213 |
| blue | MME | IL6R | 0.025031241 |
| blue | MME | IGF2R | 0.029930293 |
| blue | MME | IFRD1 | 0.025503378 |
| blue | MME | ICAM3 | 0.031938385 |
| blue | MME | HSDL2 | 0.020319865 |
| blue | MME | GPAT3 | 0.021034103 |
| blue | MME | GCA | 0.022489058 |
| blue | MME | GAB2 | 0.044689147 |
| blue | MME | FNIP1 | 0.021960717 |
| blue | MME | DYSF | 0.036312643 |
| blue | MME | CYP4F3 | 0.022718306 |
| blue | MME | CYB5R4 | 0.021631828 |
| blue | MME | CSF3R | 0.023831429 |
| blue | MME | CPD | 0.029225616 |
| blue | MME | COP1 | 0.023300395 |
| blue | MME | CMTM2 | 0.030780756 |
| blue | MME | CHST15 | 0.029492679 |
| blue | MME | CDA | 0.024669121 |
| blue | MME | C3orf62 | 0.021090234 |
| blue | MME | BCL6 | 0.029241627 |
| blue | MME | BASP1 | 0.024677218 |

| blue | MME | ARID3A | 0.029313543 |
| --- | --- | --- | --- |
| blue | MME | AQP9 | 0.030325796 |
| blue | MME | ALOX5AP | 0.022101137 |
| blue | MME | ADGRE3 | 0.026247639 |
| blue | MME | ACSL1 | 0.024165945 |
| blue | MIR223 | IL1R2 | 0.02030899 |
| blue | MIR223 | GAB2 | 0.020909231 |
| blue | MIR223 | CSF3R | 0.02530583 |
| blue | MIR223 | CEBPB | 0.024147222 |
| blue | MIR223 | AQP9 | 0.023650712 |
| blue | MIDN | GAB2 | 0.02393707 |
| blue | MIDN | AQP9 | 0.022168985 |
| blue | MGAM2 | MGAM | 0.088289017 |
| blue | MGAM2 | KLHL2 | 0.022601888 |
| blue | MGAM2 | GCA | 0.021437786 |
| blue | MGAM2 | GAB2 | 0.031593205 |
| blue | MGAM2 | F5 | 0.021043985 |
| blue | MGAM2 | DYSF | 0.032523429 |
| blue | MGAM2 | CYSTM1 | 0.022795691 |
| blue | MGAM2 | CHST15 | 0.022977295 |
| blue | MGAM2 | BCL6 | 0.051336519 |
| blue | MGAM2 | BASP1 | 0.023963532 |
| blue | MGAM2 | AQP9 | 0.03830545 |
| blue | MGAM2 | APMAP | 0.023831307 |
| blue | MGAM2 | ACSL1 | 0.04284458 |
| blue | MGAM | LMNB1 | 0.021092013 |
| blue | MGAM | KLHL2 | 0.020947635 |
| blue | MGAM | KCNJ15 | 0.024885694 |
| blue | MGAM | IQSEC1 | 0.025678157 |
| blue | MGAM | IL6R | 0.027820765 |
| blue | MGAM | IGF2R | 0.027892072 |
| blue | MGAM | IFRD1 | 0.022739816 |
| blue | MGAM | GCA | 0.024033244 |
| blue | MGAM | GAB2 | 0.040978815 |
| blue | MGAM | FRAT1 | 0.023658053 |
| blue | MGAM | FNIP1 | 0.021061779 |
| blue | MGAM | FLOT2 | 0.023280283 |
| blue | MGAM | FAM160B1 | 0.027100794 |
| blue | MGAM | F5 | 0.041737915 |
| blue | MGAM | EXOC6 | 0.026945974 |
| blue | MGAM | ETS2 | 0.02298098 |
| blue | MGAM | ELL | 0.026480289 |
| blue | MGAM | DYSF | 0.042297055 |
| blue | MGAM | DENND5A | 0.023273977 |
| blue | MGAM | CYSTM1 | 0.02594163 |
| blue | MGAM | CTBS | 0.021205103 |
| blue | MGAM | CSF3R | 0.024955636 |
| blue | MGAM | CPD | 0.023394768 |
| blue | MGAM | COP1 | 0.026482651 |
| blue | MGAM | CMTM2 | 0.02383213 |
| blue | MGAM | CHST15 | 0.031751984 |
| blue | MGAM | CEBPB | 0.021463427 |

| blue | MGAM | CDA | 0.027464857 |
| --- | --- | --- | --- |
| blue | MGAM | CCNJL | 0.024779496 |
| blue | MGAM | C3orf62 | 0.021904174 |
| blue | MGAM | BCL6 | 0.055877576 |
| blue | MGAM | BASP1 | 0.028193232 |
| blue | MGAM | B4GALT5 | 0.024743796 |
| blue | MGAM | ARID3A | 0.022950469 |
| blue | MGAM | AQP9 | 0.045147109 |
| blue | MGAM | APMAP | 0.026003188 |
| blue | MGAM | AGO4 | 0.022626289 |
| blue | MGAM | ADGRG3 | 0.021621883 |
| blue | MGAM | ADGRE3 | 0.022022446 |
| blue | MGAM | ACSL1 | 0.042920367 |
| blue | MEGF9 | LRRK2 | 0.021094714 |
| blue | MEGF9 | KLHL2 | 0.0262197 |
| blue | MEGF9 | KIAA0319L | 0.021519755 |
| blue | MEGF9 | IRS2 | 0.023430008 |
| blue | MEGF9 | HECW2 | 0.026517663 |
| blue | MEGF9 | GCA | 0.030544622 |
| blue | MEGF9 | GAB2 | 0.030748949 |
| blue | MEGF9 | FRAT1 | 0.02340992 |
| blue | MEGF9 | FAM49B | 0.021882328 |
| blue | MEGF9 | EGLN1 | 0.021388924 |
| blue | MEGF9 | CYB5R4 | 0.022886064 |
| blue | MEGF9 | CPSF4 | 0.032118198 |
| blue | MEGF9 | CMTM6 | 0.022676028 |
| blue | MEGF9 | CHIC2 | 0.0227114 |
| blue | MEGF9 | CEBPD | 0.022868004 |
| blue | MEGF9 | BCL6 | 0.025006439 |
| blue | MEGF9 | BCL2A1 | 0.023477572 |
| blue | MEGF9 | BASP1 | 0.021740343 |
| blue | MEGF9 | AQP9 | 0.02886641 |
| blue | MEGF9 | ACSL4 | 0.021008061 |
| blue | MEGF9 | ACSL1 | 0.024823934 |
| blue | MEGF9 | ACOX1 | 0.020948157 |
| blue | MEFV | IQGAP1 | 0.022962931 |
| blue | MEFV | CANT1 | 0.020464875 |
| blue | MEFV | C5AR1 | 0.020603828 |
| blue | MEFV | C16orf58 | 0.022579493 |
| blue | MEFV | ARID3A | 0.021142905 |
| blue | MED13L | ARAP1 | 0.02307455 |
| blue | MCL1 | GAB2 | 0.037750574 |
| blue | MCL1 | CSF3R | 0.022386411 |
| blue | MCEMP1 | FLOT2 | 0.021359358 |
| blue | MCEMP1 | BCL6 | 0.023513996 |
| blue | MCEMP1 | ACSL1 | 0.033352841 |
| blue | MBOAT7 | MAST3 | 0.022807037 |
| blue | MBOAT7 | MAP3K11 | 0.024599148 |
| blue | MBOAT7 | LYN | 0.027313834 |
| blue | MBOAT7 | LITAF | 0.026720867 |
| blue | MBOAT7 | LCP1 | 0.02056879 |
| blue | MBOAT7 | LAT2 | 0.024948999 |

| blue | MBOAT7 | ITPK1 | 0.022982443 |
| --- | --- | --- | --- |
| blue | MBOAT7 | IQSEC1 | 0.022247118 |
| blue | MBOAT7 | IL17RA | 0.027142331 |
| blue | MBOAT7 | ICAM3 | 0.025626589 |
| blue | MBOAT7 | HSDL2 | 0.02314094 |
| blue | MBOAT7 | HLX | 0.020686514 |
| blue | MBOAT7 | HCK | 0.027624473 |
| blue | MBOAT7 | GLT1D1 | 0.021364551 |
| blue | MBOAT7 | GCA | 0.020487575 |
| blue | MBOAT7 | GAB2 | 0.041463144 |
| blue | MBOAT7 | FPR1 | 0.020050152 |
| blue | MBOAT7 | FCGRT | 0.020869895 |
| blue | MBOAT7 | DYSF | 0.027069955 |
| blue | MBOAT7 | CXCR1 | 0.023937391 |
| blue | MBOAT7 | CSF3R | 0.020036061 |
| blue | MBOAT7 | CANT1 | 0.027818969 |
| blue | MBOAT7 | C5AR1 | 0.028571593 |
| blue | MBOAT7 | C15orf39 | 0.021356374 |
| blue | MBOAT7 | BCL3 | 0.020928863 |
| blue | MBOAT7 | BASP1 | 0.027671321 |
| blue | MBOAT7 | ATP6V1B2 | 0.02004142 |
| blue | MBOAT7 | ARID3A | 0.022434497 |
| blue | MBOAT7 | ARHGAP25 | 0.025349004 |
| blue | MBOAT7 | ARAP1 | 0.031876094 |
| blue | MBOAT7 | ADAM8 | 0.023317797 |
| blue | MAZ | GAB2 | 0.020988173 |
| blue | MAST3 | IQGAP1 | 0.022678028 |
| blue | MAST3 | GAB2 | 0.025342974 |
| blue | MARCKS | BCL6 | 0.032557134 |
| blue | MARCKS | BCL3 | 0.020447054 |
| blue | MARCKS | BASP1 | 0.024391494 |
| blue | MARCKS | AQP9 | 0.02494557 |
| blue | MARCKS | ACSL1 | 0.02911535 |
| blue | 1-Mar | KLHL2 | 0.022825088 |
| blue | 1-Mar | GCA | 0.021884486 |
| blue | 1-Mar | GAB2 | 0.025060495 |
| blue | 1-Mar | CEBPB | 0.02203907 |
| blue | 1-Mar | AQP9 | 0.023171758 |
| blue | MAPK1 | CEBPB | 0.033754665 |
| blue | MAPK1 | BCL6 | 0.021663127 |
| blue | MAPK1 | AQP9 | 0.030595281 |
| blue | MAP4K1 | GAB2 | 0.031722196 |
| blue | MAP3K5 | LAT2 | 0.025301145 |
| blue | MAP3K5 | GAB2 | 0.021453868 |
| blue | MAP3K5 | DYSF | 0.021346169 |
| blue | MAP3K5 | ARID3A | 0.023648953 |
| blue | MAP3K11 | C5AR1 | 0.024239441 |
| blue | MAP3K11 | ARAP1 | 0.023032166 |
| blue | MAP2K4 | GPSM3 | 0.02000219 |
| blue | MAP2K4 | GMIP | 0.020212057 |
| blue | MAP2K4 | DPP7 | 0.021358515 |
| blue | MAP2K4 | CUX1 | 0.03215751 |

| blue | MAP2K4 | CBL | 0.037260891 |
| --- | --- | --- | --- |
| blue | MAP2K4 | C5AR1 | 0.036102603 |
| blue | MAP2K4 | APBB1IP | 0.020551358 |
| blue | MANSC1 | KLHL2 | 0.024339853 |
| blue | MANSC1 | IL1R2 | 0.020897023 |
| blue | MANSC1 | GCA | 0.026161249 |
| blue | MANSC1 | EVI2B | 0.023884979 |
| blue | MANSC1 | CSF3R | 0.021736209 |
| blue | MANSC1 | CMTM2 | 0.022258401 |
| blue | MANSC1 | CEBPB | 0.027982509 |
| blue | MANSC1 | BCL6 | 0.027000618 |
| blue | MANSC1 | AQP9 | 0.043468309 |
| blue | MANSC1 | ACSL1 | 0.023443866 |
| blue | MAEA | BCL6 | 0.036152887 |
| blue | MAEA | AQP9 | 0.035171185 |
| blue | MAEA | ACSL1 | 0.028311998 |
| blue | LYST | LAT2 | 0.025506888 |
| blue | LYST | CUX1 | 0.025434924 |
| blue | LYST | CBL | 0.026337937 |
| blue | LYST | BAZ2B | 0.023685559 |
| blue | LYST | ARAP1 | 0.020328754 |
| blue | LYST | ADAM8 | 0.02372034 |
| blue | LYN | LRRK2 | 0.022823581 |
| blue | LYN | LITAF | 0.051912401 |
| blue | LYN | LCP1 | 0.025971543 |
| blue | LYN | JUNB | 0.021083085 |
| blue | LYN | ITPR3 | 0.025596352 |
| blue | LYN | ITM2B | 0.048728059 |
| blue | LYN | IL1RN | 0.030898283 |
| blue | LYN | IFNGR2 | 0.030114945 |
| blue | LYN | HLX | 0.024803999 |
| blue | LYN | HCK | 0.034106052 |
| blue | LYN | GCA | 0.044426769 |
| blue | LYN | GAB2 | 0.039754967 |
| blue | LYN | FPR1 | 0.028019552 |
| blue | LYN | FNDC3B | 0.02815322 |
| blue | LYN | FCGR2A | 0.020664099 |
| blue | LYN | FAM8A1 | 0.021282668 |
| blue | LYN | EGLN1 | 0.022110614 |
| blue | LYN | DYSF | 0.02518787 |
| blue | LYN | CYB5R4 | 0.02672389 |
| blue | LYN | CSF3R | 0.022344798 |
| blue | LYN | CMTM6 | 0.029791006 |
| blue | LYN | CAP1 | 0.02149599 |
| blue | LYN | C5AR1 | 0.02616317 |
| blue | LYN | C15orf39 | 0.03118898 |
| blue | LYN | BCL6 | 0.020655545 |
| blue | LYN | BCL3 | 0.042094484 |
| blue | LYN | BASP1 | 0.040674868 |
| blue | LYN | ATP6V1B2 | 0.035822656 |
| blue | LYN | ATP6V1A | 0.020838623 |
| blue | LYN | AQP9 | 0.030927628 |

| blue | LYN | AKIRIN2 | 0.027785643 |
| --- | --- | --- | --- |
| blue | LYN | ACSL1 | 0.020718481 |
| blue | LYN | ACOX1 | 0.025650284 |
| blue | LY96 | GCA | 0.033798922 |
| blue | LY96 | GAB2 | 0.0300444 |
| blue | LY96 | CSF3R | 0.02439559 |
| blue | LY96 | CEBPB | 0.029418518 |
| blue | LY96 | BCL6 | 0.035493623 |
| blue | LY96 | AQP9 | 0.055260586 |
| blue | LY96 | ACSL1 | 0.030384251 |
| blue | LTBR | HCK | 0.023921545 |
| blue | LRRK2 | LRMP | 0.023182203 |
| blue | LRRK2 | HLX | 0.026437344 |
| blue | LRRK2 | GCA | 0.027935665 |
| blue | LRRK2 | GAB2 | 0.035022868 |
| blue | LRRK2 | EGLN1 | 0.021444816 |
| blue | LRRK2 | DYSF | 0.022655155 |
| blue | LRRK2 | DENND5A | 0.021179766 |
| blue | LRRK2 | CYB5R4 | 0.0234247 |
| blue | LRRK2 | CXCR1 | 0.02161051 |
| blue | LRRK2 | CSF3R | 0.023073477 |
| blue | LRRK2 | CMTM6 | 0.023685054 |
| blue | LRRK2 | BCL6 | 0.021046183 |
| blue | LRRK2 | BASP1 | 0.020067076 |
| blue | LRRK2 | AQP9 | 0.023389447 |
| blue | LRRK2 | ACOX1 | 0.025585237 |
| blue | LRP10 | ITPRIP | 0.022414792 |
| blue | LRP10 | GAB2 | 0.029240122 |
| blue | LRP10 | CXCR1 | 0.020427942 |
| blue | LRP10 | CSF3R | 0.035671233 |
| blue | LRP10 | BCL6 | 0.020210788 |
| blue | LRP10 | AQP9 | 0.029896845 |
| blue | LRMP | GCA | 0.02196295 |
| blue | LRMP | GAB2 | 0.031207761 |
| blue | LRMP | BCL6 | 0.020692649 |
| blue | LRG1 | ITPRIP | 0.039876168 |
| blue | LRG1 | IMPDH1 | 0.020680833 |
| blue | LRG1 | IFNGR1 | 0.024039115 |
| blue | LRG1 | GCA | 0.024456235 |
| blue | LRG1 | GAB2 | 0.026664399 |
| blue | LRG1 | FPR1 | 0.022756606 |
| blue | LRG1 | CXCR1 | 0.023775561 |
| blue | LRG1 | CSF3R | 0.047799621 |
| blue | LRG1 | CEBPB | 0.056193118 |
| blue | LRG1 | CEACAM3 | 0.024337027 |
| blue | LRG1 | CA4 | 0.020613897 |
| blue | LRG1 | BCL6 | 0.036718028 |
| blue | LRG1 | AQP9 | 0.065778584 |
| blue | LRG1 | ALOX5 | 0.021016132 |
| blue | LRG1 | ACSL1 | 0.026943896 |
| blue | LPGAT1 | CHST15 | 0.026696843 |
| blue | LPGAT1 | AQP9 | 0.021107686 |

| blue | LMNB1 | KLHL2 | 0.021525722 |
| --- | --- | --- | --- |
| blue | LMNB1 | GCA | 0.030958484 |
| blue | LMNB1 | FLOT2 | 0.039847558 |
| blue | LMNB1 | FBXL5 | 0.025334052 |
| blue | LMNB1 | CEBPB | 0.026415637 |
| blue | LMNB1 | BCL6 | 0.063405647 |
| blue | LMNB1 | BASP1 | 0.020637994 |
| blue | LMNB1 | AQP9 | 0.059838016 |
| blue | LMNB1 | ANXA3 | 0.034598465 |
| blue | LMNB1 | ACSL1 | 0.070342459 |
| blue | LITAF | ITM2B | 0.029846996 |
| blue | LITAF | GCA | 0.039956454 |
| blue | LITAF | GAB2 | 0.03653105 |
| blue | LITAF | DYSF | 0.02893983 |
| blue | LITAF | CYB5R4 | 0.025269845 |
| blue | LITAF | CSF3R | 0.020464685 |
| blue | LITAF | C5AR1 | 0.027225037 |
| blue | LITAF | BCL6 | 0.020563591 |
| blue | LITAF | BCL3 | 0.03319971 |
| blue | LITAF | BASP1 | 0.046671628 |
| blue | LITAF | ATP6V1B2 | 0.020272297 |
| blue | LITAF | AQP9 | 0.030077136 |
| blue | LITAF | ACSL1 | 0.0216902 |
| blue | LILRA2 | ITPRIP | 0.027766112 |
| blue | LILRA2 | CSF3R | 0.022274198 |
| blue | LILRA2 | CEBPB | 0.024558926 |
| blue | LILRA2 | AQP9 | 0.026763092 |
| blue | LILRA2 | ALOX5 | 0.022225439 |
| blue | LCP1 | GCA | 0.021335551 |
| blue | LCP1 | GAB2 | 0.024257154 |
| blue | LCP1 | CAP1 | 0.030287311 |
| blue | LCP1 | BASP1 | 0.027302459 |
| blue | LCP1 | ATP6V1B2 | 0.030572076 |
| blue | LAT2 | IL17RA | 0.024906404 |
| blue | LAT2 | GPAT3 | 0.025449926 |
| blue | LAT2 | GAB2 | 0.024557939 |
| blue | LAT2 | DYSF | 0.026516328 |
| blue | LAT2 | CUX1 | 0.021425977 |
| blue | LAT2 | C15orf39 | 0.022127894 |
| blue | LAT2 | BRI3 | 0.021132245 |
| blue | LAT2 | BASP1 | 0.021397853 |
| blue | LAT2 | ARID3A | 0.035353192 |
| blue | LAT2 | ARAP1 | 0.024997218 |
| blue | LAT2 | ANPEP | 0.023005929 |
| blue | LAMP2 | ICAM3 | 0.052564566 |
| blue | LAMP2 | GAB2 | 0.027185224 |
| blue | LAMP2 | CMTM2 | 0.021179307 |
| blue | LAMP2 | CHST15 | 0.022079337 |
| blue | LAMP2 | ATP6V1B2 | 0.023321244 |
| blue | KLHL2 | IRS2 | 0.021674269 |
| blue | KLHL2 | IRAK3 | 0.033177198 |
| blue | KLHL2 | IL1R2 | 0.034943208 |

| blue | KLHL2 | IFNGR1 | 0.026556394 |
| --- | --- | --- | --- |
| blue | KLHL2 | HECW2 | 0.02037491 |
| blue | KLHL2 | HAL | 0.021659771 |
| blue | KLHL2 | GCA | 0.034383911 |
| blue | KLHL2 | GAB2 | 0.029440853 |
| blue | KLHL2 | FRAT1 | 0.025998623 |
| blue | KLHL2 | FLOT2 | 0.023608498 |
| blue | KLHL2 | EXOC6 | 0.028741234 |
| blue | KLHL2 | ETS2 | 0.025013503 |
| blue | KLHL2 | CSF3R | 0.02601088 |
| blue | KLHL2 | CELF2 | 0.026930828 |
| blue | KLHL2 | CEBPB | 0.031549078 |
| blue | KLHL2 | C1RL | 0.023353146 |
| blue | KLHL2 | BCL6 | 0.036452601 |
| blue | KLHL2 | BCL2A1 | 0.030480592 |
| blue | KLHL2 | AQP9 | 0.039424473 |
| blue | KLHL2 | ANKS1A | 0.025683647 |
| blue | KLHL2 | ACSL1 | 0.037861638 |
| blue | KLHL2 | ACOX1 | 0.023588284 |
| blue | KIF1B | DYSF | 0.025920939 |
| blue | KIF1B | CUX1 | 0.028969805 |
| blue | KIF1B | BASP1 | 0.020027516 |
| blue | KIAA0513 | GAB2 | 0.028234789 |
| blue | KIAA0513 | ARID3A | 0.022481337 |
| blue | KIAA0319L | CPSF4 | 0.028948854 |
| blue | KIAA0319L | CEBPD | 0.023476226 |
| blue | KIAA0319L | BMS1 | 0.03134129 |
| blue | KCNJ2 | FCGR2A | 0.021938934 |
| blue | KCNJ2 | CXCR1 | 0.023781787 |
| blue | KCNJ2 | AQP9 | 0.020044284 |
| blue | KCNJ2 | ALPK1 | 0.027181252 |
| blue | KCNJ15 | GCA | 0.030067881 |
| blue | KCNJ15 | GAB2 | 0.026453679 |
| blue | KCNJ15 | FLOT2 | 0.022028763 |
| blue | KCNJ15 | FCGR2A | 0.022066114 |
| blue | KCNJ15 | DYSF | 0.033083966 |
| blue | KCNJ15 | CSF3R | 0.02297129 |
| blue | KCNJ15 | BCL6 | 0.051045366 |
| blue | KCNJ15 | BCL3 | 0.020893792 |
| blue | KCNJ15 | BASP1 | 0.029101451 |
| blue | KCNJ15 | AQP9 | 0.041633777 |
| blue | KCNJ15 | ANXA3 | 0.042765689 |
| blue | KCNJ15 | ALPK1 | 0.021900005 |
| blue | KCNJ15 | ACSL1 | 0.049219801 |
| blue | KBTBD7 | GAB2 | 0.025329465 |
| blue | KBTBD7 | FRAT1 | 0.028719009 |
| blue | JUNB | BASP1 | 0.024776933 |
| blue | JAML | GAB2 | 0.020602542 |
| blue | JAML | ARAP1 | 0.021034996 |
| blue | ITPRIP | IMPDH1 | 0.02356348 |
| blue | ITPRIP | IGF2R | 0.025253266 |
| blue | ITPRIP | IFNGR1 | 0.023295022 |

| blue | ITPRIP | IFITM2 | 0.021934014 |
| --- | --- | --- | --- |
| blue | ITPRIP | HSPA1A | 0.020458314 |
| blue | ITPRIP | GCA | 0.029555849 |
| blue | ITPRIP | GAB2 | 0.042604386 |
| blue | ITPRIP | FPR1 | 0.028622379 |
| blue | ITPRIP | FGR | 0.021274764 |
| blue | ITPRIP | FCGR2A | 0.023910586 |
| blue | ITPRIP | FAM49A | 0.020180679 |
| blue | ITPRIP | EXOC6 | 0.023218713 |
| blue | ITPRIP | EVI2B | 0.021334805 |
| blue | ITPRIP | CXCR1 | 0.033496271 |
| blue | ITPRIP | CSF3R | 0.060304197 |
| blue | ITPRIP | CPD | 0.022799194 |
| blue | ITPRIP | COP1 | 0.028247445 |
| blue | ITPRIP | CMTM2 | 0.023961467 |
| blue | ITPRIP | CHST15 | 0.02194125 |
| blue | ITPRIP | CEBPB | 0.041939368 |
| blue | ITPRIP | CEACAM3 | 0.022814118 |
| blue | ITPRIP | CA4 | 0.023945753 |
| blue | ITPRIP | BCL6 | 0.037042237 |
| blue | ITPRIP | ATP6V1B2 | 0.022240536 |
| blue | ITPRIP | ATP6V0B | 0.027922607 |
| blue | ITPRIP | AQP9 | 0.063359664 |
| blue | ITPRIP | ALOX5 | 0.021546583 |
| blue | ITPRIP | ADGRE5 | 0.027839813 |
| blue | ITPRIP | ACSL1 | 0.02470688 |
| blue | ITPRIP | ABTB1 | 0.031415313 |
| blue | ITM2B | GCA | 0.03102138 |
| blue | ITM2B | FOS | 0.023116049 |
| blue | ITM2B | CYB5R4 | 0.021348262 |
| blue | ITM2B | CPSF4 | 0.021262948 |
| blue | ITM2B | CMTM6 | 0.021509367 |
| blue | ITM2B | CD58 | 0.034453986 |
| blue | ITM2B | C5AR1 | 0.023985001 |
| blue | ITM2B | BASP1 | 0.028313912 |
| blue | ITM2B | AKIRIN2 | 0.023413664 |
| blue | ITGAM | DYSF | 0.024950893 |
| blue | ITGAM | CUX1 | 0.021402037 |
| blue | ITGAM | BASP1 | 0.021123201 |
| blue | ITGAM | ACSL4 | 0.020814945 |
| blue | IRS2 | IL1R2 | 0.022980612 |
| blue | IRS2 | GAB2 | 0.020859009 |
| blue | IRS2 | FRAT1 | 0.02586844 |
| blue | IRS2 | CEBPD | 0.027673087 |
| blue | IRAK3 | CEBPB | 0.024599508 |
| blue | IQSEC1 | IQGAP1 | 0.022498159 |
| blue | IQSEC1 | GAB2 | 0.023878499 |
| blue | IQSEC1 | DYSF | 0.042803901 |
| blue | IQSEC1 | CANT1 | 0.020198256 |
| blue | IQSEC1 | BASP1 | 0.026108786 |
| blue | IQSEC1 | ARAP1 | 0.028995033 |
| blue | IQGAP1 | ICAM3 | 0.020192131 |

| blue | IQGAP1 | EXTL3 | 0.022563443 |
| --- | --- | --- | --- |
| blue | IQGAP1 | DCP2 | 0.022434878 |
| blue | IQGAP1 | CANT1 | 0.02291087 |
| blue | IQGAP1 | C5AR1 | 0.027254647 |
| blue | INPP5A | CUX1 | 0.028741946 |
| blue | IMPDH1 | CSF3R | 0.021980285 |
| blue | IMPDH1 | CEBPB | 0.025559916 |
| blue | IMPDH1 | CEACAM3 | 0.023422016 |
| blue | IMPDH1 | AQP9 | 0.026753555 |
| blue | IMPDH1 | ALOX5 | 0.02310941 |
| blue | IL6R | GAB2 | 0.028610401 |
| blue | IL6R | CSF3R | 0.021367178 |
| blue | IL6R | BCL6 | 0.022663959 |
| blue | IL6R | AQP9 | 0.026640199 |
| blue | IL1RN | HCK | 0.020854983 |
| blue | IL1RN | GCA | 0.020541894 |
| blue | IL1RN | DYSF | 0.020689312 |
| blue | IL1RN | BCL3 | 0.022159801 |
| blue | IL1RN | BASP1 | 0.031327217 |
| blue | IL1RN | AQP9 | 0.020555234 |
| blue | IL1RN | ACSL1 | 0.020770876 |
| blue | IL1RAP | GAB2 | 0.02425839 |
| blue | IL1RAP | CSF3R | 0.021917768 |
| blue | IL1R2 | IFNGR1 | 0.020590809 |
| blue | IL1R2 | CELF2 | 0.036887151 |
| blue | IL1R2 | CEBPB | 0.025091929 |
| blue | IL1R2 | ANKS1A | 0.032423837 |
| blue | IL17RA | GAB2 | 0.020452099 |
| blue | IL17RA | DYSF | 0.02128694 |
| blue | IL17RA | ARID3A | 0.02115492 |
| blue | IL17RA | ARAP1 | 0.029105699 |
| blue | IGF2R | HSPA1A | 0.020449008 |
| blue | IGF2R | HMGCR | 0.027578469 |
| blue | IGF2R | GCA | 0.020239656 |
| blue | IGF2R | GAB2 | 0.038532797 |
| blue | IGF2R | CSF3R | 0.031727196 |
| blue | IGF2R | CPD | 0.023962609 |
| blue | IGF2R | COP1 | 0.033032219 |
| blue | IGF2R | CMTM2 | 0.029766328 |
| blue | IGF2R | CHST15 | 0.049001418 |
| blue | IGF2R | CEBPB | 0.023735287 |
| blue | IGF2R | BTBD10 | 0.02307379 |
| blue | IGF2R | BCL6 | 0.025342793 |
| blue | IGF2R | ATP6V1B2 | 0.02406718 |
| blue | IGF2R | ATP6V0B | 0.024724687 |
| blue | IGF2R | ASPRV1 | 0.022794138 |
| blue | IGF2R | AQP9 | 0.037322969 |
| blue | IGF2R | ADGRE3 | 0.032097503 |
| blue | IFRD1 | HSDL2 | 0.021848611 |
| blue | IFRD1 | GAB2 | 0.031790505 |
| blue | IFRD1 | CHST15 | 0.023453261 |
| blue | IFRD1 | AQP9 | 0.022595022 |

| blue | IFNGR2 | GCA | 0.035215703 |
| --- | --- | --- | --- |
| blue | IFNGR2 | GAB2 | 0.030019449 |
| blue | IFNGR2 | FRAT2 | 0.048178585 |
| blue | IFNGR2 | FPR1 | 0.020974671 |
| blue | IFNGR2 | CSF3R | 0.023237926 |
| blue | IFNGR2 | CEBPB | 0.029165225 |
| blue | IFNGR2 | BCL6 | 0.025832183 |
| blue | IFNGR2 | BASP1 | 0.022566976 |
| blue | IFNGR2 | ATP6V1B2 | 0.043139796 |
| blue | IFNGR2 | AQP9 | 0.036889702 |
| blue | IFNGR2 | ACSL1 | 0.026888031 |
| blue | IFNGR1 | GCA | 0.031166754 |
| blue | IFNGR1 | GAB2 | 0.024986046 |
| blue | IFNGR1 | CSF3R | 0.031288625 |
| blue | IFNGR1 | CELF2 | 0.023199109 |
| blue | IFNGR1 | CEBPB | 0.075518903 |
| blue | IFNGR1 | BCL6 | 0.026791868 |
| blue | IFNGR1 | AQP9 | 0.049544631 |
| blue | IFNGR1 | ACSL1 | 0.021833807 |
| blue | IFNAR1 | AQP9 | 0.025472224 |
| blue | IFITM2 | GCA | 0.02848803 |
| blue | IFITM2 | GAB2 | 0.032866782 |
| blue | IFITM2 | FPR1 | 0.022481727 |
| blue | IFITM2 | CXCR1 | 0.024659263 |
| blue | IFITM2 | CSF3R | 0.035721439 |
| blue | IFITM2 | CEBPB | 0.024344365 |
| blue | IFITM2 | BCL6 | 0.026503218 |
| blue | IFITM2 | AQP9 | 0.047575236 |
| blue | IFITM2 | ACSL1 | 0.024439335 |
| blue | ICAM3 | HSDL2 | 0.03601684 |
| blue | ICAM3 | GAB2 | 0.031031755 |
| blue | ICAM3 | FCGRT | 0.033003787 |
| blue | ICAM3 | EXTL3 | 0.031658677 |
| blue | ICAM3 | CHST15 | 0.023147138 |
| blue | ICAM3 | C5AR1 | 0.025191991 |
| blue | ICAM3 | ATP6V1B2 | 0.020548583 |
| blue | ICAM3 | ARID3A | 0.026151114 |
| blue | ICAM3 | ADGRE3 | 0.020349872 |
| blue | HSPA1A | GCA | 0.029938444 |
| blue | HSPA1A | GAB2 | 0.030607802 |
| blue | HSPA1A | CSF3R | 0.025044184 |
| blue | HSPA1A | CHST15 | 0.02165097 |
| blue | HSPA1A | CEBPB | 0.028922562 |
| blue | HSPA1A | BCL6 | 0.025325361 |
| blue | HSPA1A | ATP6V1B2 | 0.028013334 |
| blue | HSPA1A | AQP9 | 0.038012212 |
| blue | HSPA1A | ACSL1 | 0.022252546 |
| blue | HSDL2 | BASP1 | 0.020086156 |
| blue | HSD17B11 | CYB5R4 | 0.020275995 |
| blue | HMGCR | CMTM2 | 0.021371919 |
| blue | HMGCR | CHST15 | 0.020076511 |
| blue | HMGCR | AQP9 | 0.020252737 |

| blue | HLX | GCA | 0.026001007 |
| --- | --- | --- | --- |
| blue | HLX | GAB2 | 0.035998816 |
| blue | HLX | FNDC3B | 0.020368583 |
| blue | HLX | CXCR1 | 0.021827861 |
| blue | HLX | CSF3R | 0.021889708 |
| blue | HLX | ATP6V1B2 | 0.026673011 |
| blue | HLX | AQP9 | 0.021459208 |
| blue | HIST2H2AC | HIST1H2BE | 0.021984088 |
| blue | HIST2H2AC | HIST1H2BC | 0.024392759 |
| blue | HIST2H2AC | H2AFJ | 0.115318374 |
| blue | HIST2H2AC | GCA | 0.022987091 |
| blue | HIST2H2AC | CEBPB | 0.030638662 |
| blue | HIST2H2AC | BCL6 | 0.028571765 |
| blue | HIST2H2AC | AQP9 | 0.038076351 |
| blue | HIST2H2AC | ACSL1 | 0.024292883 |
| blue | HIST1H2BE | HIST1H2BC | 0.065486828 |
| blue | HIST1H2BE | GCA | 0.02063134 |
| blue | HIST1H2BE | AQP9 | 0.021706462 |
| blue | HIST1H2BC | GCA | 0.022663797 |
| blue | HIST1H2BC | GAB2 | 0.021461227 |
| blue | HIST1H2BC | CSF3R | 0.026622893 |
| blue | HIST1H2BC | CEBPB | 0.023501039 |
| blue | HIST1H2BC | BCL6 | 0.020438142 |
| blue | HIST1H2BC | AQP9 | 0.036183801 |
| blue | HCK | GCA | 0.035387426 |
| blue | HCK | GAB2 | 0.033994346 |
| blue | HCK | DYSF | 0.032997922 |
| blue | HCK | DENND5A | 0.024195826 |
| blue | HCK | CXCR1 | 0.020021421 |
| blue | HCK | CSF3R | 0.021536705 |
| blue | HCK | BCL6 | 0.029277732 |
| blue | HCK | BCL3 | 0.028679751 |
| blue | HCK | BASP1 | 0.039689902 |
| blue | HCK | ARAP1 | 0.022265054 |
| blue | HCK | AQP9 | 0.033122573 |
| blue | HCK | ACSL1 | 0.029395767 |
| blue | HAL | GAB2 | 0.026956263 |
| blue | HAL | CSF3R | 0.034581025 |
| blue | HAL | CHST15 | 0.022695718 |
| blue | HAL | CEBPB | 0.022275195 |
| blue | HAL | BCL6 | 0.020205684 |
| blue | HAL | AQP9 | 0.028775178 |
| blue | H2AFJ | AQP9 | 0.021353028 |
| blue | GPAT3 | ARID3A | 0.044761324 |
| blue | GNB2 | C5AR1 | 0.027955186 |
| blue | GMIP | CUX1 | 0.025163388 |
| blue | GK | GCA | 0.020804885 |
| blue | GK | FCGR2A | 0.055641682 |
| blue | GK | CXCR1 | 0.024846782 |
| blue | GK | CSF3R | 0.021340401 |
| blue | GK | BCL6 | 0.030274878 |
| blue | GK | AQP9 | 0.044564188 |

| blue | GK | ACSL1 | 0.028065845 |
| --- | --- | --- | --- |
| blue | GCA | GAB2 | 0.038784083 |
| blue | GCA | FRAT2 | 0.034817135 |
| blue | GCA | FPR1 | 0.035886748 |
| blue | GCA | FOS | 0.02938399 |
| blue | GCA | FNDC3B | 0.026293381 |
| blue | GCA | FLOT2 | 0.042043737 |
| blue | GCA | FCGR2A | 0.03022788 |
| blue | GCA | FBXL5 | 0.033513835 |
| blue | GCA | FAM8A1 | 0.021482845 |
| blue | GCA | FAM49B | 0.02159574 |
| blue | GCA | EXOC6 | 0.03736209 |
| blue | GCA | EVI2B | 0.027653629 |
| blue | GCA | ETS2 | 0.032363222 |
| blue | GCA | EGLN1 | 0.033857518 |
| blue | GCA | DYSF | 0.023187356 |
| blue | GCA | CYSTM1 | 0.022221918 |
| blue | GCA | CYB5R4 | 0.0408069 |
| blue | GCA | CXCR2 | 0.023238253 |
| blue | GCA | CXCR1 | 0.020511657 |
| blue | GCA | CSF3R | 0.033900078 |
| blue | GCA | CPD | 0.021233722 |
| blue | GCA | COP1 | 0.021593812 |
| blue | GCA | CMTM6 | 0.033386698 |
| blue | GCA | CMTM2 | 0.029525681 |
| blue | GCA | CHIC2 | 0.020572742 |
| blue | GCA | CELF2 | 0.024581528 |
| blue | GCA | CEBPD | 0.023031491 |
| blue | GCA | CEBPB | 0.040457193 |
| blue | GCA | CD58 | 0.02754762 |
| blue | GCA | BEST1 | 0.027314668 |
| blue | GCA | BCL6 | 0.039930493 |
| blue | GCA | BCL3 | 0.042648791 |
| blue | GCA | BCL2A1 | 0.025411707 |
| blue | GCA | BASP1 | 0.041484788 |
| blue | GCA | B4GALT5 | 0.024580716 |
| blue | GCA | ATP6V1B2 | 0.030642564 |
| blue | GCA | ATP6V0B | 0.025438512 |
| blue | GCA | ARHGAP9 | 0.023281666 |
| blue | GCA | AQP9 | 0.054699891 |
| blue | GCA | ANXA3 | 0.022954666 |
| blue | GCA | ALOX5AP | 0.024224691 |
| blue | GCA | AKIRIN2 | 0.023437081 |
| blue | GCA | ADGRE5 | 0.025137478 |
| blue | GCA | ACSL4 | 0.027490239 |
| blue | GCA | ACSL1 | 0.041905011 |
| blue | GCA | ACOX1 | 0.039106575 |
| blue | GCA | ABHD5 | 0.022520699 |
| blue | GAB2 | FRAT2 | 0.023705827 |
| blue | GAB2 | FRAT1 | 0.027725439 |
| blue | GAB2 | FPR1 | 0.041744776 |
| blue | GAB2 | FOS | 0.021968208 |

| blue | GAB2 | FNIP1 | 0.043950167 |
| --- | --- | --- | --- |
| blue | GAB2 | FNDC3B | 0.037341251 |
| blue | GAB2 | FLOT2 | 0.023260237 |
| blue | GAB2 | FCHO2 | 0.023452976 |
| blue | GAB2 | FCGRT | 0.023934964 |
| blue | GAB2 | FCGR2A | 0.035577822 |
| blue | GAB2 | FBXL5 | 0.022380334 |
| blue | GAB2 | FAM8A1 | 0.028874446 |
| blue | GAB2 | FAM49B | 0.021793926 |
| blue | GAB2 | FAM160B1 | 0.024430112 |
| blue | GAB2 | EXTL3 | 0.020993897 |
| blue | GAB2 | EXOC6 | 0.040076311 |
| blue | GAB2 | EVI2B | 0.026258781 |
| blue | GAB2 | ETS2 | 0.037610917 |
| blue | GAB2 | ELL | 0.020672227 |
| blue | GAB2 | EGLN1 | 0.021556355 |
| blue | GAB2 | DYSF | 0.035047347 |
| blue | GAB2 | DNTTIP1 | 0.027437414 |
| blue | GAB2 | DIP2B | 0.021363014 |
| blue | GAB2 | DENND5A | 0.031564402 |
| blue | GAB2 | CYB5R4 | 0.033029273 |
| blue | GAB2 | CXCR2 | 0.028224191 |
| blue | GAB2 | CXCR1 | 0.033619631 |
| blue | GAB2 | CTBS | 0.038827125 |
| blue | GAB2 | CSF3R | 0.037914695 |
| blue | GAB2 | CPSF4 | 0.023174392 |
| blue | GAB2 | CPD | 0.046940034 |
| blue | GAB2 | COP1 | 0.03677437 |
| blue | GAB2 | CMTM6 | 0.027637907 |
| blue | GAB2 | CMTM2 | 0.03572784 |
| blue | GAB2 | CHST15 | 0.042634381 |
| blue | GAB2 | CFLAR | 0.024220256 |
| blue | GAB2 | CEBPD | 0.02636483 |
| blue | GAB2 | CEBPB | 0.037847695 |
| blue | GAB2 | CDA | 0.02127608 |
| blue | GAB2 | CCNJL | 0.029772344 |
| blue | GAB2 | C5AR1 | 0.02232691 |
| blue | GAB2 | C3orf62 | 0.030672051 |
| blue | GAB2 | C1RL | 0.023745112 |
| blue | GAB2 | C15orf39 | 0.032918174 |
| blue | GAB2 | BEST1 | 0.03552483 |
| blue | GAB2 | BCL6 | 0.04068031 |
| blue | GAB2 | BCL3 | 0.030863332 |
| blue | GAB2 | BCL2A1 | 0.029908291 |
| blue | GAB2 | BAZ2B | 0.020077512 |
| blue | GAB2 | BASP1 | 0.034645286 |
| blue | GAB2 | B4GALT5 | 0.030214013 |
| blue | GAB2 | ATP6V1B2 | 0.043993514 |
| blue | GAB2 | ATP6V0B | 0.03426054 |
| blue | GAB2 | ASPRV1 | 0.022311882 |
| blue | GAB2 | ARID3A | 0.032888 |
| blue | GAB2 | ARHGAP25 | 0.02016164 |

| blue | GAB2 | ARAP1 | 0.029328491 |
| --- | --- | --- | --- |
| blue | GAB2 | AQP9 | 0.046036086 |
| blue | GAB2 | ANKRD13A | 0.02064564 |
| blue | GAB2 | ALPK1 | 0.025419314 |
| blue | GAB2 | ALOX5AP | 0.023808936 |
| blue | GAB2 | AKR1B1 | 0.025978388 |
| blue | GAB2 | AGTPBP1 | 0.029351809 |
| blue | GAB2 | AGO4 | 0.027271324 |
| blue | GAB2 | ADGRE5 | 0.031146962 |
| blue | GAB2 | ADGRE3 | 0.028900732 |
| blue | GAB2 | ACSL4 | 0.028410035 |
| blue | GAB2 | ACSL1 | 0.034646193 |
| blue | GAB2 | ACOX1 | 0.03214253 |
| blue | GAB2 | ABHD5 | 0.02536359 |
| blue | FRAT2 | FBXL5 | 0.026410343 |
| blue | FRAT2 | CEBPB | 0.026377106 |
| blue | FRAT2 | BCL6 | 0.037538412 |
| blue | FRAT2 | BASP1 | 0.025507863 |
| blue | FRAT2 | AQP9 | 0.042514881 |
| blue | FRAT2 | ACSL1 | 0.041900915 |
| blue | FRAT1 | BCL6 | 0.02148895 |
| blue | FRAT1 | ARHGAP19 | 0.026797047 |
| blue | FRAT1 | AQP9 | 0.021313896 |
| blue | FRAT1 | ACSL1 | 0.021981975 |
| blue | FPR1 | FCGR2A | 0.02192445 |
| blue | FPR1 | CXCR1 | 0.033205601 |
| blue | FPR1 | CSF3R | 0.041999688 |
| blue | FPR1 | CEBPB | 0.039185656 |
| blue | FPR1 | BCL6 | 0.035311701 |
| blue | FPR1 | BASP1 | 0.021962281 |
| blue | FPR1 | ATP6V1B2 | 0.027314734 |
| blue | FPR1 | ATP6V0B | 0.020024552 |
| blue | FPR1 | AQP9 | 0.0487124 |
| blue | FPR1 | ACSL1 | 0.028456122 |
| blue | FOS | CYB5R4 | 0.029686084 |
| blue | FOS | CD58 | 0.024812061 |
| blue | FOS | AQP9 | 0.023158141 |
| blue | FOS | ACSL1 | 0.020212006 |
| blue | FNIP1 | CXCR1 | 0.022923422 |
| blue | FNIP1 | CSF3R | 0.032865034 |
| blue | FNIP1 | CHST15 | 0.021011013 |
| blue | FNIP1 | AQP9 | 0.021962766 |
| blue | FNDC3B | DYSF | 0.023341049 |
| blue | FNDC3B | CPSF4 | 0.023846624 |
| blue | FLOT2 | DYSF | 0.022164744 |
| blue | FLOT2 | CYSTM1 | 0.035167474 |
| blue | FLOT2 | CSF3R | 0.024668102 |
| blue | FLOT2 | CEBPB | 0.032438792 |
| blue | FLOT2 | BCL6 | 0.058573961 |
| blue | FLOT2 | BCL3 | 0.020328126 |
| blue | FLOT2 | BASP1 | 0.029287918 |
| blue | FLOT2 | AQP9 | 0.06636712 |

| blue | FLOT2 | ANXA3 | 0.038362758 |
| --- | --- | --- | --- |
| blue | FLOT2 | ACSL1 | 0.074004528 |
| blue | FGR | CSF3R | 0.031497965 |
| blue | FGR | CEBPB | 0.045205996 |
| blue | FGR | AQP9 | 0.026750941 |
| blue | FGR | ADGRE5 | 0.020388762 |
| blue | FFAR2 | FCGR2A | 0.021951943 |
| blue | FCHO2 | CSF3R | 0.022645524 |
| blue | FCGRT | ATP6V1B2 | 0.023341793 |
| blue | FCGR2A | FBXL5 | 0.023702324 |
| blue | FCGR2A | ETS2 | 0.022715763 |
| blue | FCGR2A | CXCR2 | 0.026780379 |
| blue | FCGR2A | CXCR1 | 0.043482711 |
| blue | FCGR2A | CSF3R | 0.038026724 |
| blue | FCGR2A | CEBPB | 0.024776409 |
| blue | FCGR2A | BCL6 | 0.038912555 |
| blue | FCGR2A | BCL3 | 0.020412059 |
| blue | FCGR2A | BASP1 | 0.021859148 |
| blue | FCGR2A | AQP9 | 0.050857314 |
| blue | FCGR2A | ALPK1 | 0.021483303 |
| blue | FCGR2A | ACSL1 | 0.033373964 |
| blue | FBXL5 | DYSF | 0.020388745 |
| blue | FBXL5 | CYB5R4 | 0.022369216 |
| blue | FBXL5 | BCL6 | 0.044726773 |
| blue | FBXL5 | BASP1 | 0.029006974 |
| blue | FBXL5 | AQP9 | 0.043354427 |
| blue | FBXL5 | ACSL1 | 0.049511745 |
| blue | FAM8A1 | CSF3R | 0.021962322 |
| blue | FAM8A1 | BCL6 | 0.025449927 |
| blue | FAM8A1 | AQP9 | 0.026832926 |
| blue | FAM8A1 | ACSL1 | 0.02306049 |
| blue | FAM49A | CXCR1 | 0.020337664 |
| blue | FAM49A | CSF3R | 0.032214624 |
| blue | FAM49A | CEBPB | 0.023010986 |
| blue | FAM49A | AQP9 | 0.023886566 |
| blue | FAM160B1 | DYSF | 0.025095201 |
| blue | FAM160B1 | CHST15 | 0.022019821 |
| blue | FAM160B1 | BCL6 | 0.024660325 |
| blue | FAM160B1 | AQP9 | 0.020310479 |
| blue | F5 | DYSF | 0.028648777 |
| blue | F5 | BCL6 | 0.046348462 |
| blue | F5 | AQP9 | 0.030933205 |
| blue | F5 | ACSL1 | 0.046614087 |
| blue | F11R | CXCR1 | 0.027964349 |
| blue | F11R | CSF3R | 0.024490808 |
| blue | EXTL3 | CHST15 | 0.021718985 |
| blue | EXTL3 | CCNJL | 0.022339102 |
| blue | EXTL3 | ARID3A | 0.02101054 |
| blue | EXOC6 | CSF3R | 0.03446354 |
| blue | EXOC6 | CHST15 | 0.020859398 |
| blue | EXOC6 | CEBPB | 0.042896005 |
| blue | EXOC6 | BCL6 | 0.042530024 |

| blue | EXOC6 | AQP9 | 0.048935179 |
| --- | --- | --- | --- |
| blue | EXOC6 | ACSL1 | 0.031351694 |
| blue | EVI2B | CXCR2 | 0.04742529 |
| blue | EVI2B | CXCR1 | 0.028118872 |
| blue | EVI2B | CSF3R | 0.03386323 |
| blue | EVI2B | CMTM2 | 0.021340229 |
| blue | EVI2B | CEBPB | 0.026966975 |
| blue | EVI2B | ATP6V1B2 | 0.023796042 |
| blue | EVI2B | AQP9 | 0.038937066 |
| blue | ETS2 | DYSF | 0.020626572 |
| blue | ETS2 | CXCR1 | 0.027122347 |
| blue | ETS2 | CSF3R | 0.029413309 |
| blue | ETS2 | CEBPB | 0.029507755 |
| blue | ETS2 | BCL6 | 0.036328128 |
| blue | ETS2 | BASP1 | 0.021040449 |
| blue | ETS2 | AQP9 | 0.042511621 |
| blue | ETS2 | ACSL1 | 0.03199902 |
| blue | ELL | CHST15 | 0.021591789 |
| blue | ELL | BCL6 | 0.024008609 |
| blue | ELL | AQP9 | 0.022353415 |
| blue | EGLN1 | BCL6 | 0.033391275 |
| blue | EGLN1 | BASP1 | 0.027486161 |
| blue | EGLN1 | AQP9 | 0.036541298 |
| blue | EGLN1 | ACSL1 | 0.033813281 |
| blue | EGLN1 | ACOX1 | 0.021056244 |
| blue | DYSF | DENND5A | 0.045633595 |
| blue | DYSF | CYSTM1 | 0.021203036 |
| blue | DYSF | CREB5 | 0.025430743 |
| blue | DYSF | CHST15 | 0.023814416 |
| blue | DYSF | CDA | 0.024599945 |
| blue | DYSF | CCNJL | 0.026496666 |
| blue | DYSF | CANT1 | 0.021652502 |
| blue | DYSF | C5AR1 | 0.025549426 |
| blue | DYSF | C3orf62 | 0.021545283 |
| blue | DYSF | C15orf39 | 0.022061429 |
| blue | DYSF | BRI3 | 0.020666725 |
| blue | DYSF | BCL6 | 0.031360942 |
| blue | DYSF | BCL3 | 0.045272108 |
| blue | DYSF | BAZ2B | 0.021485309 |
| blue | DYSF | BASP1 | 0.049694359 |
| blue | DYSF | ARID3A | 0.030763579 |
| blue | DYSF | ARAP1 | 0.026175418 |
| blue | DYSF | AQP9 | 0.023136728 |
| blue | DYSF | APMAP | 0.027519356 |
| blue | DYSF | APBB1IP | 0.024247874 |
| blue | DYSF | ANPEP | 0.027857412 |
| blue | DYSF | ALPK1 | 0.02024843 |
| blue | DYSF | AKIRIN2 | 0.021569345 |
| blue | DYSF | AGTPBP1 | 0.028565973 |
| blue | DYSF | ADGRG3 | 0.021396259 |
| blue | DYSF | ADAM8 | 0.020836632 |
| blue | DYSF | ACSL4 | 0.023890268 |

| blue | DYSF | ACSL1 | 0.035060383 |
| --- | --- | --- | --- |
| blue | DPP7 | CUX1 | 0.021492495 |
| blue | DNTTIP1 | CSF3R | 0.023738499 |
| blue | DNTTIP1 | CEBPB | 0.023388832 |
| blue | DNTTIP1 | AQP9 | 0.031953411 |
| blue | DHX34 | CSF3R | 0.029932594 |
| blue | DHX34 | ABTB1 | 0.020672107 |
| blue | DENND5A | BCL6 | 0.031032914 |
| blue | DENND5A | BASP1 | 0.029133316 |
| blue | DENND5A | AQP9 | 0.023615105 |
| blue | DENND5A | ACSL1 | 0.026284989 |
| blue | DCP2 | ARID3A | 0.024152479 |
| blue | DAZAP2 | ATP6V1B2 | 0.026850954 |
| blue | CYSTM1 | BCL6 | 0.041961233 |
| blue | CYSTM1 | BASP1 | 0.021493694 |
| blue | CYSTM1 | AQP9 | 0.04155237 |
| blue | CYSTM1 | ANXA3 | 0.028683595 |
| blue | CYSTM1 | ACSL1 | 0.050202429 |
| blue | CYB5R4 | CSF3R | 0.022442804 |
| blue | CYB5R4 | CMTM6 | 0.028983368 |
| blue | CYB5R4 | CMTM2 | 0.020471573 |
| blue | CYB5R4 | CEBPB | 0.022554089 |
| blue | CYB5R4 | CD58 | 0.024922819 |
| blue | CYB5R4 | CD46 | 0.021208052 |
| blue | CYB5R4 | BCL6 | 0.029699777 |
| blue | CYB5R4 | BASP1 | 0.025714127 |
| blue | CYB5R4 | AQP9 | 0.036915124 |
| blue | CYB5R4 | ACSL1 | 0.026294576 |
| blue | CYB5R4 | ACOX1 | 0.02628859 |
| blue | CXCR2 | CXCR1 | 0.051264125 |
| blue | CXCR2 | CSF3R | 0.038433367 |
| blue | CXCR2 | AQP9 | 0.029378072 |
| blue | CXCR1 | CTBS | 0.020111998 |
| blue | CXCR1 | CSF3R | 0.043667821 |
| blue | CXCR1 | CPD | 0.02201976 |
| blue | CXCR1 | CFLAR | 0.021567714 |
| blue | CXCR1 | CEBPB | 0.020517406 |
| blue | CXCR1 | BEST1 | 0.02390497 |
| blue | CXCR1 | BCL6 | 0.020256236 |
| blue | CXCR1 | AQP9 | 0.026094688 |
| blue | CXCR1 | ALPK1 | 0.031670627 |
| blue | CXCR1 | AKR1B1 | 0.023285465 |
| blue | CUX1 | CBL | 0.049831583 |
| blue | CUX1 | CAPZA2 | 0.033967309 |
| blue | CUX1 | BRI3 | 0.020057994 |
| blue | CUX1 | APBB1IP | 0.021285767 |
| blue | CTBS | CSF3R | 0.027740292 |
| blue | CTBS | CHST15 | 0.025054739 |
| blue | CTBS | BCL6 | 0.020897663 |
| blue | CTBS | AQP9 | 0.024706751 |
| blue | CSF3R | CPD | 0.032205175 |
| blue | CSF3R | COP1 | 0.034903874 |

| blue | CSF3R | CMTM2 | 0.033145126 |
| --- | --- | --- | --- |
| blue | CSF3R | CHST15 | 0.033625539 |
| blue | CSF3R | CFLAR | 0.023910839 |
| blue | CSF3R | CELF2 | 0.027691865 |
| blue | CSF3R | CEBPB | 0.047280261 |
| blue | CSF3R | CEACAM3 | 0.024840946 |
| blue | CSF3R | CA4 | 0.025268986 |
| blue | CSF3R | C1RL | 0.02405356 |
| blue | CSF3R | BEST1 | 0.035866574 |
| blue | CSF3R | BCL6 | 0.034856749 |
| blue | CSF3R | BCL3 | 0.020088788 |
| blue | CSF3R | BCL2A1 | 0.022004899 |
| blue | CSF3R | B4GALT5 | 0.029939446 |
| blue | CSF3R | ATP6V1B2 | 0.029226116 |
| blue | CSF3R | ATP6V0B | 0.032041038 |
| blue | CSF3R | ASPRV1 | 0.023250342 |
| blue | CSF3R | AQP9 | 0.048925456 |
| blue | CSF3R | ALPK1 | 0.022108308 |
| blue | CSF3R | ALOX5 | 0.030037469 |
| blue | CSF3R | AKR1B1 | 0.021974444 |
| blue | CSF3R | AGO4 | 0.022631114 |
| blue | CSF3R | ADGRE5 | 0.044492351 |
| blue | CSF3R | ACSL1 | 0.026582102 |
| blue | CSF3R | ACOX1 | 0.025854341 |
| blue | CSF3R | ABTB1 | 0.04445461 |
| blue | CREB5 | BASP1 | 0.023831624 |
| blue | CREB5 | ACSL1 | 0.024953725 |
| blue | CPSF4 | CEBPD | 0.023431075 |
| blue | CPSF4 | C16orf58 | 0.022076301 |
| blue | CPSF4 | BMS1 | 0.024992525 |
| blue | CPSF4 | ACSL4 | 0.021392661 |
| blue | CPD | COP1 | 0.021187276 |
| blue | CPD | CMTM2 | 0.020464115 |
| blue | CPD | CHST15 | 0.029185926 |
| blue | CPD | CEBPB | 0.020520888 |
| blue | CPD | BCL6 | 0.021465422 |
| blue | CPD | ATP6V1B2 | 0.024195289 |
| blue | CPD | ARID3A | 0.021398371 |
| blue | CPD | AQP9 | 0.03249681 |
| blue | COP1 | CMTM2 | 0.024933995 |
| blue | COP1 | CHST15 | 0.036741683 |
| blue | COP1 | CEBPB | 0.026283601 |
| blue | COP1 | BCL6 | 0.033075166 |
| blue | COP1 | ATP6V1B2 | 0.024134388 |
| blue | COP1 | AQP9 | 0.04207664 |
| blue | COP1 | AGO4 | 0.02113472 |
| blue | COP1 | ADGRE3 | 0.020971632 |
| blue | CMTM6 | ATP6V1B2 | 0.024983328 |
| blue | CMTM6 | AQP9 | 0.025641769 |
| blue | CMTM2 | CHST15 | 0.026563099 |
| blue | CMTM2 | CEBPB | 0.025997876 |
| blue | CMTM2 | BCL6 | 0.02623244 |

| blue | CMTM2 | ATP6V1B2 | 0.033063931 |
| --- | --- | --- | --- |
| blue | CMTM2 | ATP6V0B | 0.033713801 |
| blue | CMTM2 | AQP9 | 0.04202339 |
| blue | CMTM2 | ADGRE3 | 0.026743695 |
| blue | CHST15 | CEBPB | 0.021012978 |
| blue | CHST15 | CCNJL | 0.021470946 |
| blue | CHST15 | BCL6 | 0.026288827 |
| blue | CHST15 | ARID3A | 0.021749762 |
| blue | CHST15 | AQP9 | 0.033258041 |
| blue | CHST15 | ADGRE3 | 0.029958478 |
| blue | CHMP2A | CEBPB | 0.027863059 |
| blue | CHMP2A | AQP9 | 0.031644986 |
| blue | CFLAR | BCL6 | 0.025582671 |
| blue | CFLAR | ATG16L2 | 0.021931227 |
| blue | CELF2 | CEBPB | 0.045479875 |
| blue | CELF2 | BCL6 | 0.029210091 |
| blue | CELF2 | AQP9 | 0.038230847 |
| blue | CELF2 | ACSL1 | 0.021516181 |
| blue | CEBPB | CEACAM3 | 0.028241818 |
| blue | CEBPB | CA4 | 0.022110201 |
| blue | CEBPB | C1RL | 0.0247203 |
| blue | CEBPB | BTBD10 | 0.020581845 |
| blue | CEBPB | BEST1 | 0.023586499 |
| blue | CEBPB | BCL6 | 0.042182054 |
| blue | CEBPB | BCL2A1 | 0.025937846 |
| blue | CEBPB | B4GALT5 | 0.031322621 |
| blue | CEBPB | ATP6V1B2 | 0.024212845 |
| blue | CEBPB | ATP6V0B | 0.025070193 |
| blue | CEBPB | ASPRV1 | 0.020958689 |
| blue | CEBPB | AQP9 | 0.066276015 |
| blue | CEBPB | ANKS1A | 0.023874462 |
| blue | CEBPB | ALOX5AP | 0.025380171 |
| blue | CEBPB | ALOX5 | 0.034728898 |
| blue | CEBPB | AGO4 | 0.020667549 |
| blue | CEBPB | ADGRE5 | 0.041334749 |
| blue | CEBPB | ACSL1 | 0.033829041 |
| blue | CEBPB | ACOX1 | 0.022140797 |
| blue | CEBPB | ABTB1 | 0.025183273 |
| blue | CEACAM3 | BCL6 | 0.046990814 |
| blue | CEACAM3 | AQP9 | 0.052770473 |
| blue | CEACAM3 | ALOX5 | 0.030788898 |
| blue | CEACAM3 | ACSL1 | 0.030102844 |
| blue | CD93 | ARID3A | 0.020242372 |
| blue | CD46 | AQP9 | 0.022519185 |
| blue | CCNJL | ARID3A | 0.021546283 |
| blue | CBL | CAPZA2 | 0.023554516 |
| blue | CAPZA2 | C5AR1 | 0.023565408 |
| blue | CAPZA2 | C16orf58 | 0.022614154 |
| blue | CAP1 | ATP6V1B2 | 0.024305168 |
| blue | CANT1 | C5AR1 | 0.026867077 |
| blue | CANT1 | C16orf58 | 0.0231635 |
| blue | CANT1 | ARAP1 | 0.020052993 |

| blue | CANT1 | APBB1IP | 0.021816147 |
| --- | --- | --- | --- |
| blue | CANT1 | ADAM8 | 0.024359676 |
| blue | CA4 | BCL6 | 0.024894333 |
| blue | CA4 | AQP9 | 0.042249106 |
| blue | CA4 | ACSL1 | 0.02245658 |
| blue | C5AR1 | C16orf58 | 0.027278623 |
| blue | C5AR1 | BRI3 | 0.023467643 |
| blue | C5AR1 | BASP1 | 0.03141746 |
| blue | C5AR1 | APBB1IP | 0.03035474 |
| blue | C1RL | BCL6 | 0.030737744 |
| blue | C1RL | AQP9 | 0.028448418 |
| blue | C1RL | ACSL1 | 0.028351209 |
| blue | C15orf39 | ATP6V1B2 | 0.024457808 |
| blue | BTBD10 | AQP9 | 0.022810591 |
| blue | BST1 | AQP9 | 0.025002963 |
| blue | BRI3 | BASP1 | 0.025366525 |
| blue | BEST1 | BCL6 | 0.025548213 |
| blue | BEST1 | AQP9 | 0.031741002 |
| blue | BCL6 | BCL3 | 0.033541076 |
| blue | BCL6 | BCL2A1 | 0.031719274 |
| blue | BCL6 | BASP1 | 0.037058299 |
| blue | BCL6 | B4GALT5 | 0.047595627 |
| blue | BCL6 | ASPRV1 | 0.021094799 |
| blue | BCL6 | ARHGAP9 | 0.023877734 |
| blue | BCL6 | AQP9 | 0.080968144 |
| blue | BCL6 | ANXA3 | 0.052135776 |
| blue | BCL6 | ALOX5 | 0.03144252 |
| blue | BCL6 | AGO4 | 0.023062104 |
| blue | BCL6 | ADM | 0.026163631 |
| blue | BCL6 | ADGRE5 | 0.021974581 |
| blue | BCL6 | ACSL4 | 0.020256527 |
| blue | BCL6 | ACSL1 | 0.076815611 |
| blue | BCL6 | ACOX1 | 0.026106116 |
| blue | BCL3 | BASP1 | 0.070929672 |
| blue | BCL3 | AQP9 | 0.037047877 |
| blue | BCL3 | ACSL1 | 0.039047465 |
| blue | BCL2A1 | AQP9 | 0.04075772 |
| blue | BCL2A1 | ACSL1 | 0.03679424 |
| blue | BAZ2B | ARAP1 | 0.022483815 |
| blue | BAZ2B | ANKRD13A | 0.02464543 |
| blue | BAZ2B | ADAM8 | 0.027876611 |
| blue | BASP1 | ARID3A | 0.021296085 |
| blue | BASP1 | AQP9 | 0.037565605 |
| blue | BASP1 | APMAP | 0.03197199 |
| blue | BASP1 | APBB1IP | 0.038382382 |
| blue | BASP1 | ANXA3 | 0.022923167 |
| blue | BASP1 | AKIRIN2 | 0.030093918 |
| blue | BASP1 | ACSL4 | 0.022461028 |
| blue | BASP1 | ACSL1 | 0.039660198 |
| blue | BASP1 | ACOX1 | 0.022740072 |
| blue | B4GALT5 | AQP9 | 0.049250058 |
| blue | B4GALT5 | ACSL1 | 0.03673894 |

| blue | ATP6V1B2 | ATP6V1A | 0.033683224 |
| --- | --- | --- | --- |
| blue | ATP6V1B2 | ATP6V0B | 0.043067975 |
| blue | ATP6V1B2 | AQP9 | 0.030303495 |
| blue | ATP6V1B2 | ALOX5AP | 0.023346961 |
| blue | ATP6V0B | AQP9 | 0.03314807 |
| blue | ASPRV1 | AQP9 | 0.030091836 |
| blue | ARID3A | ARAP1 | 0.021470482 |
| blue | ARID3A | ANPEP | 0.02601507 |
| blue | ARHGAP9 | AQP9 | 0.020764155 |
| blue | ARHGAP9 | ACSL1 | 0.021374569 |
| blue | ARAP1 | ANPEP | 0.028617887 |
| blue | ARAP1 | ADAM8 | 0.026739705 |
| blue | AQP9 | ANXA3 | 0.040177487 |
| blue | AQP9 | ANP32A | 0.025229572 |
| blue | AQP9 | ALOX5AP | 0.025937084 |
| blue | AQP9 | ALOX5 | 0.040007937 |
| blue | AQP9 | AGO4 | 0.026310124 |
| blue | AQP9 | ADGRE5 | 0.042671094 |
| blue | AQP9 | ACSL4 | 0.020804156 |
| blue | AQP9 | ACSL1 | 0.069884807 |
| blue | AQP9 | ACOX1 | 0.037082991 |
| blue | AQP9 | ABTB1 | 0.030284519 |
| blue | ANXA3 | ACSL1 | 0.074965982 |
| blue | ANPEP | ADAM8 | 0.020684216 |
| blue | ALPK1 | AKR1B1 | 0.022448165 |
| blue | ALOX5 | ACSL1 | 0.029118836 |
| blue | ADM | ACSL1 | 0.026112468 |
| blue | ACSL4 | ACSL1 | 0.021332907 |
| blue | ACSL1 | ACOX1 | 0.023920469 |
| yellow | ZRANB2 | LUC7L3 | 0.067096805 |
| yellow | ZNF876P | ZNF860 | 0.105314046 |
| yellow | ZNF876P | ZNF786 | 0.081884258 |
| yellow | ZNF876P | ZNF738 | 0.10324801 |
| yellow | ZNF876P | ZNF69 | 0.162759879 |
| yellow | ZNF876P | ZNF682 | 0.164101283 |
| yellow | ZNF876P | ZNF674 | 0.088338644 |
| yellow | ZNF876P | ZNF669 | 0.130492202 |
| yellow | ZNF876P | ZNF652 | 0.064248512 |
| yellow | ZNF876P | ZNF577 | 0.140626942 |
| yellow | ZNF876P | ZNF557 | 0.11235246 |
| yellow | ZNF876P | ZNF549 | 0.162046798 |
| yellow | ZNF876P | ZNF493 | 0.101855644 |
| yellow | ZNF876P | ZNF483 | 0.160879814 |
| yellow | ZNF876P | ZNF430 | 0.111810103 |
| yellow | ZNF876P | ZNF394 | 0.148780876 |
| yellow | ZNF876P | ZNF223 | 0.073003307 |
| yellow | ZNF876P | ZNF14 | 0.140842551 |
| yellow | ZNF876P | ZMAT3 | 0.124148284 |
| yellow | ZNF876P | ZFC3H1 | 0.043682049 |
| yellow | ZNF876P | YRDC | 0.183322812 |
| yellow | ZNF876P | XRCC2 | 0.19440684 |
| yellow | ZNF876P | XPNPEP3 | 0.129810722 |
