# Supplementary material for: Identification of diagnostic mRNA biomarkers in whole blood for ankylosing spondylitis using WGCNA and machine learning feature selection
Source: Front Immunol. 2022 Sep 12;13:956027. doi: 10.3389/fimmu.2022.956027 (PMC9510835; doi:10.3389/fimmu.2022.956027)
Supplement: Supplementary file 4 [file Table_3.docx]

Supplementary Table 3. Differentail expressed mRNAs

| mRNA | logFC | AveExpr | P.Value | adj.P.Val |
| --- | --- | --- | --- | --- |
| SMG7 | 0.330499 | 7.9929491 | 1.10E-09 | 1.29E-05 |
| SORL1 | 0.604582 | 11.206737 | 1.79E-09 | 1.29E-05 |
| TINF2 | -0.32563 | 9.3175894 | 2.42E-09 | 1.29E-05 |
| UQCRBP1 | -0.27505 | 11.732818 | 2.47E-09 | 1.29E-05 |
| PSMC4 | -0.24685 | 7.3901318 | 3.18E-09 | 1.33E-05 |
| MTMR4 | 0.239517 | 8.4028192 | 4.35E-09 | 1.52E-05 |
| STAT5B | 0.363932 | 8.5394859 | 7.80E-09 | 2.16E-05 |
| NDUFA1 | -0.27273 | 11.012421 | 8.27E-09 | 2.16E-05 |
| PAN3 | 0.275047 | 9.2896014 | 9.62E-09 | 2.20E-05 |
| BAZ1A | 0.381628 | 9.4803588 | 1.07E-08 | 2.20E-05 |
| PDIA3P1 | -0.36681 | 8.3288672 | 1.17E-08 | 2.20E-05 |
| TAB2 | 0.260212 | 7.8654931 | 1.26E-08 | 2.20E-05 |
| PUF60 | -0.316 | 9.4642798 | 1.45E-08 | 2.33E-05 |
| NRDC | 0.320641 | 9.8191803 | 1.90E-08 | 2.72E-05 |
| LARP4B | 0.203783 | 8.6429796 | 1.95E-08 | 2.72E-05 |
| GPN2 | -0.16852 | 7.6041816 | 2.17E-08 | 2.84E-05 |
| LRRC41 | -0.23992 | 7.6712201 | 2.41E-08 | 2.94E-05 |
| GGA1 | 0.188929 | 14.605382 | 2.61E-08 | 2.94E-05 |
| PAPOLA | 0.203915 | 9.8549887 | 2.66E-08 | 2.94E-05 |
| FAM50A | -0.19275 | 9.3798233 | 3.36E-08 | 3.52E-05 |
| CEP350 | 0.312323 | 8.8228964 | 3.61E-08 | 3.60E-05 |
| HPS6 | -0.33361 | 8.7494068 | 3.81E-08 | 3.62E-05 |
| JMJD8 | -0.30442 | 9.138643 | 4.25E-08 | 3.87E-05 |
| PDXP | -0.26373 | 7.6324682 | 5.32E-08 | 3.97E-05 |
| C8orf76 | -0.1236 | 8.3919258 | 5.34E-08 | 3.97E-05 |
| C1001909 | 0.45464 | 8.2905899 | 5.35E-08 | 3.97E-05 |
| ZFC3H1 | 0.227336 | 7.9237441 | 5.37E-08 | 3.97E-05 |
| DIMT1 | -0.28183 | 8.3917061 | 5.38E-08 | 3.97E-05 |
| NFAT5 | 0.143513 | 6.9142144 | 5.50E-08 | 3.97E-05 |
| CD81 | -0.43697 | 11.308614 | 5.87E-08 | 4.09E-05 |
| CREBRF | 0.347376 | 9.6527548 | 6.79E-08 | 4.59E-05 |
| SETD3 | -0.18055 | 9.0830376 | 7.61E-08 | 4.89E-05 |
| KAT6A | 0.360718 | 9.7638791 | 7.71E-08 | 4.89E-05 |
| FKBP1A | 0.416862 | 9.456871 | 8.81E-08 | 5.20E-05 |
| INTS3 | 0.247729 | 7.9754301 | 8.92E-08 | 5.20E-05 |
| RERE | 0.333967 | 8.5240519 | 8.94E-08 | 5.20E-05 |
| NCKAP1L | -0.25638 | 9.2973663 | 9.23E-08 | 5.22E-05 |
| ASTE1 | -0.17109 | 7.6813702 | 9.87E-08 | 5.36E-05 |
| BCL7B | -0.1717 | 7.359428 | 9.98E-08 | 5.36E-05 |
| PLPP5 | 0.459048 | 13.222841 | 1.03E-07 | 5.38E-05 |
| MRPL36 | -0.26319 | 8.0692143 | 1.08E-07 | 5.44E-05 |
| CDC37 | -0.26364 | 11.041396 | 1.10E-07 | 5.44E-05 |
| DOCK8 | 0.315311 | 10.199894 | 1.12E-07 | 5.44E-05 |
| MATK | -0.59604 | 8.8084052 | 1.17E-07 | 5.55E-05 |
| TMEM35B | -0.29561 | 8.7207761 | 1.19E-07 | 5.55E-05 |
| CDC42SE | 0.403972 | 9.6270349 | 1.25E-07 | 5.70E-05 |
| CUX1 | 0.308644 | 7.9837243 | 1.33E-07 | 5.92E-05 |
| RPL20-A | -0.22752 | 8.0330323 | 1.37E-07 | 5.96E-05 |
| SBNO2 | 0.32769 | 7.70938 | 1.45E-07 | 6.15E-05 |
| PICALM | 0.380154 | 10.735255 | 1.47E-07 | 6.15E-05 |

| DENND4B | 0.315358 | 8.9106013 | 1.50E-07 | 6.17E-05 |
| --- | --- | --- | --- | --- |
| HIRIP3 | -0.22012 | 7.4978946 | 1.63E-07 | 6.57E-05 |
| LCMT1 | -0.25675 | 8.2531674 | 1.71E-07 | 6.74E-05 |
| COX17 | -0.27137 | 9.3719557 | 1.81E-07 | 7.02E-05 |
| ZNF549 | 0.478282 | 12.920973 | 2.02E-07 | 7.50E-05 |
| PPP1R10 | 0.182959 | 7.5432952 | 2.04E-07 | 7.50E-05 |
| APBB1IP | 0.35307 | 10.191328 | 2.04E-07 | 7.50E-05 |
| GLRX3 | -0.26005 | 8.0316912 | 2.08E-07 | 7.52E-05 |
| IRAK2 | -0.18866 | 7.3500209 | 2.44E-07 | 8.51E-05 |
| NIN | 0.267081 | 9.558111 | 2.44E-07 | 8.51E-05 |
| CBL | 0.344939 | 9.0396927 | 2.49E-07 | 8.53E-05 |
| THEM6 | -0.24257 | 7.4986879 | 2.59E-07 | 8.68E-05 |
| CLIP1 | 0.244108 | 9.137064 | 2.61E-07 | 8.68E-05 |
| MAN2A1 | 0.24809 | 8.2120821 | 2.69E-07 | 8.79E-05 |
| VPS13B | 0.253024 | 7.2581366 | 2.73E-07 | 8.79E-05 |
| C1orf174 | -0.20547 | 8.1673059 | 2.83E-07 | 8.98E-05 |
| TMEM141 | -0.19086 | 7.4462314 | 2.90E-07 | 9.07E-05 |
| SLC41A3 | -0.24728 | 7.9003096 | 3.02E-07 | 9.31E-05 |
| XXYLT1 | -0.19419 | 7.3723536 | 3.12E-07 | 9.47E-05 |
| NDUFA12 | -0.38692 | 8.825007 | 3.26E-07 | 9.75E-05 |
| NDUFB4 | -0.28571 | 9.4780357 | 3.49E-07 | 0.00010217 |
| UBE4A | 0.194254 | 8.5826673 | 3.51E-07 | 0.00010217 |
| XPNPEP3 | 0.360157 | 14.225289 | 3.71E-07 | 0.000106238 |
| POLE3 | -0.32513 | 9.4169614 | 3.84E-07 | 0.000108742 |
| SDHAF2 | -0.33957 | 8.9029166 | 4.07E-07 | 0.000113658 |
| PKN2 | 0.337405 | 8.1510797 | 4.17E-07 | 0.000114976 |
| HARS2 | 0.144889 | 8.798931 | 4.27E-07 | 0.00011608 |
| CANT1 | 0.297158 | 8.4343037 | 4.40E-07 | 0.000118045 |
| EHBP1L1 | 0.356292 | 9.5775293 | 4.50E-07 | 0.000119158 |
| NUDC | -0.32036 | 9.0967747 | 4.68E-07 | 0.00012249 |
| UBAC2 | -0.25437 | 8.4584236 | 4.76E-07 | 0.000122975 |
| GPN3 | -0.19826 | 7.5219613 | 4.99E-07 | 0.00012681 |
| MAN1B1 | -0.1767 | 8.1979261 | 5.03E-07 | 0.00012681 |
| SYF2 | -0.23255 | 9.756177 | 5.34E-07 | 0.00013289 |
| RESF1 | 0.382545 | 11.330908 | 5.44E-07 | 0.00013289 |
| CENPB | -0.29561 | 9.0620678 | 5.46E-07 | 0.00013289 |
| NGRN | -0.25834 | 9.3729076 | 5.58E-07 | 0.000133001 |
| RHEB | -0.2198 | 8.8747222 | 5.59E-07 | 0.000133001 |
| KLRC1 | -0.28023 | 7.072911 | 5.87E-07 | 0.000136937 |
| SFR1 | -0.18374 | 7.234696 | 5.90E-07 | 0.000136937 |
| SLC9A3R | -0.31592 | 9.0516546 | 5.95E-07 | 0.000136937 |
| MED13L | 0.287715 | 7.7686135 | 6.03E-07 | 0.000137191 |
| ZNFX1 | 0.272624 | 8.8285794 | 6.41E-07 | 0.000144027 |
| UNC50 | -0.15832 | 9.0757619 | 6.48E-07 | 0.000144027 |
| MIF4GD | -0.23064 | 8.1479964 | 6.54E-07 | 0.000144027 |
| KMT2B | 0.231377 | 8.2115058 | 6.76E-07 | 0.000147347 |
| MRPL33 | -0.28447 | 9.7559365 | 7.20E-07 | 0.000154021 |
| NACA4P | -0.30615 | 8.4460712 | 7.21E-07 | 0.000154021 |
| STAT5A | 0.326006 | 9.1255173 | 7.49E-07 | 0.000157841 |
| RPL36AL | -0.28241 | 12.16831 | 7.55E-07 | 0.000157841 |
| RAPPC6 | -0.23464 | 7.8690492 | 7.62E-07 | 0.000157841 |
| ZNF483 | 0.611318 | 10.694246 | 7.74E-07 | 0.000158581 |

| HERC1 | 0.155201 | 8.3265865 | 7.80E-07 | 0.000158581 |
| --- | --- | --- | --- | --- |
| GDI1 | 0.256946 | 8.1113877 | 8.12E-07 | 0.000163359 |
| SF3B6 | -0.25564 | 9.5356802 | 8.58E-07 | 0.00017102 |
| DICER1 | 0.283611 | 8.4433647 | 8.73E-07 | 0.00017136 |
| TBC1D31 | -0.18465 | 7.4930095 | 8.76E-07 | 0.00017136 |
| C1orf162 | -0.24688 | 11.209387 | 8.87E-07 | 0.000171951 |
| TPRKB | -0.20993 | 7.6170235 | 9.15E-07 | 0.000175763 |
| SF3A3 | -0.38099 | 8.8762675 | 9.65E-07 | 0.000183587 |
| CIAO1 | -0.25841 | 9.2018663 | 9.91E-07 | 0.000185432 |
| PRDX3 | -0.29134 | 9.0802474 | 9.92E-07 | 0.000185432 |
| PSMB7 | -0.24777 | 8.2782547 | 1.01E-06 | 0.000187388 |
| FBRS | 0.219981 | 8.0906477 | 1.05E-06 | 0.000192248 |
| RAB5C | 0.339432 | 8.5738455 | 1.06E-06 | 0.000192248 |
| SEMA3E | 0.50237 | 10.321293 | 1.09E-06 | 0.000194467 |
| GIMAP6 | -0.29937 | 9.0950835 | 1.09E-06 | 0.000194467 |
| COMMD7 | -0.24997 | 9.3164975 | 1.11E-06 | 0.000195905 |
| TNFAIP8 | -0.3335 | 8.5539703 | 1.13E-06 | 0.000195905 |
| MTF1 | 0.304084 | 8.0455032 | 1.15E-06 | 0.000195905 |
| SPHK2 | -0.19572 | 8.0714738 | 1.16E-06 | 0.000195905 |
| PSMD10 | -0.20644 | 8.2618305 | 1.17E-06 | 0.000195905 |
| NRNPA1P | -0.42402 | 10.275634 | 1.17E-06 | 0.000195905 |
| LSMEM1 | 0.212163 | 7.1360251 | 1.18E-06 | 0.000195905 |
| PLD3 | -0.2273 | 8.4559813 | 1.19E-06 | 0.000195905 |
| COX5B | -0.22731 | 10.453706 | 1.20E-06 | 0.000195905 |
| RNF216 | -0.18715 | 8.3298623 | 1.20E-06 | 0.000195905 |
| GTF2E2 | -0.19224 | 9.0566812 | 1.21E-06 | 0.000195905 |
| C20orf27 | -0.2845 | 8.0077947 | 1.21E-06 | 0.000195905 |
| IL2RB | -0.55193 | 9.8116904 | 1.23E-06 | 0.000195905 |
| VNS1AB | 0.279093 | 8.8028152 | 1.24E-06 | 0.000195905 |
| RFX1 | 0.207327 | 8.1241792 | 1.24E-06 | 0.000195905 |
| KANSL1 | 0.187611 | 9.6303465 | 1.24E-06 | 0.000195905 |
| DDX19B | -0.22859 | 7.9143413 | 1.26E-06 | 0.000196851 |
| MAGT1 | 0.526643 | 11.599541 | 1.30E-06 | 0.000201015 |
| BAZ2B | 0.382682 | 9.0467503 | 1.32E-06 | 0.000203197 |
| MKLN1 | 0.179911 | 8.9926729 | 1.33E-06 | 0.000203888 |
| HELZ | 0.2085 | 7.8417754 | 1.38E-06 | 0.000208369 |
| EXOSC3 | -0.1926 | 8.604877 | 1.38E-06 | 0.000208369 |
| SCIMP | -0.18425 | 7.206764 | 1.43E-06 | 0.000214165 |
| PHF2 | 0.194775 | 7.8817378 | 1.48E-06 | 0.00021903 |
| RC3H2 | 0.17723 | 7.8826486 | 1.49E-06 | 0.00021903 |
| CHCHD4 | -0.17592 | 7.6405396 | 1.52E-06 | 0.000222227 |
| TMEM17 | 0.512435 | 12.415678 | 1.53E-06 | 0.000222227 |
| SQSTM1 | -0.277 | 11.135982 | 1.65E-06 | 0.000237644 |
| MEM126 | -0.26516 | 9.060251 | 1.66E-06 | 0.000237644 |
| XRCC2 | 0.552967 | 11.064795 | 1.67E-06 | 0.000237644 |
| MAP2K4 | 0.269962 | 8.2394616 | 1.69E-06 | 0.000238411 |
| MEFV | 0.347932 | 7.7062603 | 1.70E-06 | 0.000239093 |
| ALDOB | 0.109728 | 6.8362183 | 1.73E-06 | 0.000241024 |
| GATAD2B | 0.208822 | 7.6945734 | 1.75E-06 | 0.000243116 |
| RPL30 | -0.23144 | 13.273702 | 1.77E-06 | 0.000243116 |
| FEM1C | 0.226302 | 8.3391124 | 1.79E-06 | 0.000243116 |
| ERAP2 | 0.421359 | 12.568154 | 1.80E-06 | 0.000243116 |

| DGUOK | -0.19742 | 9.1676564 | 1.81E-06 | 0.000243116 |
| --- | --- | --- | --- | --- |
| IQSEC1 | 0.259434 | 8.3529074 | 1.81E-06 | 0.000243116 |
| NBEAL2 | 0.390067 | 9.1945237 | 1.84E-06 | 0.000245024 |
| NDUFS4 | -0.25841 | 8.7358309 | 1.88E-06 | 0.000249129 |
| LPP | 0.227163 | 9.5622262 | 1.90E-06 | 0.000249129 |
| CCNB1IP1 | -0.18118 | 7.791231 | 1.90E-06 | 0.000249129 |
| EXO5 | 0.477996 | 11.233416 | 1.94E-06 | 0.000251993 |
| CORO7 | 0.231117 | 8.2037157 | 1.97E-06 | 0.000253436 |
| M6PR | -0.25278 | 8.5826411 | 1.98E-06 | 0.000253436 |
| AP1G1 | 0.186865 | 7.4525839 | 1.99E-06 | 0.000253436 |
| ASCC1 | -0.1588 | 7.763572 | 2.01E-06 | 0.000255247 |
| LYRM2 | -0.20309 | 7.7857145 | 2.04E-06 | 0.000257178 |
| ASAP1-IT | 0.227627 | 7.1216929 | 2.08E-06 | 0.000260967 |
| DDX17 | 0.355459 | 10.819324 | 2.14E-06 | 0.000267017 |
| GPCPD1 | 0.262112 | 7.8655413 | 2.18E-06 | 0.000268803 |
| SEMA4D | 0.230551 | 10.59209 | 2.18E-06 | 0.000268803 |
| LCOR | 0.255712 | 8.9219953 | 2.26E-06 | 0.000276449 |
| HINT3 | -0.34831 | 7.5931367 | 2.34E-06 | 0.000285002 |
| NBPF10 | 0.425538 | 11.680579 | 2.41E-06 | 0.000288563 |
| ZNF69 | 0.485129 | 11.662531 | 2.42E-06 | 0.000288563 |
| TSC22D4 | 0.275363 | 7.726851 | 2.42E-06 | 0.000288563 |
| POLR2G | -0.22159 | 9.4166142 | 2.44E-06 | 0.000288563 |
| TSR2 | -0.19315 | 8.4929323 | 2.45E-06 | 0.000288563 |
| NGDN | -0.22063 | 8.429126 | 2.45E-06 | 0.000288563 |
| BIRC2 | 0.287986 | 9.3261193 | 2.48E-06 | 0.000289971 |
| PCMTD1 | 0.237834 | 9.5873865 | 2.49E-06 | 0.000289971 |
| EIF4E | -0.18949 | 7.8784255 | 2.57E-06 | 0.000297356 |
| FKBP14 | 0.576015 | 12.074675 | 2.60E-06 | 0.000297356 |
| DYSF | 0.638175 | 9.5552483 | 2.61E-06 | 0.000297356 |
| ALAD | -0.17094 | 7.582668 | 2.61E-06 | 0.000297356 |
| VPS51 | -0.32453 | 9.3739661 | 2.76E-06 | 0.000311861 |
| KLHL36 | -0.17638 | 7.5819135 | 2.78E-06 | 0.000311861 |
| NCK1 | -0.22584 | 8.6770764 | 2.80E-06 | 0.000311861 |
| MFSD11 | 0.363162 | 9.2884652 | 2.80E-06 | 0.000311861 |
| UBAP2L | 0.186653 | 7.8179393 | 2.86E-06 | 0.000314665 |
| TMEM160 | -0.21679 | 8.2028065 | 2.86E-06 | 0.000314665 |
| KPNA6 | 0.268849 | 8.2027253 | 2.87E-06 | 0.000314665 |
| RRP36 | -0.1887 | 8.3963141 | 2.92E-06 | 0.000317846 |
| CHCHD2 | -0.26926 | 11.583266 | 2.99E-06 | 0.000324218 |
| GSDMA | -0.24084 | 7.0336719 | 3.04E-06 | 0.000327506 |
| CIRBP | -0.25755 | 10.103684 | 3.06E-06 | 0.000328414 |
| CYBC1 | 0.238161 | 8.6707453 | 3.09E-06 | 0.000329525 |
| RPL13AP6 | -0.45454 | 10.634995 | 3.16E-06 | 0.000335662 |
| GTF3C6 | -0.2519 | 8.4424029 | 3.25E-06 | 0.00034361 |
| PUM2 | 0.189463 | 8.8817892 | 3.34E-06 | 0.000351762 |
| DIP2B | 0.267651 | 9.399089 | 3.38E-06 | 0.000353588 |
| STARD3 | 0.157673 | 7.5353532 | 3.40E-06 | 0.000353588 |
| MSL1 | 0.32279 | 7.7545649 | 3.44E-06 | 0.000356205 |
| MTMR3 | 0.40684 | 9.6760801 | 3.48E-06 | 0.000357703 |
| CD69 | -0.33784 | 8.3177231 | 3.49E-06 | 0.000357703 |
| PSMA1 | -0.21653 | 9.506999 | 3.55E-06 | 0.000362617 |
| TUBB | -0.3725 | 9.7370744 | 3.58E-06 | 0.000363383 |

| BLOC1S4 | -0.19497 | 7.9998571 | 3.64E-06 | 0.000368251 |
| --- | --- | --- | --- | --- |
| POLR2A | 0.214952 | 9.8264779 | 3.66E-06 | 0.000368603 |
| YRDC | 0.502118 | 11.846859 | 3.81E-06 | 0.000375735 |
| HSPA4 | -0.1848 | 8.5065926 | 3.83E-06 | 0.000375735 |
| PIM2 | 0.269889 | 9.0573627 | 3.85E-06 | 0.000375735 |
| NCALD | -0.33374 | 7.7577554 | 3.85E-06 | 0.000375735 |
| NDUFA11 | -0.21845 | 8.5419562 | 3.87E-06 | 0.000375735 |
| EIF3CL | -0.17967 | 7.1088609 | 3.88E-06 | 0.000375735 |
| SNHG6 | -0.34228 | 9.876673 | 3.88E-06 | 0.000375735 |
| NDUFB7 | -0.26195 | 8.0451019 | 3.88E-06 | 0.000375735 |
| ATXN7L3 | 0.167491 | 8.2646492 | 3.90E-06 | 0.000375735 |
| MCRS1 | -0.17447 | 8.5917042 | 3.96E-06 | 0.000380069 |
| ZNF672 | -0.18547 | 7.8293982 | 3.99E-06 | 0.000381443 |
| TMEM88 | 0.200095 | 7.0770802 | 4.04E-06 | 0.000384304 |
| ZMAT3 | 0.39314 | 12.219469 | 4.06E-06 | 0.000384413 |
| ZNF394 | 0.481966 | 11.688177 | 4.08E-06 | 0.000384413 |
| DLGAP4 | 0.138165 | 7.2880827 | 4.19E-06 | 0.000393476 |
| GZMK | -0.65383 | 9.4474333 | 4.27E-06 | 0.00039367 |
| POLR2F | -0.19416 | 8.4832736 | 4.28E-06 | 0.00039367 |
| VKORC1 | -0.19443 | 9.1725716 | 4.29E-06 | 0.00039367 |
| ATG4B | -0.17146 | 7.4840941 | 4.30E-06 | 0.00039367 |
| CNOT2 | -0.17329 | 8.5222758 | 4.32E-06 | 0.00039367 |
| CREB5 | 0.563164 | 9.8635332 | 4.34E-06 | 0.00039367 |
| C8orf33 | -0.15984 | 7.4205105 | 4.34E-06 | 0.00039367 |
| ALDH9A1 | -0.23412 | 10.763273 | 4.34E-06 | 0.00039367 |
| ZNF585A | -0.13906 | 7.2505129 | 4.41E-06 | 0.00039824 |
| ATP5MG | -0.26832 | 11.026415 | 4.45E-06 | 0.000399189 |
| AFG3L2 | -0.20789 | 8.1235972 | 4.46E-06 | 0.000399189 |
| NIP7 | -0.21113 | 8.0094227 | 4.54E-06 | 0.000404186 |
| TRIM38 | 0.247245 | 8.0542274 | 4.56E-06 | 0.000404445 |
| ITGAX | 0.383321 | 9.3315331 | 4.60E-06 | 0.000406367 |
| ZDHHC18 | 0.395576 | 9.1184403 | 4.66E-06 | 0.0004097 |
| GNPDA1 | -0.2249 | 8.3343591 | 4.68E-06 | 0.00041018 |
| MRPL17 | -0.18424 | 7.9833412 | 4.77E-06 | 0.000416202 |
| TRIM21 | 0.325371 | 8.2755485 | 4.81E-06 | 0.000417424 |
| TCIRG1 | 0.309912 | 9.1940617 | 4.86E-06 | 0.000420712 |
| KIDINS22 | 0.254637 | 8.7245778 | 4.90E-06 | 0.000421626 |
| RRP7BP | 0.148367 | 14.717606 | 4.92E-06 | 0.000421626 |
| GRIPAP1 | 0.462413 | 11.101816 | 5.03E-06 | 0.000429815 |
| PDPK1 | 0.227189 | 8.6278425 | 5.09E-06 | 0.000431689 |
| ZNHIT3 | -0.26012 | 9.1846306 | 5.09E-06 | 0.000431689 |
| VEGFB | -0.38172 | 9.1144469 | 5.17E-06 | 0.000433814 |
| RRP8 | -0.12246 | 7.7376708 | 5.18E-06 | 0.000433814 |
| C6orf62 | 0.278383 | 10.079562 | 5.19E-06 | 0.000433814 |
| PQBP1 | -0.15442 | 7.6587978 | 5.21E-06 | 0.000433814 |
| CRTAP | -0.25736 | 7.8163695 | 5.22E-06 | 0.000433814 |
| UQCRH | -0.29069 | 10.760349 | 5.25E-06 | 0.000433994 |
| RPL13AP5 | -0.26869 | 13.815275 | 5.29E-06 | 0.00043567 |
| YTHDC1 | 0.20742 | 10.078849 | 5.31E-06 | 0.000436013 |
| ATP5PF | -0.2635 | 9.6087245 | 5.34E-06 | 0.000436254 |
| MEM184 | 0.20663 | 7.4555479 | 5.37E-06 | 0.000436254 |
| ZNF493 | 0.251658 | 7.5947903 | 5.38E-06 | 0.000436254 |

| MRPS18C | -0.21293 | 8.9801904 | 5.47E-06 | 0.000442225 |
| --- | --- | --- | --- | --- |
| SLC25A22 | -0.16581 | 7.185214 | 5.53E-06 | 0.000442436 |
| ENSA | -0.20256 | 7.7796977 | 5.53E-06 | 0.000442436 |
| CYCS | -0.27703 | 7.7572694 | 5.54E-06 | 0.000442436 |
| SON | 0.176157 | 10.515612 | 5.56E-06 | 0.000442786 |
| HNRNPA0 | -0.22928 | 8.6687153 | 5.66E-06 | 0.000448357 |
| FYB1 | 0.201894 | 11.252109 | 5.71E-06 | 0.00045069 |
| COX8A | -0.19698 | 10.588462 | 5.78E-06 | 0.000454457 |
| PIK3CA | 0.159271 | 7.5653604 | 5.95E-06 | 0.000464902 |
| VCPIP1 | 0.173793 | 8.2451084 | 5.97E-06 | 0.000464902 |
| CEP295 | 0.165141 | 7.7977885 | 5.98E-06 | 0.000464902 |
| EDEM3 | 0.199908 | 8.4084762 | 6.01E-06 | 0.000464902 |
| MAPK8IP | 0.282523 | 8.4358986 | 6.02E-06 | 0.000464902 |
| TRANK1 | 0.306895 | 8.1807897 | 6.14E-06 | 0.000472132 |
| SP3 | 0.272065 | 9.1738866 | 6.18E-06 | 0.000472132 |
| NRDE2 | 0.157132 | 7.9475538 | 6.18E-06 | 0.000472132 |
| KLRB1 | -0.58151 | 10.816669 | 6.35E-06 | 0.000483653 |
| LMF2 | 0.196267 | 7.678937 | 6.52E-06 | 0.000494798 |
| CD160 | -0.54131 | 7.8449455 | 6.55E-06 | 0.000494974 |
| KLHL22 | -0.2564 | 8.5066747 | 6.59E-06 | 0.000496062 |
| C16orf58 | -0.2548 | 8.4615237 | 6.64E-06 | 0.000497772 |
| DENND5A | 0.322697 | 9.6258807 | 6.78E-06 | 0.00050682 |
| COMMD5 | -0.16233 | 7.9335942 | 6.86E-06 | 0.000509642 |
| CCDC28B | -0.14531 | 7.0021491 | 6.88E-06 | 0.000509642 |
| NFKBIE | -0.19831 | 7.353519 | 6.92E-06 | 0.000509642 |
| CEP19 | 0.419856 | 11.724778 | 6.93E-06 | 0.000509642 |
| DNAJC8 | -0.25198 | 9.8011566 | 6.94E-06 | 0.000509642 |
| SIK3 | 0.185462 | 9.1701275 | 7.04E-06 | 0.000514877 |
| GCLM | 0.303232 | 7.6477134 | 7.14E-06 | 0.000520343 |
| CSNK1E | -0.19016 | 7.8973208 | 7.16E-06 | 0.000520343 |
| SCO1 | -0.13267 | 7.9679914 | 7.25E-06 | 0.000522298 |
| COX6A1 | -0.15718 | 9.0773942 | 7.26E-06 | 0.000522298 |
| REXO4 | -0.25211 | 7.346672 | 7.27E-06 | 0.000522298 |
| ENOPH1 | -0.22914 | 7.9493906 | 7.29E-06 | 0.000522298 |
| RBM4 | -0.1858 | 8.2354336 | 7.41E-06 | 0.000529172 |
| ZSWIM8 | 0.225136 | 8.6172643 | 7.43E-06 | 0.000529172 |
| RPS25 | -0.20723 | 14.128119 | 7.55E-06 | 0.000534594 |
| RUNX3 | -0.42283 | 10.474204 | 7.59E-06 | 0.000534594 |
| CCNQ | -0.16469 | 7.58311 | 7.59E-06 | 0.000534594 |
| GSTP1 | -0.26413 | 10.361869 | 7.61E-06 | 0.000534594 |
| EMC6 | -0.19355 | 8.9215253 | 7.75E-06 | 0.000542656 |
| MED29 | -0.20743 | 7.8885628 | 7.83E-06 | 0.000546518 |
| MDH2 | -0.20978 | 11.371137 | 7.87E-06 | 0.00054742 |
| CRSP8P | -0.14977 | 7.6336272 | 8.03E-06 | 0.000556472 |
| CTSA | 0.36109 | 8.5158598 | 8.28E-06 | 0.000571628 |
| EXOSC2 | -0.13781 | 7.0689214 | 8.38E-06 | 0.000576681 |
| MYO9B | 0.244299 | 8.5827064 | 8.42E-06 | 0.000577636 |
| ROCK1 | 0.274337 | 8.3833711 | 8.46E-06 | 0.000578908 |
| UROS | -0.26747 | 8.2263407 | 8.82E-06 | 0.000601597 |
| INTS8 | 0.176045 | 8.3099237 | 8.88E-06 | 0.000602382 |
| PACSIN1 | -0.16289 | 7.0301514 | 8.92E-06 | 0.000602382 |
| ARID1A | -0.21932 | 9.4437927 | 8.92E-06 | 0.000602382 |

| R3HCC1 | -0.17786 | 7.7922467 | 9.05E-06 | 0.000609379 |
| --- | --- | --- | --- | --- |
| CACYBP | -0.21071 | 7.8271469 | 9.18E-06 | 0.000615152 |
| UNKL | 0.163266 | 7.4475285 | 9.20E-06 | 0.000615152 |
| HIGD1A | -0.1979 | 9.0675621 | 9.29E-06 | 0.000619126 |
| GIMAP1 | -0.28158 | 8.9854068 | 9.35E-06 | 0.000619761 |
| MRGBP | -0.13896 | 8.0329933 | 9.36E-06 | 0.000619761 |
| TSNAX | -0.19306 | 8.1425299 | 9.46E-06 | 0.000624865 |
| TCAF1 | 0.290477 | 14.060719 | 9.70E-06 | 0.000638267 |
| IL12RB1 | -0.17861 | 7.2893577 | 9.75E-06 | 0.000639743 |
| KCTD5 | -0.16161 | 8.6002267 | 9.82E-06 | 0.000642105 |
| ZNF281 | 0.322697 | 9.1946997 | 9.90E-06 | 0.000645129 |
| EP300 | 0.222687 | 7.7213121 | 9.93E-06 | 0.000645129 |
| DSTNP2 | -0.1637 | 7.3417924 | 9.97E-06 | 0.000646247 |
| ZNF577 | 0.399094 | 8.9462031 | 1.00E-05 | 0.000647536 |
| TDP1 | 0.443041 | 10.599984 | 1.01E-05 | 0.000648448 |
| DCTPP1 | -0.27315 | 8.3112831 | 1.01E-05 | 0.000650584 |
| EDEM1 | 0.174593 | 8.1419046 | 1.03E-05 | 0.000661385 |
| PHF5A | -0.17767 | 8.9818843 | 1.04E-05 | 0.000661385 |
| DYNLL2 | -0.11308 | 7.1770065 | 1.05E-05 | 0.000671029 |
| SHROOM | 0.456595 | 10.250514 | 1.06E-05 | 0.000673655 |
| GTPBP6 | -0.27095 | 9.1299123 | 1.07E-05 | 0.000673655 |
| PIGU | -0.17142 | 7.3798997 | 1.07E-05 | 0.000673655 |
| MTMR12 | 0.105241 | 14.921806 | 1.12E-05 | 0.000702593 |
| NBPF9 | 0.442528 | 11.639226 | 1.13E-05 | 0.000705281 |
| VPS16 | -0.22548 | 8.4075655 | 1.15E-05 | 0.000713768 |
| PMS2P5 | -0.13853 | 7.2060225 | 1.15E-05 | 0.000713768 |
| CTDNEP1 | 0.178181 | 7.60392 | 1.15E-05 | 0.000713768 |
| LSM2 | -0.2916 | 8.8885595 | 1.15E-05 | 0.000713768 |
| COX7A2 | -0.23014 | 10.643306 | 1.16E-05 | 0.000713768 |
| XRN1 | 0.179737 | 8.7820953 | 1.16E-05 | 0.000713768 |
| B4GALT7 | -0.18445 | 7.6377003 | 1.16E-05 | 0.000713768 |
| INTS9 | -0.1666 | 8.0805127 | 1.17E-05 | 0.000713768 |
| SPG11 | 0.207851 | 9.1969425 | 1.17E-05 | 0.000716928 |
| XPC | 0.189692 | 8.7386663 | 1.19E-05 | 0.0007228 |
| RNF19B | 0.243888 | 7.735075 | 1.19E-05 | 0.000723236 |
| SEC16A | 0.212114 | 8.8150721 | 1.21E-05 | 0.000729966 |
| METTL21 | 0.417876 | 12.41683 | 1.22E-05 | 0.00073551 |
| MRPL20 | -0.21952 | 8.0076573 | 1.22E-05 | 0.00073551 |
| FAU | -0.23334 | 11.907221 | 1.23E-05 | 0.00073551 |
| ZSWIM6 | 0.228283 | 8.1475828 | 1.26E-05 | 0.000752392 |
| FCAR | 0.438047 | 9.6806906 | 1.26E-05 | 0.000753492 |
| MICAL1 | 0.27223 | 8.3768559 | 1.30E-05 | 0.000770024 |
| UPF2 | 0.136334 | 9.6458599 | 1.31E-05 | 0.000773888 |
| TRAPPC8 | 0.152452 | 8.8322243 | 1.32E-05 | 0.000781583 |
| MFN2 | 0.241148 | 7.608823 | 1.33E-05 | 0.000784605 |
| LYST | 0.353983 | 9.168581 | 1.34E-05 | 0.000786109 |
| ARAP1 | 0.262154 | 8.2345877 | 1.35E-05 | 0.000793835 |
| TBC1D14 | 0.26758 | 8.9387416 | 1.36E-05 | 0.000796089 |
| DCTN2 | -0.14434 | 9.3090412 | 1.38E-05 | 0.000802493 |
| UFC1 | -0.2078 | 9.0266752 | 1.40E-05 | 0.00081142 |
| TYSND1 | -0.20771 | 8.0880702 | 1.40E-05 | 0.000811939 |
| N4BP2L2 | 0.298089 | 8.5512927 | 1.42E-05 | 0.0008176 |

| RPL17 | -0.3547 | 9.2363981 | 1.42E-05 | 0.0008176 |
| --- | --- | --- | --- | --- |
| ZNF821 | -0.11383 | 6.9959597 | 1.45E-05 | 0.0008328 |
| SBK1 | -0.31643 | 8.4283153 | 1.45E-05 | 0.000833456 |
| MYCBP2 | 0.235529 | 8.6488397 | 1.47E-05 | 0.000842505 |
| PPM1G | -0.22741 | 9.7205991 | 1.48E-05 | 0.000843538 |
| MVB12A | -0.15214 | 7.8050273 | 1.50E-05 | 0.000854275 |
| TGS1 | -0.14477 | 6.9751444 | 1.51E-05 | 0.000858231 |
| RTCB | -0.16142 | 7.4376214 | 1.52E-05 | 0.000858231 |
| DGCR2 | 0.218571 | 7.8292393 | 1.54E-05 | 0.000865979 |
| IMP4 | -0.26754 | 8.6542939 | 1.54E-05 | 0.000865979 |
| TMEM14B | -0.21916 | 9.1257764 | 1.55E-05 | 0.000867681 |
| HECA | 0.17485 | 8.9494329 | 1.56E-05 | 0.000874684 |
| RAB35 | -0.16725 | 8.3318009 | 1.58E-05 | 0.000882006 |
| NEDD8 | -0.15671 | 11.32112 | 1.61E-05 | 0.000898979 |
| ALG13 | -0.19022 | 7.5505999 | 1.66E-05 | 0.000918173 |
| POLR3GL | -0.23697 | 9.5509073 | 1.66E-05 | 0.000918173 |
| ZNF669 | 0.386085 | 8.4922907 | 1.66E-05 | 0.000918173 |
| ROCK1P1 | 0.159564 | 7.5118045 | 1.67E-05 | 0.000921668 |
| PTPN6 | 0.224364 | 9.6129552 | 1.68E-05 | 0.0009248 |
| MEM150 | -0.15944 | 7.5025995 | 1.69E-05 | 0.0009248 |
| SLC25A43 | -0.19353 | 7.3942262 | 1.69E-05 | 0.0009248 |
| LMOD3 | 0.472196 | 10.136556 | 1.70E-05 | 0.0009248 |
| RPL24 | -0.26269 | 12.369629 | 1.71E-05 | 0.000927734 |
| RAB9A | -0.17699 | 8.4757443 | 1.74E-05 | 0.000944194 |
| ACAP2 | 0.202935 | 8.8314801 | 1.75E-05 | 0.000947345 |
| EID2B | 0.404639 | 12.347861 | 1.77E-05 | 0.000953405 |
| SNIP1 | -0.14996 | 7.7483398 | 1.77E-05 | 0.000953405 |
| IGFBP7 | -0.21249 | 7.3908436 | 1.78E-05 | 0.000953408 |
| ABCF1 | -0.24319 | 8.9315381 | 1.79E-05 | 0.000959443 |
| ZMAT5 | -0.15297 | 7.4671175 | 1.80E-05 | 0.000962145 |
| ANPEP | 0.458354 | 8.4087005 | 1.81E-05 | 0.000962145 |
| ID2 | -0.40164 | 9.803535 | 1.83E-05 | 0.000972774 |
| AP5B1 | 0.308891 | 7.3561424 | 1.84E-05 | 0.000973341 |
| MED27 | -0.12321 | 7.2478178 | 1.85E-05 | 0.000975777 |
| TCEA1 | -0.25543 | 8.1103668 | 1.85E-05 | 0.000975777 |
| NBN | 0.235723 | 8.2236949 | 1.86E-05 | 0.000975777 |
| ZNF689 | -0.1734 | 8.0456811 | 1.87E-05 | 0.000979562 |
| TUT7 | 0.230298 | 7.8083212 | 1.87E-05 | 0.000979562 |
| C1001282 | 0.429338 | 11.359919 | 1.88E-05 | 0.000979562 |
| TIAL1 | 0.17397 | 8.5839576 | 1.89E-05 | 0.000982404 |
| NUP153 | 0.137879 | 7.6720227 | 1.91E-05 | 0.00099395 |
| PTAR1 | 0.145231 | 7.9355547 | 1.92E-05 | 0.000994469 |
| LRRFIP1 | 0.354665 | 11.01173 | 1.93E-05 | 0.000994469 |
| RAD17 | -0.09323 | 6.9397677 | 1.93E-05 | 0.000994469 |
| SCYL2 | 0.15092 | 8.5492662 | 1.94E-05 | 0.00099668 |
| C7orf50 | -0.25515 | 9.0244489 | 1.96E-05 | 0.001005318 |
| LSM1 | -0.16707 | 9.7664217 | 1.97E-05 | 0.001005578 |
| HSD17B7 | 0.399742 | 11.92733 | 1.97E-05 | 0.001007214 |
| DPM3 | -0.14117 | 7.3184698 | 1.98E-05 | 0.001008683 |
| TMED1 | -0.1525 | 7.9539785 | 2.03E-05 | 0.001030785 |
| PEA15 | -0.24667 | 8.6479495 | 2.03E-05 | 0.001030785 |
| FAM216A | -0.10248 | 7.0363708 | 2.05E-05 | 0.00103607 |

| SLIRP | -0.24782 | 9.8509989 | 2.09E-05 | 0.001054686 |
| --- | --- | --- | --- | --- |
| WASHC4 | 0.22566 | 7.9419684 | 2.11E-05 | 0.001062025 |
| CCDC102 | -0.18423 | 7.2422121 | 2.14E-05 | 0.001072346 |
| STK38 | 0.197734 | 9.7449954 | 2.18E-05 | 0.001089504 |
| RCN2 | -0.19355 | 7.9385812 | 2.18E-05 | 0.001089504 |
| ITGAM | 0.391385 | 10.034864 | 2.19E-05 | 0.001092367 |
| BSDC1 | 0.158632 | 8.9300204 | 2.20E-05 | 0.001092367 |
| PARK7 | -0.2746 | 8.71988 | 2.21E-05 | 0.001096612 |
| MAFF | 0.284581 | 7.5236318 | 2.24E-05 | 0.00110303 |
| TAPBP | 0.266794 | 9.7673333 | 2.24E-05 | 0.00110303 |
| PRELID1 | -0.22675 | 9.1318546 | 2.24E-05 | 0.00110303 |
| TBC1D2B | 0.151645 | 8.4155402 | 2.31E-05 | 0.001133683 |
| MED10P | -0.26711 | 9.1710049 | 2.32E-05 | 0.001138363 |
| SNHG10 | -0.16293 | 6.8428281 | 2.35E-05 | 0.001148059 |
| SSR2 | -0.24183 | 9.1924586 | 2.36E-05 | 0.001153765 |
| RPS7 | -0.36231 | 11.361307 | 2.38E-05 | 0.001155469 |
| MED10 | -0.15576 | 7.9994917 | 2.38E-05 | 0.001155469 |
| SYNGR2 | -0.2324 | 10.999744 | 2.38E-05 | 0.001155469 |
| TDRD1 | 0.318958 | 8.3649342 | 2.41E-05 | 0.00116395 |
| IQGAP1 | 0.324074 | 10.167473 | 2.42E-05 | 0.001165824 |
| SLC25A3 | -0.24406 | 10.712193 | 2.44E-05 | 0.001174938 |
| WTAP | 0.159965 | 9.3988826 | 2.49E-05 | 0.00119622 |
| ADCY10P | 0.163976 | 7.164132 | 2.50E-05 | 0.00119622 |
| WDR61 | -0.2044 | 8.095587 | 2.53E-05 | 0.001208144 |
| C5AR1 | 0.4681 | 10.160251 | 2.56E-05 | 0.001222818 |
| PPM1A | 0.257658 | 8.3719767 | 2.58E-05 | 0.001226443 |
| STXBP2 | 0.29448 | 9.4760608 | 2.59E-05 | 0.001228336 |
| CRYZL1 | -0.13674 | 7.4110625 | 2.61E-05 | 0.00123432 |
| GDPD1 | 0.357229 | 8.1974772 | 2.62E-05 | 0.001235348 |
| SLC9A8 | 0.241104 | 7.5975455 | 2.62E-05 | 0.001235348 |
| DDA1 | -0.22008 | 8.6247644 | 2.63E-05 | 0.001235348 |
| EMC4 | -0.22556 | 8.9851647 | 2.63E-05 | 0.001235348 |
| MTLN | -0.18291 | 7.337532 | 2.66E-05 | 0.001246132 |
| BASP1 | 0.495785 | 12.314105 | 2.68E-05 | 0.001249854 |
| PGAP3 | -0.16642 | 7.7625393 | 2.70E-05 | 0.001259524 |
| REPIN1 | -0.25473 | 8.736451 | 2.73E-05 | 0.001267561 |
| PLEKHF1 | -0.31961 | 7.9918547 | 2.74E-05 | 0.001269355 |
| PRRC2A | 0.177014 | 7.498005 | 2.75E-05 | 0.001269355 |
| SERTAD2 | -0.22652 | 9.6125171 | 2.75E-05 | 0.001269355 |
| TCF25 | -0.22074 | 9.3744662 | 2.76E-05 | 0.00127015 |
| HNRNPU | 0.545392 | 10.491551 | 2.82E-05 | 0.001298676 |
| PPP4R1 | 0.337822 | 9.7256137 | 2.83E-05 | 0.001299435 |
| NADK | 0.388145 | 8.7017559 | 2.85E-05 | 0.001306589 |
| LPIN2 | 0.293057 | 8.7489456 | 2.88E-05 | 0.001314109 |
| C16orf87 | -0.15264 | 7.1558317 | 2.89E-05 | 0.001318912 |
| RPS21 | -0.19999 | 7.7161041 | 2.91E-05 | 0.001323318 |
| MBD4 | 0.40033 | 10.632606 | 2.92E-05 | 0.001323566 |
| NAT1 | -0.12184 | 7.1062016 | 2.93E-05 | 0.001327849 |
| TBCE | -0.12602 | 7.5320449 | 2.94E-05 | 0.001328297 |
| COMT | -0.24277 | 8.8592435 | 2.94E-05 | 0.001328297 |
| PLEKHF2 | 0.217531 | 7.7979442 | 2.96E-05 | 0.001333139 |
| SBDSP1 | -0.10823 | 6.9677891 | 3.03E-05 | 0.0013588 |

| MICOS13 | -0.21372 | 8.4921526 | 3.06E-05 | 0.001371917 |
| --- | --- | --- | --- | --- |
| PPP1R12A | 0.215289 | 8.3497486 | 3.07E-05 | 0.001371917 |
| AIMP2 | -0.19525 | 8.0851214 | 3.12E-05 | 0.001393009 |
| SNU13 | -0.28186 | 9.4415919 | 3.15E-05 | 0.001396996 |
| SVIL | 0.342298 | 9.478166 | 3.15E-05 | 0.001396996 |
| RPS11 | -0.20688 | 13.184022 | 3.15E-05 | 0.001396996 |
| H2AFY | -0.21431 | 11.750068 | 3.16E-05 | 0.001397517 |
| NCOA3 | 0.183632 | 9.4909398 | 3.17E-05 | 0.001399566 |
| PCF11 | 0.134378 | 7.9141593 | 3.19E-05 | 0.001404157 |
| ARMCX6 | -0.15659 | 7.5350821 | 3.25E-05 | 0.001430736 |
| TLE3 | 0.176477 | 7.2254478 | 3.31E-05 | 0.001453333 |
| POFUT1 | 0.362596 | 12.944453 | 3.36E-05 | 0.001472764 |
| FKBP5 | 0.437133 | 11.278438 | 3.38E-05 | 0.001474721 |
| CCT2 | -0.20588 | 8.5454508 | 3.38E-05 | 0.001475432 |
| MAP3K11 | 0.213098 | 8.0944636 | 3.39E-05 | 0.001475432 |
| ADAMTS | -0.1062 | 6.7078854 | 3.43E-05 | 0.001488209 |
| NXT1 | -0.20319 | 7.8325696 | 3.44E-05 | 0.00149219 |
| MAP11 | 0.197061 | 7.6832873 | 3.48E-05 | 0.001503042 |
| RPL35A | -0.20888 | 12.973899 | 3.50E-05 | 0.001509774 |
| SETD2 | 0.141525 | 8.1795103 | 3.59E-05 | 0.001542682 |
| UBE2G2 | 0.370369 | 8.4155838 | 3.59E-05 | 0.001542682 |
| IFNAR2 | 0.278002 | 8.2115522 | 3.61E-05 | 0.00154741 |
| LINC0259 | 0.359964 | 12.541095 | 3.62E-05 | 0.001549109 |
| FAM43A | -0.19027 | 7.633956 | 3.65E-05 | 0.00155753 |
| KIAA0930 | -0.13413 | 7.1264308 | 3.66E-05 | 0.00155962 |
| PALM2 | 0.431217 | 10.793905 | 3.70E-05 | 0.001569971 |
| TMA7 | -0.17317 | 10.963165 | 3.70E-05 | 0.001569971 |
| TACC3 | 0.220568 | 8.5994552 | 3.71E-05 | 0.001569971 |
| RPE | -0.10614 | 7.1522173 | 3.75E-05 | 0.001587243 |
| MAN2A2 | 0.253323 | 7.6135047 | 3.79E-05 | 0.001598926 |
| SPAG9 | 0.233507 | 8.2869686 | 3.80E-05 | 0.001598926 |
| MRM2 | -0.11235 | 6.996281 | 3.82E-05 | 0.001606247 |
| MRPS11 | -0.17591 | 7.7843957 | 3.85E-05 | 0.00161529 |
| YWHAQ | -0.29842 | 10.86961 | 3.86E-05 | 0.001616031 |
| ZDHHC9 | -0.13108 | 7.026706 | 3.88E-05 | 0.001622749 |
| MSN | 0.296557 | 11.078408 | 3.92E-05 | 0.001632348 |
| BOP1 | -0.17089 | 7.9777072 | 3.93E-05 | 0.001635061 |
| PTPN2 | -0.15326 | 7.3615903 | 4.03E-05 | 0.001673034 |
| BRD8 | 0.148335 | 8.0612844 | 4.04E-05 | 0.001673034 |
| SNRPF | -0.33565 | 8.9317246 | 4.07E-05 | 0.001680457 |
| CD247 | -0.37938 | 11.160986 | 4.07E-05 | 0.001680457 |
| RNF24 | 0.406821 | 9.1970516 | 4.15E-05 | 0.001707847 |
| SAMD4B | 0.10615 | 6.9693855 | 4.15E-05 | 0.001707847 |
| SCARNA3 | 0.274968 | 7.5049399 | 4.16E-05 | 0.001708321 |
| NUP62 | -0.26132 | 9.9072446 | 4.18E-05 | 0.001711014 |
| VPS29 | -0.17499 | 9.620752 | 4.24E-05 | 0.001734519 |
| NRNPA1P | -0.37594 | 10.63231 | 4.25E-05 | 0.001734519 |
| EI24 | -0.19997 | 7.7784865 | 4.28E-05 | 0.001739816 |
| TRIM33 | 0.152816 | 8.951187 | 4.29E-05 | 0.001739816 |
| TUBA1B | -0.21583 | 11.554484 | 4.29E-05 | 0.001739816 |
| ARID3A | 0.289015 | 8.6149086 | 4.32E-05 | 0.001749819 |
| EMD | -0.19796 | 8.8912002 | 4.33E-05 | 0.001750565 |

| MRPL22 | -0.18321 | 8.1266522 | 4.42E-05 | 0.001784356 |
| --- | --- | --- | --- | --- |
| TOMM40 | -0.2271 | 8.1838123 | 4.44E-05 | 0.001785642 |
| PTGER2 | -0.24806 | 8.2853888 | 4.45E-05 | 0.001786957 |
| ZC3HC1 | -0.16254 | 7.9571719 | 4.50E-05 | 0.001799923 |
| RPL11 | -0.20129 | 13.957551 | 4.50E-05 | 0.001799923 |
| MXD1 | 0.421484 | 11.043182 | 4.51E-05 | 0.001799923 |
| SPCS2P4 | -0.28156 | 8.122613 | 4.53E-05 | 0.001807292 |
| GEMIN6 | -0.1395 | 7.4737709 | 4.60E-05 | 0.001822326 |
| SNRPB2 | -0.23674 | 9.387236 | 4.60E-05 | 0.001822326 |
| ANTXR2 | 0.288245 | 9.4843073 | 4.61E-05 | 0.001822326 |
| ITPR1 | 0.149867 | 7.7386071 | 4.61E-05 | 0.001822326 |
| SLC2A8 | -0.15998 | 7.1677781 | 4.61E-05 | 0.001822326 |
| BIRC3 | 0.367092 | 10.687788 | 4.66E-05 | 0.001836565 |
| PACS1 | 0.215875 | 7.5848072 | 4.71E-05 | 0.001852927 |
| SLX4IP | 0.14134 | 7.1258637 | 4.76E-05 | 0.001870209 |
| GLTP | -0.17291 | 9.7925007 | 4.81E-05 | 0.001885967 |
| PHACTR4 | -0.1787 | 8.0841376 | 4.83E-05 | 0.001889655 |
| GNPTG | -0.13246 | 7.7728381 | 4.84E-05 | 0.001890232 |
| COA3 | -0.20599 | 8.6483537 | 4.90E-05 | 0.001911139 |
| HUWE1 | 0.1665 | 7.2426099 | 4.95E-05 | 0.001926322 |
| HACL1 | -0.17799 | 7.988511 | 4.97E-05 | 0.001929305 |
| CFLAR | 0.35971 | 11.053039 | 5.08E-05 | 0.001968194 |
| POBEC3 | -0.32368 | 8.2440527 | 5.09E-05 | 0.001968194 |
| RGL2 | 0.22154 | 7.9517236 | 5.22E-05 | 0.002010831 |
| LEMD2 | -0.1282 | 7.4090827 | 5.22E-05 | 0.002010831 |
| TAOK1 | 0.139645 | 7.0344527 | 5.24E-05 | 0.002015006 |
| MAP3K2 | 0.279347 | 8.5082998 | 5.25E-05 | 0.00201757 |
| RAB8B | 0.192526 | 10.266525 | 5.32E-05 | 0.002039012 |
| MRPL11 | -0.22354 | 7.9094902 | 5.34E-05 | 0.00204197 |
| ALDOC | -0.20317 | 8.2073524 | 5.35E-05 | 0.00204197 |
| AKIRIN2 | 0.239531 | 9.4049008 | 5.38E-05 | 0.002052139 |
| EXOSC10 | -0.20557 | 8.6656388 | 5.47E-05 | 0.002082014 |
| RPSAP52 | -0.31942 | 12.631715 | 5.53E-05 | 0.00210218 |
| TOMM5 | -0.26367 | 8.1649553 | 5.58E-05 | 0.002116995 |
| BRAT1 | -0.26699 | 8.3690718 | 5.65E-05 | 0.00213807 |
| MIS18BP1 | 0.212934 | 8.715988 | 5.66E-05 | 0.00213807 |
| GPBP1L1 | 0.172612 | 8.1920808 | 5.67E-05 | 0.00213807 |
| MYO1F | 0.309271 | 8.5170076 | 5.71E-05 | 0.002145103 |
| PMVK | -0.12392 | 7.3022585 | 5.71E-05 | 0.002145103 |
| UQCRB | -0.14325 | 8.1552094 | 5.73E-05 | 0.002147886 |
| NOL7 | -0.21996 | 9.4051247 | 5.77E-05 | 0.002158177 |
| MARK2 | 0.190206 | 7.2392055 | 5.78E-05 | 0.002158177 |
| RPL12 | -0.27314 | 12.232747 | 5.78E-05 | 0.002158177 |
| RASSF2 | 0.36402 | 11.305184 | 5.81E-05 | 0.002164121 |
| DCK | -0.20404 | 8.1272838 | 5.90E-05 | 0.002194844 |
| KPNA1 | 0.143781 | 7.9510091 | 5.93E-05 | 0.002199442 |
| MRPS21 | -0.26455 | 9.135627 | 5.98E-05 | 0.002216464 |
| DBI | -0.22676 | 10.208539 | 6.01E-05 | 0.002222166 |
| TOMM22 | -0.22318 | 8.3222785 | 6.02E-05 | 0.002222529 |
| DERA | -0.14943 | 8.695413 | 6.07E-05 | 0.00223762 |
| PHF21A | 0.263754 | 9.9128392 | 6.09E-05 | 0.002238631 |
| LC2A4R | -0.14231 | 7.0207576 | 6.11E-05 | 0.002242654 |

| ADGRG5 | -0.27502 | 7.5891317 | 6.12E-05 | 0.002242654 |
| --- | --- | --- | --- | --- |
| GALT | -0.13896 | 7.1829368 | 6.14E-05 | 0.002242654 |
| MRPL37 | -0.20398 | 8.4972158 | 6.14E-05 | 0.002242654 |
| LSM8 | -0.13262 | 6.9652863 | 6.17E-05 | 0.002248686 |
| PRKD2 | 0.210077 | 8.2776012 | 6.20E-05 | 0.002258055 |
| ACP1 | -0.36113 | 9.1680159 | 6.28E-05 | 0.002280161 |
| EIF4G3 | 0.139009 | 7.7436893 | 6.35E-05 | 0.002304708 |
| ZNF850 | -0.08404 | 6.7773214 | 6.40E-05 | 0.002318096 |
| ATP5IF1 | -0.15601 | 7.981433 | 6.49E-05 | 0.002345686 |
| MRFAP1L | -0.20386 | 9.8469204 | 6.54E-05 | 0.002358774 |
| ADAM8 | 0.306035 | 9.3454336 | 6.57E-05 | 0.002362857 |
| PPP1CC | -0.17576 | 10.601225 | 6.58E-05 | 0.002362857 |
| SPG21 | -0.17824 | 7.8855788 | 6.58E-05 | 0.002362857 |
| RRC37BP | 0.377184 | 11.97023 | 6.64E-05 | 0.002378366 |
| AHCTF1 | 0.235021 | 8.14919 | 6.77E-05 | 0.002420847 |
| UBA1 | 0.245823 | 9.2007971 | 6.78E-05 | 0.002420847 |
| PSMD8 | -0.18494 | 7.7428091 | 6.80E-05 | 0.002420847 |
| TBCA | -0.24706 | 10.303444 | 6.80E-05 | 0.002420847 |
| MAP1LC3 | 0.324396 | 8.2485919 | 6.85E-05 | 0.002429484 |
| CLIC3 | -0.38515 | 7.9460496 | 6.87E-05 | 0.002429484 |
| EOMES | -0.47365 | 8.7350306 | 6.89E-05 | 0.002429484 |
| CXCL14 | 0.117541 | 6.9498216 | 6.90E-05 | 0.002429484 |
| APIP | -0.15222 | 7.6765603 | 6.90E-05 | 0.002429484 |
| KRTCAP2 | -0.25825 | 9.1282408 | 6.90E-05 | 0.002429484 |
| SERINC1 | 0.24888 | 9.2899215 | 6.91E-05 | 0.002429484 |
| MMP23B | -0.21322 | 7.072093 | 6.95E-05 | 0.002437418 |
| PRKACB | -0.11491 | 7.0119636 | 6.95E-05 | 0.002437418 |
| AMP2-AS | 0.328049 | 13.867199 | 6.97E-05 | 0.002439448 |
| TMEM41A | -0.11061 | 6.9336611 | 7.00E-05 | 0.002441774 |
| DMC1 | 0.382832 | 12.784925 | 7.00E-05 | 0.002441774 |
| BCKDHA | -0.17022 | 7.5636004 | 7.04E-05 | 0.002450859 |
| MTMR14 | -0.18128 | 8.5865873 | 7.05E-05 | 0.002452537 |
| HDHD5 | -0.19625 | 8.09163 | 7.11E-05 | 0.002465247 |
| ZNF671 | -0.14425 | 7.7642685 | 7.11E-05 | 0.002465247 |
| OSBPL8 | 0.305929 | 10.319428 | 7.16E-05 | 0.002475729 |
| KDM2A | 0.141041 | 8.4561727 | 7.18E-05 | 0.002479303 |
| RPS6KB2 | -0.23223 | 9.4620582 | 7.36E-05 | 0.002538445 |
| CALM3 | -0.26724 | 11.123398 | 7.43E-05 | 0.002557744 |
| BICRAL | 0.172807 | 8.8768607 | 7.47E-05 | 0.002562077 |
| SULT1A1 | 0.311457 | 11.976515 | 7.47E-05 | 0.002562077 |
| GEMIN4 | -0.21141 | 7.7671103 | 7.51E-05 | 0.002570669 |
| PDHB | -0.2217 | 9.8928358 | 7.53E-05 | 0.002570669 |
| KCTD20 | 0.167362 | 8.2161807 | 7.53E-05 | 0.002570669 |
| MAN2B2 | -0.16914 | 10.10362 | 7.57E-05 | 0.002580758 |
| HECTD1 | 0.129343 | 8.4307238 | 7.62E-05 | 0.002592294 |
| SH3BP5L | 0.162897 | 7.5640793 | 7.75E-05 | 0.002632666 |
| MLF2 | 0.197884 | 7.7735089 | 7.82E-05 | 0.002652848 |
| ALPK1 | 0.319584 | 8.04601 | 7.84E-05 | 0.002652848 |
| HNRNPL | 0.26842 | 8.0983633 | 7.85E-05 | 0.002652848 |
| ARAF | -0.20395 | 8.8191376 | 7.92E-05 | 0.002672311 |
| RPL27 | -0.25733 | 12.399081 | 7.94E-05 | 0.002672311 |
| STK40 | 0.270189 | 10.530405 | 7.95E-05 | 0.002672311 |

| BRD2 | -0.17451 | 10.186411 | 7.95E-05 | 0.002672311 |
| --- | --- | --- | --- | --- |
| TMED3 | -0.2527 | 8.893877 | 7.99E-05 | 0.002677209 |
| ZHX3 | -0.09813 | 6.893332 | 7.99E-05 | 0.002677209 |
| AKT1 | 0.233399 | 9.3559134 | 8.01E-05 | 0.002678427 |
| NIPBL | 0.181282 | 8.4327008 | 8.03E-05 | 0.002680463 |
| NKRD13 | 0.199423 | 10.207358 | 8.05E-05 | 0.002681264 |
| OCIAD1 | 0.364519 | 12.394159 | 8.08E-05 | 0.002688251 |
| CED1B-A | 0.129364 | 6.9391618 | 8.10E-05 | 0.002691142 |
| TSSC4 | -0.17276 | 8.4518708 | 8.12E-05 | 0.002691836 |
| HDAC1 | -0.25678 | 9.3794177 | 8.18E-05 | 0.002708467 |
| CHRNA5 | 0.373862 | 9.464698 | 8.21E-05 | 0.00271377 |
| TPK1-AS | 0.360995 | 12.754812 | 8.34E-05 | 0.002753578 |
| FIBP | -0.15703 | 8.1144363 | 8.39E-05 | 0.002766603 |
| FAM214B | 0.350577 | 8.556833 | 8.48E-05 | 0.002785798 |
| CNOT11 | -0.17858 | 9.139484 | 8.48E-05 | 0.002785798 |
| RNY3 | 0.229927 | 8.1371702 | 8.51E-05 | 0.002792134 |
| GZMA | -0.58042 | 10.316926 | 8.61E-05 | 0.002819999 |
| SEM1 | -0.28623 | 9.33696 | 8.65E-05 | 0.002829595 |
| CENPBD1 | -0.17894 | 7.8786695 | 8.71E-05 | 0.002844029 |
| DUSP28 | -0.12102 | 7.4071292 | 8.72E-05 | 0.00284424 |
| ZNF207 | -0.15387 | 8.9192405 | 8.76E-05 | 0.002846204 |
| TAF15 | 0.324171 | 9.1744978 | 8.76E-05 | 0.002846204 |
| C6orf47 | -0.138 | 7.8333492 | 8.77E-05 | 0.002846204 |
| FASLG | -0.16793 | 6.9825383 | 8.79E-05 | 0.002847944 |
| PAF1 | -0.12095 | 8.4440091 | 8.84E-05 | 0.002858398 |
| MRPL45P | -0.17663 | 7.3512354 | 8.86E-05 | 0.002861159 |
| PHOSPH | 0.177442 | 9.5276135 | 8.88E-05 | 0.002863983 |
| PTPMT1 | -0.17791 | 8.015196 | 8.97E-05 | 0.002889772 |
| CAPZA2 | 0.229068 | 10.048791 | 9.03E-05 | 0.002901974 |
| ETFA | -0.1924 | 8.9371683 | 9.05E-05 | 0.002901974 |
| FAR1 | 0.186896 | 8.1100004 | 9.05E-05 | 0.002901974 |
| ST8SIA4 | 0.250786 | 8.4499495 | 9.07E-05 | 0.002902589 |
| ERH | -0.23435 | 9.2286184 | 9.08E-05 | 0.002902589 |
| RAPGEF6 | 0.199877 | 8.4521546 | 9.17E-05 | 0.00292461 |
| PIGK | -0.15798 | 7.5268029 | 9.19E-05 | 0.002926086 |
| PDCD6 | -0.14063 | 9.7932135 | 9.21E-05 | 0.002929012 |
| PPID | -0.21376 | 7.1119272 | 9.24E-05 | 0.002934479 |
| OC72839 | 0.28601 | 8.5450338 | 9.25E-05 | 0.002934479 |
| GFM2 | -0.09611 | 6.8854896 | 9.31E-05 | 0.002946904 |
| RPL13A | -0.2705 | 13.529728 | 9.35E-05 | 0.002954875 |
| AGA | -0.15989 | 7.2753474 | 9.38E-05 | 0.002962229 |
| CFAP74 | 0.417988 | 11.315819 | 9.42E-05 | 0.002964548 |
| GPKOW | -0.16395 | 7.7815999 | 9.43E-05 | 0.002964548 |
| BEX4 | -0.26401 | 8.516035 | 9.44E-05 | 0.002964548 |
| OGA | 0.194746 | 10.832103 | 9.45E-05 | 0.002964548 |
| POLA2 | -0.1363 | 7.2033751 | 9.64E-05 | 0.00301397 |
| ADH5 | -0.17962 | 7.6624567 | 9.65E-05 | 0.00301397 |
| PHF20L1 | 0.195171 | 8.506385 | 9.65E-05 | 0.00301397 |
| ATG10 | 0.23487 | 8.5890888 | 9.66E-05 | 0.00301397 |
| C1orf109 | -0.09673 | 6.9478402 | 9.72E-05 | 0.00302678 |
| KDM6B | 0.167235 | 7.2465838 | 9.96E-05 | 0.003097204 |
| LAT2 | 0.305884 | 9.5922407 | 9.98E-05 | 0.003099644 |

| LENG8 | 0.080485 | 6.7855805 | 0.0001004 | 0.003112283 |
| --- | --- | --- | --- | --- |
| CMTM7 | -0.23076 | 10.499637 | 0.0001005 | 0.003112283 |
| MAP3K1 | 0.203487 | 10.844532 | 0.0001008 | 0.003114978 |
| GTF2B | -0.14329 | 8.2306388 | 0.0001021 | 0.003152078 |
| WDR33 | -0.16685 | 8.4327447 | 0.0001028 | 0.003167938 |
| SRSF10 | -0.18537 | 7.9786446 | 0.0001029 | 0.003167938 |
| GLT8D1 | -0.13336 | 7.6680723 | 0.0001037 | 0.003188316 |
| FLII | 0.162683 | 7.7744634 | 0.0001048 | 0.003210347 |
| TLN1 | 0.236856 | 9.6123094 | 0.0001048 | 0.003210347 |
| CARD8 | 0.302254 | 8.4264257 | 0.0001054 | 0.003224356 |
| EIPR1 | -0.12317 | 7.6542857 | 0.0001064 | 0.00324963 |
| PHIP | 0.14524 | 9.2882986 | 0.0001065 | 0.00324963 |
| UNC13D | 0.160679 | 7.4361609 | 0.0001067 | 0.00324996 |
| ACTB | 0.226484 | 13.816766 | 0.0001073 | 0.003262434 |
| TEDC1 | -0.13013 | 7.059389 | 0.0001074 | 0.003262434 |
| ATG4A | -0.10981 | 7.3796806 | 0.0001076 | 0.003262434 |
| PREX1 | 0.276638 | 7.9265724 | 0.000109 | 0.003300183 |
| HERPUD1 | -0.17335 | 9.0308439 | 0.0001104 | 0.003338683 |
| CENPT | 0.163004 | 7.6453084 | 0.0001109 | 0.003350747 |
| ZNF384 | 0.107396 | 7.1902989 | 0.0001113 | 0.003356674 |
| CHCHD3 | -0.10906 | 7.2810044 | 0.0001116 | 0.003360759 |
| COQ3 | -0.11506 | 7.109033 | 0.0001127 | 0.003388274 |
| PSMB1 | -0.18314 | 11.014275 | 0.000113 | 0.003392996 |
| XKR8 | 0.259635 | 8.0426971 | 0.0001132 | 0.003392996 |
| DIAPH1 | 0.196061 | 9.4600383 | 0.0001141 | 0.003415394 |
| NIT2 | -0.1222 | 6.9275517 | 0.0001163 | 0.003476474 |
| CPEB2 | 0.167216 | 7.3407033 | 0.0001165 | 0.003476474 |
| STAT3 | 0.284473 | 9.4707783 | 0.0001168 | 0.003476474 |
| TNFSF15 | 0.307995 | 8.2334552 | 0.0001169 | 0.003476474 |
| AUH | -0.09329 | 7.1454153 | 0.0001169 | 0.003476474 |
| RABL3 | -0.10522 | 7.0594502 | 0.0001173 | 0.003483007 |
| SLC19A1 | 0.220072 | 7.5824278 | 0.0001184 | 0.003509183 |
| HRF1BP1 | 0.139259 | 7.3577248 | 0.0001186 | 0.003510886 |
| USP15 | 0.234796 | 8.2064423 | 0.0001188 | 0.003512318 |
| COMMD6 | -0.2625 | 10.690055 | 0.0001195 | 0.003520113 |
| HOPX | -0.50876 | 8.7821276 | 0.0001195 | 0.003520113 |
| FBXO8 | -0.13487 | 7.8927667 | 0.0001196 | 0.003520113 |
| TMEM60 | -0.15141 | 8.5761857 | 0.0001215 | 0.003572676 |
| CPT1B | 0.168419 | 7.1079569 | 0.0001217 | 0.003572676 |
| IL17RA | 0.317653 | 8.7258425 | 0.0001221 | 0.003577994 |
| MBOAT2 | 0.247892 | 7.647963 | 0.0001226 | 0.003587537 |
| SAFB | -0.2274 | 8.5349369 | 0.0001229 | 0.003587537 |
| CXXC5 | -0.30829 | 9.2639584 | 0.0001229 | 0.003587537 |
| DCP2 | 0.169358 | 10.029811 | 0.0001232 | 0.003590207 |
| ATP5MC2 | -0.24132 | 10.695826 | 0.0001238 | 0.003604007 |
| CDC42 | 0.183008 | 9.5712417 | 0.0001241 | 0.003608166 |
| DDX3X | 0.248743 | 10.171253 | 0.0001244 | 0.003611971 |
| GORASP1 | 0.133063 | 7.2455579 | 0.0001261 | 0.003656091 |
| GNL1 | -0.13004 | 7.4318371 | 0.0001277 | 0.003691647 |
| PLPPR2 | 0.247134 | 7.6597698 | 0.0001277 | 0.003691647 |
| BMS1P1 | 0.36584 | 12.140529 | 0.0001286 | 0.003713779 |
| MED20 | -0.14806 | 7.9657874 | 0.0001291 | 0.003722142 |

| MRTO4 | -0.10231 | 6.9501899 | 0.0001297 | 0.003733791 |
| --- | --- | --- | --- | --- |
| ARRB1 | -0.22426 | 8.5859876 | 0.0001306 | 0.003749615 |
| DDX47 | -0.23001 | 8.7828306 | 0.0001306 | 0.003749615 |
| SENP6 | 0.130852 | 8.0169147 | 0.0001331 | 0.003814567 |
| MINDY1 | 0.283593 | 9.5869687 | 0.0001334 | 0.003814567 |
| OTUD5 | 0.122946 | 7.3559603 | 0.0001338 | 0.003814567 |
| S1PR4 | 0.233117 | 9.6272608 | 0.0001338 | 0.003814567 |
| BTBD2 | -0.19287 | 9.7089066 | 0.0001339 | 0.003814567 |
| SAMM50 | -0.20501 | 8.2287257 | 0.000134 | 0.003814567 |
| DTWD2 | 0.407552 | 11.301951 | 0.0001345 | 0.003825655 |
| RPGR | 0.206671 | 7.6323301 | 0.0001351 | 0.003836542 |
| RCC2 | -0.21538 | 9.1304735 | 0.0001359 | 0.003854287 |
| SYNJ1 | 0.189852 | 8.4722534 | 0.0001361 | 0.003854626 |
| KANSL2 | -0.16133 | 8.3988962 | 0.0001376 | 0.00389135 |
| BLZF1 | 0.375208 | 12.310235 | 0.0001379 | 0.003893437 |
| FOXK1 | 0.385306 | 12.773714 | 0.000138 | 0.003893437 |
| DPP7 | -0.25065 | 8.4178133 | 0.0001389 | 0.003910171 |
| SH2B3 | 0.220418 | 9.497398 | 0.000139 | 0.003910171 |
| NLRP1 | 0.140642 | 7.1213042 | 0.0001393 | 0.0039144 |
| ARL2 | -0.21266 | 8.2818562 | 0.0001399 | 0.003924394 |
| PIGC | -0.11796 | 8.3767701 | 0.0001405 | 0.003936698 |
| HAUS7 | -0.12616 | 7.6732854 | 0.0001408 | 0.003939255 |
| FAM32A | -0.15706 | 8.988362 | 0.0001414 | 0.003945189 |
| DNAJB14 | 0.159149 | 8.4604212 | 0.0001414 | 0.003945189 |
| LINC0066 | 0.36663 | 9.9612005 | 0.0001423 | 0.003966133 |
| ZNF12 | 0.157442 | 7.5702257 | 0.0001431 | 0.003981528 |
| CD7 | -0.37139 | 9.4896748 | 0.0001434 | 0.003986848 |
| SLC25A5 | -0.21063 | 11.806395 | 0.0001441 | 0.004000914 |
| NMT2 | 0.413831 | 11.367723 | 0.0001449 | 0.004015454 |
| FAM162A | -0.17262 | 7.495294 | 0.0001454 | 0.004015454 |
| MUL1 | -0.1825 | 8.4421591 | 0.0001454 | 0.004015454 |
| MRPL10 | -0.14734 | 7.8235951 | 0.0001454 | 0.004015454 |
| GNPDA2 | -0.12457 | 7.2616744 | 0.0001468 | 0.004048318 |
| GNL2 | -0.19738 | 8.286784 | 0.0001473 | 0.004055492 |
| DOLPP1 | -0.10153 | 7.0102519 | 0.0001483 | 0.004072389 |
| TARP | -0.45603 | 7.9624664 | 0.0001484 | 0.004072389 |
| WAC | 0.177482 | 10.685607 | 0.0001485 | 0.004072389 |
| MDM1 | -0.08865 | 6.8674709 | 0.0001503 | 0.004117752 |
| PXN | 0.208488 | 7.9568366 | 0.0001506 | 0.004120714 |
| SMIM11A | -0.13707 | 7.3615228 | 0.0001509 | 0.004123026 |
| RNF220 | -0.15509 | 7.9935776 | 0.0001514 | 0.004131551 |
| OSGEP | -0.11823 | 7.533448 | 0.0001517 | 0.004133315 |
| NSMCE1 | -0.17147 | 7.9300782 | 0.0001543 | 0.004199187 |
| TMEM258 | -0.22234 | 10.733867 | 0.0001568 | 0.004260874 |
| NP32A-IT | 0.211751 | 7.579508 | 0.0001592 | 0.004321471 |
| HIC2 | -0.1063 | 7.3006344 | 0.00016 | 0.004335715 |
| PKM | 0.275852 | 8.7202032 | 0.0001603 | 0.004335715 |
| RPUSD2 | -0.18653 | 7.7148285 | 0.0001603 | 0.004335715 |
| TSPAN31 | -0.14476 | 7.7299346 | 0.0001622 | 0.004381001 |
| CARD11 | -0.30491 | 8.7305923 | 0.0001627 | 0.004385345 |
| STX6 | 0.17043 | 8.2572843 | 0.0001628 | 0.004385345 |
| UBR5 | 0.113848 | 7.8489593 | 0.000163 | 0.004385345 |

| SARNP | -0.15618 | 8.9708794 | 0.0001633 | 0.004387926 |
| --- | --- | --- | --- | --- |
| KLRF1 | -0.56422 | 9.0945969 | 0.0001635 | 0.004387926 |
| NPEPPS | 0.234442 | 8.4073258 | 0.000164 | 0.004394275 |
| ATP2A3 | 0.157834 | 7.590101 | 0.0001658 | 0.004438328 |
| FAM126B | 0.300393 | 12.894158 | 0.0001672 | 0.004469779 |
| RIOK2 | -0.14895 | 7.5108963 | 0.000168 | 0.004484839 |
| KHNYN | 0.15293 | 7.7911165 | 0.0001684 | 0.004484839 |
| TBC1D4 | 0.191848 | 7.4911545 | 0.0001684 | 0.004484839 |
| SLC27A3 | -0.23382 | 8.9905326 | 0.000169 | 0.004491259 |
| GHITM | -0.13325 | 10.925177 | 0.0001691 | 0.004491259 |
| FBXL3 | 0.167439 | 8.1961942 | 0.0001696 | 0.004498592 |
| ATP6AP1 | -0.18474 | 10.509002 | 0.0001702 | 0.004509979 |
| TNFAIP2 | 0.225309 | 7.5380862 | 0.0001705 | 0.004510806 |
| MTFP1 | -0.20719 | 7.3065191 | 0.0001719 | 0.004542699 |
| INPP5A | 0.199016 | 7.6595527 | 0.0001725 | 0.004553234 |
| GNMT | 0.093822 | 6.7340862 | 0.0001735 | 0.004573764 |
| DIS3L | -0.20954 | 8.1219859 | 0.0001751 | 0.004603972 |
| SUSD6 | 0.263987 | 8.838278 | 0.0001752 | 0.004603972 |
| TSEN15 | -0.17951 | 7.8278518 | 0.0001753 | 0.004603972 |
| STN1 | -0.15659 | 8.0273377 | 0.000176 | 0.004615326 |
| SHOC2 | 0.151182 | 9.0978457 | 0.0001767 | 0.004628298 |
| NDUFB2 | -0.23929 | 9.8813169 | 0.0001776 | 0.004646547 |
| NRBP1 | 0.105083 | 7.1620946 | 0.0001785 | 0.004663104 |
| CKS1B | -0.17383 | 7.3391905 | 0.0001789 | 0.004668071 |
| AKR7A2 | -0.19231 | 8.9585232 | 0.00018 | 0.00469043 |
| NDUFA8 | -0.1697 | 8.7213772 | 0.000181 | 0.004709525 |
| NPIPB13 | 0.308725 | 10.904684 | 0.0001811 | 0.004709525 |
| GMIP | 0.191612 | 7.9719343 | 0.0001816 | 0.004713951 |
| ILVBL | -0.14336 | 7.366806 | 0.0001818 | 0.004713951 |
| MRPL32 | -0.19405 | 8.5421302 | 0.0001824 | 0.004716777 |
| QARS | -0.22585 | 10.328967 | 0.0001825 | 0.004716777 |
| AGPAT1 | 0.157989 | 7.2855653 | 0.0001825 | 0.004716777 |
| MRFAP1 | -0.1332 | 9.7729204 | 0.0001834 | 0.004732769 |
| RRAGA | -0.10901 | 9.0873679 | 0.000185 | 0.004768795 |
| MAPRE1 | -0.12808 | 9.9125193 | 0.0001856 | 0.004778251 |
| UFSP2 | -0.14373 | 7.3163897 | 0.0001865 | 0.004796437 |
| SEPTIN2 | -0.16894 | 10.602287 | 0.0001869 | 0.004800239 |
| AKAP5 | 0.248009 | 13.703739 | 0.0001881 | 0.004825934 |
| PDZD8 | 0.163039 | 7.3898005 | 0.0001896 | 0.004858075 |
| SERPINB | -0.2345 | 9.4274257 | 0.0001924 | 0.004917282 |
| MAGED1 | -0.26887 | 8.3409595 | 0.0001924 | 0.004917282 |
| VAMP8 | -0.25333 | 10.138134 | 0.0001928 | 0.004921861 |
| SKAP1 | -0.33001 | 9.7918937 | 0.0001969 | 0.005018934 |
| ARRB2 | 0.269941 | 7.8880651 | 0.0001984 | 0.005052004 |
| COMMD1 | -0.15472 | 7.8724446 | 0.0001989 | 0.005059296 |
| PSMB10 | -0.19696 | 11.484131 | 0.0001995 | 0.005066799 |
| SIVA1 | -0.17334 | 8.603208 | 0.0002001 | 0.005075824 |
| CLASP1 | 0.128991 | 7.6422635 | 0.0002006 | 0.00508198 |
| VPS8 | 0.164342 | 8.0438943 | 0.0002009 | 0.005083267 |
| RGS2 | 0.367653 | 13.373377 | 0.000203 | 0.005121446 |
| WBP1L | 0.106039 | 7.3037493 | 0.0002031 | 0.005121446 |
| SAYSD1 | -0.12971 | 7.0247156 | 0.0002032 | 0.005121446 |

| MRPS26 | -0.16129 | 7.5739729 | 0.0002033 | 0.005121446 |
| --- | --- | --- | --- | --- |
| RPL22 | -0.30503 | 11.868396 | 0.0002037 | 0.005123562 |
| SRPK1 | 0.310092 | 8.6990713 | 0.0002045 | 0.00513934 |
| EPHB1 | 0.169255 | 7.0423688 | 0.0002065 | 0.005181317 |
| TMEM39B | 0.157558 | 7.7744671 | 0.0002072 | 0.005188702 |
| C12orf10 | -0.20939 | 8.7144907 | 0.0002075 | 0.005188702 |
| UVSSA | 0.171334 | 7.4850971 | 0.0002075 | 0.005188702 |
| SEPHS2 | -0.14259 | 8.168644 | 0.0002087 | 0.005206779 |
| RPL9 | -0.28784 | 12.832963 | 0.0002087 | 0.005206779 |
| AKR1C3 | -0.34104 | 7.6893564 | 0.000209 | 0.005207707 |
| MYD88 | 0.212284 | 8.8549877 | 0.0002096 | 0.005212625 |
| TSPAN3 | -0.22333 | 7.6813145 | 0.0002097 | 0.005212625 |
| HS6ST1 | -0.11892 | 7.2033432 | 0.00021 | 0.005212625 |
| HDAC7 | 0.132074 | 7.232337 | 0.0002103 | 0.005213916 |
| AGFG1 | 0.14488 | 7.6495501 | 0.0002112 | 0.005231233 |
| GVINP1 | 0.354848 | 9.7739433 | 0.0002117 | 0.005237046 |
| RXYLT1 | -0.11605 | 7.5038267 | 0.0002138 | 0.005283961 |
| USP6 | 0.175442 | 7.2103552 | 0.0002155 | 0.005306065 |
| RPL14 | -0.37148 | 11.116175 | 0.0002155 | 0.005306065 |
| ZNF876P | 0.28256 | 13.576568 | 0.0002155 | 0.005306065 |
| PSMC6 | -0.18935 | 8.9973791 | 0.0002185 | 0.00537414 |
| PSMA6 | -0.21065 | 10.543112 | 0.0002196 | 0.005386877 |
| LTN1 | 0.114918 | 7.6081559 | 0.0002197 | 0.005386877 |
| TMCO1 | -0.18081 | 9.5090883 | 0.00022 | 0.005386877 |
| BICD2 | 0.219945 | 8.7582232 | 0.0002201 | 0.005386877 |
| CUTA | -0.29191 | 9.7401745 | 0.000222 | 0.0054272 |
| SNAP23 | 0.255817 | 9.4286937 | 0.0002231 | 0.005442125 |
| RPLP0 | -0.32028 | 12.39468 | 0.0002231 | 0.005442125 |
| RXRB | -0.186 | 8.4173526 | 0.0002239 | 0.005454257 |
| ZC3H4 | 0.159889 | 8.7951154 | 0.000225 | 0.005475306 |
| MGRN1 | 0.187227 | 7.7470444 | 0.0002262 | 0.005499544 |
| LSM4 | -0.16096 | 7.8694369 | 0.0002266 | 0.005500346 |
| NPRL2 | -0.16574 | 8.8031854 | 0.0002269 | 0.005500346 |
| UBIAD1 | -0.16127 | 7.6171207 | 0.0002271 | 0.005500346 |
| BCDIN3D | -0.12508 | 7.5853263 | 0.0002277 | 0.005508674 |
| PPFIA1 | 0.177665 | 8.4199532 | 0.000228 | 0.005509901 |
| UBE3B | 0.117447 | 7.3632067 | 0.0002286 | 0.005518721 |
| UGGT1 | 0.160538 | 7.1409336 | 0.0002325 | 0.00560202 |
| RNF126 | -0.21233 | 7.967204 | 0.0002326 | 0.00560202 |
| MEPCE | -0.18158 | 7.925382 | 0.0002337 | 0.00561951 |
| DNLZ | -0.27688 | 7.8654004 | 0.0002339 | 0.00561951 |
| BRI3 | 0.288745 | 10.09078 | 0.0002351 | 0.005643177 |
| STX5 | -0.14508 | 9.2941376 | 0.0002364 | 0.005668173 |
| TARDBP | 0.129751 | 7.3171741 | 0.0002375 | 0.005682957 |
| UBE4B | 0.125463 | 7.9998896 | 0.0002379 | 0.005682957 |
| SLC40A1 | 0.297392 | 9.4074349 | 0.0002379 | 0.005682957 |
| HECTD4 | 0.198206 | 7.7996175 | 0.0002382 | 0.005682957 |
| GNA13 | 0.140704 | 9.9068132 | 0.0002384 | 0.005682957 |
| RBMX | -0.16526 | 7.9933595 | 0.0002409 | 0.005736193 |
| BRAXAS | 0.393694 | 11.37928 | 0.000242 | 0.005755773 |
| ALKBH5 | -0.17333 | 10.693591 | 0.0002428 | 0.005766818 |
| DEXI | -0.23583 | 8.6999587 | 0.000243 | 0.005766818 |

| CBLL1 | -0.14313 | 8.5373196 | 0.000244 | 0.005783267 |
| --- | --- | --- | --- | --- |
| GLYR1 | 0.165885 | 8.1331396 | 0.0002446 | 0.005790512 |
| SZRD1 | -0.14079 | 8.5785831 | 0.0002456 | 0.005803427 |
| CDC26 | -0.15552 | 9.2903208 | 0.0002457 | 0.005803427 |
| UBXN2B | 0.255577 | 8.0792307 | 0.0002462 | 0.005809407 |
| GPS1 | -0.15798 | 8.6918493 | 0.0002469 | 0.005819785 |
| LRMP | 0.189253 | 9.4231161 | 0.0002477 | 0.005829475 |
| RBBP4 | -0.22578 | 8.0214072 | 0.0002481 | 0.005829475 |
| ORC4 | 0.257512 | 7.848429 | 0.0002482 | 0.005829475 |
| QRFPR | 0.382989 | 10.294869 | 0.0002485 | 0.005831182 |
| NXF1 | 0.160897 | 8.7400345 | 0.0002499 | 0.005856245 |
| COX14 | -0.14604 | 7.4660561 | 0.0002504 | 0.005862753 |
| MYO3B | 0.294199 | 8.04419 | 0.0002525 | 0.005904685 |
| METTL1 | -0.13832 | 7.1525589 | 0.0002534 | 0.005919111 |
| NRNPUL | -0.15337 | 9.6972116 | 0.0002553 | 0.00595679 |
| EIF4G2 | 0.161896 | 12.059055 | 0.0002557 | 0.005958798 |
| SLC25A19 | -0.13114 | 7.2796571 | 0.0002575 | 0.005995859 |
| CHN2 | -0.14065 | 7.3530687 | 0.000259 | 0.006023092 |
| BRK1 | -0.19348 | 8.3294611 | 0.0002611 | 0.006065537 |
| BCKDK | -0.16347 | 8.7704755 | 0.0002616 | 0.006069911 |
| HSPB11 | -0.1592 | 7.6718296 | 0.0002621 | 0.006074674 |
| KHSRP | -0.13896 | 7.5689337 | 0.0002625 | 0.006077968 |
| F13A1 | 0.490105 | 9.5001785 | 0.0002635 | 0.00609107 |
| POTEF | 0.23078 | 13.1251 | 0.0002637 | 0.00609107 |
| FCHO1 | 0.117457 | 7.3103483 | 0.0002648 | 0.006109848 |
| DDX46 | -0.12285 | 7.6242722 | 0.0002676 | 0.006160874 |
| MOCS2 | -0.11262 | 6.8704517 | 0.0002677 | 0.006160874 |
| YIF1A | -0.15571 | 7.4401307 | 0.0002679 | 0.006160874 |
| NBPF3 | 0.194765 | 8.5365827 | 0.0002699 | 0.006201242 |
| GNAI2 | 0.217791 | 10.485961 | 0.0002702 | 0.006201242 |
| IGF1R | 0.256322 | 7.7260917 | 0.000271 | 0.006212267 |
| AK2 | -0.17532 | 8.1464974 | 0.000272 | 0.006222387 |
| MCL1 | 0.206075 | 10.968242 | 0.0002723 | 0.006222387 |
| TMEM115 | -0.11024 | 7.5716954 | 0.0002724 | 0.006222387 |
| DCTN6 | -0.14017 | 7.6040535 | 0.000273 | 0.006222387 |
| EEF1E1 | -0.17935 | 7.9266282 | 0.0002735 | 0.006222387 |
| PJA2 | 0.235991 | 11.111762 | 0.0002737 | 0.006222387 |
| ATP5PB | -0.21314 | 10.506828 | 0.0002738 | 0.006222387 |
| PLAC8 | -0.3004 | 10.037891 | 0.0002739 | 0.006222387 |
| MCEE | -0.15243 | 7.5379542 | 0.0002741 | 0.006222387 |
| DUSP19 | 0.335231 | 11.735664 | 0.0002744 | 0.00622311 |
| PISD | 0.294731 | 8.6990302 | 0.0002753 | 0.006235219 |
| NDUFB5 | -0.22165 | 9.8456341 | 0.0002773 | 0.006273551 |
| TMEM242 | -0.1404 | 7.4257091 | 0.0002778 | 0.006278426 |
| SRRT | -0.16207 | 9.728974 | 0.0002784 | 0.006286606 |
| SMIM19 | -0.15714 | 7.867514 | 0.0002815 | 0.006336412 |
| NUP35 | -0.09633 | 7.01638 | 0.0002816 | 0.006336412 |
| NCR3 | -0.19711 | 7.1969625 | 0.0002819 | 0.006336412 |
| PSMC3 | -0.17279 | 7.9973252 | 0.0002821 | 0.006336412 |
| AIFM1 | -0.13219 | 7.7352653 | 0.0002822 | 0.006336412 |
| RAB33B | 0.197616 | 8.8063706 | 0.0002828 | 0.006336533 |
| MED19 | -0.12556 | 7.5170733 | 0.0002828 | 0.006336533 |

| NSDHL | -0.12573 | 7.0966115 | 0.0002833 | 0.006341888 |
| --- | --- | --- | --- | --- |
| GLG1 | -0.18965 | 10.048845 | 0.0002838 | 0.006345347 |
| ELP6 | -0.13465 | 7.4649328 | 0.0002869 | 0.00640819 |
| NRGN | 0.643854 | 10.23381 | 0.000288 | 0.006426406 |
| TRIP12 | 0.267903 | 9.6642999 | 0.0002925 | 0.006519988 |
| ZNF557 | 0.271621 | 8.5815061 | 0.0002935 | 0.006521004 |
| ATP6V0E | -0.3269 | 9.1056675 | 0.0002936 | 0.006521004 |
| GLO1 | -0.21707 | 8.5439217 | 0.0002936 | 0.006521004 |
| UBE2N | -0.21806 | 8.7334395 | 0.0002938 | 0.006521004 |
| MRPL47 | -0.11412 | 7.1394081 | 0.0002954 | 0.006549654 |
| CRBN | -0.19136 | 9.2509618 | 0.0002963 | 0.006560796 |
| TMEM205 | -0.19119 | 8.5642502 | 0.0002965 | 0.006560796 |
| NDST2 | -0.13705 | 7.7178431 | 0.0002973 | 0.006571035 |
| UBALD2 | 0.220118 | 7.9717834 | 0.0002977 | 0.006572859 |
| SRSF3 | -0.20441 | 9.603401 | 0.0002986 | 0.006576918 |
| NDUFC2 | -0.08536 | 6.7997784 | 0.0002988 | 0.006576918 |
| RUVBL2 | -0.11912 | 7.4895149 | 0.0002988 | 0.006576918 |
| SNAPIN | -0.11359 | 7.4052989 | 0.0002998 | 0.006591934 |
| UBR4 | 0.159284 | 8.4786321 | 0.0003028 | 0.006651069 |
| RPL21P44 | 0.102092 | 6.9242423 | 0.0003056 | 0.006692067 |
| ZBTB34 | 0.227797 | 8.3753812 | 0.0003056 | 0.006692067 |
| RUBCNL | 0.256532 | 8.0756934 | 0.0003057 | 0.006692067 |
| FURIN | 0.224642 | 7.3217767 | 0.0003114 | 0.006809653 |
| SPI1 | 0.42732 | 9.9346966 | 0.0003118 | 0.006811074 |
| INPP1 | -0.14011 | 7.4330844 | 0.000313 | 0.006830957 |
| TOMM34 | -0.1471 | 7.7771222 | 0.0003134 | 0.006830957 |
| RNF121 | -0.10579 | 7.4953134 | 0.0003136 | 0.006830957 |
| STX8 | -0.15969 | 7.9718009 | 0.0003149 | 0.006850777 |
| MRPL42 | -0.11763 | 7.3374003 | 0.0003161 | 0.006870472 |
| OPA3 | -0.11922 | 7.0379409 | 0.0003165 | 0.006872755 |
| TMEM230 | -0.21555 | 9.8838651 | 0.0003178 | 0.006891801 |
| TRAPPC2 | -0.20088 | 8.3559085 | 0.0003195 | 0.006922634 |
| SNX8 | -0.11438 | 7.3380355 | 0.0003247 | 0.007027263 |
| TNF | -0.15678 | 7.5446873 | 0.0003251 | 0.007027263 |
| PPM1K | 0.291523 | 8.3766558 | 0.0003253 | 0.007027263 |
| HIGD2A | -0.24442 | 9.7722394 | 0.0003292 | 0.007102105 |
| TAP2 | 0.222059 | 8.2494648 | 0.0003295 | 0.007102105 |
| KLHDC2 | -0.15403 | 8.6509826 | 0.0003302 | 0.00710945 |
| HCP5 | -0.21759 | 10.717291 | 0.0003326 | 0.007154351 |
| CAMLG | -0.14117 | 10.180495 | 0.0003331 | 0.007156936 |
| EIF3H | -0.2274 | 9.7379495 | 0.0003337 | 0.0071628 |
| MED8 | -0.10632 | 7.1759869 | 0.0003345 | 0.007172548 |
| EIF2B5 | -0.13048 | 6.9773081 | 0.0003349 | 0.007174361 |
| PRPF3 | 0.145813 | 8.3251618 | 0.0003352 | 0.007174361 |
| NR1H3 | -0.09972 | 7.0049361 | 0.0003371 | 0.007203924 |
| RASSF4 | -0.12477 | 7.0886397 | 0.0003373 | 0.007203924 |
| ECI2 | -0.20308 | 8.4797416 | 0.000338 | 0.007204234 |
| FBL | -0.32902 | 9.6159745 | 0.000338 | 0.007204234 |
| SUMO3 | -0.20761 | 9.6649772 | 0.0003423 | 0.007288697 |
| RNF44 | 0.121067 | 8.7389746 | 0.0003429 | 0.007292752 |
| OXP1-IT | 0.204225 | 7.5728431 | 0.0003442 | 0.007314207 |
| DECR1 | -0.17223 | 10.256924 | 0.000345 | 0.007322713 |

| COX4I1 | -0.17917 | 11.972413 | 0.0003453 | 0.007322713 |
| --- | --- | --- | --- | --- |
| AP1B1 | -0.16021 | 8.011401 | 0.0003481 | 0.007373287 |
| BCL6B | -0.06315 | 6.7298465 | 0.0003484 | 0.007373287 |
| TBC1D19 | -0.07267 | 6.7504036 | 0.0003505 | 0.007410889 |
| FUT6 | 0.183489 | 7.4079408 | 0.0003516 | 0.007426693 |
| SEMA4B | 0.193769 | 7.5687201 | 0.0003524 | 0.007434489 |
| E4F1 | -0.21305 | 8.5117764 | 0.0003528 | 0.007436911 |
| USP8 | 0.121425 | 8.5310405 | 0.0003539 | 0.007451834 |
| MBD6 | 0.293301 | 9.3419072 | 0.0003549 | 0.007464913 |
| COTL1 | 0.281097 | 9.2273762 | 0.0003553 | 0.007464913 |
| PYGL | 0.41562 | 10.54147 | 0.0003556 | 0.007464913 |
| RPL26L1 | -0.14409 | 7.9405897 | 0.0003565 | 0.007476525 |
| RAPPC2 | -0.14488 | 6.9972932 | 0.0003569 | 0.007478196 |
| EHF | -0.06604 | 6.6565954 | 0.0003591 | 0.007514518 |
| ZEB2 | 0.203194 | 8.0022081 | 0.0003594 | 0.007514518 |
| RPL29 | -0.2607 | 12.024388 | 0.0003606 | 0.007532743 |
| PDP1 | 0.142131 | 7.4650378 | 0.0003632 | 0.007578113 |
| THOC7 | -0.15173 | 9.7701557 | 0.0003636 | 0.00758053 |
| RAN | -0.26752 | 9.7854655 | 0.0003647 | 0.007595156 |
| MRPS12 | -0.2209 | 8.9036954 | 0.0003668 | 0.007630554 |
| ZFP90 | -0.20962 | 7.9366509 | 0.0003685 | 0.007659027 |
| UGP2 | -0.1519 | 9.6875447 | 0.0003698 | 0.007678352 |
| CD2BP2 | -0.16402 | 9.1533448 | 0.000371 | 0.007695793 |
| EXOSC7 | -0.15663 | 7.4597174 | 0.0003727 | 0.007722903 |
| COX6C | -0.29601 | 10.632187 | 0.0003738 | 0.007734775 |
| YPEL5 | 0.193396 | 10.117636 | 0.000374 | 0.007734775 |
| SMARCA | 0.152915 | 8.2245889 | 0.0003752 | 0.007753126 |
| KCNJ2 | 0.369372 | 8.5874723 | 0.0003775 | 0.007792998 |
| CLK4 | 0.181443 | 7.6886755 | 0.0003849 | 0.007936987 |
| CDK14 | 0.101409 | 6.9704621 | 0.0003875 | 0.007981948 |
| FAM160B | 0.209561 | 9.060505 | 0.0003908 | 0.008032367 |
| ITGA2B | 0.540919 | 7.9394513 | 0.0003909 | 0.008032367 |
| RHGAP2 | 0.130759 | 7.0411377 | 0.0003914 | 0.008032367 |
| BLOC1S6 | 0.326843 | 10.536287 | 0.0003914 | 0.008032367 |
| POLR1C | -0.15011 | 7.6772334 | 0.0003935 | 0.00806708 |
| RXRA | 0.246766 | 10.340764 | 0.0003942 | 0.008072577 |
| TPI1P2 | -0.19018 | 7.8675586 | 0.0003965 | 0.008111585 |
| PTGES2 | -0.13335 | 7.471293 | 0.0003971 | 0.008116444 |
| MFSD14C | 0.354568 | 11.613968 | 0.0004024 | 0.008216158 |
| RP9 | -0.12709 | 7.4079207 | 0.0004032 | 0.008225333 |
| CXCR6 | -0.18157 | 6.95422 | 0.0004044 | 0.008233725 |
| 4GALNT | -0.07759 | 6.7589769 | 0.0004044 | 0.008233725 |
| MRPL34 | -0.16773 | 8.0253196 | 0.0004074 | 0.008285624 |
| TP53BP1 | 0.097588 | 7.1393268 | 0.0004091 | 0.008314018 |
| PARVG | 0.220066 | 8.9336629 | 0.0004103 | 0.008328401 |
| PQLC3 | -0.20402 | 9.1462585 | 0.0004139 | 0.008393312 |
| WBP2 | 0.274479 | 12.49997 | 0.00042 | 0.008509811 |
| RHGAP1 | -0.22394 | 8.770922 | 0.0004218 | 0.008538922 |
| ADK | -0.18814 | 7.5812067 | 0.0004241 | 0.008574684 |
| PDE5A | 0.358214 | 7.9539542 | 0.0004244 | 0.008574684 |
| VPS25 | -0.11614 | 7.5027576 | 0.0004289 | 0.008647949 |
| VAPA | 0.122457 | 7.0540737 | 0.0004289 | 0.008647949 |

| IDNK | -0.10401 | 7.4857551 | 0.0004314 | 0.008689969 |
| --- | --- | --- | --- | --- |
| COPS6 | -0.08928 | 7.4096636 | 0.0004361 | 0.008777038 |
| NLRC4 | 0.164471 | 7.3369892 | 0.0004385 | 0.008815972 |
| PLEKHO1 | -0.22254 | 9.3267861 | 0.0004424 | 0.008886206 |
| OSBPL5 | -0.19945 | 7.4801752 | 0.0004433 | 0.008890046 |
| SELPLG | 0.201268 | 7.5147208 | 0.0004435 | 0.008890046 |
| ANAPC11 | -0.13511 | 7.9559567 | 0.0004439 | 0.008890046 |
| SEMA4C | -0.11825 | 7.0880897 | 0.0004449 | 0.008903076 |
| SNHG32 | -0.31965 | 10.464435 | 0.0004474 | 0.008943791 |
| ARHGEF1 | 0.171654 | 7.1941777 | 0.0004489 | 0.008965 |
| COPS7A | -0.13654 | 8.5583574 | 0.0004508 | 0.008995033 |
| CCT6P3 | -0.16889 | 9.1309548 | 0.0004514 | 0.008998189 |
| MAP3K5 | 0.178576 | 9.4165468 | 0.0004524 | 0.009008534 |
| DIABLO | -0.13588 | 9.0534789 | 0.0004566 | 0.009082109 |
| ARFGEF1 | 0.132733 | 8.5731792 | 0.0004573 | 0.009082109 |
| MAGOH | -0.15836 | 7.6893596 | 0.0004574 | 0.009082109 |
| SNX13 | 0.107563 | 7.427091 | 0.0004578 | 0.009082986 |
| GNAI3 | 0.188164 | 8.2904289 | 0.0004586 | 0.009090231 |
| CBWD2 | -0.21068 | 8.1482292 | 0.0004606 | 0.00911243 |
| GTF2H5 | -0.15395 | 8.6225112 | 0.0004606 | 0.00911243 |
| MYL12B | -0.13273 | 11.513329 | 0.0004614 | 0.009116159 |
| SELENOS | -0.23149 | 8.8667424 | 0.0004617 | 0.009116159 |
| GM2A | 0.166351 | 7.0852901 | 0.000464 | 0.009152401 |
| ACSL4 | 0.246363 | 9.1877283 | 0.0004652 | 0.009159816 |
| COG2 | -0.12657 | 7.7009822 | 0.0004652 | 0.009159816 |
| HERC4 | 0.101065 | 7.963985 | 0.0004662 | 0.00916832 |
| RPL13AP3 | -0.23087 | 12.994973 | 0.0004665 | 0.00916832 |
| GLT1D1 | 0.14826 | 7.197431 | 0.0004683 | 0.00919122 |
| PAGR1 | -0.15873 | 8.408751 | 0.0004686 | 0.00919122 |
| PIK3CD | 0.205365 | 9.216935 | 0.000469 | 0.009191935 |
| DHX9 | 0.10271 | 7.4143702 | 0.0004696 | 0.009193791 |
| SDR39U1 | -0.09887 | 6.9506916 | 0.0004715 | 0.009222119 |
| ADRB2 | -0.26528 | 8.6574711 | 0.0004728 | 0.009234838 |
| COPZ1 | -0.15408 | 8.0893092 | 0.0004732 | 0.009234838 |
| MXD4 | -0.1976 | 9.316839 | 0.0004737 | 0.009234838 |
| GTF2A2 | -0.18127 | 9.2464314 | 0.0004739 | 0.009234838 |
| JHY | 0.37279 | 8.5183984 | 0.0004748 | 0.009243997 |
| TRIB1 | 0.273453 | 8.6826573 | 0.0004821 | 0.00937433 |
| ZMYM2 | 0.095692 | 7.7064652 | 0.0004824 | 0.00937433 |
| HLA-DMA | -0.29836 | 9.8358527 | 0.0004828 | 0.009374598 |
| SEC61G | -0.23513 | 10.34771 | 0.0004841 | 0.009389575 |
| HDDC2 | -0.25218 | 9.3181028 | 0.0004853 | 0.009405518 |
| NHP2 | -0.1884 | 7.9146184 | 0.0004863 | 0.009416102 |
| PRDM4 | -0.15781 | 8.2739291 | 0.000488 | 0.009437652 |
| MRPS22 | -0.14194 | 8.4286751 | 0.0004885 | 0.009437652 |
| MRPS7 | -0.14149 | 7.6934434 | 0.0004888 | 0.009437652 |
| C16orf72 | 0.18974 | 8.4297761 | 0.0004911 | 0.009474143 |
| LINC0033 | 0.073905 | 6.6774086 | 0.0004927 | 0.009490993 |
| EMP3 | -0.19012 | 11.100985 | 0.0004934 | 0.009490993 |
| GNB2 | 0.187374 | 7.6840856 | 0.000494 | 0.009490993 |
| HADH | -0.16191 | 8.0632949 | 0.0004942 | 0.009490993 |
| ICE1 | 0.136343 | 7.5556349 | 0.0004943 | 0.009490993 |

| NUBP1 | -0.12082 | 7.3106379 | 0.0004947 | 0.009490993 |
| --- | --- | --- | --- | --- |
| RASGRP4 | 0.120493 | 7.0624617 | 0.0004964 | 0.009514055 |
| COLQ | -0.20905 | 7.3216634 | 0.0004974 | 0.009524029 |
| PTK2B | 0.225596 | 8.1064969 | 0.0004985 | 0.009537748 |
| NCOR1 | 0.095133 | 7.0934476 | 0.0004991 | 0.009540224 |
| RNPEP | -0.23195 | 8.1181242 | 0.000501 | 0.00956654 |
| CHMP1A | 0.173694 | 8.3961012 | 0.0005027 | 0.009591248 |
| PKD2 | 0.108861 | 7.0102483 | 0.0005055 | 0.00962997 |
| TBCC | -0.09746 | 7.6145856 | 0.0005057 | 0.00962997 |
| CCDC18 | -0.06763 | 6.7943034 | 0.0005072 | 0.009632785 |
| ARL16 | 0.333695 | 11.882574 | 0.0005072 | 0.009632785 |
| SLC12A6 | 0.188054 | 7.5578935 | 0.0005073 | 0.009632785 |
| PGRMC2 | -0.15284 | 8.4903955 | 0.0005076 | 0.009632785 |
| DCTN4 | 0.138969 | 7.7632962 | 0.0005084 | 0.009638902 |
| DDX50 | -0.1399 | 7.6454973 | 0.0005104 | 0.009667039 |
| TAGLN2 | 0.29568 | 11.608906 | 0.0005121 | 0.009691033 |
| PPM1B | 0.107133 | 9.702747 | 0.0005127 | 0.00969275 |
| BCYRN1 | 0.318847 | 9.4785036 | 0.000514 | 0.009707474 |
| ACAT2 | -0.14342 | 7.7812109 | 0.0005144 | 0.009707474 |
| DEDD | 0.135594 | 8.0812772 | 0.0005161 | 0.009731141 |
| WASF2 | 0.352484 | 8.9192009 | 0.0005183 | 0.009764126 |
| PMPCB | -0.15928 | 8.8865284 | 0.0005192 | 0.009771781 |
| GPSM3 | 0.204029 | 10.910192 | 0.0005265 | 0.009899707 |
| USP32 | 0.197183 | 7.3666611 | 0.0005269 | 0.009899707 |
| TRAFD1 | 0.187401 | 7.8784034 | 0.0005285 | 0.009921504 |

MSL2 0.132205 7.6634491 0.0005299 0.009937987
