# Supplementary material for: Identification of diagnostic mRNA biomarkers in whole blood for ankylosing spondylitis using WGCNA and machine learning feature selection
Source: Front Immunol. 2022 Sep 12;13:956027. doi: 10.3389/fimmu.2022.956027 (PMC9510835; doi:10.3389/fimmu.2022.956027)
Supplement: Supplementary file 5 [file Table_4.docx]

Supplementary Table 4. intersected mRNAs between modules and differential expressed mRNAs

| mRNA | logFC | AveExpr | module |
| --- | --- | --- | --- |
| ZSWIM6 | 0.228282709 | 8.147582781 | blue |
| ZNF281 | 0.322697117 | 9.194699729 | blue |
| ZDHHC18 | 0.395575637 | 9.11844034 | blue |
| ZBTB34 | 0.227796738 | 8.375381161 | blue |
| XKR8 | 0.259635082 | 8.042697106 | blue |
| VPS8 | 0.164342427 | 8.043894253 | blue |
| USP6 | 0.17544173 | 7.210355161 | blue |
| USP15 | 0.234795658 | 8.206442332 | blue |
| UNC13D | 0.160678676 | 7.436160856 | blue |
| UBXN2B | 0.255577122 | 8.079230722 | blue |
| TUT7 | 0.230298041 | 7.808321193 | blue |
| TRIB1 | 0.273453248 | 8.682657303 | blue |
| TNFAIP2 | 0.225309199 | 7.538086156 | blue |
| TMEM126B | -0.265156163 | 9.060250951 | blue |
| TLE3 | 0.176477393 | 7.22544779 | blue |
| TBC1D14 | 0.267580204 | 8.938741569 | blue |
| SYNJ1 | 0.189851577 | 8.472253403 | blue |
| SVIL | 0.342298077 | 9.478166028 | blue |
| SUSD6 | 0.263987452 | 8.838277979 | blue |
| STX6 | 0.170430116 | 8.257284322 | blue |
| STK40 | 0.270188588 | 10.53040453 | blue |
| STAT3 | 0.284473131 | 9.470778292 | blue |
| ST8SIA4 | 0.250785669 | 8.449949468 | blue |
| SRPK1 | 0.310091638 | 8.699071347 | blue |
| SPAG9 | 0.233506503 | 8.28696864 | blue |
| SORL1 | 0.604582312 | 11.20673668 | blue |
| SLC19A1 | 0.220072258 | 7.582427815 | blue |
| SH3BP5L | 0.162896687 | 7.564079347 | blue |
| SERINC1 | 0.24888019 | 9.289921479 | blue |
| SBNO2 | 0.327689978 | 7.709380014 | blue |
| RXRA | 0.2467664 | 10.34076353 | blue |
| RNF24 | 0.406820615 | 9.197051639 | blue |
| RNF19B | 0.243888401 | 7.735074993 | blue |
| RGS2 | 0.367653479 | 13.37337731 | blue |
| RFX1 | 0.20732731 | 8.124179165 | blue |
| RESF1 | 0.382545302 | 11.33090837 | blue |
| RASSF2 | 0.364019619 | 11.30518383 | blue |
| RAB5C | 0.339431766 | 8.573845504 | blue |
| RAB33B | 0.197616437 | 8.806370632 | blue |
| PYGL | 0.415620102 | 10.54147042 | blue |
| PXN | 0.208487969 | 7.956836644 | blue |
| PREX1 | 0.276638345 | 7.926572375 | blue |
| PPP4R1 | 0.337822023 | 9.725613722 | blue |
| PPP1R12A | 0.215288708 | 8.349748629 | blue |
| PPFIA1 | 0.177664594 | 8.419953243 | blue |
| PLPPR2 | 0.247134423 | 7.659769807 | blue |
| PKN2 | 0.337404644 | 8.151079692 | blue |
| PISD | 0.2947307 | 8.699030236 | blue |
| PIK3CD | 0.205365481 | 9.216935014 | blue |
| PICALM | 0.380154112 | 10.73525522 | blue |

| PHF21A | 0.263753796 | 9.912839153 | blue |
| --- | --- | --- | --- |
| PHF20L1 | 0.19517129 | 8.506384993 | blue |
| PHF2 | 0.194774622 | 7.881737806 | blue |
| PDZD8 | 0.163038787 | 7.389800515 | blue |
| PDPK1 | 0.227189183 | 8.627842451 | blue |
| NPRL2 | -0.165735937 | 8.803185424 | blue |
| NLRC4 | 0.164471343 | 7.336989224 | blue |
| NIN | 0.267081229 | 9.558111049 | blue |
| NADK | 0.388144842 | 8.701755865 | blue |
| MYO9B | 0.244299012 | 8.582706375 | blue |
| MYO1F | 0.309271467 | 8.517007596 | blue |
| MYD88 | 0.212283983 | 8.85498766 | blue |
| MXD1 | 0.421484046 | 11.04318219 | blue |
| MTF1 | 0.304084379 | 8.045503211 | blue |
| MSL1 | 0.322790457 | 7.754564946 | blue |
| MEFV | 0.347932023 | 7.706260324 | blue |
| MED13L | 0.287715445 | 7.768613493 | blue |
| MCL1 | 0.206074775 | 10.96824238 | blue |
| MAP3K5 | 0.178576113 | 9.416546813 | blue |
| MAP3K2 | 0.279346821 | 8.508299767 | blue |
| MAP3K11 | 0.213098481 | 8.094463608 | blue |
| MAP2K4 | 0.269962058 | 8.239461572 | blue |
| LYST | 0.353982598 | 9.168581049 | blue |
| LRMP | 0.189252867 | 9.423116076 | blue |
| LAT2 | 0.305884392 | 9.592240681 | blue |
| KDM6B | 0.167234852 | 7.246583751 | blue |
| KCNJ2 | 0.369371717 | 8.587472332 | blue |
| ITGAM | 0.391384771 | 10.03486423 | blue |
| IQSEC1 | 0.259433725 | 8.352907436 | blue |
| IQGAP1 | 0.324073663 | 10.16747333 | blue |
| INPP5A | 0.199016096 | 7.659552672 | blue |
| IL17RA | 0.317652694 | 8.725842485 | blue |
| GPSM3 | 0.204028894 | 10.91019183 | blue |
| GNB2 | 0.187374258 | 7.684085568 | blue |
| GMIP | 0.191612326 | 7.971934325 | blue |
| GLT1D1 | 0.14825973 | 7.197431007 | blue |
| FAM160B1 | 0.209561225 | 9.060505049 | blue |
| DYSF | 0.638175306 | 9.555248313 | blue |
| DPP7 | -0.250647985 | 8.417813256 | blue |
| DIP2B | 0.267650631 | 9.399088958 | blue |
| DENND5A | 0.322696685 | 9.625880667 | blue |
| DCP2 | 0.169357508 | 10.02981118 | blue |
| CUX1 | 0.308643778 | 7.983724315 | blue |
| CREBRF | 0.347376056 | 9.652754757 | blue |
| CREB5 | 0.56316426 | 9.863533243 | blue |
| CPEB2 | 0.167216155 | 7.340703253 | blue |
| CFLAR | 0.359709633 | 11.05303872 | blue |
| CDC42SE1 | 0.403972267 | 9.627034924 | blue |
| CBL | 0.344939273 | 9.039692708 | blue |
| CAPZA2 | 0.2290683 | 10.04879142 | blue |
| CANT1 | 0.297158302 | 8.434303729 | blue |
| C5AR1 | 0.46809996 | 10.16025099 | blue |

| C16orf58 | -0.25480161 | 8.461523715 | blue |
| --- | --- | --- | --- |
| BRI3 | 0.288745137 | 10.09077998 | blue |
| BAZ2B | 0.382681662 | 9.04675025 | blue |
| BASP1 | 0.4957846 | 12.31410506 | blue |
| ARID3A | 0.289015246 | 8.614908583 | blue |
| ARAP1 | 0.262153882 | 8.234587679 | blue |
| APBB1IP | 0.3530698 | 10.19132793 | blue |
| ANTXR2 | 0.288244762 | 9.484307347 | blue |
| ANPEP | 0.458354272 | 8.408700521 | blue |
| ANKRD13A | 0.199422579 | 10.20735837 | blue |
| ALPK1 | 0.319584483 | 8.046009971 | blue |
| AKIRIN2 | 0.239530521 | 9.40490084 | blue |
| ADAM8 | 0.306034623 | 9.345433556 | blue |
| ACSL4 | 0.246362642 | 9.18772825 | blue |
| ZNF689 | -0.173404957 | 8.045681082 | grey |
| YTHDC1 | 0.207420058 | 10.07884913 | grey |
| VPS16 | -0.225481088 | 8.407565547 | grey |
| VPS13B | 0.253023641 | 7.258136551 | grey |
| UBAC2 | -0.254373119 | 8.458423556 | grey |
| TSSC4 | -0.17276476 | 8.451870826 | grey |
| TSC22D4 | 0.275362751 | 7.726850968 | grey |
| TNFAIP8 | -0.333495488 | 8.553970272 | grey |
| THEM6 | -0.242566871 | 7.498687865 | grey |
| TCF25 | -0.220742319 | 9.374466167 | grey |
| TCEA1 | -0.25543098 | 8.11036681 | grey |
| TAPBP | 0.266793708 | 9.767333319 | grey |
| SYNGR2 | -0.232398615 | 10.99974443 | grey |
| STAT5A | 0.326006352 | 9.125517257 | grey |
| SRSF10 | -0.185368346 | 7.978644586 | grey |
| SRRT | -0.162070808 | 9.728973986 | grey |
| SQSTM1 | -0.277001588 | 11.13598197 | grey |
| SON | 0.176157238 | 10.51561197 | grey |
| SEC16A | 0.21211414 | 8.815072146 | grey |
| SAFB | -0.227401792 | 8.534936922 | grey |
| RXRB | -0.186000517 | 8.417352568 | grey |
| RPS6KB2 | -0.232226744 | 9.46205816 | grey |
| RNF220 | -0.155088646 | 7.993577633 | grey |
| RNF126 | -0.212326923 | 7.967204028 | grey |
| REPIN1 | -0.254726679 | 8.736451007 | grey |
| RAB8B | 0.192525862 | 10.26652531 | grey |
| PSMC6 | -0.189351196 | 8.997379083 | grey |
| PRRC2A | 0.177013679 | 7.498004978 | grey |
| PRDX3 | -0.291340575 | 9.080247438 | grey |
| PRDM4 | -0.157806975 | 8.273929146 | grey |
| PPM1A | 0.257658405 | 8.371976681 | grey |
| PPID | -0.213761849 | 7.111927238 | grey |
| POTEF | 0.230779912 | 13.12510006 | grey |
| POLR2A | 0.214951648 | 9.826477882 | grey |
| PKM | 0.275852012 | 8.720203153 | grey |
| PEA15 | -0.246672777 | 8.647949514 | grey |
| PACS1 | 0.215874623 | 7.5848072 | grey |
| NBPF9 | 0.442528077 | 11.63922586 | grey |

| NBPF3 | 0.19476465 | 8.536582749 | grey |
| --- | --- | --- | --- |
| NBPF10 | 0.425538225 | 11.68057856 | grey |
| MYCBP2 | 0.235529196 | 8.648839694 | grey |
| MSN | 0.296557 | 11.07840831 | grey |
| MRPL20-AS1 | -0.227521473 | 8.033032336 | grey |
| KLRB1 | -0.581509502 | 10.81666897 | grey |
| KAT6A | 0.3607183 | 9.763879097 | grey |
| HPS6 | -0.333610681 | 8.749406778 | grey |
| HLA-DMA | -0.298362646 | 9.835852667 | grey |
| H2AFY | -0.214313754 | 11.75006782 | grey |
| GSDMA | -0.240835544 | 7.033671933 | grey |
| GNAI2 | 0.217790573 | 10.48596117 | grey |
| GLYR1 | 0.165884605 | 8.133139558 | grey |
| GATAD2B | 0.208822059 | 7.694573442 | grey |
| FKBP1A | 0.416861808 | 9.456870986 | grey |
| FBXL3 | 0.167439447 | 8.196194235 | grey |
| EIF4E | -0.189491538 | 7.87842549 | grey |
| E4F1 | -0.21304575 | 8.51177635 | grey |
| DOCK8 | 0.31531104 | 10.19989422 | grey |
| CXCR6 | -0.181568472 | 6.954220019 | grey |
| CEP350 | 0.312323073 | 8.822896389 | grey |
| CDC37 | -0.263640527 | 11.0413959 | grey |
| CARD11 | -0.304908362 | 8.730592306 | grey |
| CALM3 | -0.267240346 | 11.12339821 | grey |
| C12orf10 | -0.209393835 | 8.714490694 | grey |
| BRD2 | -0.174505869 | 10.18641083 | grey |
| BRAT1 | -0.266985088 | 8.369071836 | grey |
| BICRAL | 0.172806629 | 8.876860688 | grey |
| ATP6AP1 | -0.184738208 | 10.50900161 | grey |
| ARHGAP17 | -0.223943048 | 8.770921979 | grey |
| ARAF | -0.203950021 | 8.819137563 | grey |
| ACTB | 0.226484231 | 13.81676569 | grey |
| ZNF876P | 0.282559663 | 13.57656774 | yellow |
| ZNF69 | 0.485129488 | 11.6625311 | yellow |
| ZNF669 | 0.386084838 | 8.49229065 | yellow |
| ZNF577 | 0.3990937 | 8.946203139 | yellow |
| ZNF557 | 0.271621453 | 8.581506135 | yellow |
| ZNF549 | 0.478281542 | 12.92097271 | yellow |
| ZNF493 | 0.251657558 | 7.594790296 | yellow |
| ZNF483 | 0.611317588 | 10.69424635 | yellow |
| ZNF394 | 0.481966142 | 11.68817683 | yellow |
| ZMAT3 | 0.393139552 | 12.21946852 | yellow |
| ZFC3H1 | 0.227335529 | 7.923744146 | yellow |
| YRDC | 0.502117523 | 11.84685908 | yellow |
| XRCC2 | 0.552966842 | 11.06479467 | yellow |
| XPNPEP3 | 0.360156606 | 14.22528948 | yellow |
| VKORC1 | -0.194430644 | 9.172571618 | yellow |
| USP32 | 0.197183437 | 7.36666105 | yellow |
| UBE2G2 | 0.370368993 | 8.415583772 | yellow |
| TRIM38 | 0.247245057 | 8.05422741 | yellow |
| TNFSF15 | 0.307994769 | 8.233455176 | yellow |
| TMEM17 | 0.512435265 | 12.41567836 | yellow |

| TMEM160 | -0.21679399 | 8.202806538 | yellow |
| --- | --- | --- | --- |
| TDRD1 | 0.318957971 | 8.364934244 | yellow |
| TDP1 | 0.443041204 | 10.5999839 | yellow |
| TCAF1 | 0.290476629 | 14.06071908 | yellow |
| SULT1A1 | 0.311457392 | 11.97651475 | yellow |
| SLC25A3 | -0.244058919 | 10.71219288 | yellow |
| SHROOM4 | 0.456594681 | 10.25051413 | yellow |
| SEMA3E | 0.502369956 | 10.3212934 | yellow |
| SDHAF2 | -0.339570108 | 8.902916597 | yellow |
| SCARNA3 | 0.274968258 | 7.504939861 | yellow |
| RPS25 | -0.207228756 | 14.12811931 | yellow |
| RPS11 | -0.206875867 | 13.1840217 | yellow |
| RPL35A | -0.208884044 | 12.9738987 | yellow |
| RPL30 | -0.231437715 | 13.27370189 | yellow |
| RPL11 | -0.20129286 | 13.95755105 | yellow |
| RNY3 | 0.229926731 | 8.137170194 | yellow |
| RAMP2-AS1 | 0.328048687 | 13.86719945 | yellow |
| QRFPR | 0.38298944 | 10.29486853 | yellow |
| PSMB7 | -0.247765025 | 8.278254693 | yellow |
| PPM1K | 0.291522725 | 8.37665584 | yellow |
| POFUT1 | 0.362595798 | 12.94445287 | yellow |
| PLPP5 | 0.459048425 | 13.22284092 | yellow |
| PALM2 | 0.431216833 | 10.79390498 | yellow |
| ORC4 | 0.257511895 | 7.848429018 | yellow |
| OGA | 0.19474596 | 10.83210278 | yellow |
| OCIAD1 | 0.364519148 | 12.3941586 | yellow |
| NPIPB13 | 0.308724892 | 10.90468353 | yellow |
| NMT2 | 0.413831335 | 11.36772336 | yellow |
| NBEAL2 | 0.390066867 | 9.19452366 | yellow |
| NACA4P | -0.306151221 | 8.446071185 | yellow |
| N4BP2L2 | 0.29808889 | 8.551292665 | yellow |
| MYO3B | 0.29419884 | 8.044190025 | yellow |
| MINDY1 | 0.283593475 | 9.58696866 | yellow |
| MICAL1 | 0.272229807 | 8.376855946 | yellow |
| MFSD14C | 0.354567723 | 11.61396764 | yellow |
| MFSD11 | 0.363162029 | 9.288465188 | yellow |
| METTL21A | 0.417876146 | 12.41683026 | yellow |
| MBOAT2 | 0.247892343 | 7.647963004 | yellow |
| MBD4 | 0.400329563 | 10.63260601 | yellow |
| MAPK8IP3 | 0.28252266 | 8.435898608 | yellow |
| MAGT1 | 0.526642692 | 11.59954106 | yellow |
| MAFF | 0.284580993 | 7.523631847 | yellow |
| LRRFIP1 | 0.354665444 | 11.01173034 | yellow |
| LRRC37BP1 | 0.377184413 | 11.97022997 | yellow |
| LOC100190986 | 0.45464019 | 8.290589924 | yellow |
| LOC100128288 | 0.429338315 | 11.35991885 | yellow |
| LMOD3 | 0.47219641 | 10.13655609 | yellow |
| LINC02591 | 0.359964075 | 12.5410946 | yellow |
| LINC00667 | 0.366629702 | 9.961200493 | yellow |
| LCOR | 0.255712369 | 8.921995306 | yellow |
| JHY | 0.372789858 | 8.518398435 | yellow |
| ITPK1-AS1 | 0.360995392 | 12.75481207 | yellow |

| INTS3 | 0.247728573 | 7.97543009 | yellow |
| --- | --- | --- | --- |
| HSPA4 | -0.184799179 | 8.506592576 | yellow |
| HSD17B7 | 0.399741831 | 11.92733033 | yellow |
| HNRNPU | 0.545392315 | 10.491551 | yellow |
| GTF2E2 | -0.19224071 | 9.056681188 | yellow |
| GRIPAP1 | 0.462412702 | 11.10181623 | yellow |
| GNPDA1 | -0.22489701 | 8.334359057 | yellow |
| GLO1 | -0.217073019 | 8.543921667 | yellow |
| GDPD1 | 0.357228802 | 8.197477214 | yellow |
| GCLM | 0.303231837 | 7.647713351 | yellow |
| FUT6 | 0.183489164 | 7.407940782 | yellow |
| FOXP1-IT1 | 0.204224872 | 7.572843146 | yellow |
| FOXK1 | 0.385306275 | 12.77371435 | yellow |
| FKBP5 | 0.437133394 | 11.27843795 | yellow |
| FKBP14 | 0.576014725 | 12.07467528 | yellow |
| FIBP | -0.1570281 | 8.114436307 | yellow |
| FCAR | 0.438046788 | 9.680690625 | yellow |
| FAM126B | 0.300392723 | 12.89415763 | yellow |
| EXO5 | 0.477995575 | 11.23341645 | yellow |
| ERAP2 | 0.42135856 | 12.56815434 | yellow |
| EID2B | 0.404639381 | 12.34786053 | yellow |
| DUSP19 | 0.335231017 | 11.73566384 | yellow |
| DTWD2 | 0.407552462 | 11.30195144 | yellow |
| DMC1 | 0.382832106 | 12.78492548 | yellow |
| CLK4 | 0.181442743 | 7.688675521 | yellow |
| CHRNA5 | 0.373862096 | 9.464698014 | yellow |
| CFAP74 | 0.4179877 | 11.31581931 | yellow |
| CEP19 | 0.419856004 | 11.72477824 | yellow |
| BMS1P1 | 0.365840312 | 12.1405294 | yellow |
| BLZF1 | 0.375207604 | 12.31023544 | yellow |
| BLOC1S6 | 0.326842935 | 10.53628731 | yellow |
| BIRC3 | 0.367091533 | 10.68778794 | yellow |
| BCYRN1 | 0.318847175 | 9.47850359 | yellow |
| ATP5MG | -0.268320508 | 11.02641492 | yellow |
| ATG10 | 0.23487045 | 8.589088833 | yellow |
| ARL16 | 0.333695087 | 11.88257401 | yellow |
| AKAP5 | 0.248008994 | 13.70373913 | yellow |
| ABRAXAS1 | 0.393693912 | 11.37928004 | yellow |
