# Supplementary material for: Identification of diagnostic mRNA biomarkers in whole blood for ankylosing spondylitis using WGCNA and machine learning feature selection
Source: Front Immunol. 2022 Sep 12;13:956027. doi: 10.3389/fimmu.2022.956027 (PMC9510835; doi:10.3389/fimmu.2022.956027)
Supplement: Supplementary file 6 [file Table_5.docx]

Supplementary Table 5. Nodes’ scores of selected hub mRNAs.

| Node mRNAs | MCC | DMNC | MNC | Degree | EPC | BottleNeck | EcCentricity | Closeness | Radiality | Betweenness | Stress | ClusteringCoefficient | Presenting NO. |
| --- | --- | --- | --- | --- | --- | --- | --- | --- | --- | --- | --- | --- | --- |
| CSL4 | 1 | 0 | 1 | 1 | 40.391 | 1 | 0.13298 | 59.97857 | 8.74375 | 0 | 0 | 0 | 12 |
| PEA15 | 2 | 0 | 1 | 2 | 57.817 | 1 | 0.11636 | 55.77976 | 8.44735 | 35.46252 | 236 | 0 | 12 |
| EXO5 | 1 | 0 | 1 | 1 | 8.261 | 1 | 0.09309 | 35.02738 | 6.46055 | 0 | 0 | 0 | 11 |
| CDC42SE1 | 1 | 0 | 1 | 1 | 35.826 | 1 | 0.11636 | 52.475 | 8.27599 | 0 | 0 | 0 | 11 |
| NBPF3 | 1 | 0 | 1 | 1 | 14.41 | 1 | 0.10343 | 41.10675 | 7.22934 | 0 | 0 | 0 | 11 |
| TBC1D14 | 1 | 0 | 1 | 1 | 22.397 | 1 | 0.11636 | 44.54048 | 7.62299 | 0 | 0 | 0 | 11 |
| H2AFY | 4 | 0 | 1 | 4 | 60.725 | 4 | 0.13298 | 64.16905 | 8.87342 | 748.0556 | 2698 | 0 | 11 |
| ALPK1 | 1 | 0 | 1 | 1 | 37.578 | 1 | 0.11636 | 47.80952 | 7.90086 | 0 | 0 | 0 | 11 |
| TLE3 | 1 | 0 | 1 | 1 | 30.019 | 1 | 0.11636 | 49.74167 | 8.1139 | 0 | 0 | 0 | 11 |
| TNFAIP8 | 2 | 0 | 1 | 2 | 35.48 | 2 | 0.11636 | 51.07619 | 8.11853 | 400 | 1292 | 0 | 11 |
| SLC19A1 | 1 | 0 | 1 | 1 | 1.347 | 1 | 0.00922 | 1 | 0.02765 | 0 | 0 | 0 | 10 |
| MFSD11 | 1 | 0 | 1 | 1 | 1.349 | 1 | 0.00922 | 1 | 0.02765 | 0 | 0 | 0 | 10 |
| FUT6 | 1 | 0 | 1 | 1 | 1.364 | 1 | 0.00922 | 1 | 0.02765 | 0 | 0 | 0 | 10 |
| GRIPAP1 | 2 | 0 | 1 | 2 | 10.74 | 2 | 0.09309 | 36.97976 | 6.62265 | 400 | 2456 | 0 | 10 |
| VPS13B | 1 | 0 | 1 | 1 | 17.028 | 1 | 0.09309 | 36.83016 | 6.60875 | 0 | 0 | 0 | 9 |
| ZNF689 | 1 | 0 | 1 | 1 | 36.911 | 1 | 0.10343 | 51.3504 | 8.16948 | 0 | 0 | 0 | 9 |
| DIP2B | 1 | 0 | 1 | 1 | 1.363 | 1 | 0.00922 | 1 | 0.02765 | 0 | 0 | 0 | 9 |
| TDRD1 | 2 | 0 | 1 | 2 | 38.309 | 2 | 0.11636 | 52.1131 | 8.14632 | 194.5509 | 388 | 0 | 9 |
| PRDX3 | 2 | 0 | 1 | 2 | 59.497 | 1 | 0.13298 | 62.71905 | 8.85026 | 26.60101 | 250 | 0 | 9 |
| SEMA3E | 1 | 0 | 1 | 1 | 1.371 | 1 | 0.00922 | 1 | 0.02765 | 0 | 0 | 0 | 9 |
| MICAL1 | 1 | 0 | 1 | 1 | 1.371 | 1 | 0.00922 | 1 | 0.02765 | 0 | 0 | 0 | 9 |
| TSSC4 | 1 | 0 | 1 | 1 | 22.237 | 1 | 0.11636 | 47.83571 | 7.94718 | 0 | 0 | 0 | 9 |
| NBPF10 | 1 | 0 | 1 | 1 | 15.08 | 1 | 0.11636 | 45.89643 | 7.79435 | 0 | 0 | 0 | 9 |
| NADK | 1 | 0 | 1 | 1 | 6.679 | 1 | 0.08463 | 34.61591 | 6.29846 | 0 | 0 | 0 | 9 |
| SH3BP5L | 1 | 0 | 1 | 1 | 19.744 | 1 | 0.10343 | 41.64722 | 7.3127 | 0 | 0 | 0 | 9 |
| ST8SIA4 | 1 | 0 | 1 | 1 | 1.364 | 1 | 0.00922 | 1 | 0.02765 | 0 | 0 | 0 | 9 |
| SON | 1 | 0 | 1 | 1 | 16.413 | 1 | 0.08463 | 36.6048 | 6.57633 | 0 | 0 | 0 | 9 |
| FKBP14 | 1 | 0 | 1 | 1 | 35.787 | 1 | 0.11636 | 50.375 | 8.15095 | 0 | 0 | 0 | 9 |
| OCIAD1 | 1 | 0 | 1 | 1 | 39.684 | 1 | 0.13298 | 59.97857 | 8.74375 | 0 | 0 | 0 | 9 |
| CPEB2 | 1 | 0 | 1 | 1 | 5.233 | 1 | 0.08463 | 31.004 | 5.6964 | 0 | 0 | 0 | 9 |
| ZBTB34 | 1 | 0 | 1 | 1 | 22.195 | 1 | 0.10343 | 43.02103 | 7.42848 | 0 | 0 | 0 | 9 |
| SLC25A3 | 3 | 0 | 1 | 3 | 37.18 | 2 | 0.13298 | 52.23095 | 8.22042 | 198.8027 | 664 | 0 | 9 |
| GLO1 | 2 | 0 | 1 | 2 | 25.337 | 1 | 0.10343 | 46.6504 | 7.73414 | 192.0626 | 360 | 0 | 9 |
| TMEM126B | 2 | 0 | 1 | 2 | 39.616 | 2 | 0.13298 | 53.7381 | 8.34083 | 363.3709 | 1252 | 0 | 9 |
| FAM160B1 | 1 | 0 | 1 | 1 | 4.848 | 1 | 0.07757 | 31.07035 | 5.66398 | 0 | 0 | 0 | 8 |
| ZNF493 | 1 | 0 | 1 | 1 | 1.347 | 1 | 0.00922 | 1 | 0.02765 | 0 | 0 | 0 | 8 |
| BASP1 | 1 | 0 | 1 | 1 | 33.542 | 1 | 0.13298 | 52.06429 | 8.25747 | 0 | 0 | 0 | 8 |
| BAZ2B | 2 | 0 | 1 | 2 | 12.038 | 2 | 0.10343 | 37.40198 | 6.72453 | 400 | 1146 | 0 | 8 |
| TNFAIP2 | 1 | 0 | 1 | 1 | 13.913 | 1 | 0.10343 | 40.34365 | 7.19229 | 0 | 0 | 0 | 8 |
| TDP1 | 2 | 0 | 1 | 2 | 18.365 | 2 | 0.10343 | 42.80159 | 7.3868 | 400 | 1148 | 0 | 8 |
| MYO9B | 3 | 0.30779 | 2 | 3 | 67.821 | 2 | 0.13298 | 63.21905 | 8.85026 | 132.7662 | 416 | 0.33333 | 8 |
| TNFSF15 | 1 | 0 | 1 | 1 | 20.303 | 1 | 0.10343 | 43.50159 | 7.51184 | 0 | 0 | 0 | 7 |
| PYGL | 4 | 0 | 1 | 4 | 84.237 | 1 | 0.13298 | 64.93571 | 8.91047 | 218.5462 | 1166 | 0 | 7 |
| NPRL2 | 2 | 0 | 1 | 2 | 47.422 | 3 | 0.11636 | 53.7869 | 8.31767 | 151.4644 | 562 | 0 | 7 |
| MXD1 | 1 | 0 | 1 | 1 | 22.834 | 1 | 0.10343 | 42.94484 | 7.42385 | 0 | 0 | 0 | 7 |
| FAM175A | 2 | 0 | 1 | 2 | 37.466 | 3 | 0.13298 | 60.57857 | 8.72985 | 796 | 1936 | 0 | 7 |
| POFUT1 | 2 | 0 | 1 | 2 | 29.535 | 3 | 0.09309 | 45.30119 | 7.50721 | 796 | 3788 | 0 | 7 |
| PALM2 | 1 | 0 | 1 | 1 | 14.789 | 1 | 0.10343 | 43.26587 | 7.51647 | 0 | 0 | 0 | 6 |
| ZDHHC18 | 1 | 0 | 1 | 1 | 1.363 | 1 | 0.00922 | 1 | 0.02765 | 0 | 0 | 0 | 6 |
| GLYR1 | 2 | 0 | 1 | 2 | 40.381 | 2 | 0.11636 | 53.09048 | 8.22042 | 411.087 | 1028 | 0 | 6 |
| CREB5 | 1 | 0 | 1 | 1 | 14.23 | 1 | 0.10343 | 41.79008 | 7.36364 | 0 | 0 | 0 | 6 |
| PHF21A | 2 | 0 | 1 | 2 | 37.827 | 2 | 0.11636 | 53.39762 | 8.28989 | 400 | 1224 | 0 | 6 |
| UBAC2 | 1 | 0 | 1 | 1 | 24.671 | 1 | 0.10343 | 46.67897 | 7.78972 | 0 | 0 | 0 | 6 |
| PPM1K | 1 | 0 | 1 | 1 | 9.155 | 1 | 0.09309 | 36.66151 | 6.67822 | 0 | 0 | 0 | 5 |
| DENND5A | 2 | 0 | 1 | 2 | 11.647 | 2 | 0.08463 | 37.13813 | 6.59023 | 400 | 1896 | 0 | 5 |
| SDHAF2 | 1 | 0 | 1 | 1 | 15.948 | 1 | 0.10343 | 39.37024 | 7.03019 | 0 | 0 | 0 | 4 |
| ANKRD13A | 1 | 0 | 1 | 1 | 15.555 | 1 | 0.11636 | 46.11071 | 7.82676 | 0 | 0 | 0 | 4 |
| INPP5A | 1 | 0 | 1 | 1 | 32.336 | 1 | 0.10343 | 48.67659 | 7.9796 | 0 | 0 | 0 | 4 |
| USP32 | 1 | 0 | 1 | 1 | 4.912 | 1 | 0.09309 | 31.32897 | 5.79829 | 0 | 0 | 0 | 4 |
| USP15 | 1 | 0 | 1 | 1 | 36.181 | 1 | 0.11636 | 52.65595 | 8.27599 | 0 | 0 | 0 | 4 |
| EID2B | 1 | 0 | 1 | 1 | 1.349 | 1 | 0.00922 | 1 | 0.02765 | 0 | 0 | 0 | 4 |
| C12orf10 | 1 | 0 | 1 | 1 | 1.355 | 1 | 0.00922 | 1 | 0.02765 | 0 | 0 | 0 | 4 |
| LMOD3 | 1 | 0 | 1 | 1 | 1.355 | 1 | 0.00922 | 1 | 0.02765 | 0 | 0 | 0 | 4 |
